# Oligotyping reveals community level habitat selection within the genus *Vibrio*

**DOI:** 10.3389/fmicb.2014.00563

**Published:** 2014-11-13

**Authors:** Victor T. Schmidt, Julie Reveillaud, Erik Zettler, Tracy J. Mincer, Leslie Murphy, Linda A. Amaral-Zettler

**Affiliations:** ^1^Marine Biological Laboratory, Josephine Bay Paul Center for Comparative Molecular Biology and EvolutionWoods Hole, MA, USA; ^2^Department of Ecology and Evolutionary Biology, Brown UniversityProvidence, RI, USA; ^3^Sea Education AssociationWoods Hole, MA, USA; ^4^Department of Marine Chemistry and Geochemistry, Woods Hole Oceanographic InstitutionWoods Hole, MA, USA; ^5^Department of Earth, Environmental and Planetary Sciences, Brown UniversityProvidence, RI, USA

**Keywords:** oligotyping, *Vibrio* ecology, host-microbe interactions, illumina sequencing, 16S rRNA analysis, plastisphere, aquaculture pathogens, meta-analysis

## Abstract

The genus *Vibrio* is a metabolically diverse group of facultative anaerobic bacteria, common in aquatic environments and marine hosts. The genus contains several species of importance to human health and aquaculture, including the causative agents of human cholera and fish vibriosis. Vibrios display a wide variety of known life histories, from opportunistic pathogens to long-standing symbionts with individual host species. Studying *Vibrio* ecology has been challenging as individual species often display a wide range of habitat preferences, and groups of vibrios can act as socially cohesive groups. Although strong associations with salinity, temperature and other environmental variables have been established, the degree of habitat or host specificity at both the individual and community levels is unknown. Here we use oligotyping analyses in combination with a large collection of existing *Vibrio* 16S ribosomal RNA (rRNA) gene sequence data to reveal patterns of *Vibrio* ecology across a wide range of environmental, host, and abiotic substrate associated habitats. Our data show that individual taxa often display a wide range of habitat preferences yet tend to be highly abundant in either substrate-associated or free-living environments. Our analyses show that *Vibrio* communities share considerable overlap between two distinct hosts (i.e., sponge and fish), yet are distinct from the abiotic plastic substrates. Lastly, evidence for habitat specificity at the community level exists in some habitats, despite considerable stochasticity in others. In addition to providing insights into *Vibrio* ecology across a broad range of habitats, our study shows the utility of oligotyping as a facile, high-throughput and unbiased method for large-scale analyses of publically available sequence data repositories and suggests its wide application could greatly extend the range of possibilities to explore microbial ecology.

## Introduction

*Vibrio* is a ubiquitous, speciose and commercially important bacterial genus with both host associated and free-living representatives. Several species within the genus are pathogenic to humans and animals. *Vibrio cholerae* has caused six historic and one ongoing cholera pandemic, and countless epidemics (Mutreja et al., 2011) including a recent outbreak in Haiti that killed more than 8000 people (Chin et al., 2011). *Vibrio* pathogens are also important to the aquaculture industry, where they inflict costly losses on farmed fish, mollusks and shrimp (Austin and Austin, 2007), limiting the development of an industry poised to help bridge global food gaps and preserve wild fisheries (FAO, 2012). Due to their importance to human and animal welfare, and the ease with which they are cultured, vibrios are relatively well studied, with over 570 publicly available annotated genomes and over 64,000 16S rRNA gene sequences annotated as vibrios in GenBank as of March 2014. *Vibrio* therefore represents an ideal candidate for applying new analytical approaches using pre-existing data to gain further insights into the ecology of the genus.

Making sense of *Vibrio* ecology has been a challenge, owing in part to its complex life history, its capacity to partition resources, and a strong propensity for lateral gene transfer between closely related species (Hunt et al., 2008; Cordero et al., 2012). The complexity of the genus is well illustrated by the diversity of its life histories. On one hand, *Vibrio* has an average of 11 rRNA gene copies, allowing for rapid growth rates under good conditions (Heidelberg et al., 2000), suggestive of r-selected taxa, which can rapidly multiply given favorable conditions (Andrews and Harris, 1986). Conversely, bioluminescent vibrios have formed symbiotic relationships with squid and anglerfish over evolutionary time scales (Ruby and Nealson, 1976), suggestive of a more stable K-selected strategy. Some flexibility between r vs. K strategies may even exist within fine scale taxonomic categories, as environmental conditions such as pH, concentrations of bile, bicarbonate and nutrients may trigger rapid growth within a host in a formally dormant environmental bacterium (Skorupski and Taylor, 2013). To further complicate the ecology of individual *Vibrio* species, recent experiments indicate that disparate species can form socially cohesive groups, taking advantage of their propensity for exchanging genetic elements to confer greater antibiotic resistance among closely related strains, and to likely regulate virulence (Cordero et al., 2012).

Vibrios also seem to be highly variable in habitat preference. Traditionally *Vibrio* life history has been studied in association with multicellular marine hosts, including fish, mollusks, and a wide range of zooplankton (Liston, 1956; Aiso et al., 1968), yet they can also exist in the ambient aquatic environment, associated with plastic particles (Zettler et al., 2013), or phytoplankton blooms (Gilbert et al., 2012). Whether individual *Vibrio* species, or communities of vibrios, are specific to particular habitats is an open question, and distinguishing specialized associations from opportunistic colonization is challenging (Takemura et al., 2014). Host specificity has been observed in other bacterial genera, including *Blautia* (Eren et al., 2014) and *Nitrospira* (Reveillaud et al., 2014), but because *Vibrio* is abundant in both host and environmental habitats, distinguishing established host associations from incidental or ephemeral colonization from surrounding habitats is difficult.

Because vibrios are diverse in their habitat preferences and potentially act as socially cohesive units, large-scale analysis of *Vibrio* community structure across habitats may provide important insights into its ecology. Analyses of this type have historically involved culturing isolates from target habitats and sequencing multiple loci in order to gain sufficient taxonomic resolution within a sample, requiring the use of *Vibrio*-specific primers (Preheim et al., 2011a; Szabo et al., 2013), and making-large scale, non-targeted, multi-habitat analyses challenging and costly. More recently, oligotyping rRNA gene amplicon sequences affords extremely high resolution analysis of community structure by selecting a subset of highly informative nucleotide sites within single loci of 16S rRNA gene hypervariable regions alone (Eren et al., 2013a, 2014; Reveillaud et al., 2014). At the same time, repositories of 16S rRNA gene sequences have grown in size and scope. The Visualization and Analysis of Microbial Population Structures (VAMPS) database is one such repository that contains over 1000 datasets representing hundred of millions of publically available 16S rRNA gene sequences (Huse et al., 2014).

The aim of the present study was to use oligotyping to explore the distribution of *Vibrio* communities in a range of substrate-associated (both biotic and abiotic) and free-living aquatic environments. We used this method to test the hypothesis that distinct *Vibrio* communities occur in different habitats, and are characterized by clear distinctions between host habitats and their surrounding water.

## Materials and methods

### Sequence collection

The VAMPS database houses 16S rRNA gene amplicon sequence data projects from a wide variety of environmental and host-associated habitats. We identified seven existing projects to target for analyses of *Vibrio* diversity, representing free-living and host (abiotic and biotic) substrate associated habitats (Table 1). We chose projects with the occurrence of at least three samples with >300 sequences identified as *Vibrio* by the Global Alignment for Sequence Taxonomy (GAST) pipeline (Huse et al., 2008) in the VAMPS database. In rare cases, a sample was included that had less than 300 sequences in order to increase the number of samples for habitats with low sample numbers, or when water associated with a specific substrate was of interest but possessed low *Vibrio* sequence representation. All projects were sequenced at the Marine Biological Laboratory (MBL) Keck sequencing facility on an Illumina HiSeq 1000, and employed identical protocols in the generation and sequencing of 16S rRNA gene sequences, including the same primer cocktails to target the V6 hypervariable region, as described elsewhere (Eren et al., 2013b). Each project also followed the same standard MBL sequence analysis pipeline, where only perfectly overlapping paired-ends reads with zero mismatches passed quality filters (Eren et al., 2013b), and taxonomic assignment was done using the GAST pipeline (Huse et al., 2008). Both quality filtering and taxonomic assignments had already been made for all sequences across all projects as part of standard VAMPS protocols, and we were therefore able to directly download sequences using VAMPS's “data export -> TaxBySeq” feature, using the query “Bacteria; Proteobacteria; Gammaproteobacteria; Vibrionales; Vibrionaceae; *Vibrio*.”

**Table 1:** **Overview of projects used from the VAMPS database with their original citation**.

**VAMPS project**	**Habitat**	**Sample number**	**Mean *Vibrio* relative abundance**	**Geographic location**	**Salinity**	**Citation or SRA BioProject accession number**
ICM_PML_Bv6	Seawater	3	0.31 (SE 0.13)	English Channel	Marine	Gilbert et al., 2012
LAZ_MHB_Bv6	Seawater	14	0.0017 (SE 0.00021)	Northwestern Atlantic	Marine	SRP049014
LAZ_NMS_Bv6	Saltmarsh	11	0.173 (SE 0.019)	New England, USA	Mixed	SRP059013
SLM_NIH_Bv6	PAH spiked sand	11	0.012 (SE 0.002)	Gulf of Mexico	Mixed	Kappell et al., 2014
LAZ_SEA_Bv6	Seawater - associated with plastic	32	0.0036 (SE 0.00058)	Northwestern Atlantic	Marine	SRP026054 %but see Zettler et al., 2013
LAZ_SEA_Bv6	Plastic-associated	27	0.0032 (SE 0.0005)	Northwestern Atlantic	Marine	SRP026054 %but see Zettler et al., 2013
JCR_SPO_Bv6	Seawater - associated with sponge	11	0.055 (SE 0.023)	Northeastern Atlantic	Marine	Reveillaud et al., 2014
JCR_SPO_Bv6	Sponge-associated	49	0.09 (SE 0.014)	Northeastern Atlantic	Marine	Reveillaud et al., 2014
VTS_MIC_Bv6	Aquarium water - associated with fish	31	0.016 (SE 0.0037)	MBL, Woods Hole, USA	Mixed	SRP047374 (but see Supplementary Data Sheet 1)
VTS_MIC_Bv6	Fish-associated	20	0.3 (SE 0.039)	MBL, Woods Hole, USA	Mixed	SRP047374 (but see Supplementary Data Sheet 1)

*The mean Vibrio relative abundance across all samples in a given project is shown with standard error. For sequences first published by this study the accession numbers for that project's NCBI Sequence Read Archive (SRA) BioProject is given*.

Although sequence generation was identical for all projects, sample collection varied. Saltmarsh water sample collection (LAZ_NMS_Bv6, this study) employed automated collection via the Phytoplankton Sampler (PPS) (McLane Research Laboratories, Inc., East Falmouth, MA) that filters 500 mL of water through a 0.65 μm flat filter (EMD Millipore Durapore PVDP hydrophilic membrane filters) (Billerica, MA) twice a day, and stores the filter in RNAlater (Qiagen, Valencia, CA) buffer. As part of a broader project to understand microbial populations in coastal environments, we deployed PPS samplers in tidal creeks (Mill and Salt Ponds, Nauset Marsh System, MA) that receive daily tidal fluxes from the Atlantic Ocean off Cape Cod, MA. DNA extraction and purification of filters used a modified salt precipitation method with bead-beating (Gentra Puregene, Qiagen, Valencia, CA). The Rhode Island Department of Environmental Management (RIDEM) collected seawater samples from the northeastern reach of Narragansett Bay called Mount Hope Bay, MA (LAZ_MHB_Bv6) as part of their monthly water quality survey for shellfish safety. Samples collected manually from surface waters in sterile 1 L polyethylene terephthalate (PET) bottles at 17 stations throughout the 36 km^2^ bay were subsequently filtered through 0.22 μm polyethersulphone membrane Sterivex filters (Millipore, Billerica, MA) followed by DNA extraction as above. Collection details for other samples and metadata are found in respective publications (Table 1).

Samples from fish and fish tanks (VTS_MIC_Bv6) were collected as part of an experiment to understand the role of salinity and external microbiota on fish microbiomes (Schmidt et al., Submitted) (Supplementary Data Sheet 1). We acclimated ~1 inch Black Molly fish (*Poecilia sphenops*) to four salinity levels (salinities 0, 5, 18, and 30) over 30 days using nanopure water and Instant Ocean® (Blacksburg, VA) salt mix, then maintained each fish at target salinity for 12 days. Each salinity treatment contained four independent tanks, each with two fish. For our analyses here, we grouped salinities 0 and 5 (FreshwaterFish) and salinities 18 and 30 (MarineFish). After 12 days we euthanized fish in 1 mg/mL MS-222 and homogenized the entire fish. We then extracted microbial gDNA from the homogenate using a modified Gentra Puregene Yeast/Bac (Qiagen, Valencia, CA) extraction protocol (Supplementary Data Sheet 1). We collected microbial communities from tank water using sterile 1 L PET bottles, and extracted gDNA according to protocols outlined above for LAZ_MHB_Bv6 samples.

### Oligotype generation and analysis

Oligotyping is a supervised method that allows the identification of closely related but distinct bacterial taxa in high-throughput sequencing datasets of marker genes. This novel bioinformatics approach is capable of uncovering ecological patterns of microbial communities at finer scales than previously possible with *de novo* approaches (Eren et al., 2013a). Oligotying exploits the fact that some positions within a DNA marker sequence are more ecologically informative than others. The method identifies highly variable locations using Shannon entropy (that is, “entropy components”), and uses only these positions to discriminate ecological units, so called oligotypes. This process reduces the impact of noise caused by sequencing error by relying on only a small number of nucleotide positions, discarding the redundant parts of reads for the identification of oligotypes. The open-source pipeline for oligotyping is available from http://oligotyping.org. This method has been used previously to identify *Gardnerella* distributions in vaginal samples (Eren et al., 2011), *Nitrospira* specificity in sponges (Reveillaud et al., 2014), and *Blautia* specificity in animal hosts (Eren et al., 2014).

In order to get the best possible insights into *Vibrio* oligotype distributions across habitat types, we grouped samples into three broad analysis groupings; substrate (host) associated habitats only, substrate habitats along with their surrounding water samples, and environmental and substrate associated habitats (Table 2). First, we analyzed only substrate-associated samples (fish, sponge and our abiotic substrate—plastic marine debris). We subsampled these datasets to the median *Vibrio* sequence count of 30,000 prior to analysis in order to minimize the range in initial sequencing depth between samples, which can otherwise reduce the entropy value of discriminating points in datasets with much lower sequence counts. We then processed them through the oligotyping pipeline, as described in Eren et al. (2013a). To establish entropy components that fully decomposed our sequence data, we started with the strongest two components, then manually chose the next component that best removed remaining entropy in the resulting oligotypes, and re-ran the analysis. This iterative process yielded a final 12 entropy points that fully decomposed our sequences. We allowed for oligotypes to occur in only a single sample (-s 1) but discarded them if they did not represent at least 0.5% of the relative abundance of that sample (–a 0.5). Not all samples contained 30,000 sequences, and sequencing depth ranged from 83 to 30,000 after rarefaction (Table 2).

**Table 2:** **Description of samples for each analysis grouping**.

**Analysis grouping**	**Analysis grouping/Figure**	**Number of samples**	**Subsampled sequence depth range**	**Oligotypes before (after) quality filtering**	**Percentage of reads represented by top 5% (10%) oligotypes**	**Components (position in alignment)**
All substrates	1	104	83–30,000	882 (74)	76 (94)	13, 15, 20, 21, 22, 23, 25, 31, 32, 45, 50, 55
Plastics and surrounding seawater	2C	71	83–13,359	415 (71)	90 (96)	13, 15, 20, 21, 22, 23, 25, 31, 32, 45, 50, 55
Sponges and surrounding seawater	2B	58	1822–10,000	681 (45)	71 (90)	13, 15, 20, 21, 22, 23, 25, 31, 32, 45, 50, 55
Fish and surrounding water	2A	51	636–85,000	604 (21)	80 (97)	13, 15, 20, 21, 22, 23, 25, 31, 32, 45, 50, 55
Mixed habitat	4	179	83–20,000	1452 (99)	65 (90)	13, 15, 20, 21, 22, 23, 25, 31, 32, 37, 45, 50, 55

*Some samples were included in more than a single grouping*.

Global alignment prior to oligotyping for short Illumina reads is unnecessary, as positional shifts in sequencing reads due to natural indels will produce entropy peaks at the position of insertion or deletion (and subsequent positions), and the decomposition of the dataset based on any of these peaks will eventually result in the same oligotypes as if they would have been previously aligned. Furthermore, V6 primers target a hypervariable region with few insertions or deletions, and Illumina technology does not have indel error issues. We therefore did not create an alignment prior to entropy analyses. Instead, we aligned sequences at their 3′ end and padded any length discrepancies with gaps at the 5′ end prior to entropy analysis, as detailed in Eren et al. (2013a). We do note that a single Oligotype, Oligotype 10, was not fully decomposed (Supplementary Data Sheet 1). We note that this oligotype varies widely from all other *Vibrio* sequences in this study, and would require an additional 4 entropy components to fully decompose. Furthermore, it occurred in high abundance only in the Sand-PAH habitat. We make no conclusions about this oligotype across any habitat.

Next, we examined each substrate or host sample alongside its respective water sample. For fish and plastics, water samples were directly associated with the host, and collected at the same time and place as host material. Sponge and corresponding seawater were collected simultaneously, although not all sponge samples have a corresponding water samples (see Reveillaud et al., 2014). For each analysis, we subsampled *Vibrio* sequences to the median sequencing depth. The same 12 entropy components and oligotyping parameters as above fully decomposed all but one oligotype, and were therefore used again in this analysis.

We then analyzed oligotype distributions across the broadest range of samples and projects included in this study in a single analysis using the same methods and 12 entropy components, with one additional component added to fully resolve novel oligotypes from additional samples (13 components total). The added samples included water samples from saltmarshes (Saltmarsh), seawater from a large coastal bay (Seawater), open ocean seawater (Seawater), and sand samples from oiled beaches in the Gulf of Mexico inoculated with Polycyclic Aromatic Hydrocarbons (PAHs) (Sand-PAH) (Tables 1, 2). As above, samples were subsampled down to the median value of 20,000. An interactive html file of the results from this oligotyping analysis grouping (Mixed Habitat) is included in the Supplementary Material under “html_files/html.index” (Supplementary Data Sheet 2).

Finally, we grouped the representative sequences from the 10 most abundant oligotypes (30 total) from each analysis grouping. Since most oligotypes were abundant across multiple projects, this list collapsed into 17 unique oligotypes across all three analysis runs. These 17 oligotypes were assigned identifiers (“Oligotype1” through “Oligotype17”) that remained consistent across all three runs (Table 3 and Table S1).

**Table 3:** **Summary of the 10 most abundant oligotypes from each of three oligotyping analysis groupings**.

	**MEGABLAST results**		**Percentage of each isolation source category for MEGABLAST hits**						
		
**Oligotype ID**	**Number of 100% hits to nr database**	**Top species level hits (number of hits to that species)**	**1**	**2**	**3**	**4**	**5**	**No data**	**Habitats with Similarity Percentage (SIMPER) results > 10%**
Oligotype 1	22	*V. alfacsensis* (6) *V. sinaloensis* (2)	45.5	9.1	40.9	0.0	0.0	4.5	Sponge/MarineFish/MarineWater
Oligotype 2	171	*V. metschnikovii* (3) *V. neptunius* (11)	42.9	15.3	12.4	11.2	0.0	18.2	FreshwaterFish/FreshWater
Oligotype 3	780	*V. scophthalmi* (4) *V. ichthyoenteri* (31) *V. anguillarum* (37)	55.7	11.4	6.9	0.1	0.0	25.9	MarineFish/MarineWater/Seawater/Saltmarsh/Plastic
Oligotype 4	39	*V. cholerae* (9) *V. vulnificus* (17) *V. mimicus* (5)	2.6	0.0	0.0	0.0	30.8	66.7	FreshwaterFish/FreshWater/Sponge/MarineFish
Oligotype 5	1000^*^	*V. splendidus* (52) *V. mediterranei* (35) *V. gigantis* (39)	45.3	21.4	8.1	0.1	0.0	25.1	Sand-PAH/Seawater/Saltmarsh/Plastic
Oligotype 6	23	*V. ichthyoenteri* (1) *V. ordalii* (1)	70.8	8.3	0.0	0.0	0.0	20.8	Sponge/MarineFish/MarineWater
Oligotype 7	46	*V. ponticus* (12) *V. nigripulchritudo* (9)	30.4	8.7	4.3	0.0	0.0	56.5	Seawater
Oligotype 8	221	*V. cholerae* (184)	1.4	1.4	0.5	3.6	11.8	81.4	FreshwaterFish/FreshWater
Oligotype 9	1	*V. vulnificus* (1)	0.0	0.0	0.0	0.0	100	0.0	
Oligotype 10	18	*V. alginolyticus* (1)	11.1	0.0	0.0	89.0	0.0	0.0	Sand-PAH
Oligotype 11	1	None	0.0	0.0	0.0	0.0	0.0	100	
Oligotype 12	113	*V. coralliilyticus* (2)	27.4	39.8	0.0	0.0	0.0	32.7	Seawater/Plastic
Oligotype 13	66	*V. azureus* (13) *V. harveyi* (2)	54.5	6.1	0.0	11.6	5.5	22.7	Plastic
Oligotype 14	0	*V. azureus* (5) *V. owensii* (2)	0.0	0.0	0.0	0.0	0.0	100.0	
Oligotype 15	245	*V. vulnificus* (4) *V. shilonii* (3)	94.5	2.4	0.4	0.0	0.0	3.4	
Oligotype 16	140	*V. kanaloae* (3) *V. splendidus* (2)	42.1	14.3	11.4	2.1	0.0	30.0	
Oligotype 17	6	*V. splendidus* (3)	84.0	0.0	0.0	0.0	0.0	16.0	

*Overlap in the most abundant sequences between groupings reduced the total number to 17. Oligotype names were assigned arbitrarily, but are consistent across all groupings. The species assignments given by reports from 100% MEGABLAST hits, and the number of hits to each species, is shown. The proportion of MEGABLAST hits isolated from each of the five habitat categories are also shown. Categories are; 1. Marine Host, 2. Seawater, 3. Other Marine, 4. Terrestrial or Human, and 5. Freshwater (see Materials and Methods)*.

(*) *Indicates maximum requested hits*.

### Visualizations and statistical analyses of oligotype distributions

To visualize *Vibrio* community similarity between samples and habitats we constructed Nonmetric Multidimensional Scaling (NMDS) plots as part of our oligotyping pipelines. We included the covariance ellipsoids calculated as part of the oligotyping pipeline on these plots to visualize the spread of a given habitat's community variance. Importantly, covariance ellipsoids delineate the total high-dimensional space, not only the two axes shown in the NMDS plot. Statistical analyses of *Vibrio* oligotype distributions between and within habitat types followed a 4-step analysis using the ecological statistics packages PrimerE v.6 and the R package Vegan (Oksanen et al., 2012). First, we normalized an oligotype matrix, which consisted of samples across rows and oligotypes down columns (Supplementary Data Sheet 2), by percent per sample (i.e., to 100% total for each sample) and calculated pairwise Bray-Curtis similarities. Second, we assigned each sample to a habitat “factor” based on where it was collected (e.g., on plastics, sponges or seawater) and tested the null hypothesis that there were no community differences between habitat types using Analysis of Similarity (ANOSIM) permutation tests. This test builds a random distribution of oligotype abundances using 9999 permutations then assesses the likelihood that observed oligotype distributions across *a priori* assigned habitat factors occurred by chance. We then conducted pairwise ANOSIM tests to determine whether significant differences occurred between individual habitat factors. Third, when statistical groupings did occur (as they did in most cases), we identified the oligotypes that contributed most to the formation of these groupings using Similarity Percentages (SIMPER). SIMPER decomposes average Bray-Curtis similarities between all pairwise habitat comparisons into percentage contributions of each oligotype.

To gain insight into the taxonomy and ecology of our oligotype sequences, we isolated the representative sequence from the 17 most abundant oligotypes outlined above. We then used MEGABLAST to query these sequences against National Center for Biotechnology Information's (NCBI) nr database in June 2014 (nr = non-redundant amalgamation of GenBank, RefSeq, EMBL, DDBJ and PDB databases). We kept only 100% matches across the entire 60 bp query, and extracted the “isolation source” and “host” feature using Geneious (v. 6.1) annotation tables. We also extracted the most abundant 2 or 3 taxonomies from perfect hits, not including “uncultured bacterium” (Table 3). We created a PhyML tree of existing full-length *Vibrio* 16S rRNA gene sequences downloaded from type strains in the SILVA ARB v5.1 database and then added our oligotype sequences to this tree using the Maximum Parsimony feature in ARB across the V6 region only (using a V6 “filter” in ARB).

Our rationale for building this tree was not to reconstruct phylogenetic relationships between *Vibrio* species but rather to make some inference about the habitats from which closely related *Vibrio* 16S rRNA gene sequences have been isolated. To this end, we binned the isolation source and host annotations of both our oligotype MEGABLAST hits, and our ARB isolates, into five habitat categories. These were “Marine Host,” “Seawater,” “Other Marine,” “Terrestrial,” and “Freshwater.” Marine host included all sequences isolated from the skin or innards of a host in seawater (e.g., tunicates, fish, crustaceans, sponges and sea cucumbers). “Other Marine” included sediment, biofilms and algae, or other marine plant associated sources. “Terrestrial” included any sample taken from the terrestrial environment, or from a terrestrial host (e.g., humans, birds, and plants), and freshwater included hits isolated from a freshwater environment, or freshwater host (e.g., freshwater shrimp). We color-coded the proportion of each category and displayed them at the terminal nodes of each oligotype sequence in our PhyML reference tree. Lastly, to visualize the isolation source of the ARB isolate reference sequences, we color-coded the nodes of the tree according to the isolation source of each reference sequence at the terminal node of that branch. We used the online software Interactive Tree of Life (iTOL) (Letunic and Bork, 2011) to visualize the phylogenetic tree. The proportion of NCBI hits for each oligotype that fit into each category also appear in Table 3.

### “Within-habitat variance” of *Vibrio* communities

To determine differences in habitat specificity, we calculated the median *Vibrio* community variance across all datasets within the same habitat (its “within-habitat variance”), and compared that across habitats. This allowed us to determine if some habitats contained a specific *Vibrio* community, or if *Vibrio* communities varied widely even within the same habitat. To calculate the median variance of all datasets in a habitat, we normalized our oligotype abundance matrixes by the maximum value of each oligotype, then calculated Bray-Curtis community similarity between all pairwise comparisons using vegdist{vegan} function in R (Oksanen et al., 2012). We then calculated each habitat's multidimensional “centroid” using the median value of each sample within a habitat across all principal components. The distance of each sample to its habitat centroid was calculated across all principal components. The variance around the median value of sample-centroid distances was then compared across habitats in a standard ANOVA, followed by pairwise Tukey's Honestly Significant Difference (HSD) tests. This entire process, from centroid calculation to HSD tests was implemented using the betadisper{vegan} function in R. To visualize our results, we plotted each sample along their first two principal components, and plotted the multidimensional centroid. We then drew covariance ellipsoids around each habitat to illustrate the median distance for all samples in a habitat around its centroid. Median sample-centroid distances for each habitat were also plotted to better visualize the within-habitat variance.

## Results

### Oligotype distribution across biotic and abiotic substrates

Oligotyping analysis of substrate associated-habitats (fish, sponges, plastics) yielded 74 unique oligotypes across 104 samples from 1,543,415 initial sequences. The minimum relative abundance threshold removed 808 rare oligotypes. The most abundant 5 oligotypes represented 76% of the reads, with the top 10 representing 94% (Table 2). Oligotypes that were abundant in at least one sample (>1% relative abundance) were always found across all three substrate types, meaning abundant oligotypes were ubiquitous across all host-associated habitats. Oligotype richness varied across host/substrate type. Of the 74 total oligotypes, 24.1 ± SE 2.2 were found in FreshwaterFish, 27.0 ± SE0.71 in MarineFish, 31 ± SE 0.9 in Sponge and only 18 ± SE1.5 for Plastic samples.

**Figure 1:**
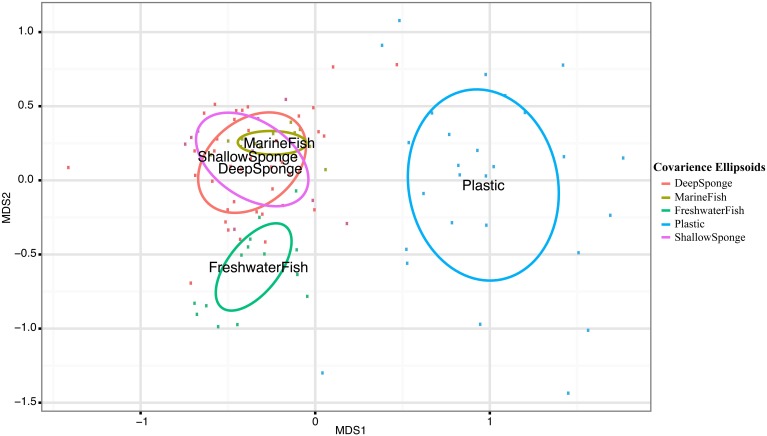
**Nonmetric Multidimensional Scaling (NMDS) plot of host-associated *Vibrio* communities based on oligotype distributions**. Labels are located at the center of covariance ellipsoids around each host type. Pairwise ANOSIM permutation tests reveal all host habitats can be significantly differentiated except MarineFish and Sponge communities (Table S1).

Pairwise Analysis of Similarity (ANOSIM) tests showed significant groupings in oligotype communities according to habitat, except between high salinity fish (MarineFish) and sponges, whose communities could not be significantly distinguished. This ANOSIM result is also visible in our NMDS analyses which shows clear overlap between both sponges (DeepSponge and ShallowSponge) and high salinity fish (MarineFish), yet separation from plastic and low salinity fish (FreshwaterFish) habitats (Figure 1). An ANOSIM test comparing marine biotic substrates (MarineFish and Sponges pooled together) revealed a significant grouping that excluded Plastic, an abiotic substrate. Similarity Percentages (SIMPER) analysis corroborated these results by illustrating the strong contribution of Oligotypes 1, 4, and 6 to both MarineFish and Sponge within-habitat similarity, and distinguished those habitats from FreshwaterFish and Plastics. Oligotypes 2, 4, and 8 contributed to both within habitat similarity, and between habitat differences for FreshwaterFish samples. Plastics were dominated by Oligotype 5, which also distinguished it from other habitat types, including biotic substrates (Tables 4A,B).

Table 4**(A,B) SIMPER analysis output for the “All substrate” analysis grouping**.

**Table:** 

**(A) Within habitat markers**							
**FreshwaterFish**	**Cont (%)**	**MarineFish**	**Cont (%)**	**Sponge**	**Cont (%)**	**Plastic**	**Cont (%)**
Oligo2	72.85	Oligo1	32.85	Oligo1	37.45	Oligo5	69.9
Oligo4	11.61	Oligo6	20.49	Oligo6	18.67	Oligo3	11.07
Oligo8	6.85	Oligo3	15.16	Oligo4	15.73	Oligo15	5.53
		Oligo4	12.85	Oligo2	6.36		
		Oligo7	7.31	Oligo8	5.43		
		Oligo5	4.98	Oligo7	4.54		
				Oligo3	2.7

**Table:** 

**(B) Between habitat markers**					
**FreshwaterFish and MarineFish Average dissimilarity = 83.6**		**FreshwaterFish and Sponge Average dissimilarity = 76.16**		**FreshwaterFish and Plastic Average dissimilarity = 90.26**	
Oligo2	33.64	Oligo2	33.5	Oligo2	30.08
Oligo1	15.51	Oligo1	14.93	Oligo5	24.16
Oligo6	11.57	Oligo8	12.8	Oligo8	8.38
Oligo4	11.1	Oligo4	11.67	Oligo4	8.02
Oligo8	8.72	Oligo6	9.46	Oligo15	5.21
Cumulative	80.54	Cumulative	82.36	Cumulative	75.85
**MarineFish and Sponge Average dissimilarity = 63.21**		**MarineFish and Plastic Average dissimilarity = 85.02**		**Sponge and Plastic Average dissimilarity = 89.48**	
Oligo1	19.77	Oligo5	23.41	Oligo5	23.33
Oligo6	16.14	Oligo1	16.07	Oligo1	13.31
Oligo4	15.01	Oligo6	12	Oligo6	8.46
Oligo8	8.77	Oligo4	9.34	Oligo4	8.18
Oligo2	8.45	Oligo7	7.04	Oligo2	7.64
Cumulative	68.14	Cumulative	67.86	Cumulative	60.92

*A: The percent contribution of each oligotype to within-habitat Bray-Curtis similarity is shown (Cont%). B: The percent contribution of each oligotype to Bray-Curtis dissimilarities between two habitats is shown, along with average Bray-Curtis dissimilarities*.

### Oligotype distributions between substrates and their surrounding water

Isolating individual biotic (hosts) and abiotic substrates along with their surrounding water allowed for direct comparisons of attached vs. free-living *Vibrio* communities. Fish hosts (FreshwaterFish and MarineFish) on average showed a nearly 20-fold enrichment of total *Vibrio* relative abundance compared to their surrounding water (30% ± SE 2.9 in fish vs. 1.6% ± SE 0.37 in water, Table 1), but samples from sponges or plastic showed no significant enrichment [although a non-significant trend of enrichment was evident for Sponge habitats (Table 1)]. Despite this enrichment in fish hosts, we could not differentiate *Vibrio* community structure in fish microbiome samples and their surrounding environment with ANOSIM analyses, so long as comparisons were made within the same salinity category (Marine and Freshwater).

**Figure 2:**
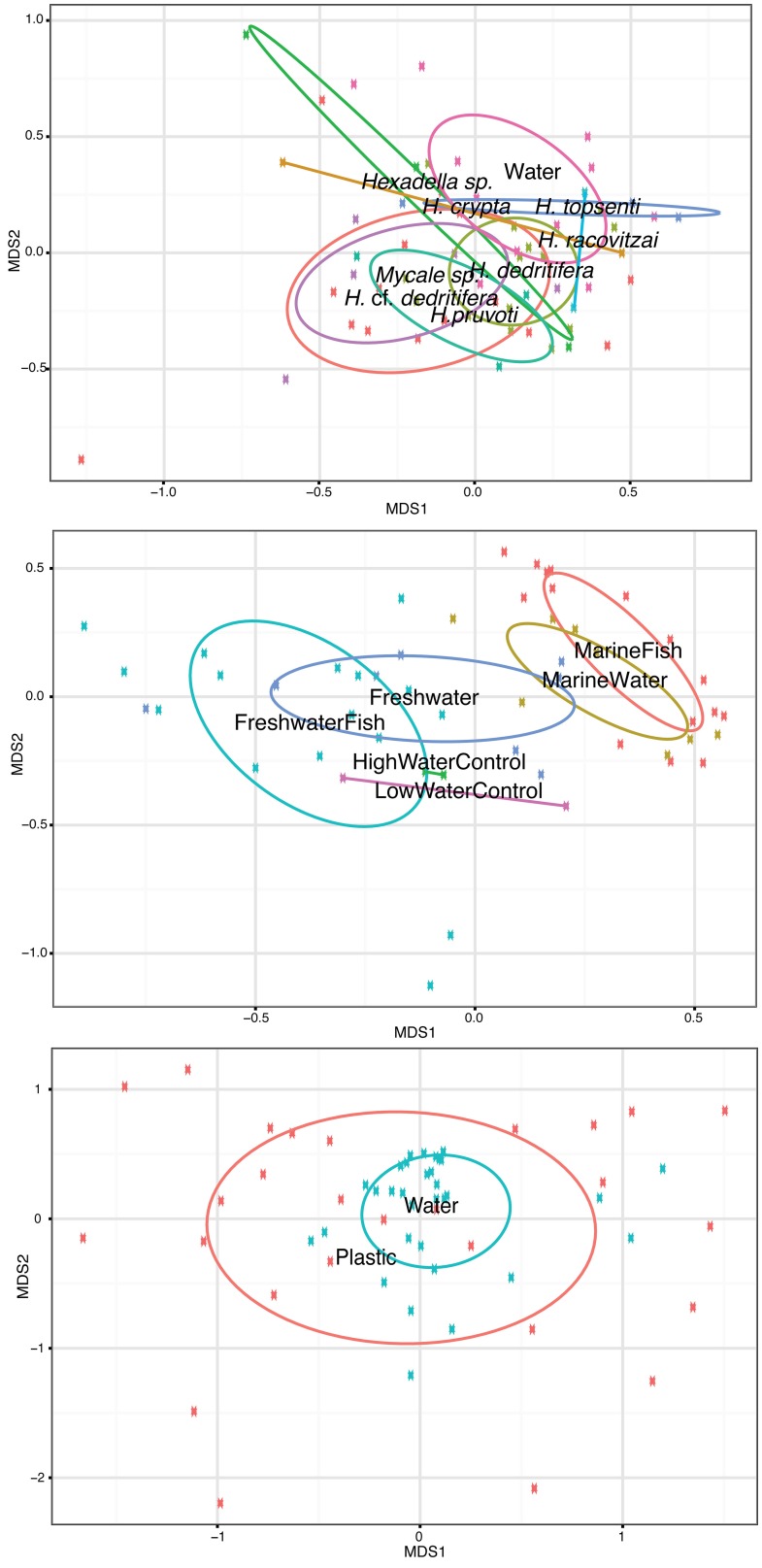
**Samples from the three host-associated studies included in this meta-analysis separated out into individual NMDS plots alongside water samples collected as part of the same study**. **Top:** Sponges are labeled according to species. Water was collected next to *Mycale* sp. and *Hexadella* cf. *dedritifera* samples only. **Middle:** Fish water was sampled directly from aquaria in which fish were housed, in addition to “control” water that was sampled from aquaria at an identical salinity, but without any fish. **Bottom:** Ocean surface water was collected at the same time and location as corresponding plastics. ANOSIM permutation tests show that Fish, Sponge and Plastic associated *Vibrio* communities cannot be statistically separated out from their surrounding water environment. Labels for each habitat are within their respective covariance ellipsoid.

**Figure 3:**
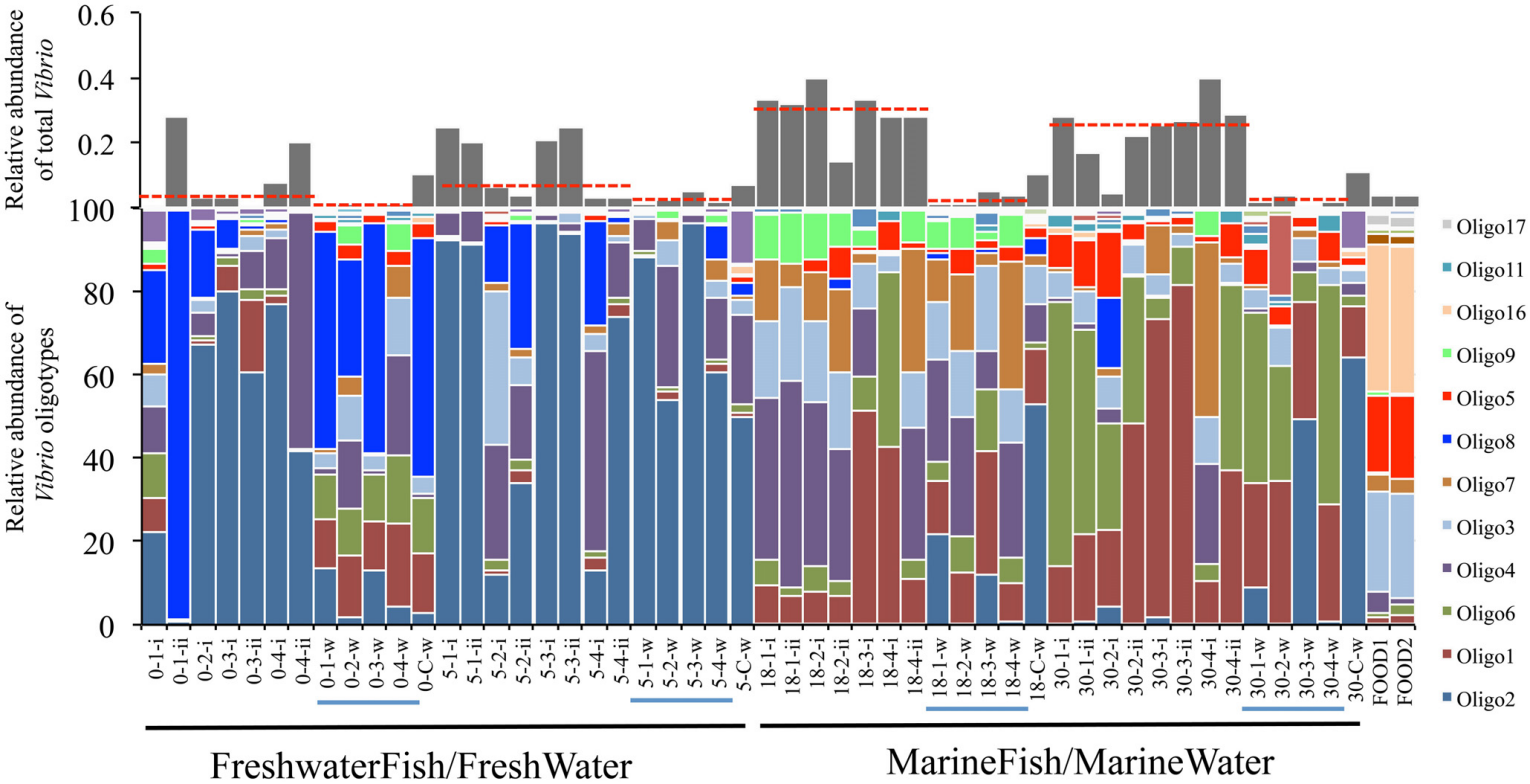
**Oligotype distribution for FreshwaterFish and MarineFish tissue (FreshwaterFish: 0-x-i and 5-x-ii, MarineFish: 18-x-i and 18-x-ii), associated water samples (x-x-W, blue lines), and water samples from aquaria containing no fish (x-C-W)**. The relative abundance of each oligotype within the total *Vibrio* diversity for each sample is shown in stacked bar graphs (bottom), and the proportion of the total *Vibrio* (relative abundance) within all bacterial diversity for each sample is shown with gray bars (top, red-dashed lines are median values for each host-habitat). A clear division between low and high salinity samples is seen, despite considerable variation within salinities. Significant differences in median total *Vibrio* relative abundance exists between FreshwaterFish and MarineFish samples, and between MarineFish samples and their surrounding water. The *Vibrio* community of fish food used during experimental period is also shown (“FOODX”).

Interestingly, although *Vibrio* communities between fish and their surrounding water at a given salinity were statistically indistinguishable, communities between fresh and marine salinity environments showed dramatic differences in both community structure and relative abundance of total *Vibrio* (Figure 2, **Middle**). Oligotypes 2, 4, and 8 dominated both FreshwaterFish and FreshWater (cumulative abundance in FreshwaterFish/FreshWater = 87.2%/71.9%), while Oligotypes 1, 3, and 6 dominated MarineFish and MarineWater (cumulative abundance in MarineFish/MarineWater = 60%/53% (Figure 3). Experimental aquaria without fish at low salinity (water only) showed a strong dominance of Oligotype 2, 4, and 8 (cumulative abundance = 67%), showing strong similarities to those aquaria that did house fish. However, marine aquaria without fish showed a strong dominance of Oligotype 2, inconsistent with marine aquaria that did contain fish. Interestingly, Oligotype 4 was found across all salinities, in water, fish and control samples, at greater than 10% relative abundance.

Sponge associated *Vibrio* communities were also statistically indistinguishable from their surrounding water (ANOSIM *P* = 0.31), although this may have been in part due to the sample size difference between sponge and associated seawater samples (Sponge = 49, Associated seawater = 11), and the high variability of particular oligotypes in some Sponge samples. Sponges showed significantly smaller relative abundance of Oligotype 7 and 5, and significantly increased abundance of Oligotypes 8 and 2 (Pairwise *T*-tests *P* < 0.01 in all cases). Sponge associated *Vibrio* communities also showed no clear groupings according to species (Figure 2, **Top**).

**Figure 4:**
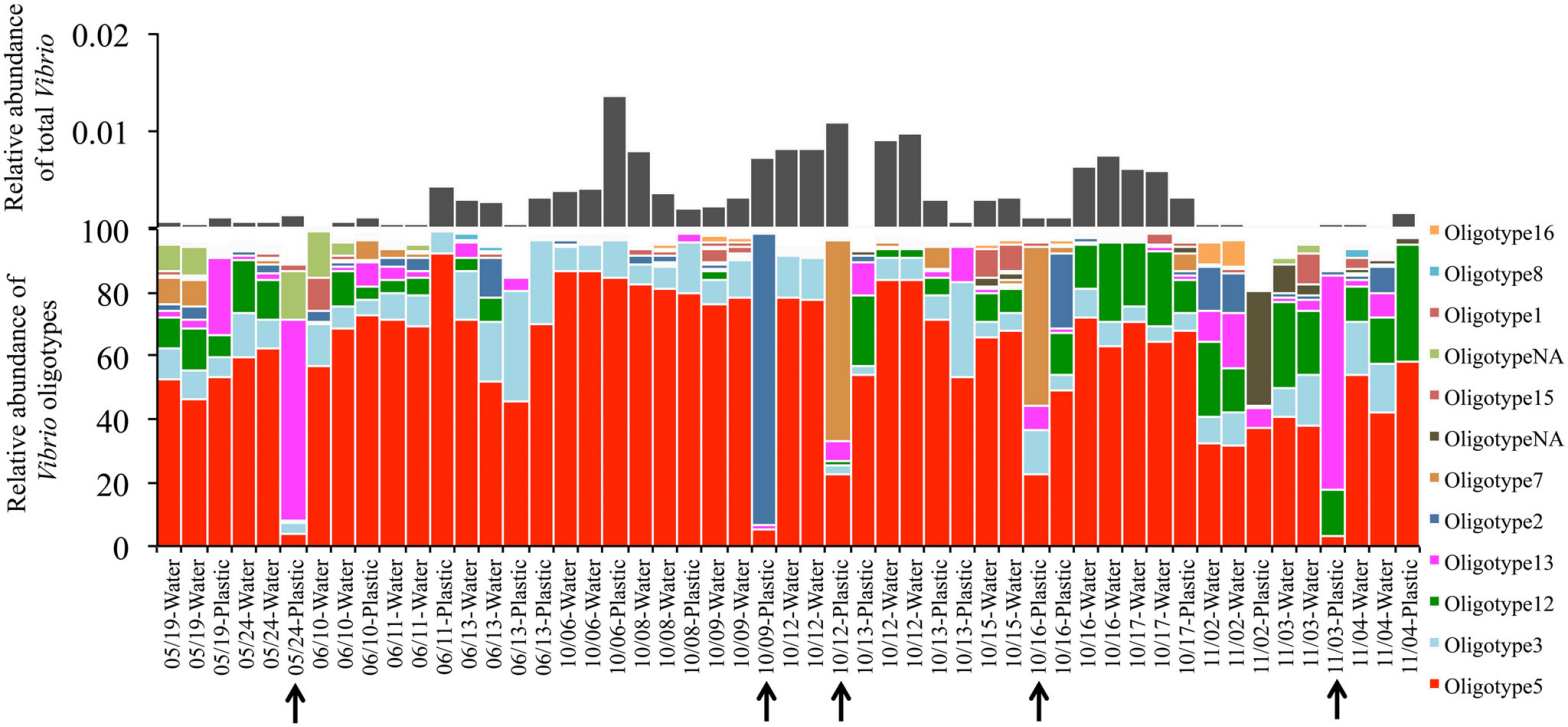
**Oligotype distributions for Plastic samples and their surrounding water**. Sample labels indicate the date the samples were collected and the sample type. Water and plastic samples collected on the same date are associated with one another. All water was collected at the surface. Black arrows indicate plastic samples that contain a single oligotype at greater than 50% relative abundance. “OligotypeNA” represents an oligotype that was not among the top 10 most abundant oligotypes from any three of our oligotype groupings.

Lastly, *Vibrio* communities from plastic substrates overlapped completely with seawater communities collected alongside them (Figure 2, **Bottom**), and Oligotypes 3, 5, and 12, dominated both Plastics and associated seawater. We found no oligotype to be significantly enriched on plastic samples compared to their surrounding water, nor were there significant increases in total *Vibrio* on plastic substrates. In several cases, we found a single oligotype that did not occur in the surrounding water but dominated in relative abundance on an individual plastic substrate. This pattern was particularly apparent with Oligotypes 13, 2, and 7, which reached extremely high relative abundances on multiple occasions (e.g., Oligotype 2 at 91% relative abundance in the “10/09-Plastic” sample) (Figure 4).

### Oligotype distributions across broad habitat types

**Figure 5:**
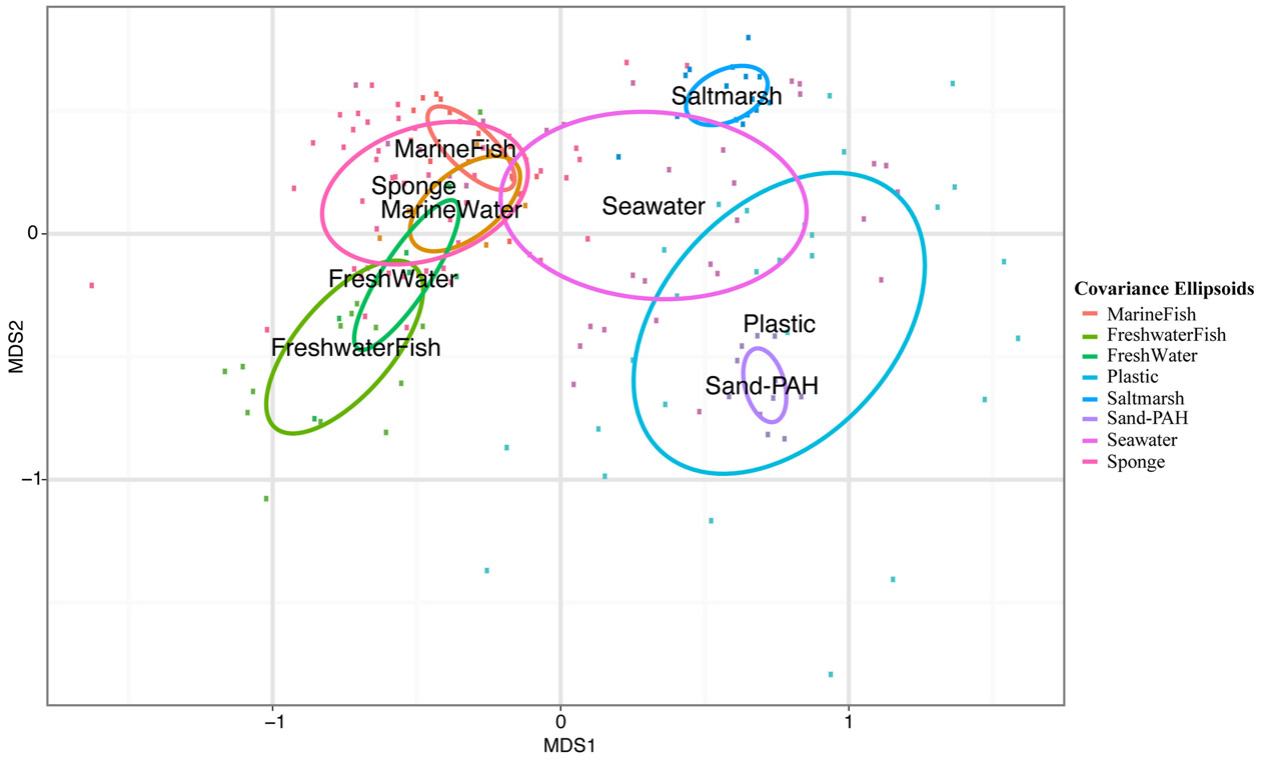
**NMDS plot with covariance ellipsoids for both host-associated and environmental samples**. Sample names refer to habitat type and VAMPS project listed in Table 1.

In order to gain as broad a view as possible of *Vibrio* oligotype distributions across habitats, we included 179 samples from 7 environmental and host-associated habitats spanning a wide range of environmental and geographical gradients (Figure 5). This analysis yielded 99 oligotypes, of which the top 5 represented 65% of all reads, while the top 10 represented 90%. We observed the top 10 oligotypes from this analysis at high abundance in previous analyses (as determined by identical representative sequences), except Oligotype 10, which was novel to Sand-PAH mesocosms and is a highly divergent oligotype which could not be fully resolved. Sand-PAH samples were taken from beach sand communities near the Deepwater Horizon oil spill, and were likely enriched for PAH-associated species (Kappell et al., 2014).

**Figure 6:**
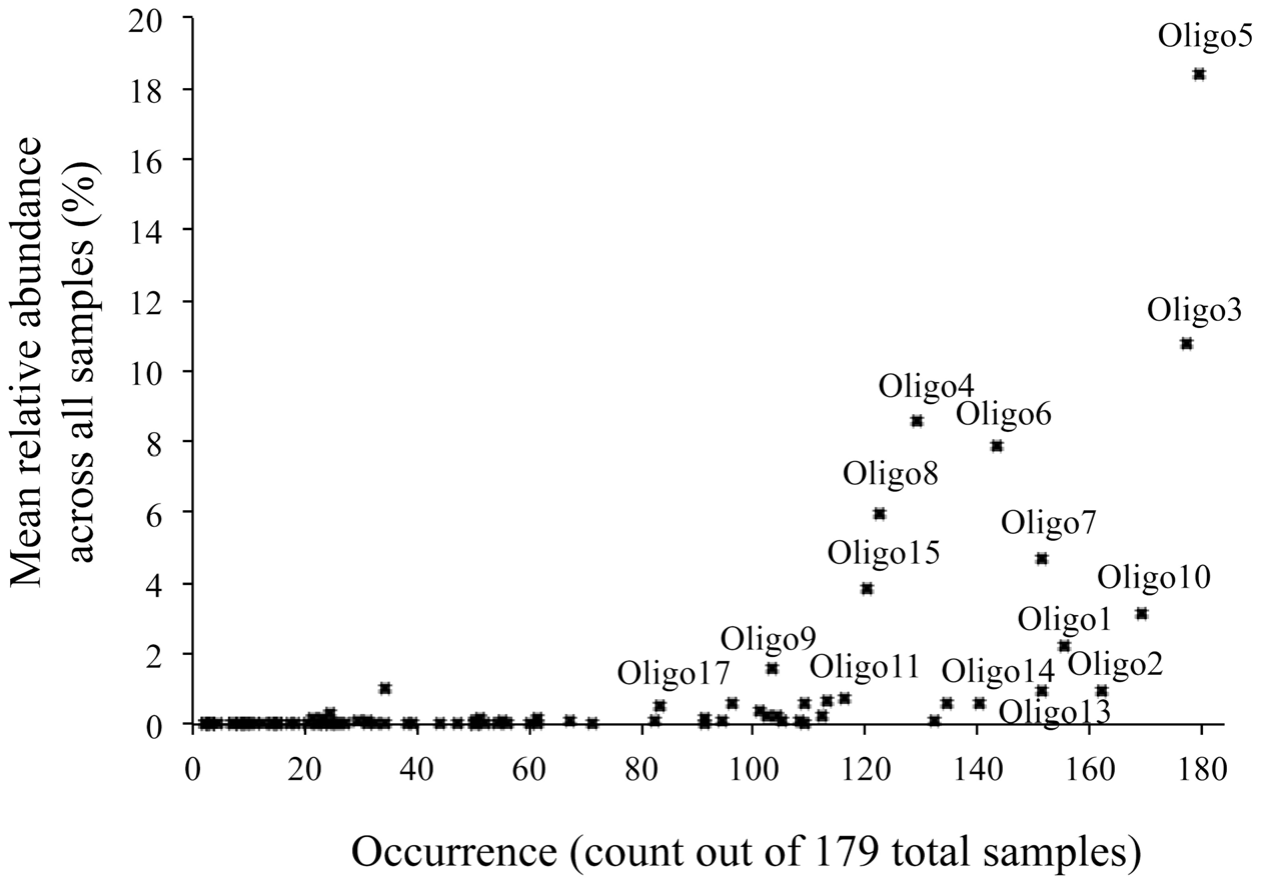
**Commonness and abundance plot of all oligotypes that were part of the mixed habitat sample grouping analysis (Table 2)**. The occurrence (presence/absence) of each of the 99 oligotypes across all 179 samples is plotted along the x-axis while its mean relative abundance across all samples is plotted on the y-axis. Samples that are both common and abundant are found in the top right, while those that are common, but rare are in the bottom right. Both rare and uncommon are found in the bottom left. The top 17 most abundant oligotypes from Table 3 are also labeled.

All oligotypes that were highly abundant in a single sample (>10% relative *Vibrio* abundance) occurred across all other habitat types. Abundant oligotypes were therefore also likely to be common across a wide variety of habitat types (Figure 6). Oligotype 5 in particular was found to be both highly abundant and frequent, occurring in all 179 samples analyzed across all habitats (Figure 6). Several oligotypes did not follow this general pattern, and despite a relative ubiquity, they maintained at low mean relative abundances across all the samples in which they occurred (e.g., Oligotypes 1, 2, 13, and 14, Figure 6). Comparing the mean relative abundance of individual oligotypes between marine hosts (Sponge, MarineFish) and marine environments (Seawater, Saltmarsh, Sand-PAH) revealed that 10 of the top 17 oligotypes (Table 3) were significantly different between these habitat categories at the bonferroni-corrected alpha level of 0.0029. SIMPER revealed the strong influence of Oligotype 3 and 5 in Saltmarsh and non-host associated Seawater communities, and Oligotype 5 in Sand-PAH mesocosms. Oligotype 5 was often extremely abundant in seawater samples, including those from a *Vibrio* bloom (from project ICM_PML_Bv6, Table 1), and on plastic samples (Figure 4). ANOSIM analyses revealed that across all habitat pairwise comparisons only Sponge and MarineFish, Seawater and Saltmarsh, Sand-PAH and Plastic, and Sand-PAH and Seawater could not be significantly differentiated from one another (Table S1).

Analysis of “within-habitat similarity” (a measure of sample dispersion within a habitat) showed significant differences between habitats. *Post-hoc* Tukeys pairwise tests revealed Sand-PAH mesocosms (median distance to centroid = 0.047) and Saltmarsh (median distance to centroid = 0.046) contained significantly less variance than Seawater (median distance to centroid = 0.388) and Sponge (median distance to centroid = 0.49) habitats. We note here however, that these results do not control for the larger geographic area over which Seawater and Sponge samples were collected as compared to Sand-PAH and Saltmarsh samples. All other pairwise comparisons were insignificant at the 0.05 alpha level after multiple comparison adjustments.

### Phylogenetic and metadata analysis of abundant oligotypes

**Figure 7:**
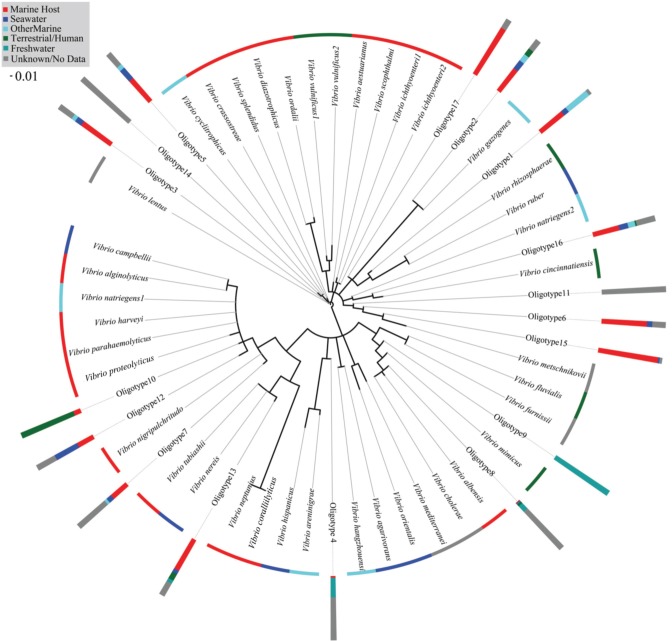
**PhyML phylogeny of full-length 16S rRNA gene sequences from type strain isolates in the SILVA ARB database, with the 17 most abundant oligotypes found in this study added by Maximum Parsimony over the 60 bp region**. The isolation source for each type strain is color coded at its terminal node. The proportion of MEGABLAST hits that fell into each isolation source category is coded for the 17 oligotype sequences added to the tree, and shown in bar graphs at each oligotype node.

Our phylogenetic analysis of ARB reference sequences revealed no 16S rRNA gene phylogeny-habitat relationship. This is evidenced by the spread of reference sequences from the same isolation source across the phylogenetic tree (Figure 7). Our 17 abundant oligotypes were also not monophyletic according to the habitat in which they were most abundant (e.g., sponges or fish). For example, oligotype sequences dominant in FreshwaterFish and Freshwater (Oligotype 2, 4, and 8) were found to branch in different parts of our phylogenetic tree (Figure 7).

MEGABLAST queries of abundant oligotypes returned on average 120 perfect matches from the NCBI nr database, although variance in this number was high (ranging from 0 to 1000). No oligotype with more than two perfect NCBI hits came from only one source, and any oligotype with more than 50 perfect NCBI hits was isolated from at least three different sources. Oligotype 5, which was the most abundant across our habitats (Figure 6), also had the maximum number of allowable perfect hits (1000). Which habitat a query sequence came from was an extremely poor predictor for the isolation source of its NCBI hits, although Oligotypes 4 and 8, which contributed to FreshwaterFish had previously been isolated from freshwater environments (Table 3), and both matched *V. cholerae*, found in brackish water, while abundant oligotypes in marine organisms (e.g., 1 and 6), did not return any previous isolations from freshwater environments. Surprisingly, Oligotype 2, which contributed most to freshwater environments, had no perfect hits from freshwater sources. The majority of isolation sources from all our NCBI hits (76%) were marine hosts, although we note the potential for database biases.

## Discussion

Our results represent the first attempt to use subtle nucleotide variation at a single, 60 bp gene marker to make sense of community level patterns across habitats within the genus *Vibrio*. Although our results are not the first to explore *Vibrio* community patterns across diverse habitats (Hunt et al., 2008; Preheim et al., 2011a; Szabo et al., 2013), we use preexisting sequence data to extend conclusions made by previous authors to a broader survey of unexplored habitats and provide novel insights into substrate-associated communities and their surrounding water.

The breadth and scope of our analyses, 211 samples representing seven unique habitats, revealed or supported several interesting patterns suggestive of two broad hypotheses regarding different aspects of *Vibrio* ecology and life history. First, *Vibrio* contains many generalist taxa, each adapted to a wide range of animal hosts. Second, our data suggest even these “host-adapted” vibrios occur as members of free-living communities facilitating long distance dispersal to disparate hosts. We suggest both of these characteristics, combined with previous understanding of rapid growth rates (McDonough et al., 2013; Skorupski and Taylor, 2013) fit the description of an “r-strategist” life history.

Several patterns within our data support these suggestions. Fish acclimated to marine salinities share highly similar *Vibrio* communities with geographically and phylogenetically distinct sponges (Figure 1, Table 4). Both marine acclimated fish and sponge communities were typified by a strong dominance of Oligotypes 1, 4, and 6, all of which were above 15% mean relative abundance in both “Sponge” and “MarineFish” samples. These oligotypes contributed at least 12% of within group Bray-Curtis similarities for each habitat (Table 4). Furthermore, ANOSIM analysis at the community level (i.e., using all oligotypes in each community) found these habitats could not be significantly separated, a conclusion supported by their overlapping distribution in 2D projections (Figures 1, 5). Although these communities do not appear to be host specific, they do appear to be specific to biotic hosts. Plastic communities, on an abiotic substrate, were typified by Oligotypes 5, 3, and 12, and while oligotypes that typified marine fish and sponges sometimes occurred on plastics, they were always in low abundance. Furthermore, pairwise ANOSIM tests showed that although Sponges and MarineFish could not be significantly separated both could be separated from Plastic. We confirmed this result with significant ANOSIM groupings of MarineFish and Sponge datasets pooled together, at the exclusion of Plastic samples (data not shown). We found groupings were again characterized by high abundance of Oligotypes 1, 4, and 6 on biotic substrates, and Oligotype 5, 3, and 12 on Plastic. This suggests that vibrios behave differently with respect to adaptation to and colonization of biotic vs. abiotic substrates. Furthermore, the finding that *Vibrio* oligotypes associated with plastics overlap with those in the surrounding seawater suggests that recruitment may take place far from the origins of the plastic marine debris itself, typically thought to be land-based.

Explaining the similarity between *Vibrio* communities in fish and sponges is challenging, but overlap in habitat geography during sampling, or overlap of laboratory sample preparation in space or time can be ruled out. Sponges were collected by SCUBA and Remote Operated Vehicle (ROV) deep-sea dives from the Northeast Atlantic and Mediterranean Sea (Reveillaud et al., 2014) from 1981 to 2011, while all fish were collected from experimental aquaria filled with sterilized water and Instant Ocean® salt mix in a Woods Hole, MA laboratory in 2013 (Schmidt et al., submitted; Supplementary Data Sheet 1). Furthermore, although sequencing was conducted on the same Illumina HiSeq instrument, run dates were several months apart, and sample storage occurred in different freezers, making contamination between projects unlikely. In addition to community level patterns outlined above, our MEGABLAST analysis of the representative sequences from oligotypes most representative of our sponge and fish samples revealed that each was previously isolated from a variety of marine hosts including crabs, jellyfish, sea squirts, corals, fish, clams, and sea cucumbers (Table 3, Table S2). Lastly, a previous comparison of two phylogenetically distinct hosts (mussels and crabs) also found significant overlap in *Vibrio* communities (Preheim et al., 2011a), although we note in this case hosts were not geographically or temporally distinct.

Such a large degree of host plasticity does not appear to be a ubiquitous feature among all bacterial taxa, and *Vibrio* clearly differs from other taxa at the community level. A previous study on sponge-microbe associations demonstrated host-specificity within the genus *Nitrospira* (Reveillaud et al., 2014). This study used the same oligotyping pipeline of V6 sequences, from the same samples analyzed here, and the authors showed strong host specificity of *Nitrospira* oligotypes to sponges at the species level (see Figure 4 in Reveillaud et al., 2014). They found closely related sponge species had differential enrichment preferences for closely related *Nitrospira* phylogenetic lineages across varying bathymetric and geographic areas. Our oligotyping analysis, focusing on *Vibrio*, highlighted the lack of sponge-specific patterns within this genus (Figure 2, **Top**), and is therefore in stark contrast to the patterns illustrated for *Nitrospira*.

Further supporting our suggestion that taxa within the genus *Vibrio* are generalist, long distance dispersing, r-strategists is the commonality of some oligotypes across the broad range of host associated and environmental habitat types in this study (Figure 7). Although *Vibrio* does not form spores (Madigan et al., 2009), it is known to enter a “viable but uncultivable” state under stressful or nutrient limiting conditions (Ramaiah et al., 2002). Research with the squid symbiont *Vibrio fischeri* found the bacteria quickly became uncultivable and non-luminescent in nutrient poor water outside of its host, but retained the ability to colonize, and luminesce, given re-entry to a suitable host (Lee and Ruby, 1995). Our results demonstrate that Oligotypes 1, 6, 4, 8, and 9 were all significantly enriched in marine hosts compared to marine environmental samples, with as much as a 168-fold enrichment, yet they all occurred at low abundance in open-ocean, host-independent samples. It is possible the rare occurrence of these “host-associated” oligotypes in seawater samples represent taxa that have entered viable but uncultivable states, giving them the ability to disperse long distances in nutrient poor waters between opportunistic colonization of a wide variety of marine hosts. Conversely, Oligotype 5 was found at an average relative abundance in environmental samples (Seawater, Saltmarsh) and an abiotic substrate (Plastic) of >40%, while averaging only 2.8% in hosts. This oligotype was found across all 179 samples analyzed as part of this study (Figure 6), and was widely represented in our MEGABLAST results (Table 3). Analysis of *Vibrio* sequences recovered from an earlier study of plastic marine debris samples (Zettler et al., 2013) also detected Oligotype 5 (data not shown) despite employing a different sequencing platform. Together, these data suggest a widely distributed, predominantly “non-host associated” *Vibrio* found in hosts only through chance or ephemeral colonization.

### Salinity drives *Vibrio* structure in water and host communities

Salinity is a known driver of *Vibrio* community structure and most vibrios are thought to occur in brackish or marine environments (Takemura et al., 2014). Schmidt et al. (submitted) (Supplementary Data Sheet 1) experimentally manipulated salt concentrations in aquaria containing a euryhaline (salt tolerant) fish, and characterized the resulting bacterial community. They found that communities in both the fish and water changed across the salt gradient, but that they did not change concurrently, resulting in drastically different communities in fish and tank water. Interestingly, fish/water differences at high salinities (18 and 30 ppt) were in part driven by high *Vibrio* relative abundance in fish, vs. its relative rarity in tank water (Figure 3). *Vibrio* also partly drove differences in fish microbiomes across the salinity gradient, with a nearly 10-fold increase in total *Vibrio* relative abundance from 0 to 30 ppt acclimated fish. This study did not, however, resolve bacteria below the genus level, and could not make conclusions about variation within a genus across salinities. The study therefore did not assess if increases in total *Vibrio* relative abundance up the gradient were due to the same taxa becoming more abundant, or to the addition of novel taxa at higher salinities. Nor were they able to assess if *Vibrio* inside fish were the same as those found in the tank water.

The fine scale resolution provided by our oligotyping analyses allowed us to answer these questions, and we show that *Vibrio* community structure between water and fish are broadly consistent, with both habitats sharing similar occurrence and relative abundances of particular *Vibrio* oligotypes (Figure 3), despite an overall enrichment of *Vibrio* relative abundance in fish vs. water. We also show that FreshWater (tank water community) and FreshwaterFish (fish microbiome community) cannot be significantly distinguished with ANOSIM tests, and SIMPER analyses find the same oligotypes (2, 4, and 8) are representative of both FreshWater and FreshwaterFish (Table 4). The same is true for comparisons between high salt acclimated fish (MarineFish) and their water (MarineWater), which are both characterized by Oligotypes 1, 6, 3, and 4. This trend for both salinities is evident from 2D projections of community structure (Figure 2, **Middle**), which show overlapping ellipsoids of fish and water habitats (although some separation is evident). Despite an overall shift in community structure across the salinity gradient, Oligotype 4 remains at high abundance in both fresh and marine samples. Oligotype 4 was highly enriched in all host-associated samples (Sponges, FreshwaterFish, MarineFish), and extremely rare in environmental water samples not associated with a host collection (i.e., completely independent of hosts). Furthermore, MEGABLAST results from this oligotype show its previous isolation from both marine and freshwater hosts, but never from seawater (Table 4). Together these results are suggestive of host-associated, potentially salt-tolerant *Vibrio* taxa.

### Advantages and limitations of oligotyping analysis for *Vibrio*

By separating regions of a marker gene that contain biologically meaningful variation from stochastic error, oligotyping allows single nucleotide differences across short marker genes to identify potentially ecologically meaningful patterns with extremely small amounts of information (Eren et al., 2013a). This technique provides substantial benefits, avoiding the need for lengthy and expensive culturing and sequencing protocols, and allows researchers to tap into massive existing databases to ask novel ecological questions at high-throughput levels across global scales. We note, however, that some serious limitations do exist for these type of data. 16S rRNA gene sequences can be nearly identical across multiple *Vibrio* species (Gomez-Gil et al., 2004), and even contain variance between copies of 16S rRNA genes within a single genome, making its use as a phylogenetic tool difficult or impossible. In addition, because our analyses use only 60 bp of DNA sequence at a single marker gene, our data are insufficient for any phylogenetic inference, and we cannot deduce relatedness between individual oligotypes. This provides major limitations in our ability to address some hypotheses about the evolutionary history of vibrios adapted to specific habitats. We could not investigate, for example, if the abundant oligotypes of fish and sponges (Oligotypes 1, 4, and 6) share common ancestry, which would suggest speciation within the genus after association with the host. We also cannot confidently tie our conclusions to previous observations about particular *Vibrio* species, such as *V. splendidus'* potentially recent adaption to particulate adhesion (Hunt et al., 2008), or *V. cholerae's* affinity for freshwater (Skorupski and Taylor, 2013), since we cannot make taxonomic assignments to any of our oligotypes. High-resolution taxonomic assignment of *Vibrio* has been a significant challenge, necessitating the use of genomic analyses including DNA-DNA hybridization, multi-locus sequence analysis (MLSA), and genome sequencing for species- or strain-level identification (Thompson et al., 2005). Analyses of multiple loci, or entire genome sequences, are therefore required to make any phylogenetic inference (Thompson et al., 2005; Preheim et al., 2011b). However, this study shows the utility of oligotyping as an easily adaptable, high-throughput and unbiased method for large-scale analyses of data from publically available sequence data repositories, and suggests its wide application could greatly extend the range of possibilities to explore microbial ecology studies of particular genera.

## Conclusions

Our analysis combines a novel bioinformatics technique with large quantities of *Vibrio* 16S rRNA gene sequence data to reveal patterns of *Vibrio* ecology across a wide range of environmental, host, and abiotic substrate-associated habitats. Despite the drawbacks for phylogenetic and taxonomic inference of using a single, short rRNA gene sequence, our analyses show strong convergence between host-associated communities, despite wide geographic and phylogenetic distance between them. We also show a surprising overlap, and a lack of significant divisions, between *Vibrio* communities in hosts and those found in their surrounding aquatic environments. Our results further support that *Vibrio*, as a genus, is largely populated by generalist r-strategist species, capable of long distance dispersal, a wide breadth of growth requirements, and rapid growth rates (Szabo et al., 2013).

### Conflict of interest statement

The authors declare that the research was conducted in the absence of any commercial or financial relationships that could be construed as a potential conflict of interest.
