# Supplementary material for: Oligotyping reveals community level habitat selection within the genus Vibrio
Source: Front Microbiol. 2014 Nov 13;5:563. doi: 10.3389/fmicb.2014.00563 (PMC4230168; doi:10.3389/fmicb.2014.00563)
Supplement: Supplementary file 5 [file DataSheet1.DOCX]

Supplementary File 2: Methods for “Community assembly of a euryhaline fish microbiome during salinity acclimation”

Submitted for publication August, 2014

*Experimental design*

We purchased *Poecilia sphenops* (the Black or Mexican Molly) individuals between 20 and 30 mm from a local supplier, where they were maintained at a salinity of 0.0 to 2.0 in mixed species aquaria. After purchase, we divided all fish into four 125-liter tanks, and acclimated each tank to either salinities of 0.1, 5.0, 18.0 or 30.0 by adding no more than 100 g (>1ppt) Instant Ocean^TM^ (Blacksburg, VA) aquarium mix per day over a 30 day period. After the acclimation period, we added two fish to each of four experimental tanks in each of our four treatments (16 tanks total with 2 fish per tank) (Figure 1). All tanks were initially filled with Nanopure^TM^ 0.2 μm-filtered deionized water and brought to target salinity with Instant Ocean salt mix with TetraWhisper^®^ air driven in-tank charcoal filters to remove solid wastes. Tanks were left uncovered and exposed to the ambient air of the flowing seawater facility at the Marine Biological Laboratory, Woods Hole, MA where experiments took place. We fed fish TetraMin^®^ (Blacksburg, VA) tropical fish flakes daily and monitored them for 12 days, changing water every four days. On three occasions additional water changes were required due to fish gamete release. Two fish died before the experiment ended and we excluded them from analyses (one at salinity 5 and the other at 18).

It is important to note that although no physical connection was shared between independent replicate tanks, the entire experiment shared a single, non-independent metacommunity. This metacommunity was the sum of the microbiomes arriving with each fish from its original source and the shared ambient environment of the seawater facility. Distinct communities between replicate tanks were not manipulated experimentally but rather developed over the course of the experiment from this shared metacommunity.

*Sample collection and DNA extraction*

After the 12-day experimental period, we euthanized fish in 10 mg/ml MS-222 according to IACUC protocols and rinsed them in sterile 1X Phosphate Buffered Saline (PBS). We then homogenized the entire fish in 30 ml of sterile 1X PBS using dissection scissors. Vortexing in a 50-ml sterile tube secured to a MoBio (Carlsbad, CA) Vortex-Genie2^®^ for 10 minutes disassociated bacterial cells from host tissue, and filtration through a 5-μm polycarbonate filter removed host tissue. We then spun the resulting filtrate at 16,000 x g for 10 minutes to pellet bacterial cells, rinsed the pellet in 1X PBS, and finally resuspended it in twice the recommended volume (600 μl) of Qiagen^®^ (Hilden, Germany) PureGene’s Yeast/Bact Kit lysis buffer. DNA extractions proceeded as per manufacturer’s protocol.

To harvest tank-water bacterial communities, we filtered one liter of water from each tank through 0.2-μm Sterivex^TM^ filters (Millipore, Billerica, MA) and extracted DNA using PureGene’s Yeast/Bac Kit as per protocols in (Smith et al., 2012). Filtrate was collected and frozen for downstream nutrient analyses at the Woods Hole Oceanographic Institution. To extract the bacterial community from food, we added 0.5 ml crumbled TetraMin^TM^ fish flakes to 0.5 ml sterile 1X PBS, ground them into a slurry with a sterilized plastic pestle and vortexed for 1 minute. This solution was spun down at 16,000 x g for five minutes and the supernatant was discarded. Pellets were suspended in PureGene’s Yeast/Bac Kit lysis buffer and extraction proceeded as per manufacturer’s protocol.

*Next-Generation Amplicon Sequencing and Bioinformatics*

We sequenced the V6 hypervariable region of the bacterial 16S rRNA gene using a custom 2-step ‘fusion primer’ Polymerase Chain Reaction (PCR) amplification. First, we perform an initial 20-cycle PCR in triplicate using a cocktail of standard forward and reverse universal bacterial primers, we then amplify this product in a 2^nd^ 10 cycle PCR using primers with Illumina HiSeq adaptors and barcodes attached to their 5’ end. Our fusion PCR protocols are further explained elsewhere (Eren et al., 2013). Paired-end sequencing was conducted at the Marine Biological Laboratory (MBL) Keck Sequencing facility on an Illumina HiSeq 1000, and generated 100-bp reads with 100% overlap of reads 1 (forward) and 2 (reverse). Quality filtering and error removal followed standard protocols at the MBL’s Bay Paul Center that removes reads wherein forward and reverse sequences do not match perfectly (Eren *et al.* 2013). We used UCLUST de novo clustering algorithm (Edgar et al., 2011) to cluster sequences at the 97% similarity level after subsampling to 900,000 reads per sample. We used ‘seed’ sequences, or those sequences that initiate a new cluster, as representatives for each cluster, and the GAST pipeline (Huber et al., 2007; Huse et al., 2008) to assigned taxonomy to each representative sequence.

**Literature Cited**

Edgar, R. C., Haas, B. J., Clemente, J. C., Quince, C., and Knight, R. (2011). UCHIME improves sensitivity and speed of chimera detection. *Bioinformatics* 27, 2194–200. doi:10.1093/bioinformatics/btr381.

Eren, M., Vineis, J. H., Morrison, H. G., and Sogin, M. L. (2013). A filtering method to generate high quality short reads using Illumina paired-end technology. *PLoS One* 8, e66643.

Huber, J., Mark Welch, D. B., Morrison, H. G., Huse, S. M., Neal, P. R., Butterfield, D., and Sogin, M. L. (2007). Microbial population structures in the deep marine biosphere. *Science (80-. ).* 318, 97–100. doi:10.1126/science.1146689.

Huse, S. M., Dethlefsen, L., Huber, J., Mark Welch, D., Welch, D. M., Relman, D., and Sogin, M. L. (2008). Exploring microbial diversity and taxonomy using SSU rRNA hypervariable tag sequencing. *PLoS Genet.* 4, e1000255. doi:10.1371/journal.pgen.1000255.

Smith, K., Schmidt, V., Rosen, G. E., and Amaral-Zettler, L. (2012). Microbial diversity and potential pathogens in ornamental fish aquarium water. *PLoS One* 7, e39971.
