# Supplementary material for: Oligotyping reveals community level habitat selection within the genus Vibrio
Source: Front Microbiol. 2014 Nov 13;5:563. doi: 10.3389/fmicb.2014.00563 (PMC4230168; doi:10.3389/fmicb.2014.00563)
Supplement: Supplementary file 6 [file DataSheet2.ZIP › HTML-OUTPUT/index.html]

noaquifer-PADDED-WITH-GAPS


Oligotyping Results for "noaquifer-PADDED-WITH-GAPS"

A user-friendly interface to make sense of oligotyping results.

Oligotyping.org

Overview

» A summary of what happened.

Oligotyping analysis was performed on
1,992,780 reads from 183 samples
for "noaquifer-PADDED-WITH-GAPS" with oligotyping pipeline version 1.0 (available from http://oligotyping.org) using
13 components
following the initial entropy analysis.
To reduce the noise, each oligotype required to
(1) appear in at least 1 sample,
(2) occur in more than 0.5% of the reads for at least one sample,
(3) represent a minimum of 0 reads in all samples combined,
and (4) have a most abundant unique sequence with a minimum abundance of 0.
Oligotypes that did not meet these criteria were removed from the analysis.
The final number of quality controlled oligotypes revealed by the analysis was 99,
and they represented 1972453 reads, which was equivalent to 98.98%
of all reads analyzed.

General Information

  

» Meta

|  |  |
| --- | --- |
| Run date | 03 Jul 14 11:53:57 |
| Library version | 1.0 |

» Given Parameters

|  |  |
| --- | --- |
| Number of entropy components chosen by the user | 13 |
| Min number of samples oligotype expected to appear | 1 |
| Min % abundance of oligotype in at least one sample | 0.5% |
| Min total abundance of oligotype in all samples | 0 |
| Min substantive abundance of an oligotype (-M) | 0 |
| Quality scores were provided | False |
| Oligotype sets were requested to be generated | True |
| Cosine similarty threshold to generate oligotype sets | 0.1 |

» Components used for Oligotyping

|  |  |
| --- | --- |
| Base locations of interest in the alignment | 13, 15, 20, 21, 22, 23, 25, 31, 32, 37, 45, 50, 55 |

» Quality filtering results

|  |  |
| --- | --- |
| Number of sequences analyzed | 1,992,780 |
|  | |
| Number of unique oligotypes (raw) | 1,452 |
| Oligotypes after "min number of samples" elimination | 1,452 |
| Oligotypes after "min % abundance in a sample" elimination | 99 |
|  | |
| Number of sequences analyzed | 1,992,780 |
| Number of samples found | 183 |
| Number of sequences represented after quality filtering | 1,972,453 |
| Percentage of reads represented in results | 98.98% |

Downloads

» Files to analyze results further via third partry applications

|  |  |
| --- | --- |
| Representative sequences per oligotype | oligo-representatives.fa.txt |
| Sample/oligotype abundance data matrix (percents) | matrix\_percents.txt |
| Sample/oligotype abundance data matrix (counts) | matrix\_counts.txt |
| Read distribution among samples table | read\_distribution.txt |
| Mapping file | sample\_mapping.txt |
| GEXF file for network analysis | network.gexf |
| Environment file | environment.txt |
| FASTA file for abundant oligotypes | oligos.fa.txt |
| NEXUS file for abundant oligotypes | oligos.nex.txt |
| Oligotypes across samples matrix (MAX normalized) | across\_samples\_max\_normalized.txt |
| Oligotypes across samples matrix (SUM normalized) | across\_samples\_sum\_normalized.txt |
| Groups of oligotypes | oligotype\_sets.txt |
| Abundance data matrix for oligotype sets (percents) | matrix\_percents\_oligo\_sets.txt |
| Abundance data matrix for oligotype sets (counts) | matrix\_counts\_oligo\_sets.txt |

Entropy

» Entropy values that components were picked from

Stackbar

» Figure shows the oligotype distribution profiles among samples. TAB separated files matrix\_percents.txt and matrix\_counts.txt hold the information that were used to generate this figure.

Read Distribution per Sample

» Total number of reads for each sample that were analyzed.

Oligotypes

» Abundant oligotypes along with their frequencies within the project are shown below. Every oligotype is followed by a representative sequence, which is the most frequent read in all reads that were collected by the given oligotype. Mouseover on an oligotype will popup a figure that shows the abundance distribution of unique sequences within the oligotype, along with the new entropy (in an ideal world there should be only one unique read with 0 entropy, but due to the random sequencing errors it is almost never the case).

**CGGCCATTATCAG** b ||||||||||||||||||||||||||||||||||||||||||||||||||||||||||||||||||||||||||

 

321,857
  
 **CGGCCATTATCAG** *b* TACTCTTGACATCCAGAGAAGCCAGTAGAGATACAGGTGTGCCTTCGGGAACTCTGAGAC--------------

 

267,250
  
 **CCGAGATCATCAG** *b* TACTCTTGACATCCTCAGAAGAGACTGGAGACAGTCTTGTGCCTTCGGGAACTGAGAGAC--------------

 

251,405
  
 **CGGCCACCGTCAG** *b* TACTCTTGACATCCAGAGAAGCCAGCGGAGACGCAGGTGTGCCTTCGGGAACTCTGAGAC--------------

 

230,945
  
 **CGTCTACCGACAG** *b* TACTCTTGACATCCAGAGAATCTAGCGGAGACGCTGGAGTGCCTTCGGGAACTCTGAGAC--------------

 

229,914
  
 **CGGCCACCGTCGG** *b* TACTCTTGACATCCAGAGAAGCCAGCGGAGACGCAGGTGTGCCTTCGGGAGCTCTGAGAC--------------

 

210,275
  
 **TGGCCACCGTCAA** *b* TACTCTTGACATCTAGAGAAGCCAGCGGAGACGCAGGTGTGCCTTCGGGAACTCTAAGAC--------------

 

116,003
  
 **CTCTTACTGGCAG** *b* TACTCTTGACATCCATAGAACTTAGCAGAGATGCTTTGGTGCCTTCGGGAACTATGAGAC--------------

 

90,807
  
 **CGTCTACCGACGG** *b* TACTCTTGACATCCAGAGAATCTAGCGGAGACGCTGGAGTGCCTTCGGGAGCTCTGAGAC--------------

 

46,765
  
 **CGCCTACCGACAG** *b* TACTCTTGACATCCAGAGAACCTAGCGGAGACGCTGGAGTGCCTTCGGGAACTCTGAGAC--------------

 

19,613
  
 **CGCTTTCTGGCAG** *b* TACTCTTGACATCCAGAGAGCTTTCCAGAGATGGATTGGTGCCTTCGGGAACTCTGAGAC--------------

 

19,156
  
 **CGGCCATCATCAG** *b* TACTCTTGACATCCAGAGAAGCCAGTGGAGACACAGGTGTGCCTTCGGGAACTCTGAGAC--------------

 

17,575
  
 **CGTTTTCTGGCAG** *b* TACTCTTGACATCCAGAGAATTTTCCAGAGATGGATTGGTGCCTTCGGGAACTCTGAGAC--------------

 

16,595
  
 **CGTCCACCGACGG** *b* TACTCTTGACATCCAGAGAATCCAGCGGAGACGCAGGAGTGCCTTCGGGAGCTCTGAGAC--------------

 

16,356
  
 **TGGCCACCGTCAG** *b* TACTCTTGACATCTAGAGAAGCCAGCGGAGACGCAGGTGTGCCTTCGGGAACTCTGAGAC--------------

 

14,176
  
 **CTCTTTCTGGCAG** *b* TACTCTTGACATCCATAGAACTTTCCAGAGATGGATTGGTGCCTTCGGGAACTATGAGAC--------------

 

13,588
  
 **CGGCCATCATCGG** *b* TACTCTTGACATCCAGAGAAGCCAGTGGAGACACAGGTGTGCCTTCGGGAGCTCTGAGAC--------------

 

12,737
  
 **CCCTTACTGGCAG** *b* TACTCTTGACATCCTCAGAACTTAGCAGAGATGCTTTGGTGCCTTCGGGAACTGAGTGAC--------------

 

11,102
  
 **TCGCCACCGTCAA** *b* TACTCTTGACATCTACAGAAGCCAGCGGAGACGCAGGTGTGCCTTCGGGAACTGTAAGAC--------------

 

9,914
  
 **CGGCCATTATCGG** *b* TACTCTTGACATCCAGAGAAGCCAGTAGAGATACAGGTGTGCCTTCGGGAGCTCTGAGAC--------------

 

7,914
  
 **CCGCTTTTATCAG** *b* TACTCTTGACATCCTCAGAAGCTTGTAGAGATACGAGTGTGCCTTCGGGAACTGAGAGAC--------------

 

6,358
  
 **CGGCCATTGTCAG** *b* TACTCTTGACATCCAGAGAAGCCAGTAGAGATGCAGGTGTGCCTTCGGGAACTCTGAGAC--------------

 

3,464
  
 **TCGCTTCCGTCAA** *b* TACTCTTGACATCTACAGAAGCTTGCGGAGACGCGAGTGTGCCTTCGGGAACTGTAAGAC--------------

 

3,002
  
 **CGGTCATCATCGG** *b* TACTCTTGACATCCAGAGAAGTCAGTGGAGACACAGGTGTGCCTTCGGGAGCTCTGAGAC--------------

 

2,580
  
 **CGGCCGATTTCGG** *b* TACTCTTGACATCCAGAGAAGCCGGAAGAGATTCTGGTGTGCCTTCGGGAGCTCTGAGAC--------------

 

2,244
  
 **CGTCCACCGACAG** *b* TACTCTTGACATCCAGAGAATCCAGCGGAGACGCAGGAGTGCCTTCGGGAACTCTGAGAC--------------

 

2,127
  
 **CGACCACCGTCGG** *b* TACTCTTGACATCCAGAGAAACCAGCGGAGACGCAGGTGTGCCTTCGGGAGCTCTGAGAC--------------

 

1,693
  
 **CGGCCACTGTCGG** *b* TACTCTTGACATCCAGAGAAGCCAGCAGAGATGCAGGTGTGCCTTCGGGAGCTCTGAGAC--------------

 

1,499
  
 **TGGCCACCGTCGA** *b* TACTCTTGACATCTAGAGAAGCCAGCGGAGACGCAGGTGTGCCTTCGGGAGCTCTAAGAC--------------

 

1,414
  
 **CGGCCACCGCCGG** *b* TACTCTTGACATCCAGAGAAGCCAGCGGAGACGCAGGCGTGCCTTCGGGAGCTCTGAGAC--------------

 

1,203
  
 **TGGCCATCATCAA** *b* TACTCTTGACATCTAGAGAAGCCAGTGGAGACACAGGTGTGCCTTCGGGAACTCTAAGAC--------------

 

1,197
  
 **CGGCCATCGTCAG** *b* TACTCTTGACATCCAGAGAAGCCAGTGGAGACGCAGGTGTGCCTTCGGGAACTCTGAGAC--------------

 

1,141
  
 **CGCTTTCCGGCAG** *b* TACTCTTGACATCCAGAGAACTTTCCAGAGACGGATTGGTGCCTTCGGGAACTCTGAGAC--------------

 

1,051
  
 **CGGCCATCACCGG** *b* TACTCTTGACATCCAGAGAAGCCAGTGGAGACACAGGCGTGCCTTCGGGAGCTCTGAGAC--------------

 

973
  
 **CGGTCGCCGTCAG** *b* TACTCTTGACATCCAGAGAAGTCGACGGAGACGTTGATGTGCCTTCGGGAACTCTGAGAC--------------

 

954
  
 **CGTTTGTTAACGG** *b* TACTCTTGACATCCAGAGAATTTGCTAGAGATAGCTTAGTGCCTTCGGGAGCTCTGAGAC--------------

 

896
  
 **CGGCCGATTTCAG** *b* TACTCTTGACATCCAGAGAAGCCGGAAGAGATTCTGGTGTGCCTTCGGGAACTCTGAGAC--------------

 

885
  
 **CCGGGATCATCAG** *b* TACTCTTGACATCCTCAGAAGGGACTGGAGACAGTCTTGTGCCTTCGGGAACTGAGAGAC--------------

 

848
  
 **CGTCCTCCGACAG** *b* TACTCTTGACATCCAGAGAATCCTGCGGAGACGCGGGAGTGCCTTCGGGAACTCTGAGAC--------------

 

833
  
 **CTCTTTCTGGCGG** *b* TACTCTTGACATCCATAGAACTTTCCAGAGATGGATTGGTGCCTTCGGGAGCTATGAGAC--------------

 

833
  
 **CGGCCACCGCCAG** *b* TACTCTTGACATCCAGAGAAGCCAGCGGAGACGCAGGCGTGCCTTCGGGAACTCTGAGAC--------------

 

825
  
 **CCGAGGTCATCAG** *b* TACTCTTGACATCCTCAGAAGAGGCTGGAGACAGTCTTGTGCCTTCGGGAACTGAGAGAC--------------

 

683
  
 **CGGCCATCGTCGG** *b* TACTCTTGACATCCAGAGAAGCCAGTGGAGACGCAGGTGTGCCTTCGGGAGCTCTGAGAC--------------

 

647
  
 **CGGCCCTCGTCGG** *b* TACTCTTGACATCCAGAGAAGCCCGTGGAGACGCAGGTGTGCCTTCGGGAGCTCTGAGAC--------------

 

641
  
 **CCGAGATCACCAG** *b* TACTCTTGACATCCTCAGAAGAGACTGGAGACAGTCTCGTGCCTTCGGGAACTGAGAGAC--------------

 

638
  
 **CCGAGATCGTCAG** *b* TACTCTTGACATCCTCAGAAGAGACTGGAGACGGTCTTGTGCCTTCGGGAACTGAGAGAC--------------

 

635
  
 **CGCTTTCTGACAG** *b* TACTCTTGACATCCAGAGAACTTTCCAGAGATGGATTAGTGCCTTCGGGAACTCTGAGAC--------------

 

608
  
 **CGGCCACCGTAGG** *b* TACTCTTGACATCCAGAGAAGCCAGCGGAGACGCAGGTGTGCCTTAGGGAGCTCTGAGAC--------------

 

593
  
 **CGTCCACCGAAGG** *b* TACTCTTGACATCCAGAGAATCCAGCGGAGACGCAGGAGTGCCTTAGGGAGCTCTGAGAC--------------

 

586
  
 **CGTCCTCCGACGG** *b* TACTCTTGACATCCAGAGAATCCTGCGGAGACGCGGGAGTGCCTTCGGGAGCTCTGAGAC--------------

 

581
  
 **CCGAGATCATCGG** *b* TACTCTTGACATCCTCAGAAGAGACTGGAGACAGTCTTGTGCCTTCGGGAGCTGAGAGAC--------------

 

539
  
 **CGTCCACCGTCGG** *b* TACTCTTGACATCCAGAGAATCCAGCGGAGACGCAGGTGTGCCTTCGGGAGCTCTGAGAC--------------

 

522
  
 **CGCTTACTGGCAG** *b* TACTCTTGACATCCAGAGAACTTAGCAGAGATGCTTTGGTGCCTTCGGGAACTCTGATAC--------------

 

495
  
 **CGGCCATTATCAA** *b* TACTCTTGACATCCAGAGAAGCCAGTAGAGATACAGGTGTGCCTTCGGGAACTCTAAGAC--------------

 

465
  
 **CGTCTGCCGACGG** *b* TACTCTTGACATCCAGAGAATCTGGCGGAGACGCTGGAGTGCCTTCGGGAGCTCTGAGAC--------------

 

444
  
 **CCACATCTGGCAG** *b* AACCCTTGACATCCTCAGCTACATCCAGAGATGGATTGGTGCCTTCGGGAACTCTGACAC--------------

 

320
  
 **TCTCCTCCGACAA** *b* TACTCTTGACATCTACAGGATCCTGCGGAGACGCGGGAGTGCCTTCGGGAACTGTAAGAC--------------

 

284
  
 **CGGCCACCGTCGT** *b* TACTCTTGACATCCAGAGAAGCCAGCGGAGACGCAGGTGTGCCTTCGGGAGCTCTTAGAC--------------

 

272
  
 **CTGCCACCGTCGG** *b* TACTCTTGACATCCATAGAAGCCAGCGGAGACGCAGGTGTGCCTTCGGGAGCTCTGAGAC--------------

 

266
  
 **TGGCCATCATCGA** *b* TACTCTTGACATCTAGAGAAGCCAGTGGAGACACAGGTGTGCCTTCGGGAGCTCTAAGAC--------------

 

258
  
 **CGGCGACTGTCGG** *b* TACTCTTGACATCCAGAGAAGCGACCAGAGATGGACGTGTGCCTTCGGGAGCTCTGAGAC--------------

 

244
  
 **CGCTTTCTGGCAA** *b* TACTCTTGACATCCAGAGAACTTTCCAGAGATGGATTGGTGCCTTCGGGAACTGTAAGAC--------------

 

223
  
 **CGTCTGCCGACAG** *b* TACTCTTGACATCCAGAGAATCTGGCGGAGACGCTGGAGTGCCTTCGGGAACTCTGAGAC--------------

 

223
  
 **CGGCCACCGTCTG** *b* TACTCTTGACATCCAGAGAAGCCAGCGGAGACGCAGGTGTGCCTTCGGGATCTCTGAGAC--------------

 

195
  
 **CGGCCACCGTCAT** *b* TACTCTTGACATCCAGAGAAGCCAGCGGAGACGCAGGTGTGCCTTCGGGGAACTCTGAGAC-------------

 

186
  
 **CGGCTACCGTCGG** *b* TACTCTTGACATCCAGAGAAGCTAGCGGAGACGCAGGTGTGCCTTCGGGAGCTCTGAGAC--------------

 

163
  
 **CGGCCACCGTCCA** *b* TACTCTTGACATCCAGAGAAGCCAGCGGAGACGCAGGTGTGCCTTCGGAGCTCTGAGAC---------------

 

163
  
 **AGGCCACCGTCGG** *b* TACTCTTGACATCAAGAGAAGCCAGCGGAGACGCAGGTGTGCCTTCGGGAGCTCTGAGAC--------------

 

152
  
 **CTCTTACTGGCGG** *b* TACTCTTGACATCCATAGAACTTAGCAGAGATGCTTTGGTGCCTTCGGGAGCTATGAGAC--------------

 

129
  
 **TCGAGAATTTCAA** *b* TACTCTTGACATCTACAGAAGAGAAAAGAGATTTTCTTGTGCCTTCGGGAACTGTAAGAC--------------

 

120
  
 **CGACATCTGGCAG** *b* AACCCTTGACATCCTGTGCTACATCCAGAGATGGATTGGTGCCTTCGGGAACTCTGAGAC--------------

 

117
  
 **TGTCCACCGACAA** *b* TACTCTTGACATCTAGAGAATCCAGCGGAGACGCAGGAGTGCCTTCGGGAACTCTAAGAC--------------

 

107
  
 **CACTTACTGGCAG** *b* TACTCTTGACATCCAAAGAACTTAGCAGAGATGCTTTGGTGCCTTCGGGAACTCTGAGAC--------------

 

103
  
 **CGGTTTCTGCCAG** *b* TACTCTTGACATCCAGAGAAGTTTCCAGAGATGGATTCGTGCCTTCGGGAACTCTGAGAC--------------

 

95
  
 **CCGGTTTTATCAG** *b* TACCCTTGACTTCCTCAGAAGGTTGTAGAGATACAGCTGTGCCTTCGGGAACTGAGAGAC--------------

 

82
  
 **CCGCTTCTGTCAG** *b* TACCCTTGACATACTCAGAAGCTTGCAGAAATGCGAGTGTGCCTTCGGGAACTGAGATAC--------------

 

74
  
 **CGTCCACCGTAAG** *b* TACTCTTGACATCCAGAGAATCCAGCGGAGACGCAGGTGTGCCTTAGGGAACTCTGAGAC--------------

 

65
  
 **TGGCCATCATAGA** *b* TACTCTTGACATCTAGAGAAGCCAGTGGAGACACAGGTGTGCCTTAGGGAGCTCTAAGAC--------------

 

57
  
 **TCTCCACCGACAA** *b* TACTCTTGACATCTACAGAATCCAGCGGAGACGCAGGAGTGCCTTCGGGAACTGTAAGAC--------------

 

56
  
 **CGCTTTCTGGGCA** *b* TACTCTTGACATCCAGAGAACTTTCCAGAGATGGATTGGTGCCTCGGGAACTCTGAGAC---------------

 

52
  
 **CGCTTCCTGGCAG** *b* TACTCTTGACATCCAGAGAACTTCTCAGAGATGAATTGGTGCCTTCGGGAACTCTGAGAC--------------

 

51
  
 **CTGCCATCATTAG** *b* TACTCTTGACATCCTTCGAAGCCACTGGAGACAGTGGTGTGCCTTTAGGAACGAAGAGAC--------------

 

47
  
 **CGCTTTCTGGCGG** *b* TACTCCTGACATCCAGAGAACTTTCCAGAGATGGATTGGTGCCTTCGGGAGCTCTGAGAC--------------

 

44
  
 **TCGCCACCGCCAA** *b* TACTCTTGACATCTACAGAAGCCAGCGGAGACGCAGGCGTGCCTTCGGGAACTGTAAGAC--------------

 

37
  
 **CGCTTTCTGGCTG** *b* TACTCTTGACATCCAGAGAACTTTCCAGAGATGGATTGGTGCCTTCGGGATCTCTGAGAC--------------

 

27
  
 **CCTTTATTAACAG** *b* TACTCTTGACATCCTCAGAATTTAGTAGAGATACTTTAGTGCCTTCGGGAACTGAGAGAC--------------

 

26
  
 **CGGCTTCCGTCGG** *b* TACTCTTGACATCCAGAGAAGCTTGCGGAGACGCAGGTGTGCCTTCGGGAGCTCTGAGAC--------------

 

26
  
 **CGGCCATTATAGG** *b* TACTCTTGACATCCAGAGAAGCCAGTAGAGATACAGGTGTGCCTTAGGGAGCTCTGAGAC--------------

 

24
  
 **CGGCCGATTTAGG** *b* TACTCTTGACATCCAGAGAAGCCGGAAGAGATTCTGGTGTGCCTTAGGGAGCTCTGAGAC--------------

 

21
  
 **GCGCTTTGATCAC** *b* TACCCTTGACATAGTCAGAAGCTTGTCGAGAGACGAGTGTGCCTTCGGGAATTGGCATAC--------------

 

19
  
 **CGGCCATTATAAG** *b* TACTCTTGACATCCAGAGAAGCCAGTAGAGATACAGGTGTGCCTTAGGGAACTCTGAGAC--------------

 

17
  
 **CGGCCATTACAAG** *b* TACTCTTGACATCCAGAGAAGCCAGTAGAGATACAGGCGTGCCTTAGGGAACTCTGAGAC--------------

 

16
  
 **CGGCCATTACAGG** *b* TACTCTTGACATCCAGAGAAGCCAGTAGAGATACAGGCGTGCCTTAGGGAGCTCTGAGAC--------------

 

14
  
 **CGTCCATCAACGG** *b* TACTCTTGACATCCAGAGAATCCAGTGGAGACATTGGAGTGCCTTCGGGAGCTCTGAGAC--------------

 

13
  
 **CGTCCTCAGACGG** *b* TACTCTTGACATCCAGAGAATCCTGCGGAGAAGCGGGAGTGCCTTCGGGAGCTCTGAGAC--------------

 

8
  
 **CGGCCAATTTCGG** *b* TACTCTTGACATCCAGAGAAGCCAGAAGAGATTCTGGTGTGCCTTCGGGAGCTCTGAGAC--------------

 

7
  
 **CTCTTTCTGGGCA** *b* TACTCTTGACATCCATAGAACTTTCCAGAGATGGATTGGTGCCTCGGGAACTATGAGAC---------------

 

4
  
 **CTTCCGCGGACAG** *b* TACTCTTGACATCCATAGAATCCGGCTGAGAGGCTGGAGTGCCTTCGGGAATTATGAGAC--------------

 

1
  
 **CGCTTTCTGGACA** *b* CACTCTTGACATCCAGAGAACTTTCCAGAGATGAATTGGTGCTTCAGGAACTCTGAGAC---------------

 

1
  
 **CGCCTTAGGGGCA** *b* TACTCTTGACATCCAGAGAACCTTCAGAGATGGATTGGTGCCTTCGGGAACTCTGAGAC---------------

Oligotype Frequency Patterns Across Samples

» Figures show the presence of oligotypes across samples normalized by sum of the percent abundance of an oligotype in all samples and normalized by max percent abundance of an oligotype in all samples.

Oligotype Sets

» Figure below shows the sets of oligotypes that are agglomerated in distinct sets based on their frequency patterns across samples. In this case cosine similarity threshold to consider two oligotypes to be in the same set was 0.1, and 99 oligotypes agglomerated into 98 sets. It can be argued that if two oligotypes co-occur in all samples with great similarity in frequency patterns, they can be considered functionally somewhat anologous.

The oligotype content of each set is shown below. This table also serves as a legend for the figure above, as well as the stackbar figure below. This information is also available in oligotype\_sets.txt file.

|  |  |
| --- | --- |
|

**Set 0** | CGGCCATTATCAG |


|

**Set 1** | CCGAGATCATCAG |


|

**Set 2** | CGGCCACCGTCAG |


|

**Set 3** | CGTCTACCGACAG |


|

**Set 4** | CGGCCACCGTCGG |


|

**Set 5** | TGGCCACCGTCAA |


|

**Set 6** | CTCTTACTGGCAG |


|

**Set 7** | CGTCTACCGACGG |


|

**Set 8** | CGCCTACCGACAG |


|

**Set 9** | CGCTTTCTGGCAG |


|

**Set 10** | CGGCCATCATCAG |


|

**Set 11** | CGTTTTCTGGCAG |


|

**Set 12** | CGTCCACCGACGG |


|

**Set 13** | TGGCCACCGTCAG |


|

**Set 14** | CTCTTTCTGGCAG |


|

**Set 15** | CGGCCATCATCGG |


|

**Set 16** | CCCTTACTGGCAG |


|

**Set 17** | TCGCCACCGTCAA |


|

**Set 18** | CGGCCATTATCGG |


|

**Set 19** | CCGCTTTTATCAG |


|

**Set 20** | CGGCCATTGTCAG |


|

**Set 21** | TCGCTTCCGTCAA |


|

**Set 22** | CGGTCATCATCGG |


|

**Set 23** | CGGCCGATTTCGG |


|

**Set 24** | CGTCCACCGACAG |


|

**Set 25** | CGACCACCGTCGG |


|

**Set 26** | CGGCCACTGTCGG |


|

**Set 27** | TGGCCACCGTCGA |


|

**Set 28** | CGGCCACCGCCGG |


|

**Set 29** | TGGCCATCATCAA |


|

**Set 30** | CGGCCATCGTCAG |


|

**Set 31** | CGCTTTCCGGCAG |


|

**Set 32** | CGGCCATCACCGG |


|

**Set 33** | CGGTCGCCGTCAG |


|

**Set 34** | CGTTTGTTAACGG |


|

**Set 35** | CGGCCGATTTCAG |


|

**Set 36** | CCGGGATCATCAG |


|

**Set 37** | CGTCCTCCGACAG |


|

**Set 38** | CTCTTTCTGGCGG |


|

**Set 39** | CGGCCACCGCCAG |


|

**Set 40** | CCGAGGTCATCAG |


|

**Set 41** | CGGCCATCGTCGG |


|

**Set 42** | CGGCCCTCGTCGG |


|

**Set 43** | CCGAGATCACCAG |


|

**Set 44** | CCGAGATCGTCAG |


|

**Set 45** | CGCTTTCTGACAG |


|

**Set 46** | CGGCCACCGTAGG |


|

**Set 47** | CGTCCACCGAAGG |


|

**Set 48** | CGTCCTCCGACGG |


|

**Set 49** | CCGAGATCATCGG |


|

**Set 50** | CGTCCACCGTCGG |


|

**Set 51** | CGCTTACTGGCAG |


|

**Set 52** | CGGCCATTATCAA |


|

**Set 53** | CGTCTGCCGACGG |


|

**Set 54** | CCACATCTGGCAG |


|

**Set 55** | TCTCCTCCGACAA |


|

**Set 56** | CGGCCACCGTCGT |


|

**Set 57** | CTGCCACCGTCGG |


|

**Set 58** | TGGCCATCATCGA |


|

**Set 59** | CGGCGACTGTCGG |


|

**Set 60** | CGCTTTCTGGCAA |


|

**Set 61** | CGTCTGCCGACAG |


|

**Set 62** | CGGCCACCGTCTG |


|

**Set 63** | CGGCCACCGTCAT |


|

**Set 64** | CGGCTACCGTCGG |


|

**Set 65** | CGGCCACCGTCCA |


|

**Set 66** | AGGCCACCGTCGG |


|

**Set 67** | CTCTTACTGGCGG |


|

**Set 68** | TCGAGAATTTCAA |


|

**Set 69** | CGACATCTGGCAG |


|

**Set 70** | TGTCCACCGACAA |


|

**Set 71** | CACTTACTGGCAG |


|

**Set 72** | CGGTTTCTGCCAG |


|

**Set 73** | CCGGTTTTATCAG |


|

**Set 74** | CCGCTTCTGTCAG |


|

**Set 75** | CGTCCACCGTAAG |


|

**Set 76** | TGGCCATCATAGA |


|

**Set 77** | TCTCCACCGACAA |


|

**Set 78** | CGCTTTCTGGGCA |


|

**Set 79** | CGCTTCCTGGCAG |


|

**Set 80** | CTGCCATCATTAG    GCGCTTTGATCAC |


|

**Set 81** | CGCTTTCTGGCGG |


|

**Set 82** | TCGCCACCGCCAA |


|

**Set 83** | CGCTTTCTGGCTG |


|

**Set 84** | CCTTTATTAACAG |


|

**Set 85** | CGGCTTCCGTCGG |


|

**Set 86** | CGGCCATTATAGG |


|

**Set 87** | CGGCCGATTTAGG |


|

**Set 88** | CGGCCATTATAAG |


|

**Set 89** | CGGCCATTACAAG |


|

**Set 90** | CGGCCATTACAGG |


|

**Set 91** | CGTCCATCAACGG |


|

**Set 92** | CGTCCTCAGACGG |


|

**Set 93** | CGGCCAATTTCGG |


|

**Set 94** | CTCTTTCTGGGCA |


|

**Set 95** | CTTCCGCGGACAG |


|

**Set 96** | CGCTTTCTGGACA |


|

**Set 97** | CGCCTTAGGGGCA |

Following figure shows the distribution of oligotype sets identified by the cosine similarity analysis. The data that was used to generate this figure is also available via matrix\_percents\_oligo\_sets.txt and matrix\_counts\_oligo\_sets.txt files.

Samples

» Overview of samples.

Basic Analyses

» Deafult

» cluster\_analysis

|  |  |  |  |  |
| --- | --- | --- | --- | --- |
| jaccard | bray | kulczynski | canberra | horn |

» nmds\_analysis

|  |  |  |  |  |
| --- | --- | --- | --- | --- |
| jaccard | bray | kulczynski | canberra | horn |

Exclusive Analyses

» environment

» nmds\_analysis

|  |  |  |  |  |
| --- | --- | --- | --- | --- |
| jaccard | bray | kulczynski | canberra | horn |

» heatmap\_analysis

|  |  |  |  |  |
| --- | --- | --- | --- | --- |
| jaccard | bray | kulczynski | canberra | horn |

General | Downloads | Entropy | Stackbar | Counts | Oligotypes | Samples | **Basic Analyses** | **Exclusive Analyses**

For questions and comments: meren / mbl.edu
