# Supplementary figures and images for: Oligotyping reveals community level habitat selection within the genus Vibrio (part 2 of 2)
Source: Front Microbiol. 2014 Nov 13;5:563. doi: 10.3389/fmicb.2014.00563 (PMC4230168; doi:10.3389/fmicb.2014.00563)

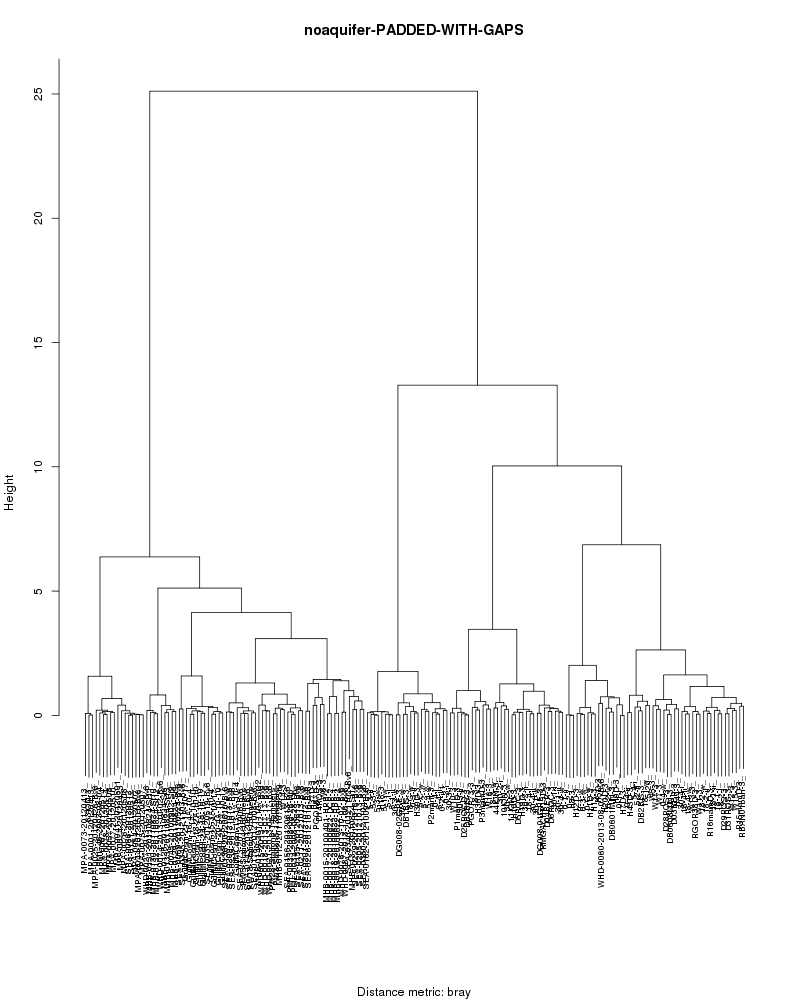

Supplement: Supplementary file 6 [file DataSheet2.ZIP › HTML-OUTPUT/basic_analyses-cluster_analysis-bray.png]

# noaquifer-PADDED-WITH-GAPS

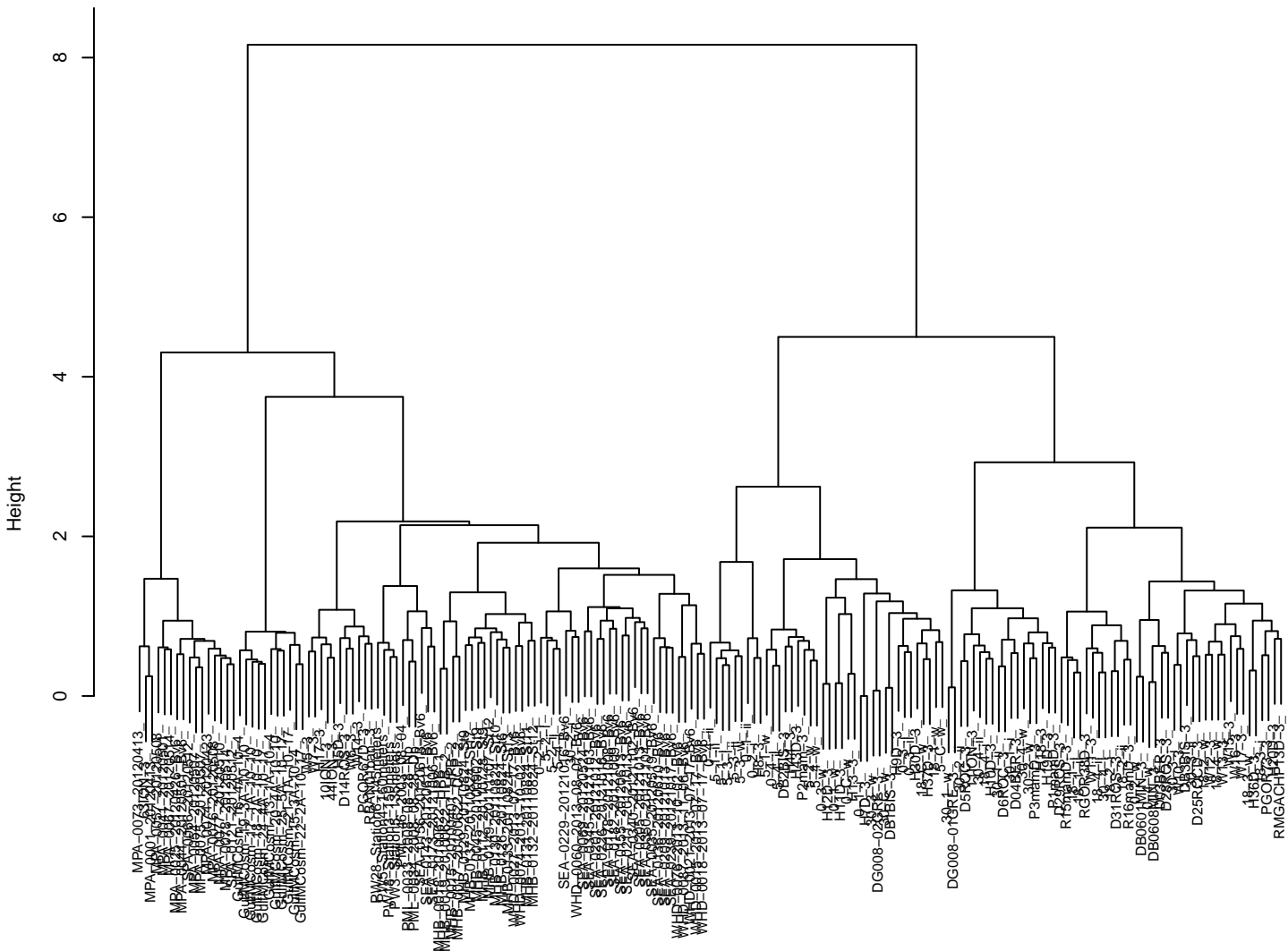

Supplement: Supplementary file 6 [file DataSheet2.ZIP › HTML-OUTPUT/basic_analyses-cluster_analysis-canberra.pdf]

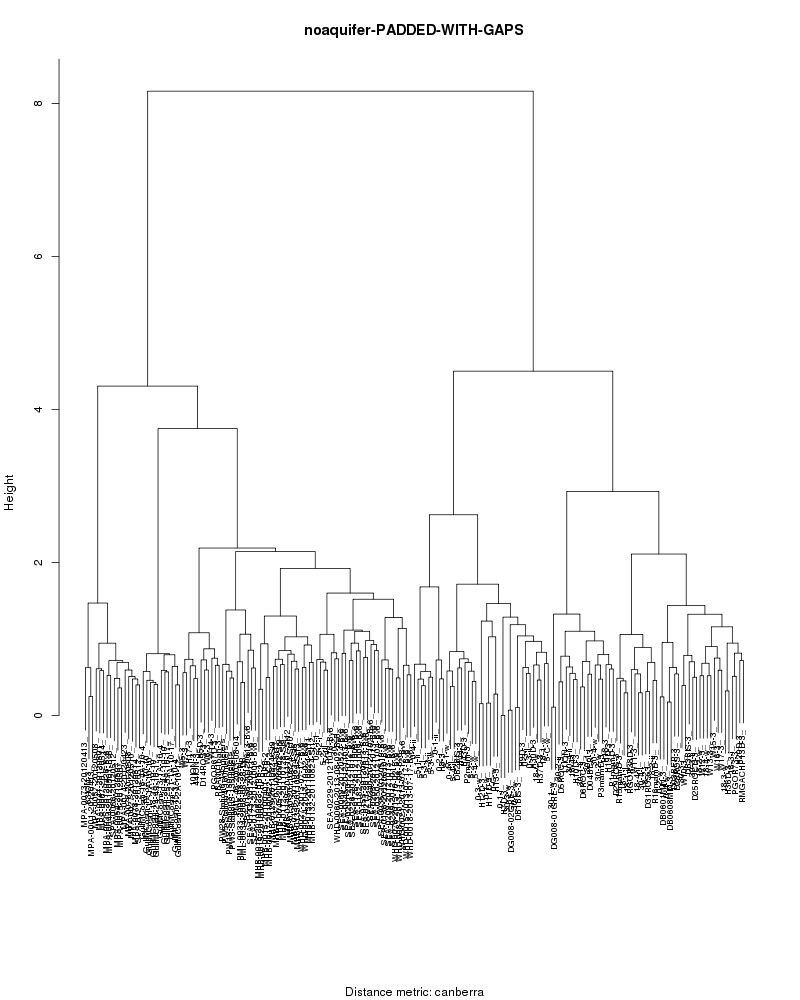

Supplement: Supplementary file 6 [file DataSheet2.ZIP › HTML-OUTPUT/basic_analyses-cluster_analysis-canberra.png]

## noaquifer-PADDED-WITH-GAPS

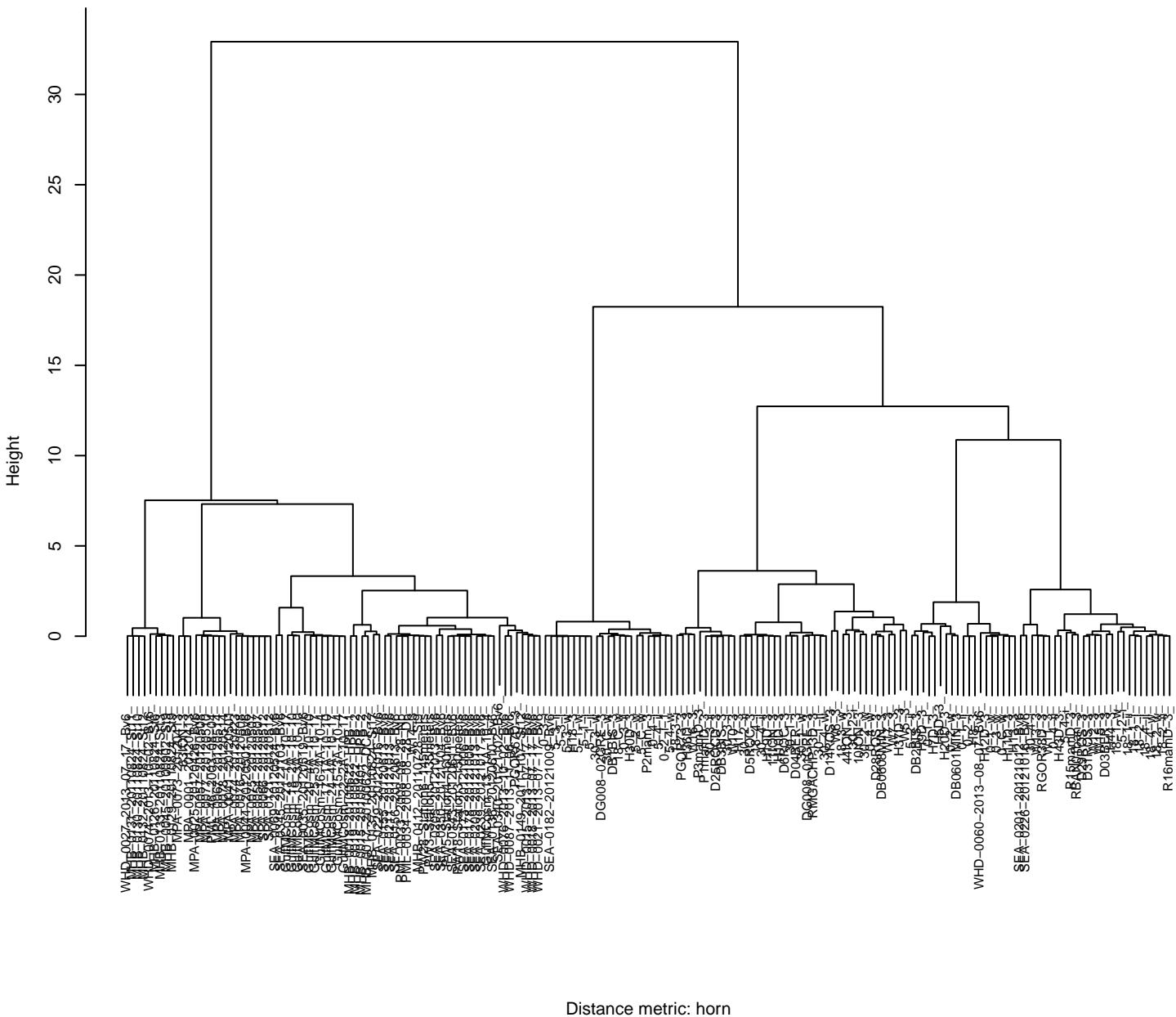

Supplement: Supplementary file 6 [file DataSheet2.ZIP › HTML-OUTPUT/basic_analyses-cluster_analysis-horn.pdf]

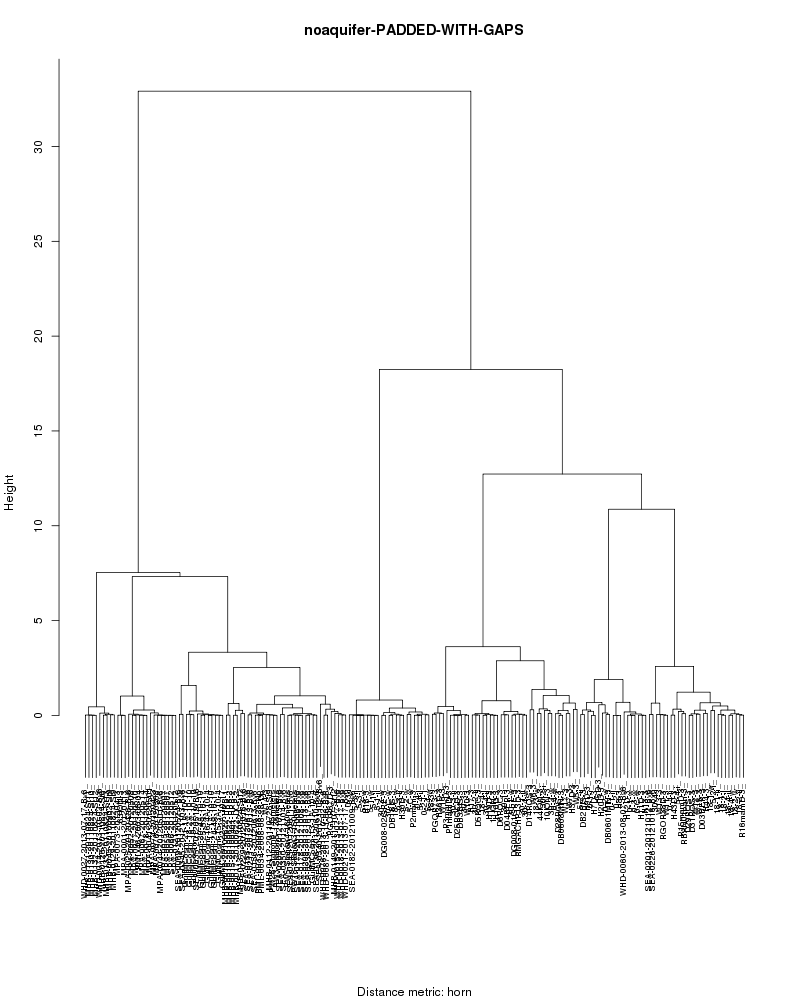

Supplement: Supplementary file 6 [file DataSheet2.ZIP › HTML-OUTPUT/basic_analyses-cluster_analysis-horn.png]

## noaquifer-PADDED-WITH-GAPS

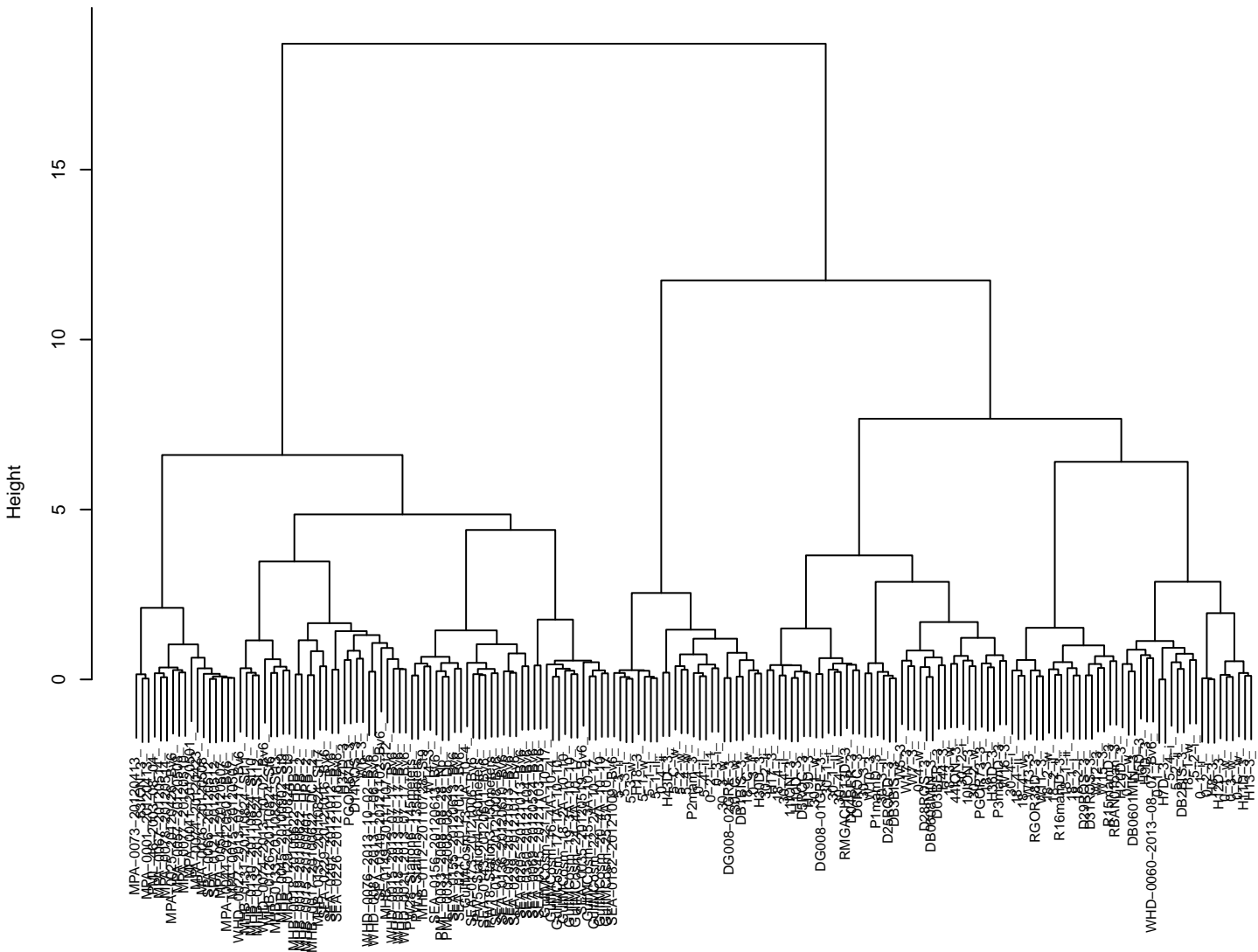

Distance metric: jaccard

Supplement: Supplementary file 6 [file DataSheet2.ZIP › HTML-OUTPUT/basic_analyses-cluster_analysis-jaccard.pdf]

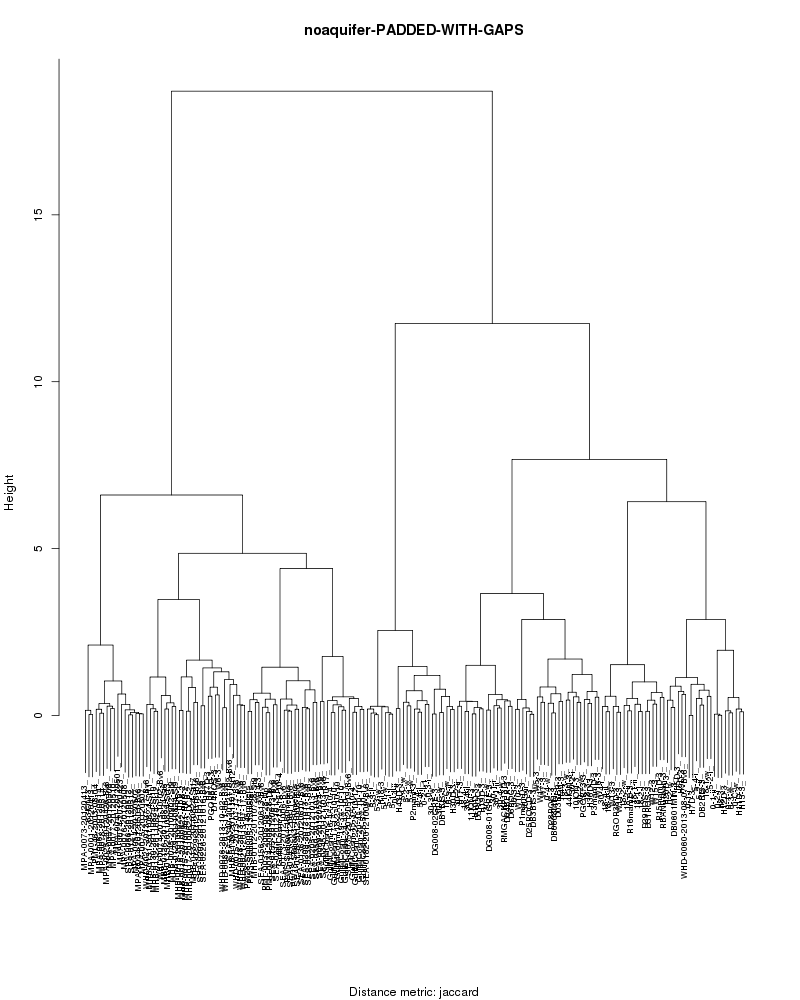

Supplement: Supplementary file 6 [file DataSheet2.ZIP › HTML-OUTPUT/basic_analyses-cluster_analysis-jaccard.png]

## noaquifer-PADDED-WITH-GAPS

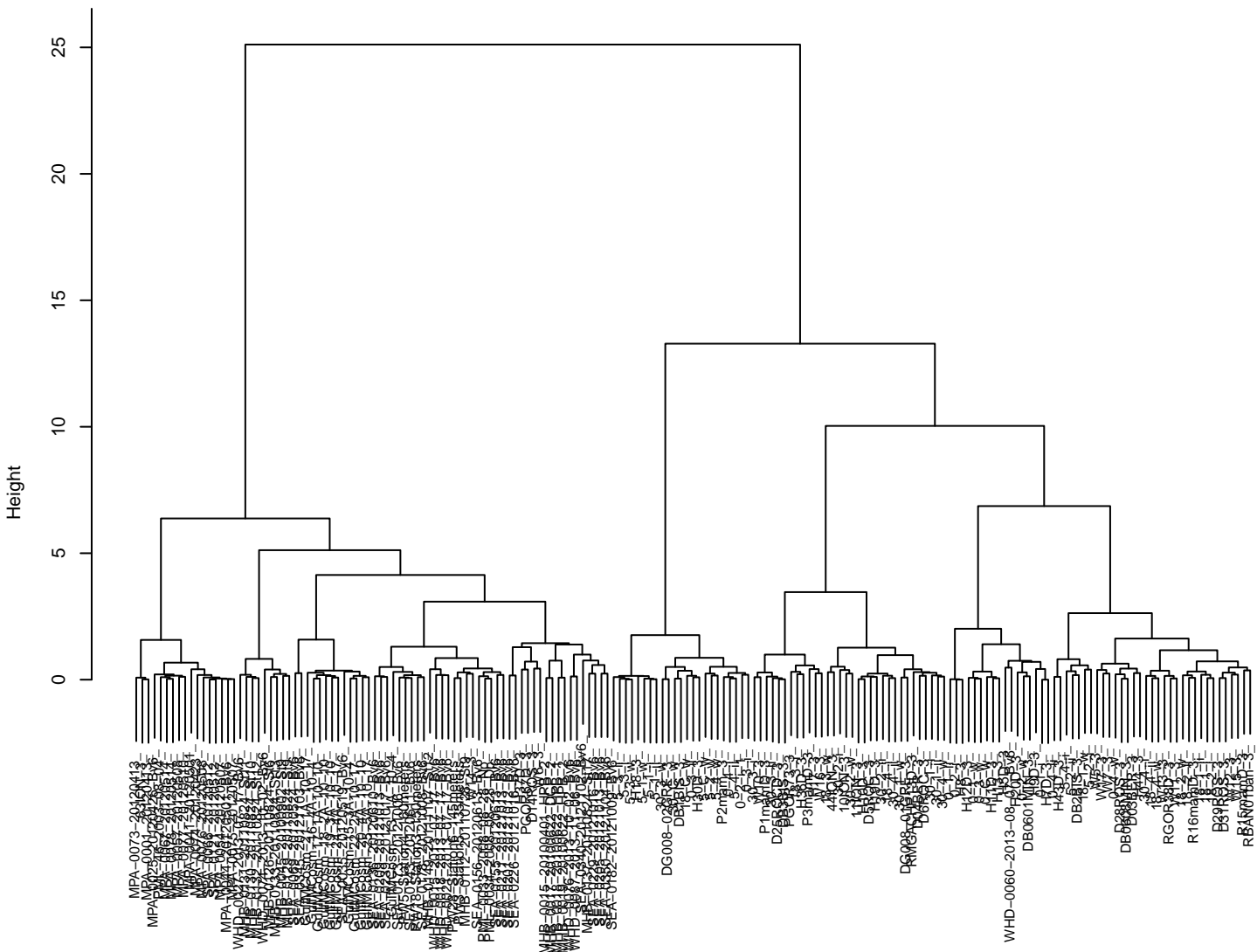

Distance metric: kulczynski

Supplement: Supplementary file 6 [file DataSheet2.ZIP › HTML-OUTPUT/basic_analyses-cluster_analysis-kulczynski.pdf]

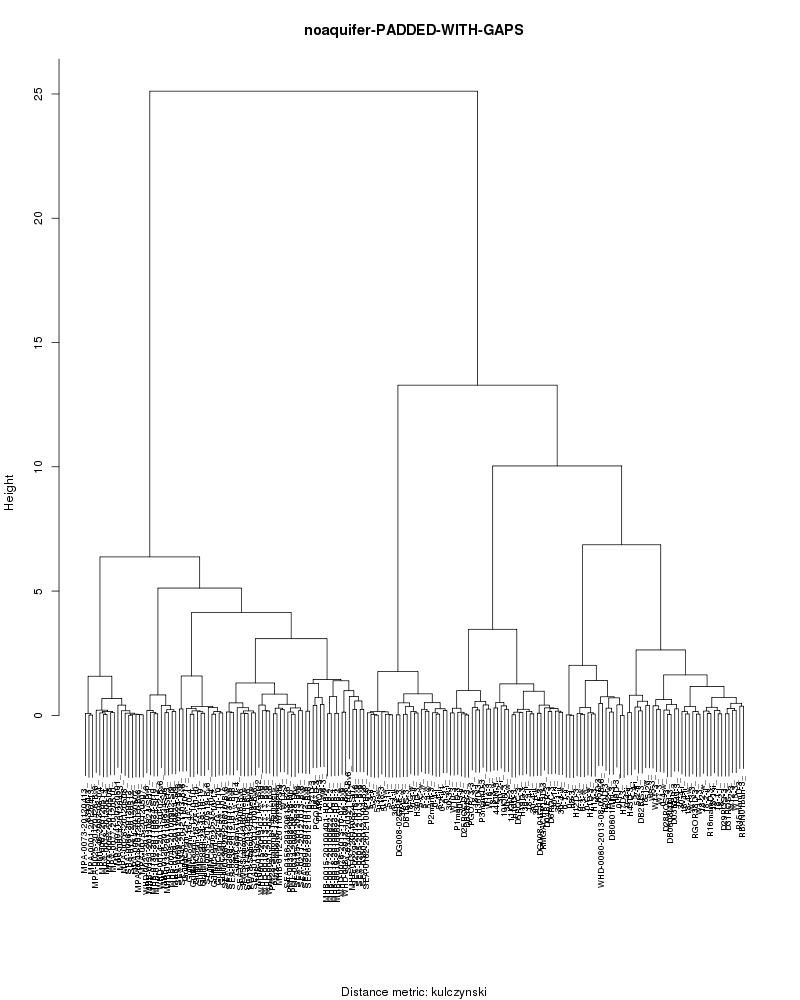

Supplement: Supplementary file 6 [file DataSheet2.ZIP › HTML-OUTPUT/basic_analyses-cluster_analysis-kulczynski.png]

## noaquifer-PADDED-WITH-GAPS

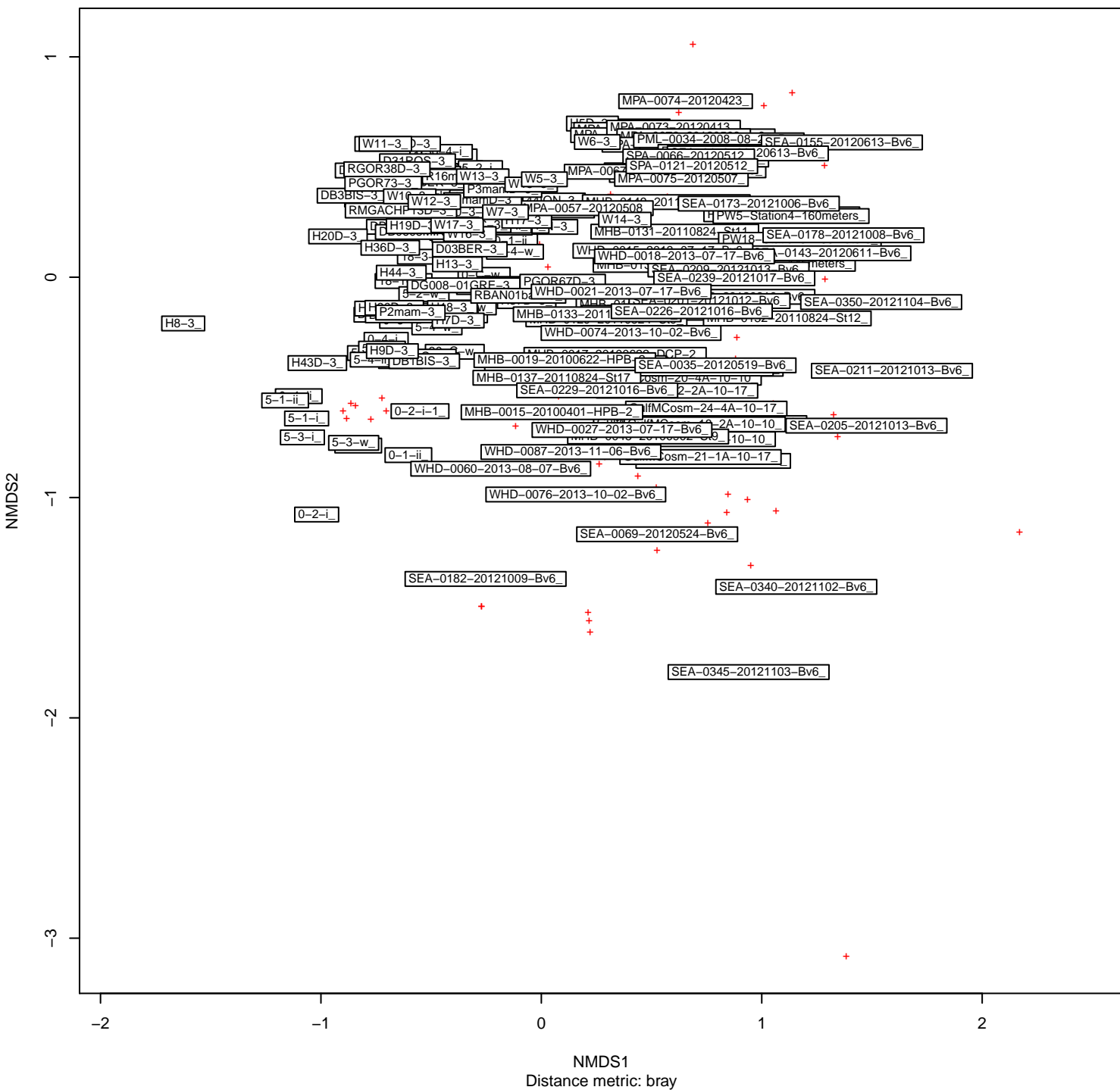

Supplement: Supplementary file 6 [file DataSheet2.ZIP › HTML-OUTPUT/basic_analyses-nmds_analysis-bray.pdf]

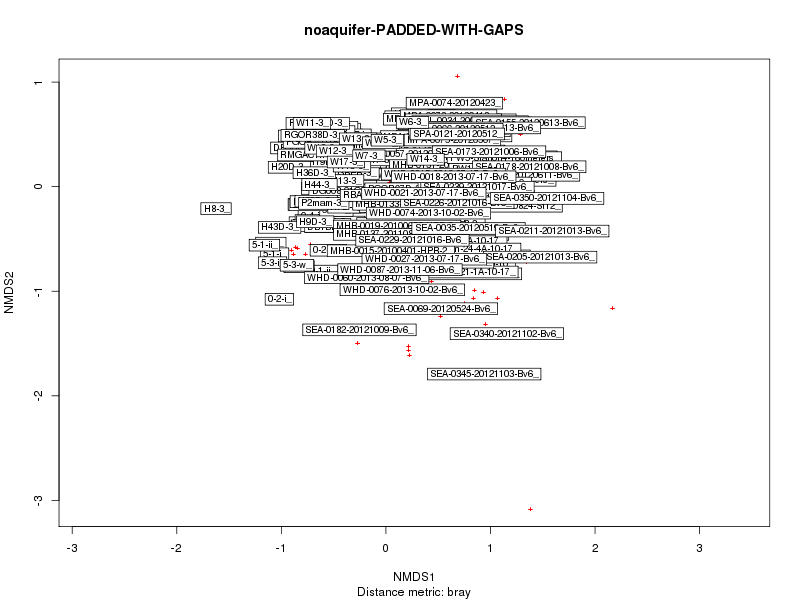

Supplement: Supplementary file 6 [file DataSheet2.ZIP › HTML-OUTPUT/basic_analyses-nmds_analysis-bray.png]

## noaquifer-PADDED-WITH-GAPS

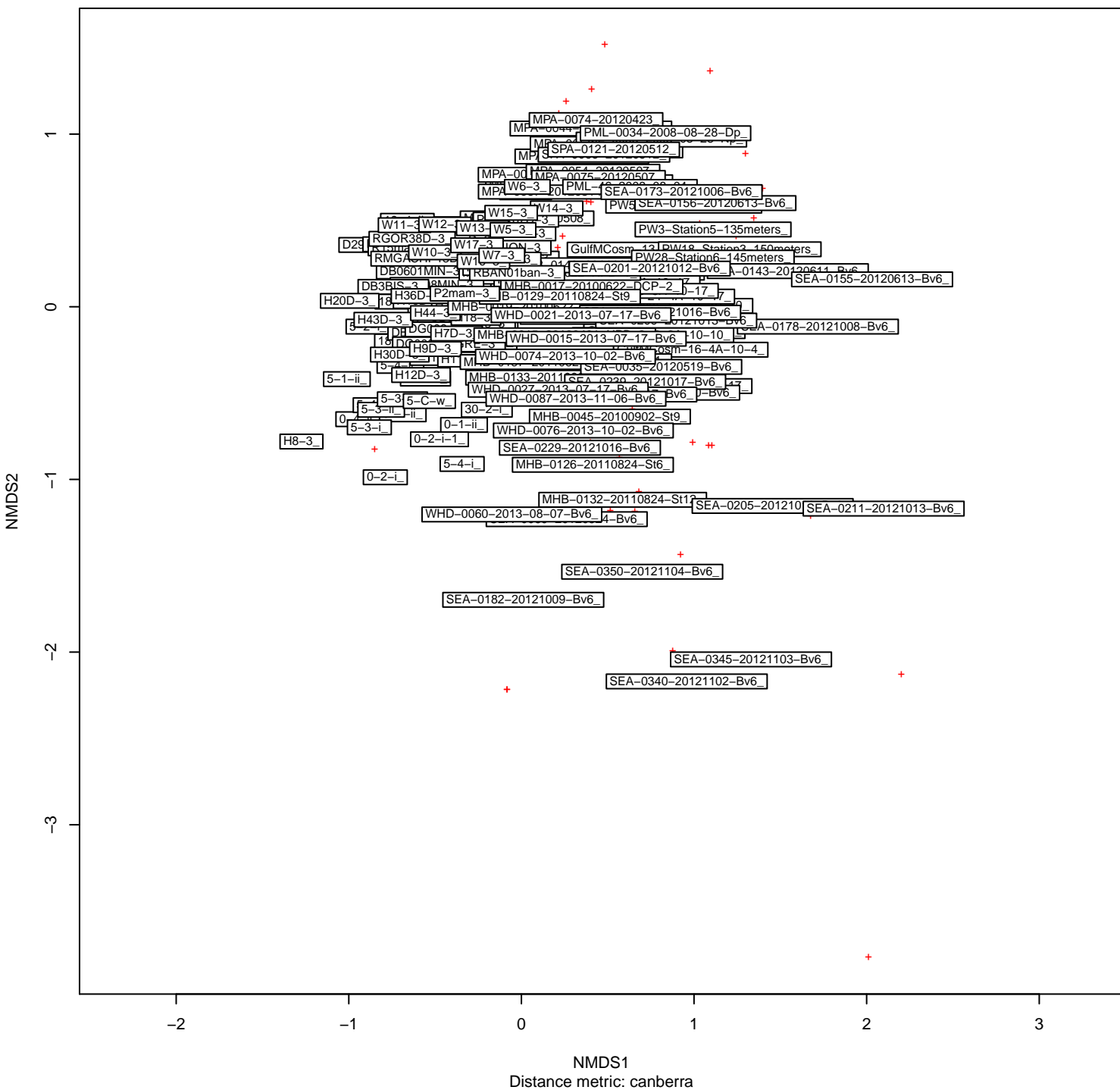

Supplement: Supplementary file 6 [file DataSheet2.ZIP › HTML-OUTPUT/basic_analyses-nmds_analysis-canberra.pdf]

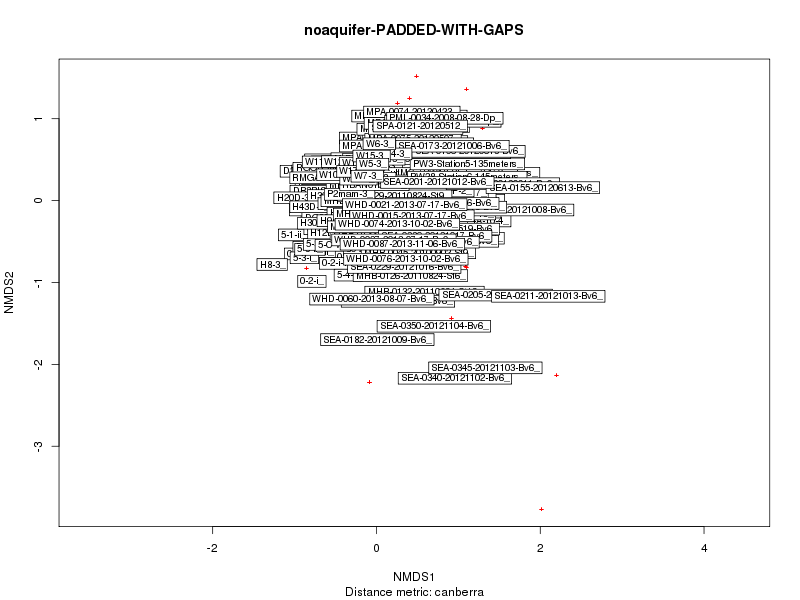

Supplement: Supplementary file 6 [file DataSheet2.ZIP › HTML-OUTPUT/basic_analyses-nmds_analysis-canberra.png]

## noaquifer-PADDED-WITH-GAPS

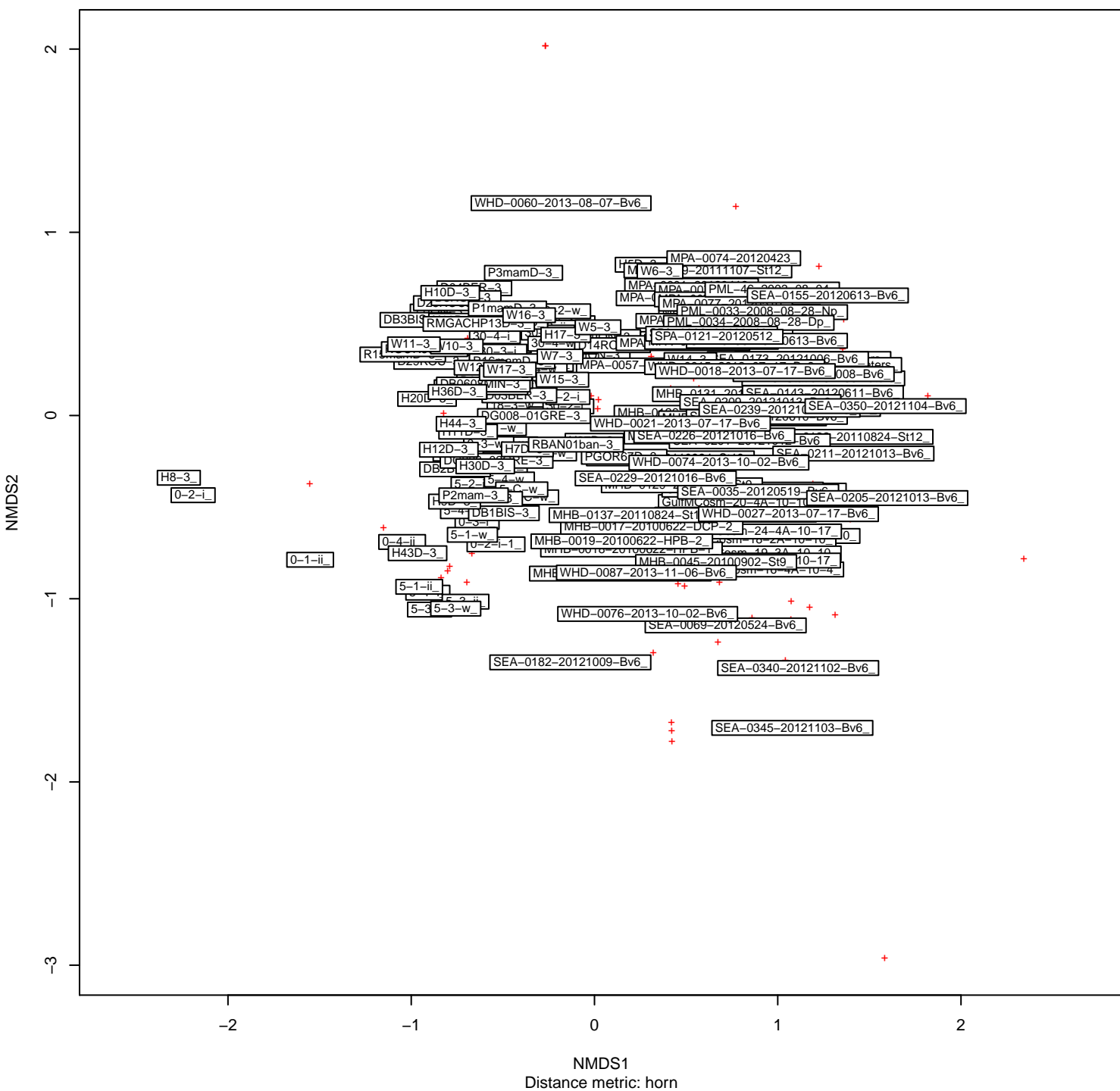

Supplement: Supplementary file 6 [file DataSheet2.ZIP › HTML-OUTPUT/basic_analyses-nmds_analysis-horn.pdf]

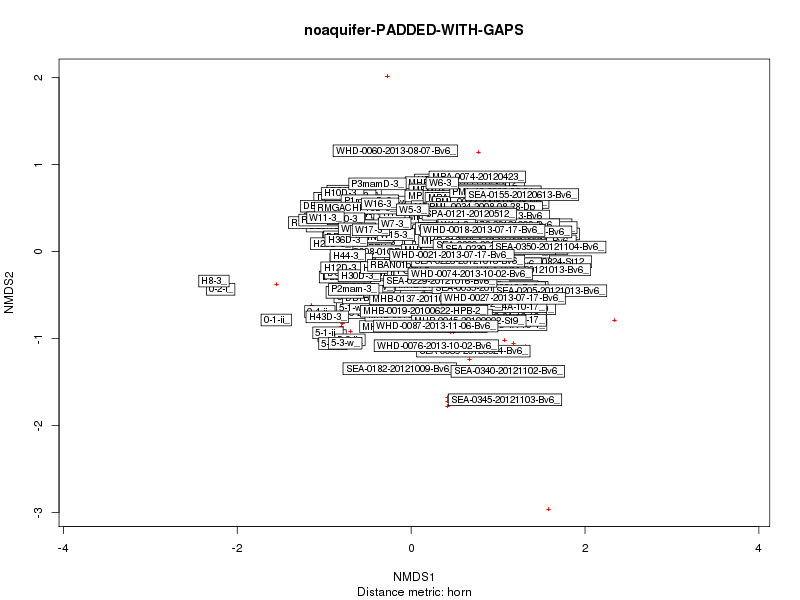

Supplement: Supplementary file 6 [file DataSheet2.ZIP › HTML-OUTPUT/basic_analyses-nmds_analysis-horn.png]

## noaquifer-PADDED-WITH-GAPS

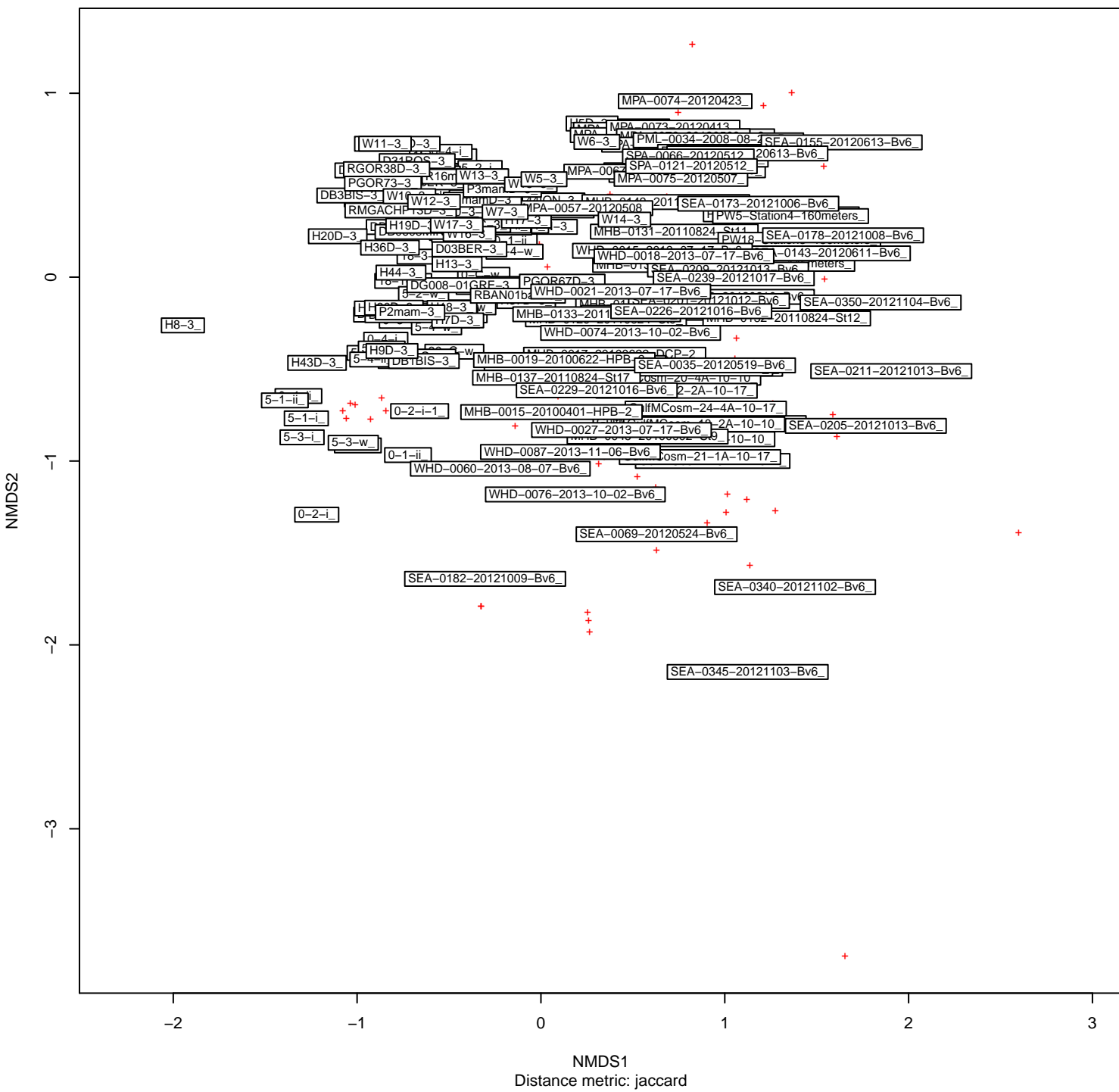

Supplement: Supplementary file 6 [file DataSheet2.ZIP › HTML-OUTPUT/basic_analyses-nmds_analysis-jaccard.pdf]

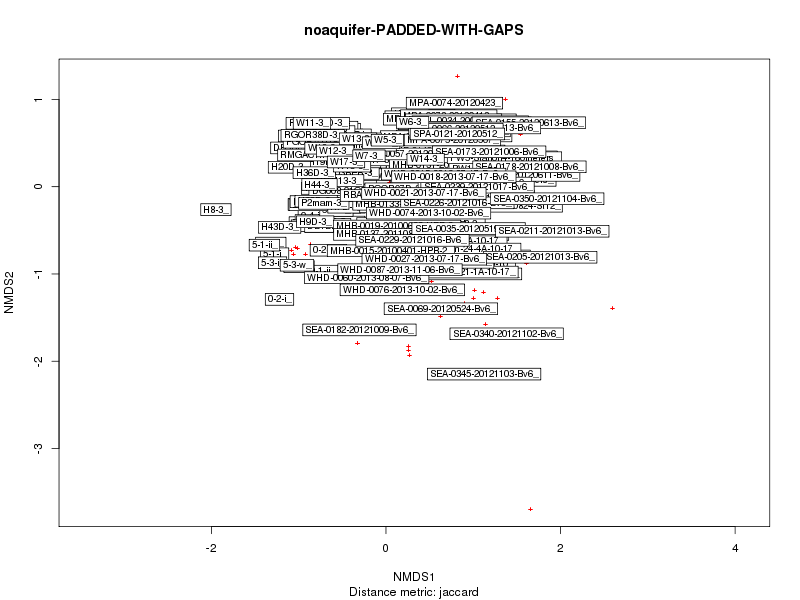

Supplement: Supplementary file 6 [file DataSheet2.ZIP › HTML-OUTPUT/basic_analyses-nmds_analysis-jaccard.png]

# noaquifer-PADDED-WITH-GAPS

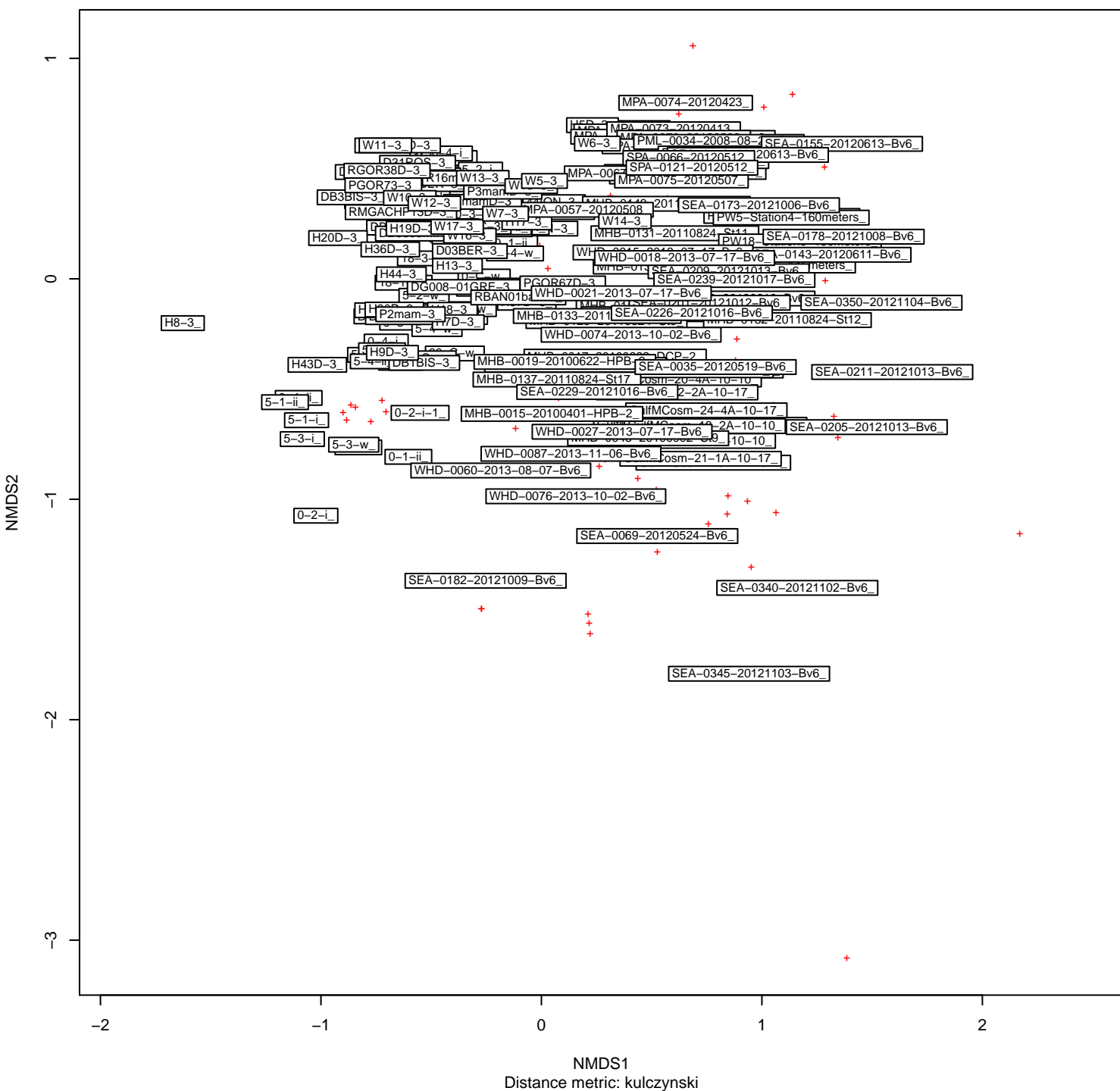

Supplement: Supplementary file 6 [file DataSheet2.ZIP › HTML-OUTPUT/basic_analyses-nmds_analysis-kulczynski.pdf]

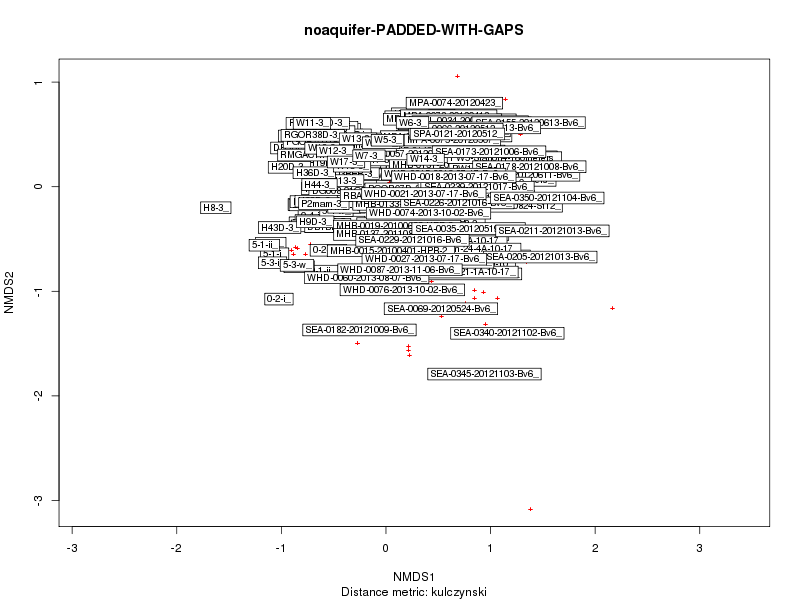

Supplement: Supplementary file 6 [file DataSheet2.ZIP › HTML-OUTPUT/basic_analyses-nmds_analysis-kulczynski.png]

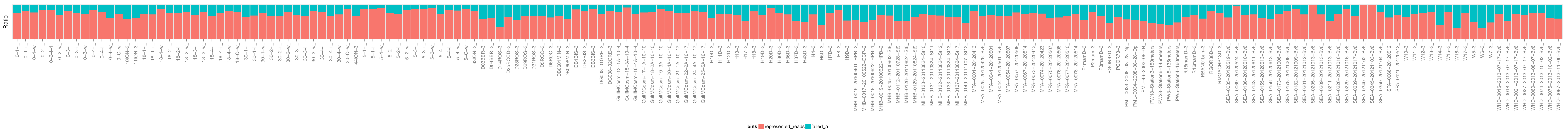

Supplement: Supplementary file 6 [file DataSheet2.ZIP › HTML-OUTPUT/basic_reports-bars-bars.pdf]

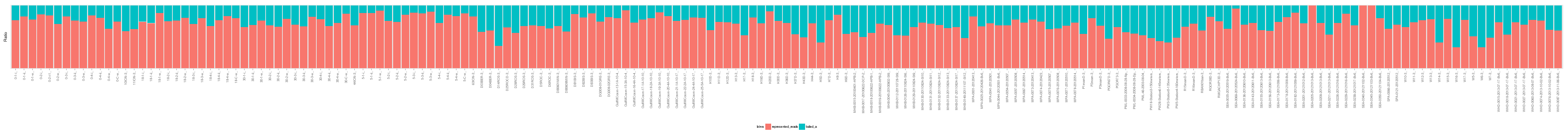

Supplement: Supplementary file 6 [file DataSheet2.ZIP › HTML-OUTPUT/basic_reports-bars-bars.png]

Number of Reads

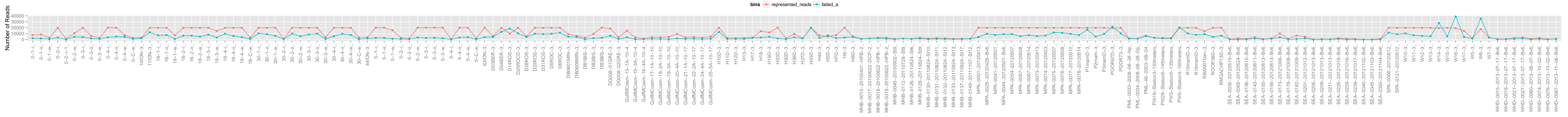

Supplement: Supplementary file 6 [file DataSheet2.ZIP › HTML-OUTPUT/basic_reports-lines-lines.pdf]

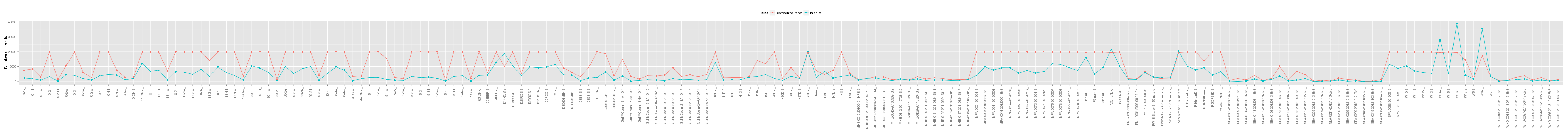

Supplement: Supplementary file 6 [file DataSheet2.ZIP › HTML-OUTPUT/basic_reports-lines-lines.png]

Distribution of Oligotypes Among Samples for noaquifer-PADDED-WITH-GAPS

Oligotypes

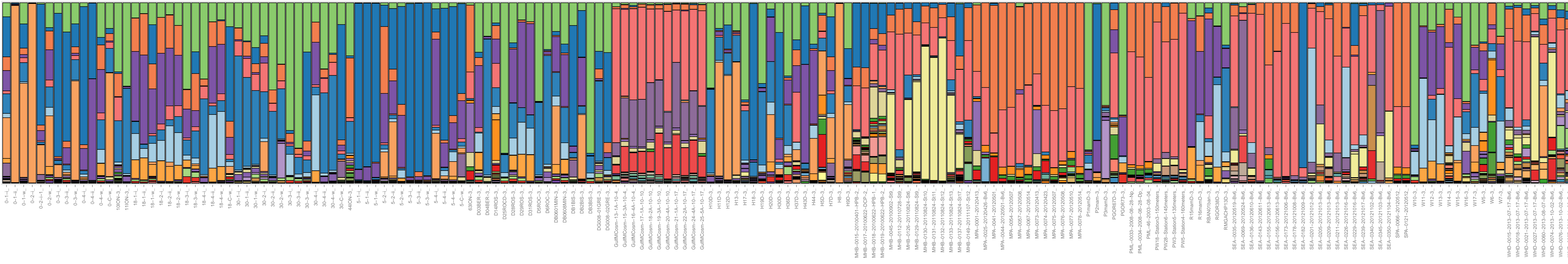

Supplement: Supplementary file 6 [file DataSheet2.ZIP › HTML-OUTPUT/basic_reports-stackbar-stackbar.pdf]

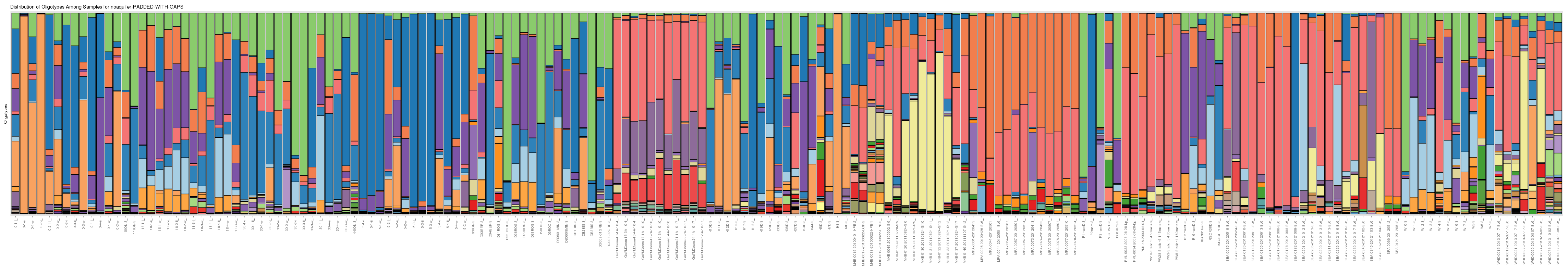

Supplement: Supplementary file 6 [file DataSheet2.ZIP › HTML-OUTPUT/basic_reports-stackbar-stackbar.png]

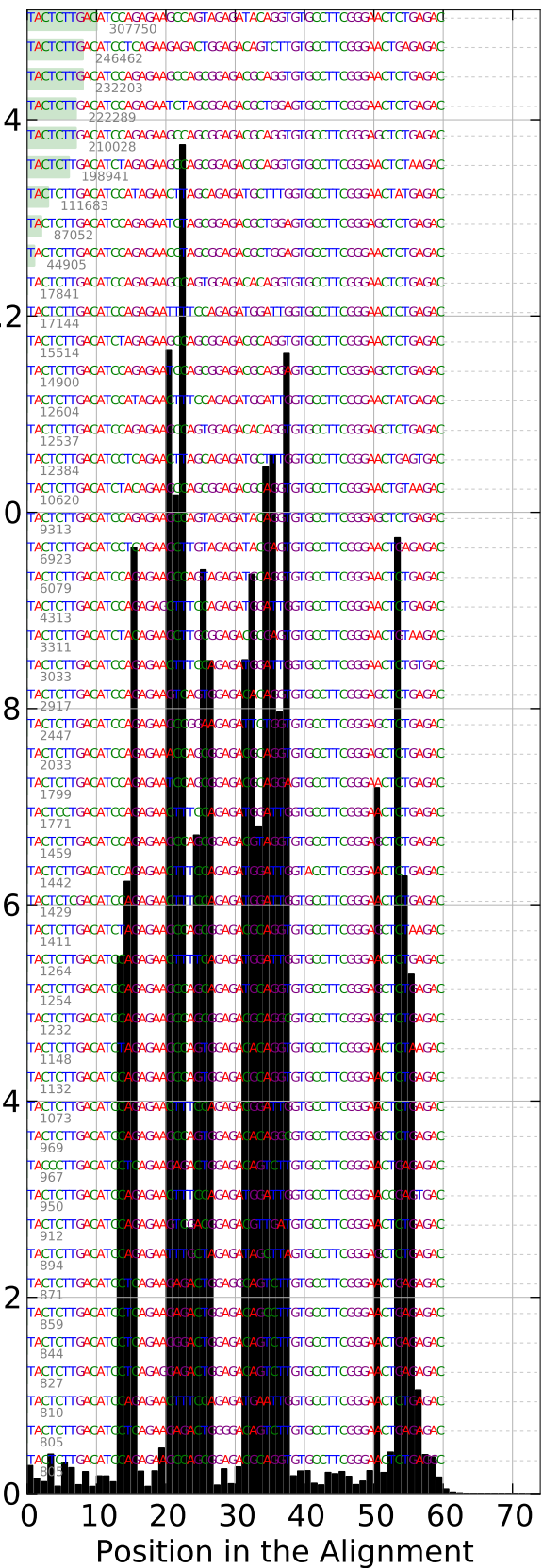

Supplement: Supplementary file 6 [file DataSheet2.ZIP › HTML-OUTPUT/entropy.pdf]

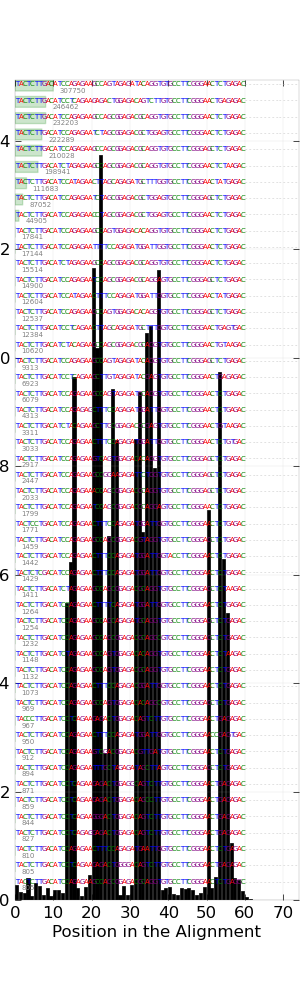

Supplement: Supplementary file 6 [file DataSheet2.ZIP › HTML-OUTPUT/entropy.png]

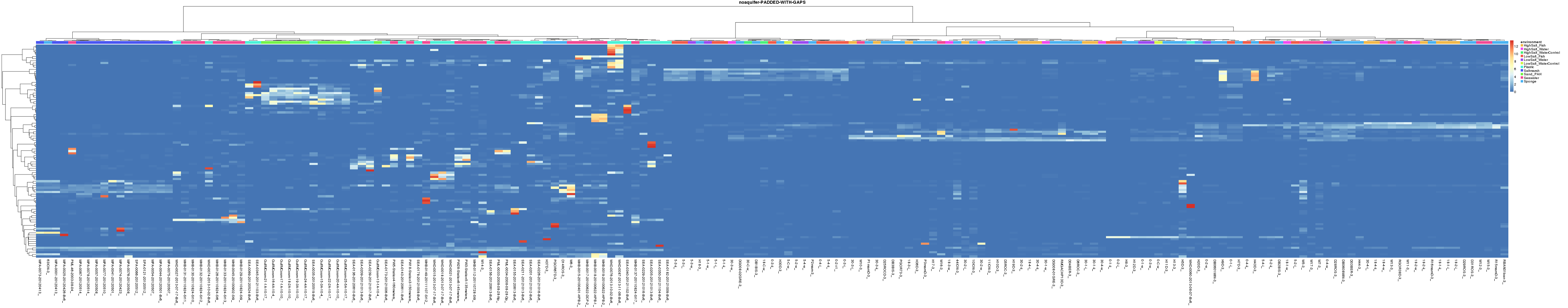

Supplement: Supplementary file 6 [file DataSheet2.ZIP › HTML-OUTPUT/environment-heatmap_analysis-bray.png]

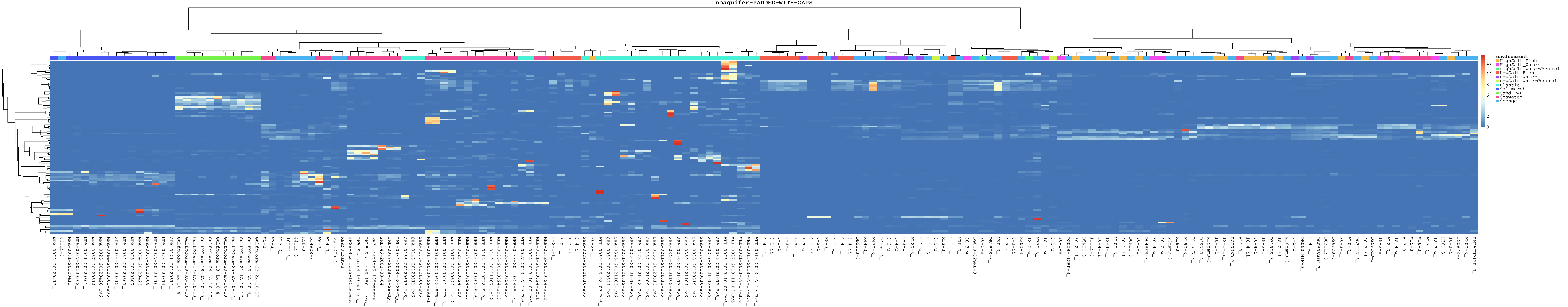

Supplement: Supplementary file 6 [file DataSheet2.ZIP › HTML-OUTPUT/environment-heatmap_analysis-canberra.pdf]

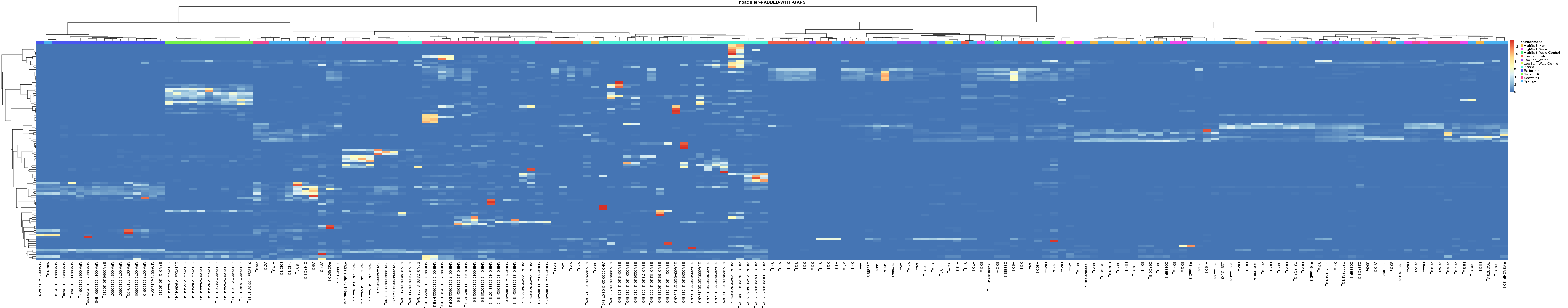

Supplement: Supplementary file 6 [file DataSheet2.ZIP › HTML-OUTPUT/environment-heatmap_analysis-canberra.png]

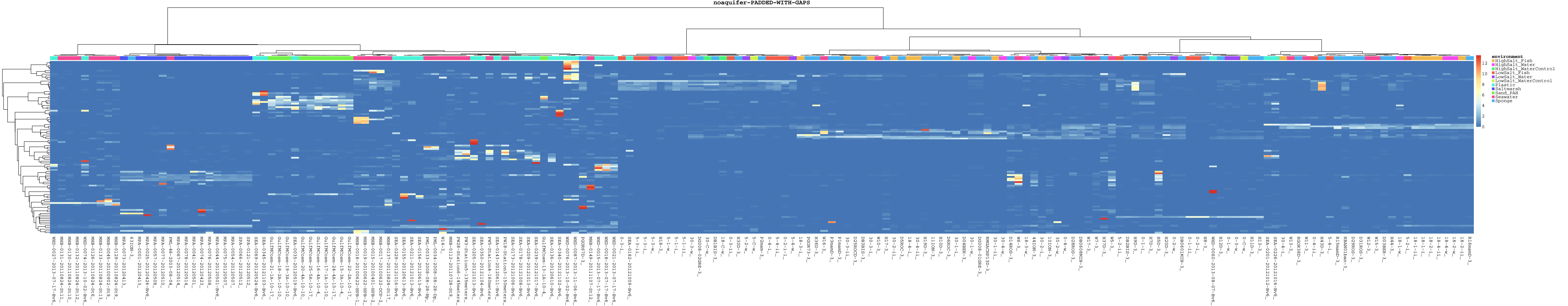

Supplement: Supplementary file 6 [file DataSheet2.ZIP › HTML-OUTPUT/environment-heatmap_analysis-horn.pdf]

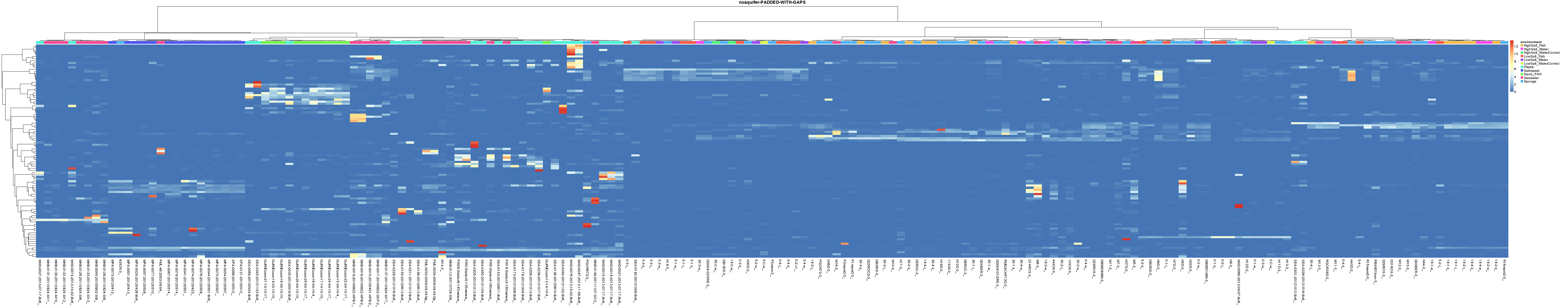

Supplement: Supplementary file 6 [file DataSheet2.ZIP › HTML-OUTPUT/environment-heatmap_analysis-horn.png]

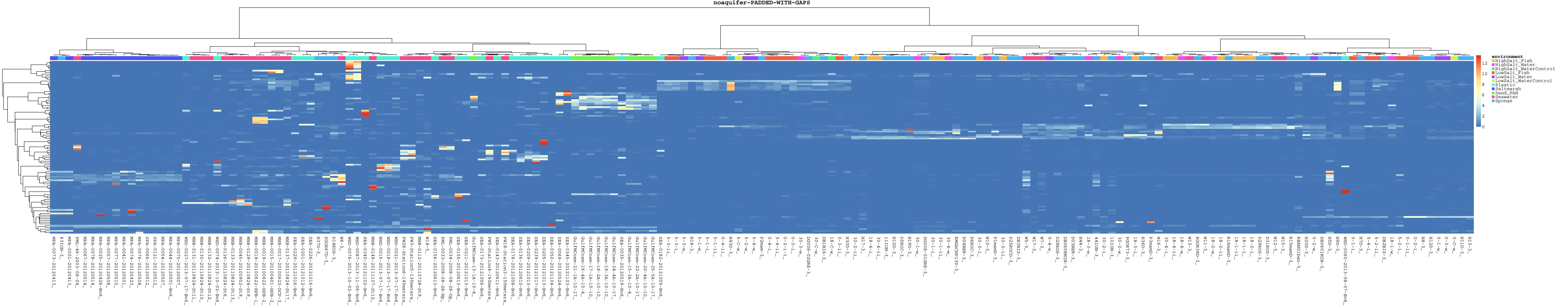

Supplement: Supplementary file 6 [file DataSheet2.ZIP › HTML-OUTPUT/environment-heatmap_analysis-jaccard.pdf]

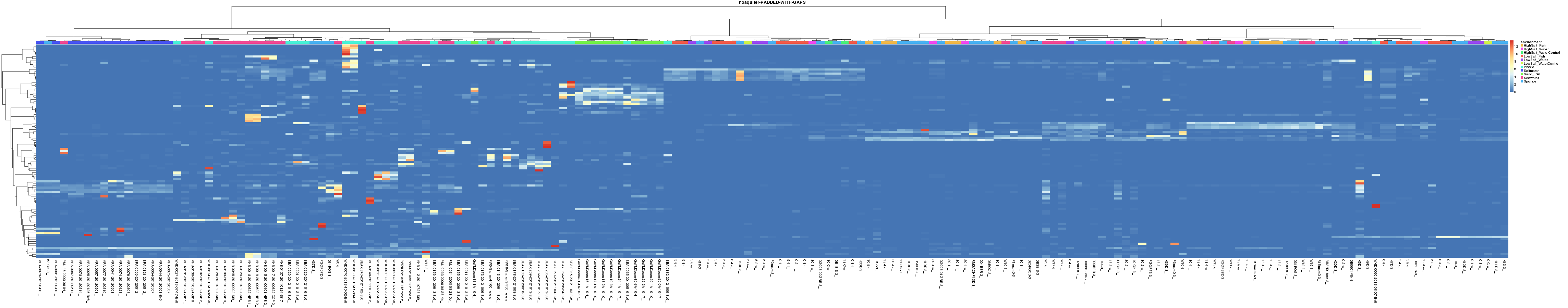

Supplement: Supplementary file 6 [file DataSheet2.ZIP › HTML-OUTPUT/environment-heatmap_analysis-jaccard.png]

noaquifer-PADDED-WITH-GAPS

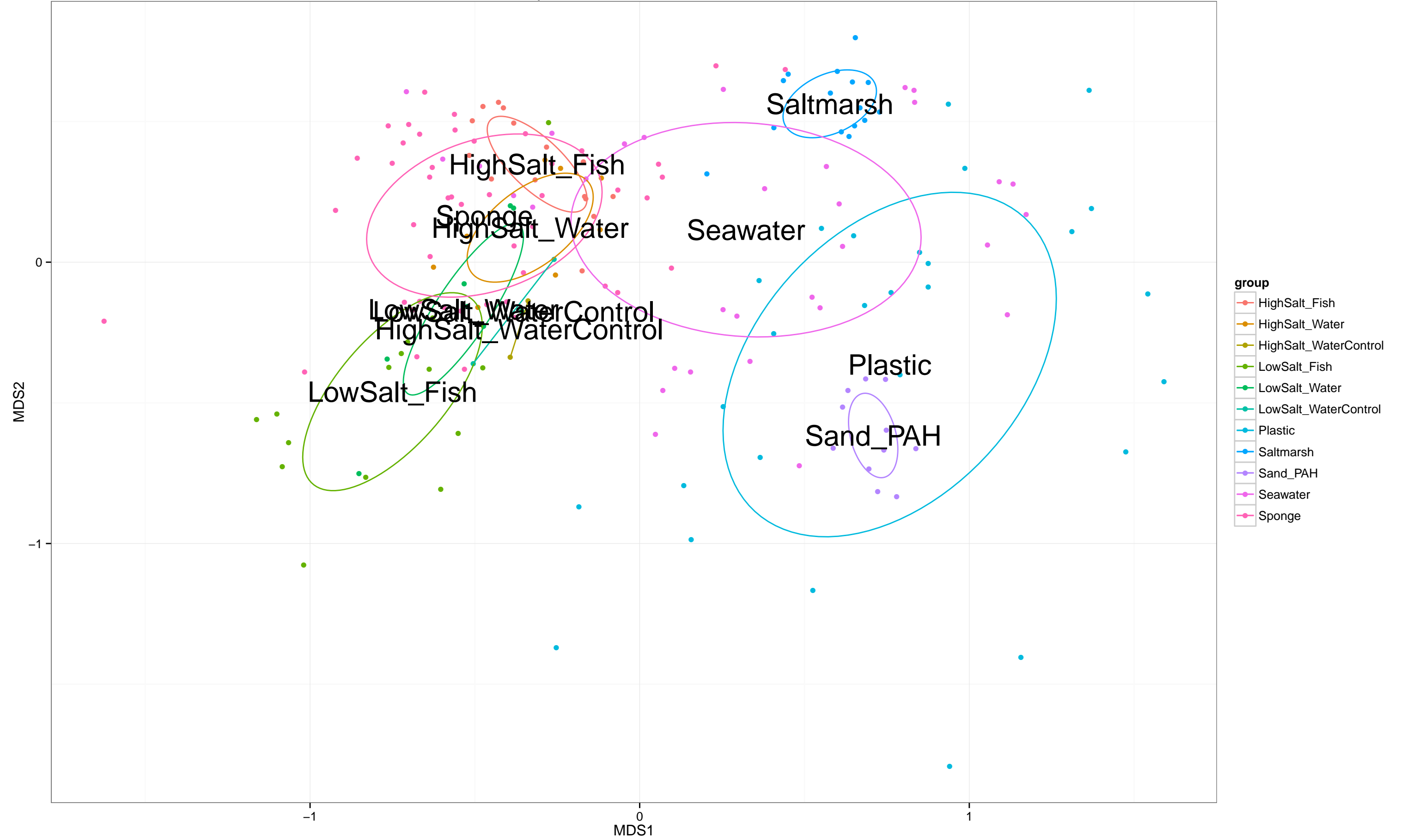

Supplement: Supplementary file 6 [file DataSheet2.ZIP › HTML-OUTPUT/environment-nmds_analysis-bray.pdf]

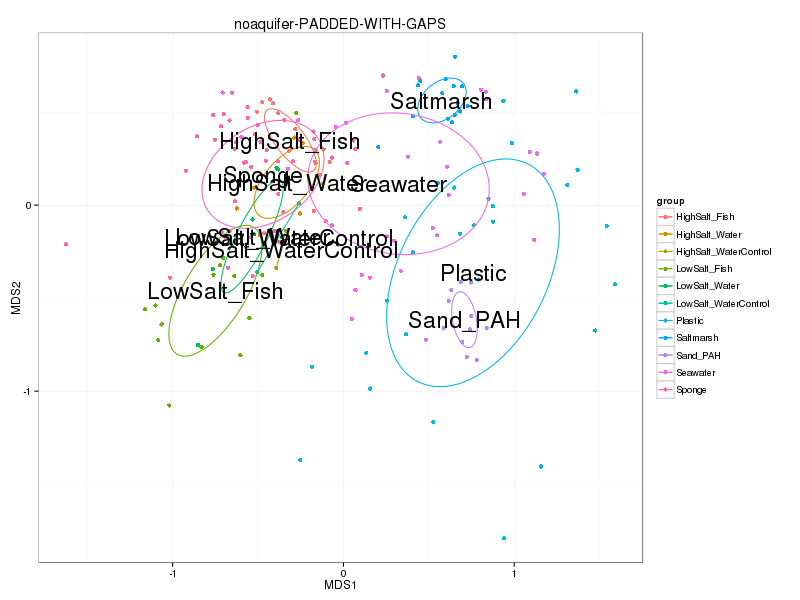

Supplement: Supplementary file 6 [file DataSheet2.ZIP › HTML-OUTPUT/environment-nmds_analysis-bray.png]

noaquifer-PADDED-WITH-GAPS

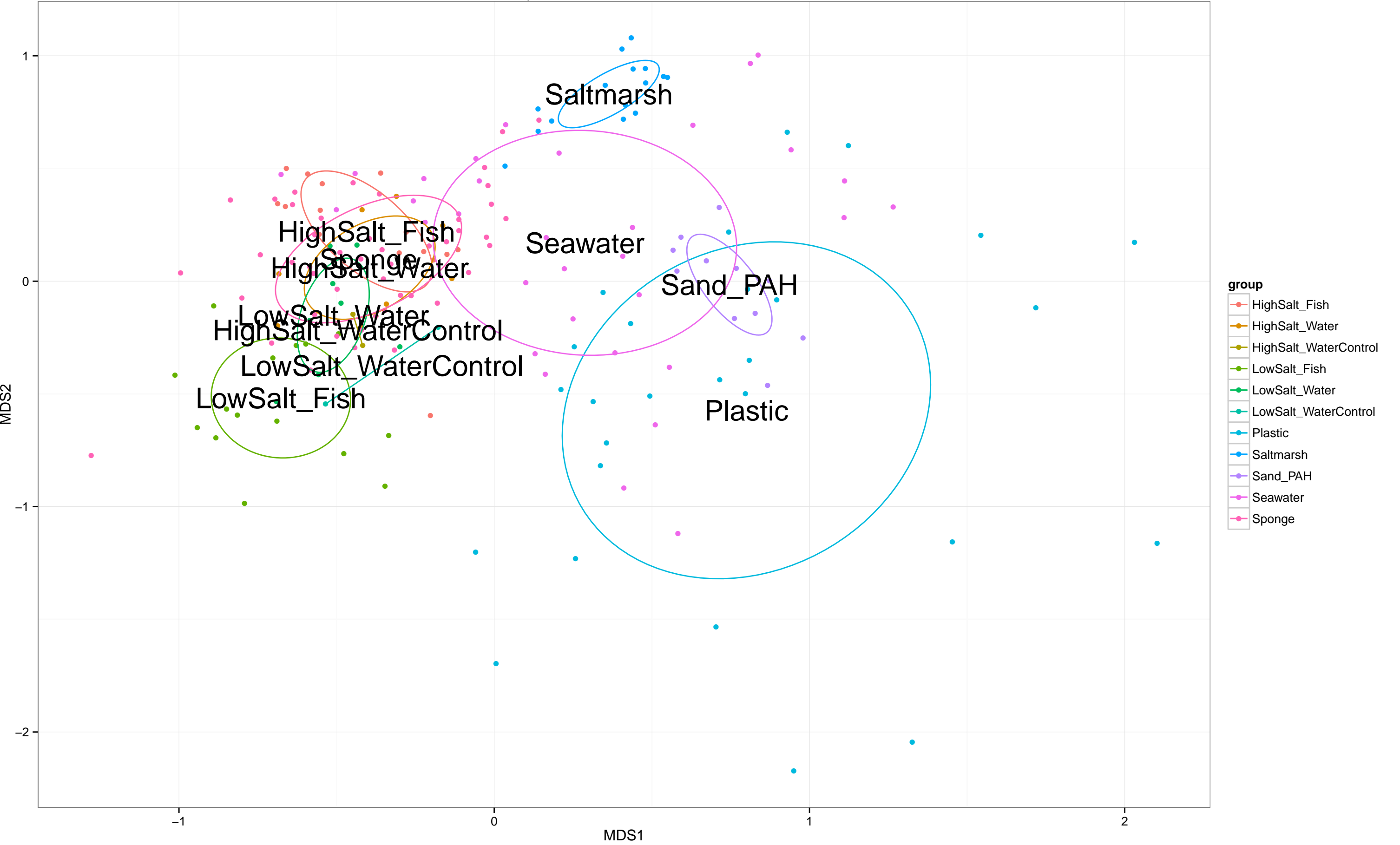

Supplement: Supplementary file 6 [file DataSheet2.ZIP › HTML-OUTPUT/environment-nmds_analysis-canberra.pdf]

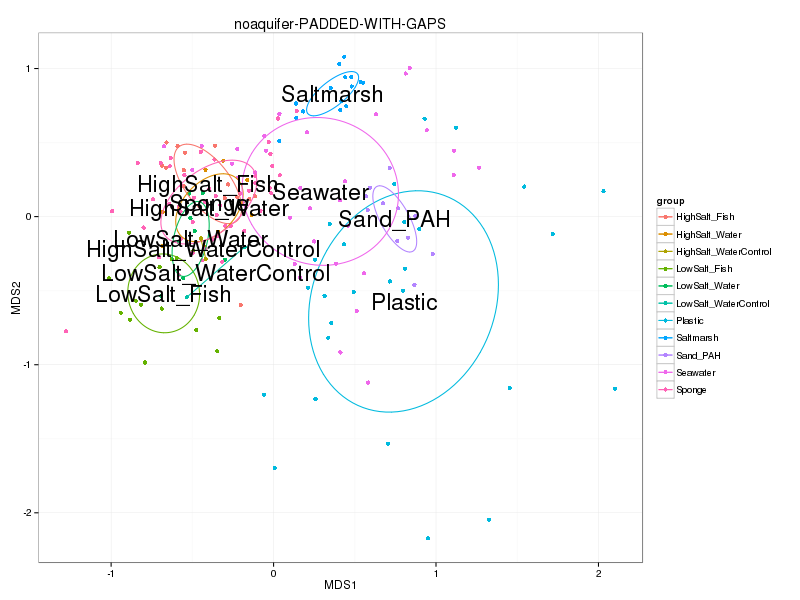

Supplement: Supplementary file 6 [file DataSheet2.ZIP › HTML-OUTPUT/environment-nmds_analysis-canberra.png]

noaquifer-PADDED-WITH-GAPS

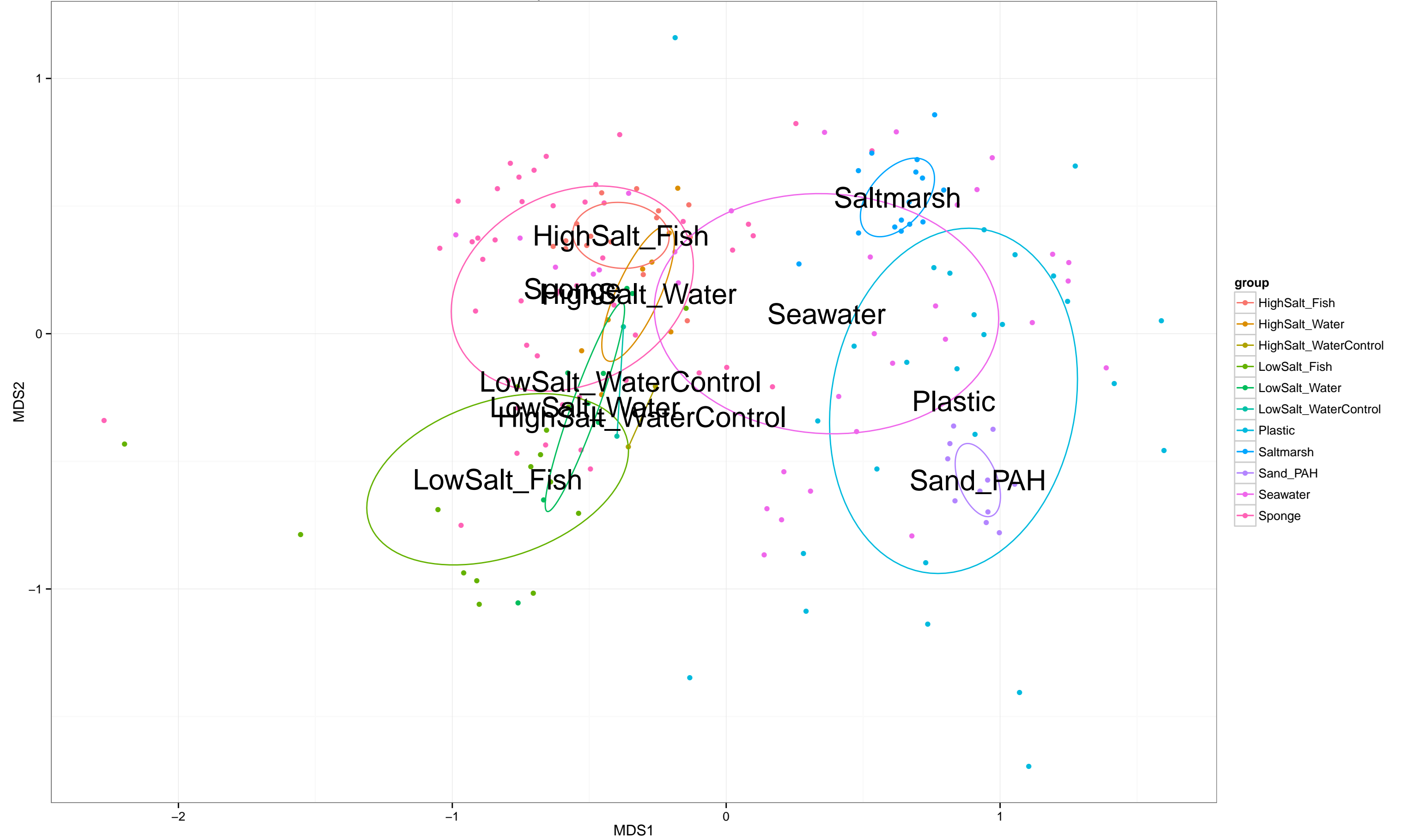

Supplement: Supplementary file 6 [file DataSheet2.ZIP › HTML-OUTPUT/environment-nmds_analysis-horn.pdf]

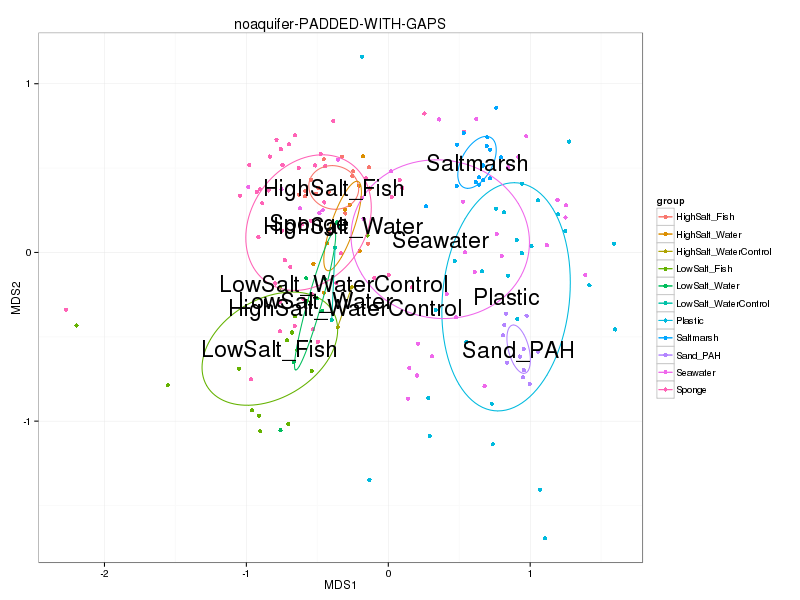

Supplement: Supplementary file 6 [file DataSheet2.ZIP › HTML-OUTPUT/environment-nmds_analysis-horn.png]

noaquifer-PADDED-WITH-GAPS

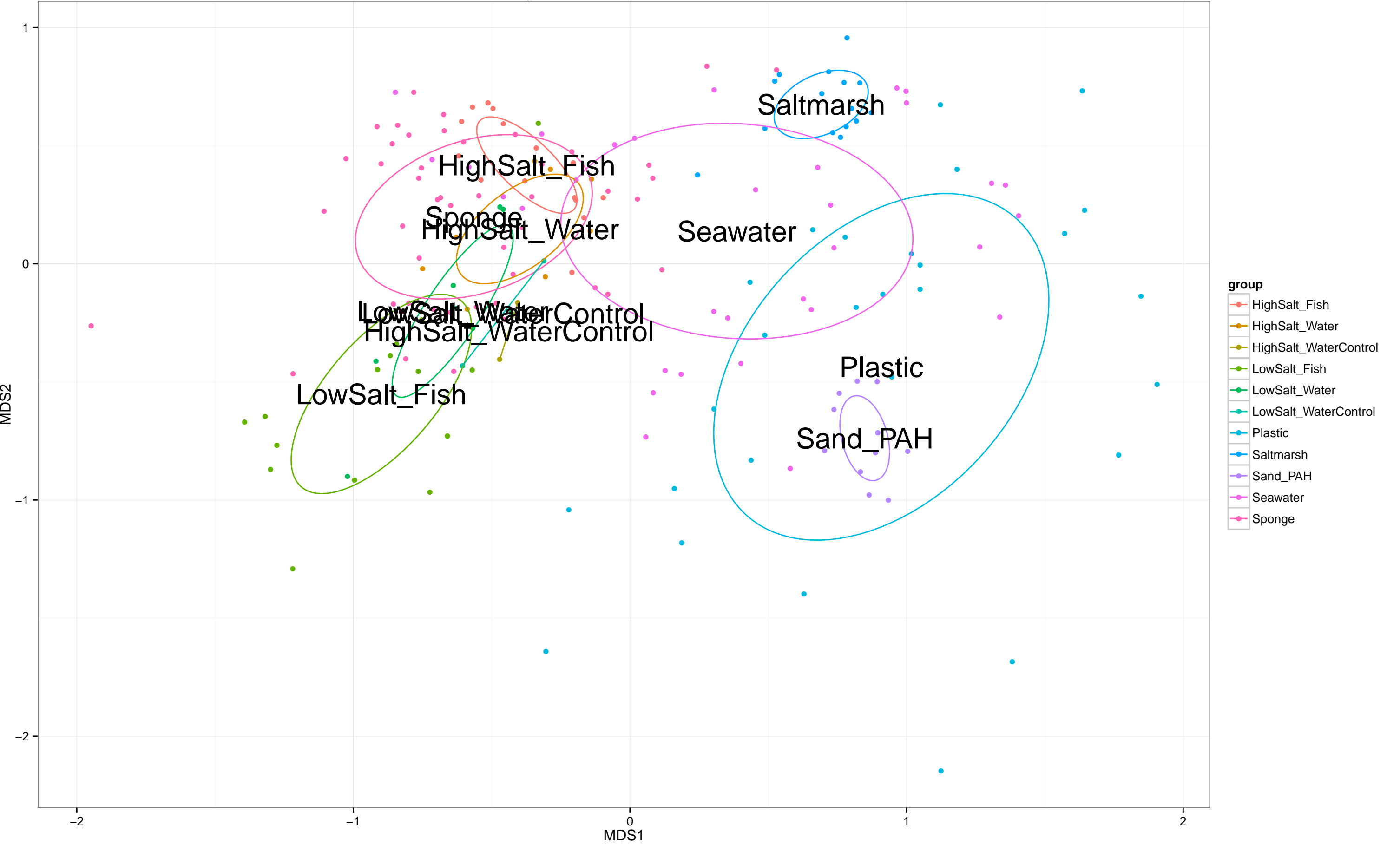

Supplement: Supplementary file 6 [file DataSheet2.ZIP › HTML-OUTPUT/environment-nmds_analysis-jaccard.pdf]

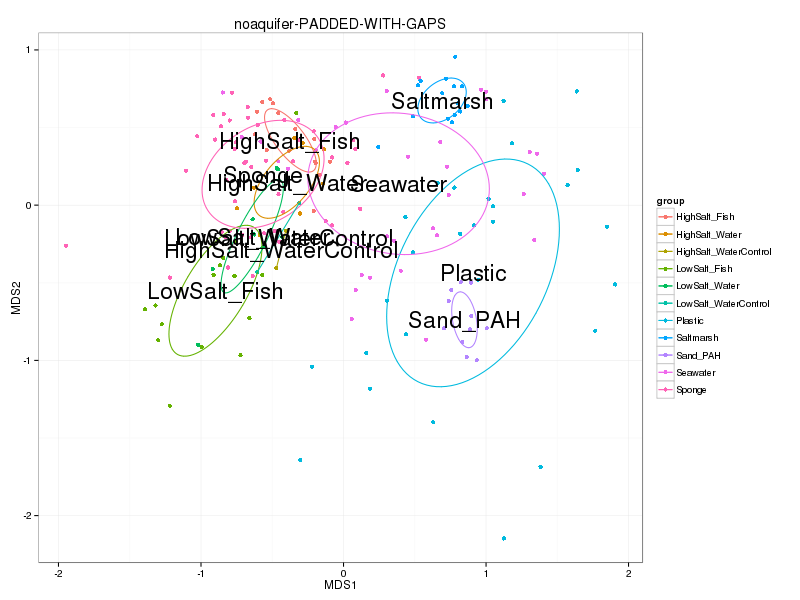

Supplement: Supplementary file 6 [file DataSheet2.ZIP › HTML-OUTPUT/environment-nmds_analysis-jaccard.png]

noaquifer-PADDED-WITH-GAPS

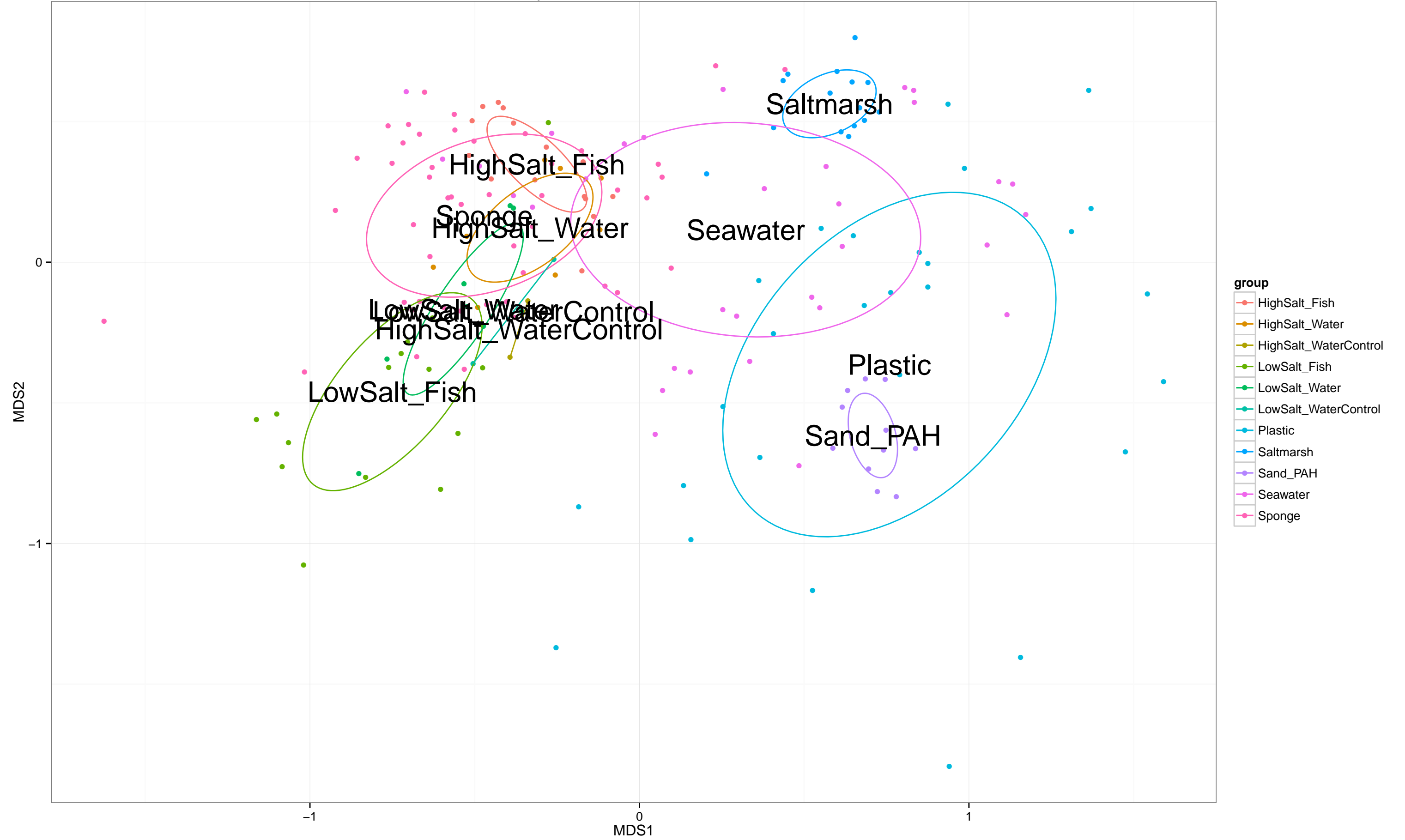

Supplement: Supplementary file 6 [file DataSheet2.ZIP › HTML-OUTPUT/environment-nmds_analysis-kulczynski.pdf]

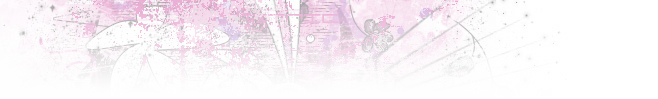

Supplement: Supplementary file 6 [file DataSheet2.ZIP › HTML-OUTPUT/header.png]

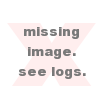

Supplement: Supplementary file 6 [file DataSheet2.ZIP › HTML-OUTPUT/missing.png]
