# Supplementary figures and images for: Oligotyping reveals community level habitat selection within the genus Vibrio (part 1 of 2)
Source: Front Microbiol. 2014 Nov 13;5:563. doi: 10.3389/fmicb.2014.00563 (PMC4230168; doi:10.3389/fmicb.2014.00563)

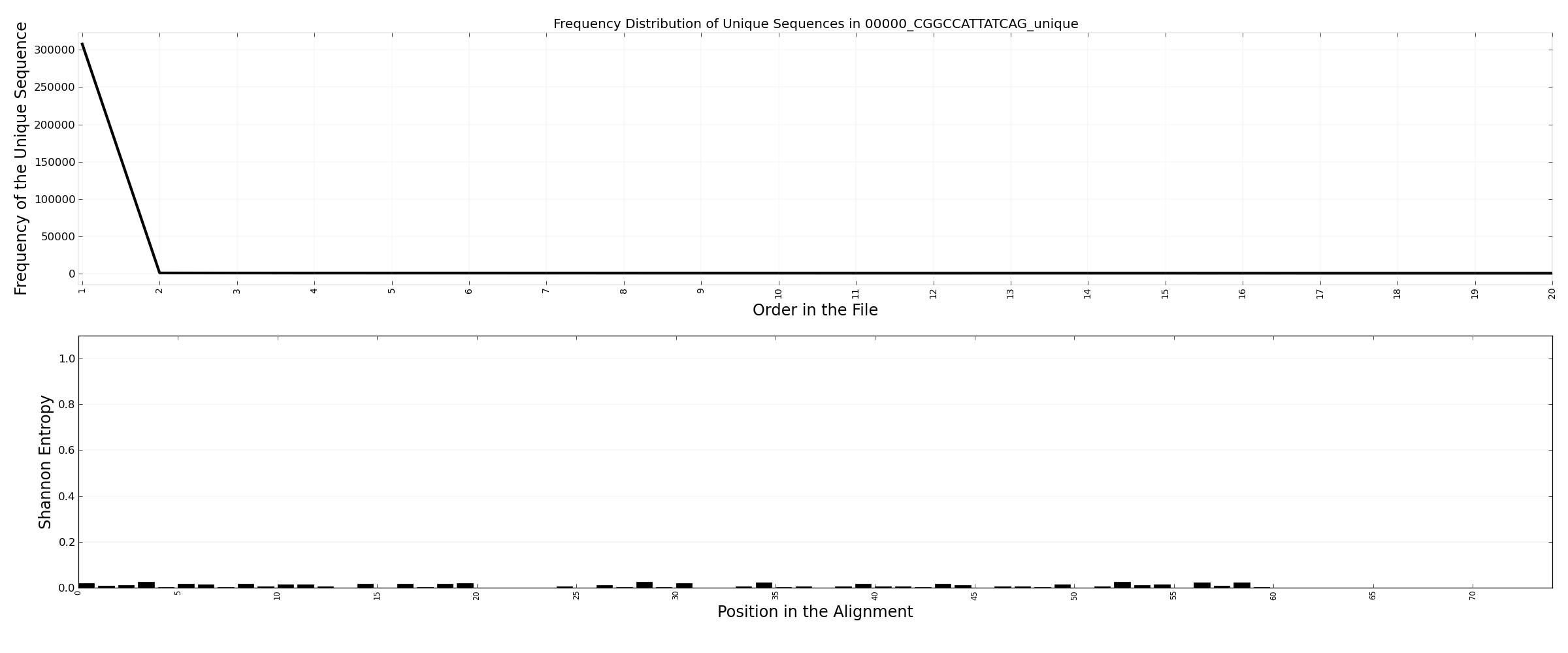

Supplement: Supplementary file 6 [file DataSheet2.ZIP › HTML-OUTPUT/00000_CGGCCATTATCAG_unique.png]

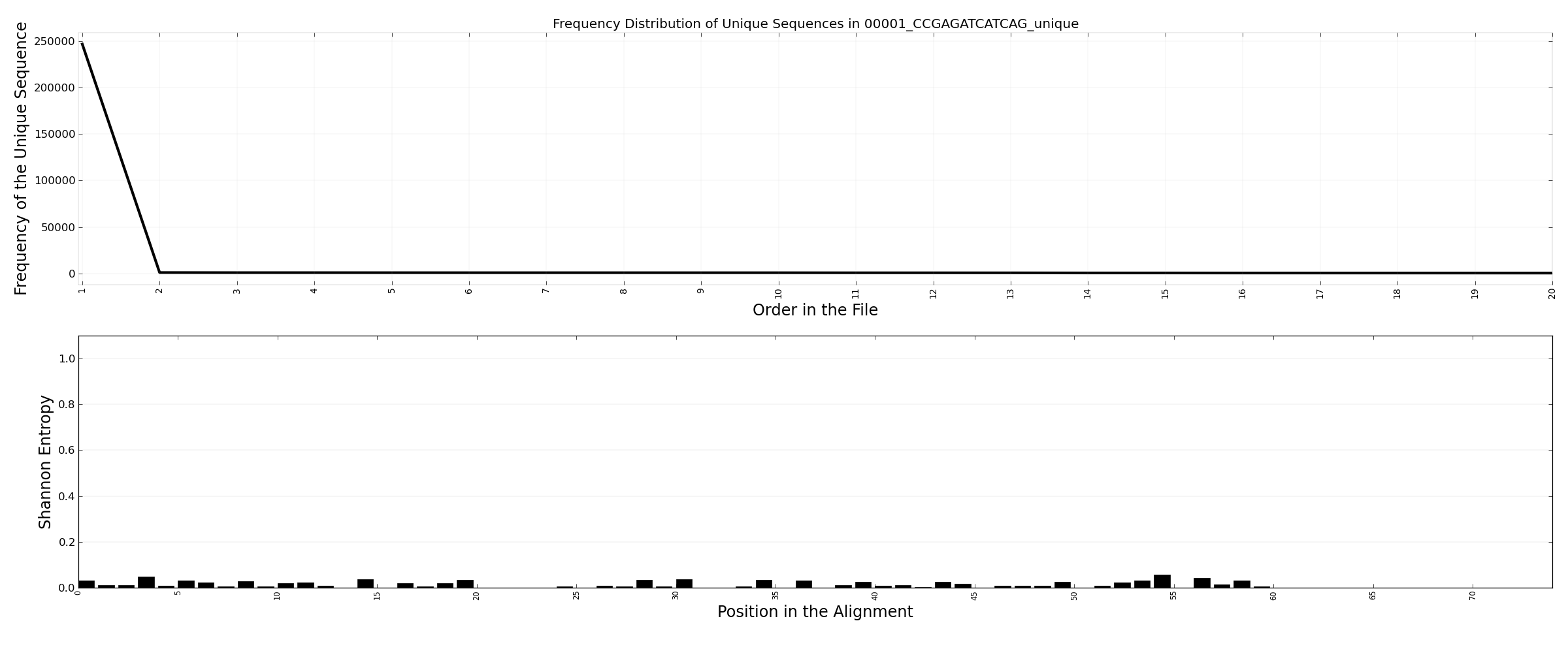

Supplement: Supplementary file 6 [file DataSheet2.ZIP › HTML-OUTPUT/00001_CCGAGATCATCAG_unique.png]

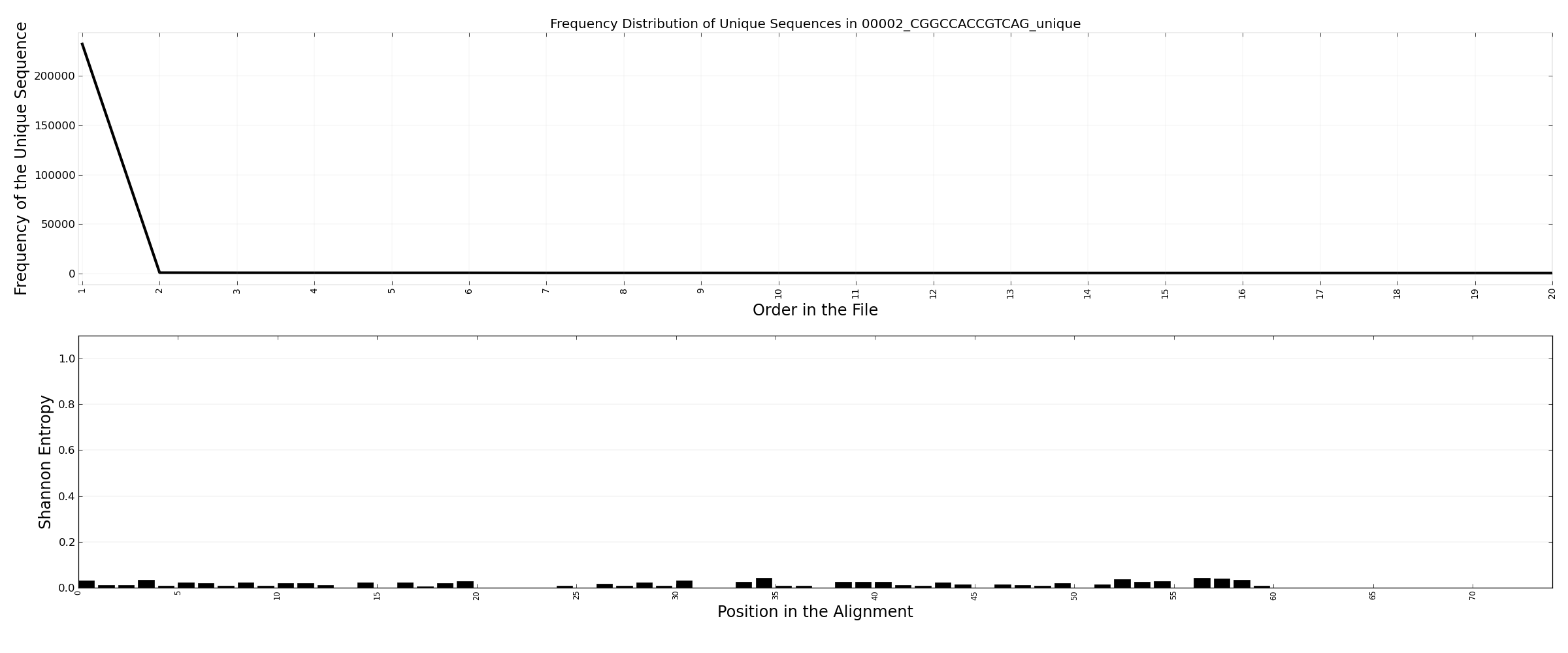

Supplement: Supplementary file 6 [file DataSheet2.ZIP › HTML-OUTPUT/00002_CGGCCACCGTCAG_unique.png]

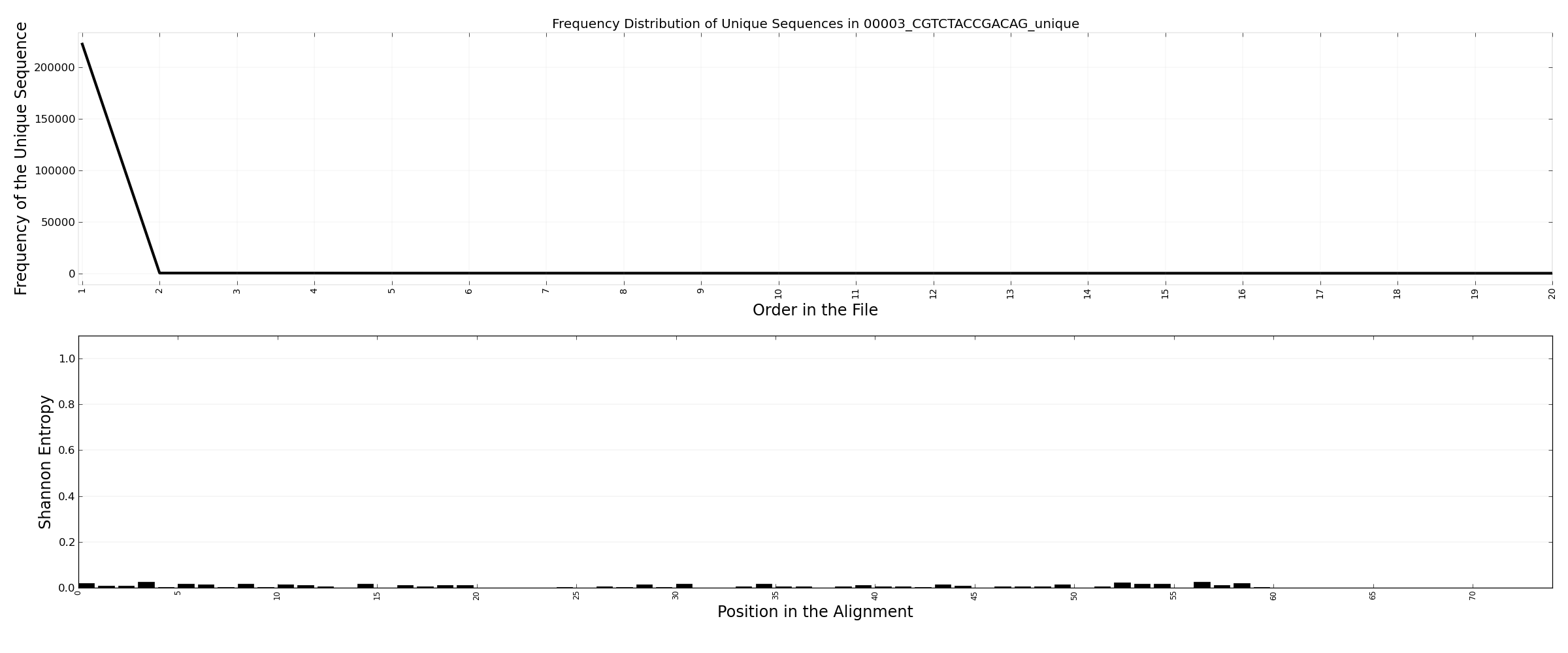

Supplement: Supplementary file 6 [file DataSheet2.ZIP › HTML-OUTPUT/00003_CGTCTACCGACAG_unique.png]

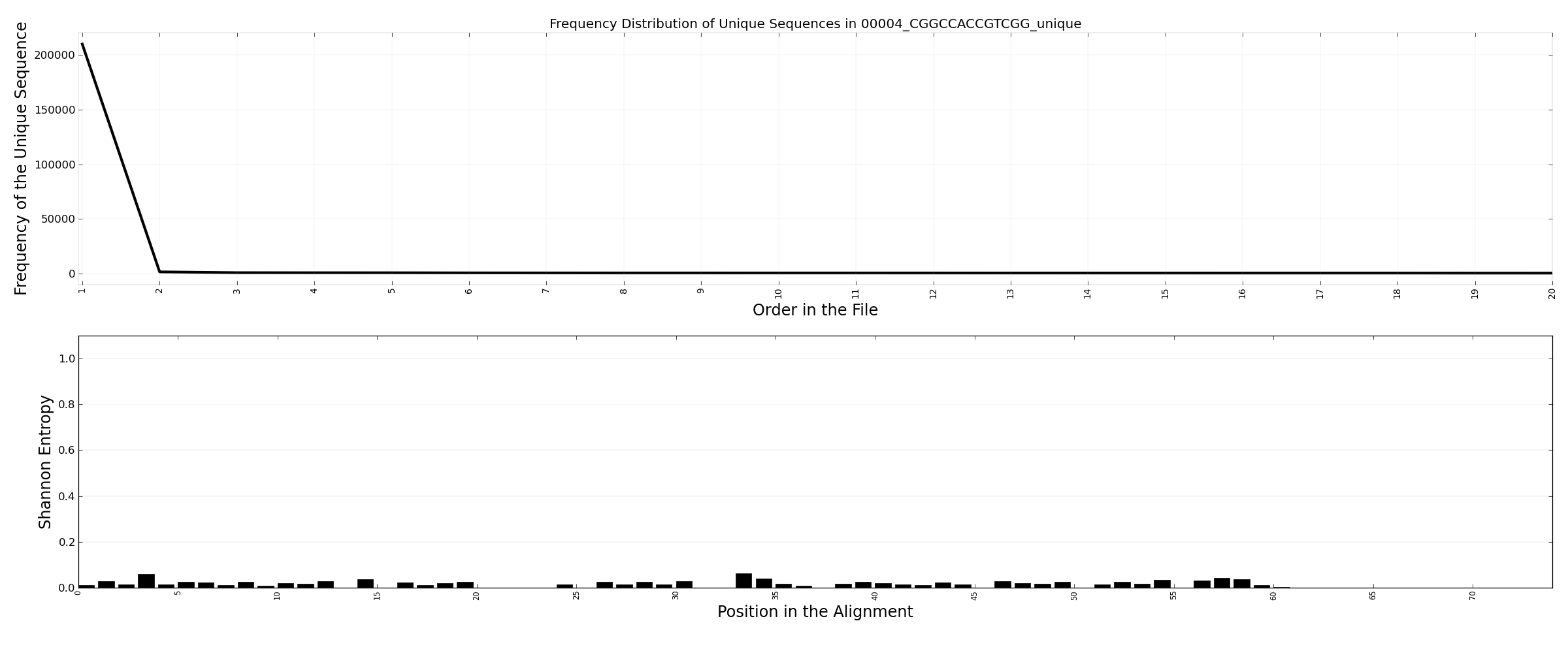

Supplement: Supplementary file 6 [file DataSheet2.ZIP › HTML-OUTPUT/00004_CGGCCACCGTCGG_unique.png]

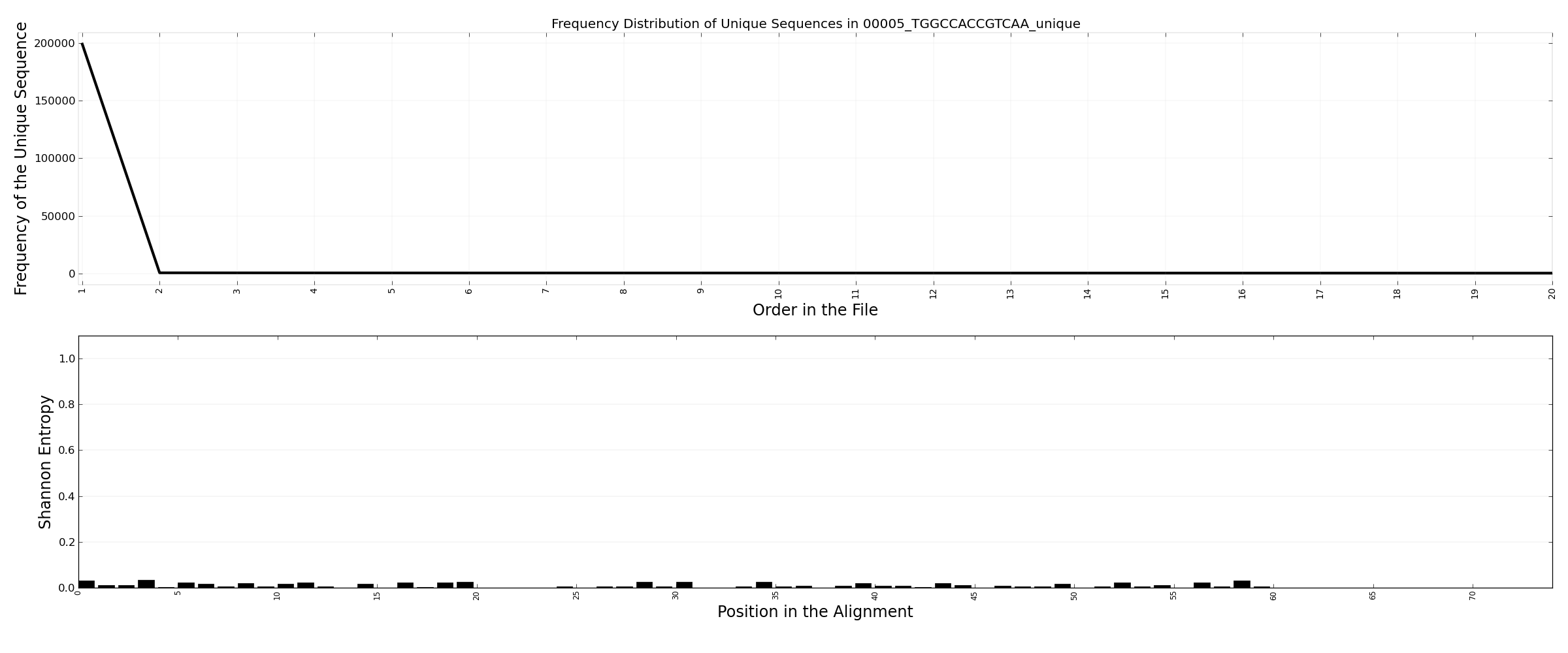

Supplement: Supplementary file 6 [file DataSheet2.ZIP › HTML-OUTPUT/00005_TGGCCACCGTCAA_unique.png]

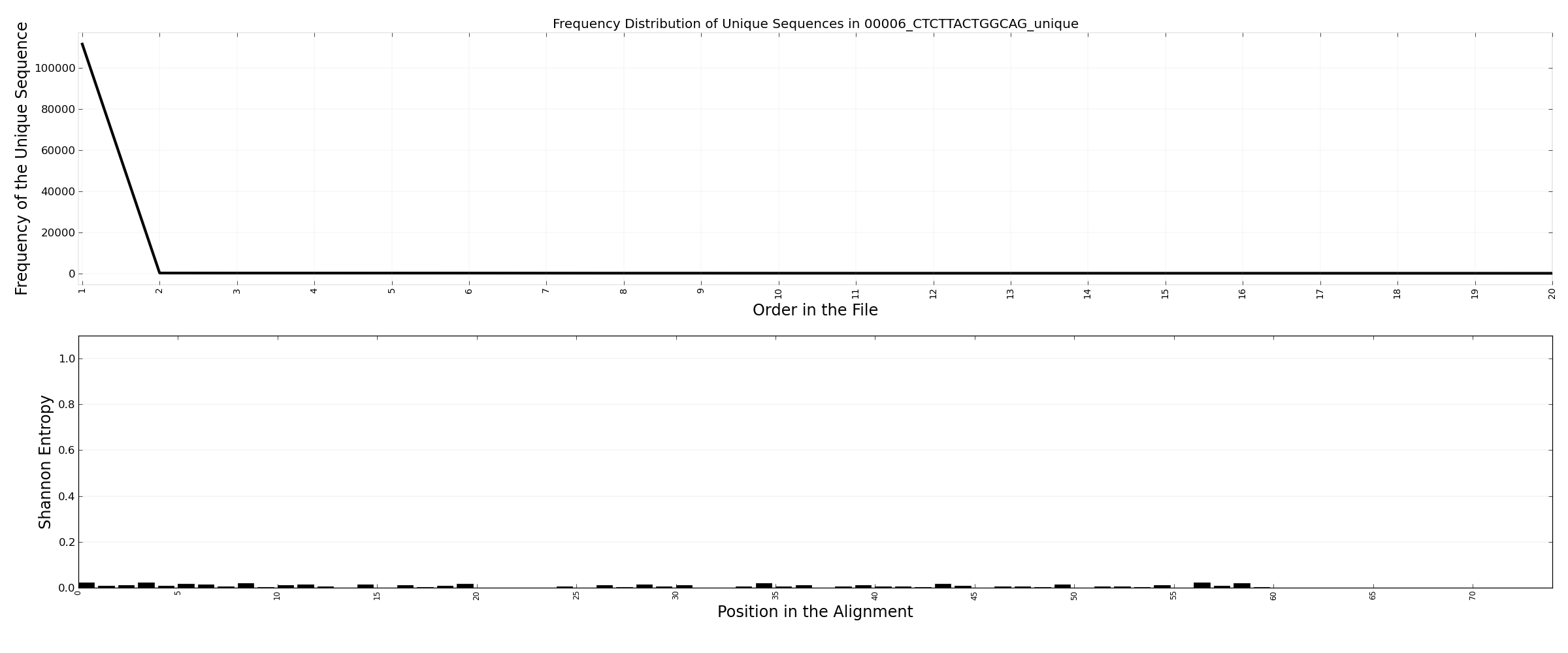

Supplement: Supplementary file 6 [file DataSheet2.ZIP › HTML-OUTPUT/00006_CTCTTACTGGCAG_unique.png]

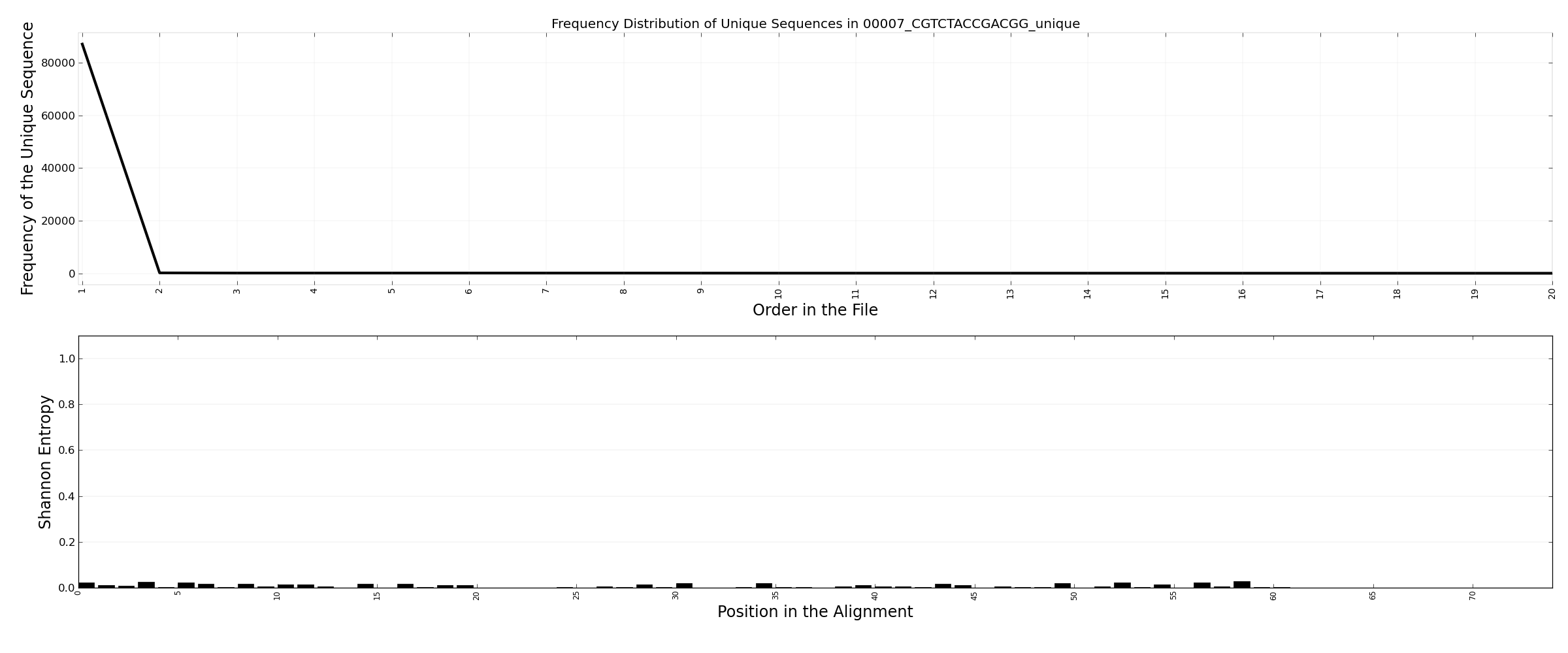

Supplement: Supplementary file 6 [file DataSheet2.ZIP › HTML-OUTPUT/00007_CGTCTACCGACGG_unique.png]

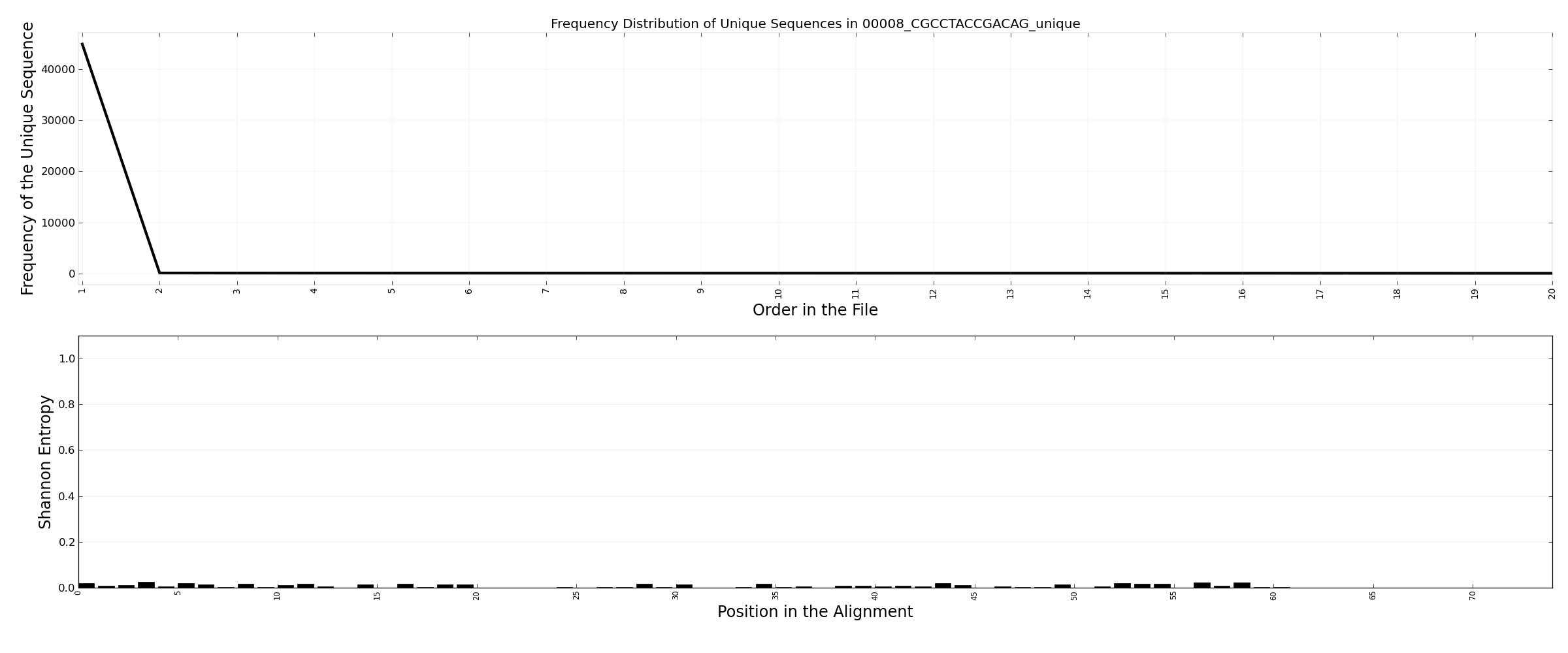

Supplement: Supplementary file 6 [file DataSheet2.ZIP › HTML-OUTPUT/00008_CGCCTACCGACAG_unique.png]

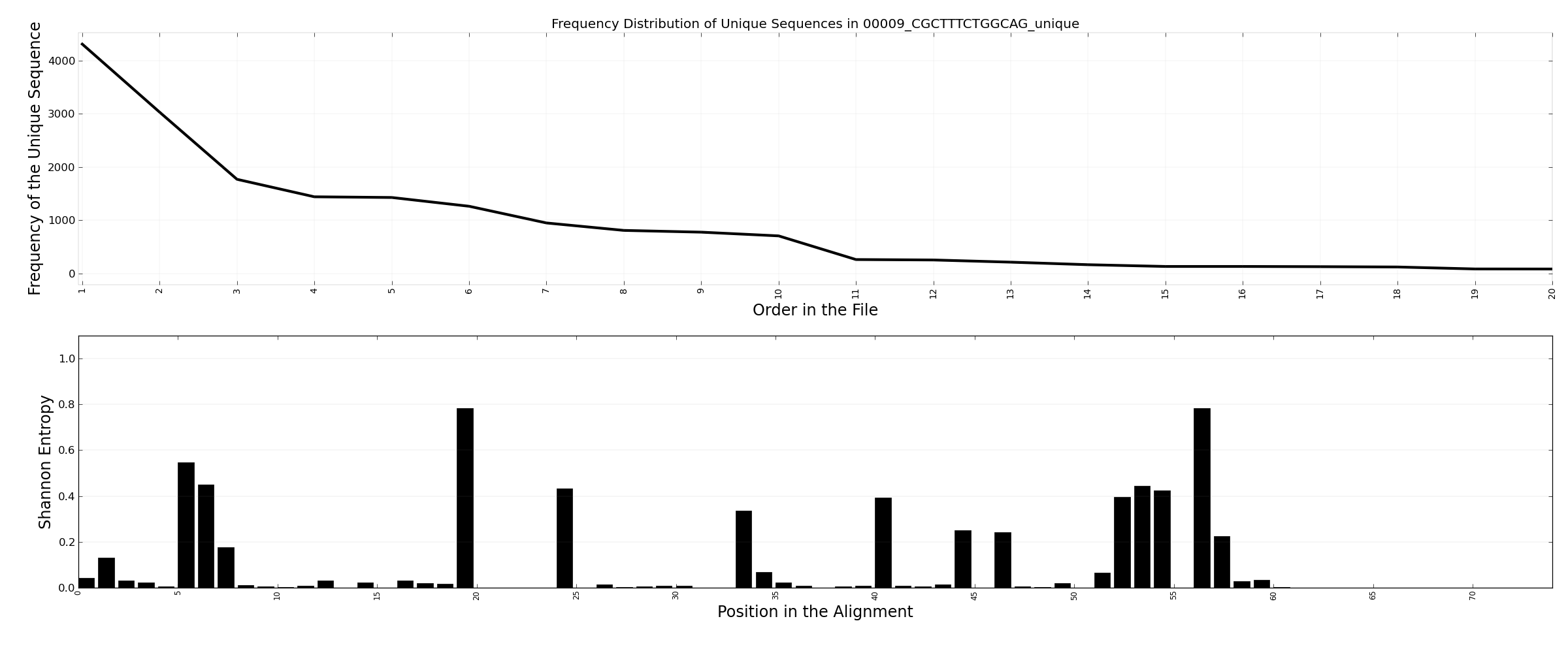

Supplement: Supplementary file 6 [file DataSheet2.ZIP › HTML-OUTPUT/00009_CGCTTTCTGGCAG_unique.png]

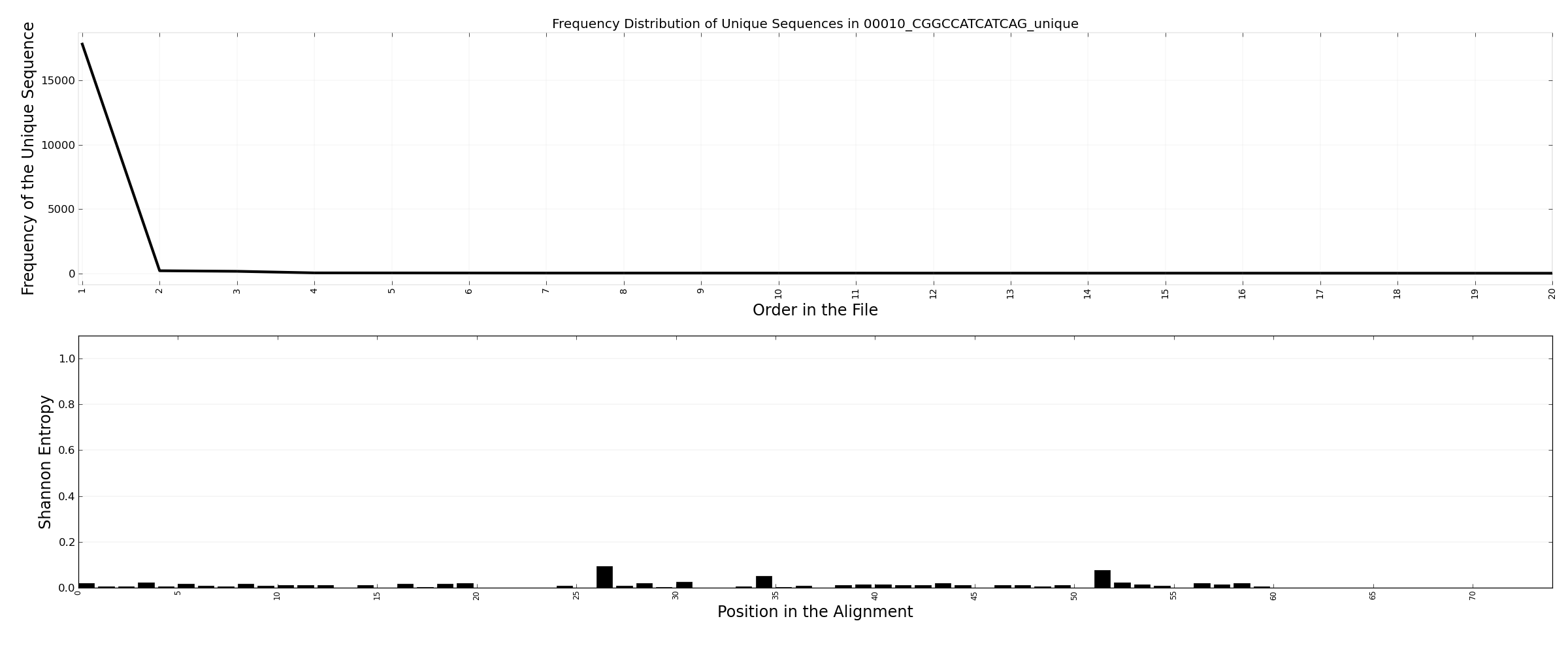

Supplement: Supplementary file 6 [file DataSheet2.ZIP › HTML-OUTPUT/00010_CGGCCATCATCAG_unique.png]

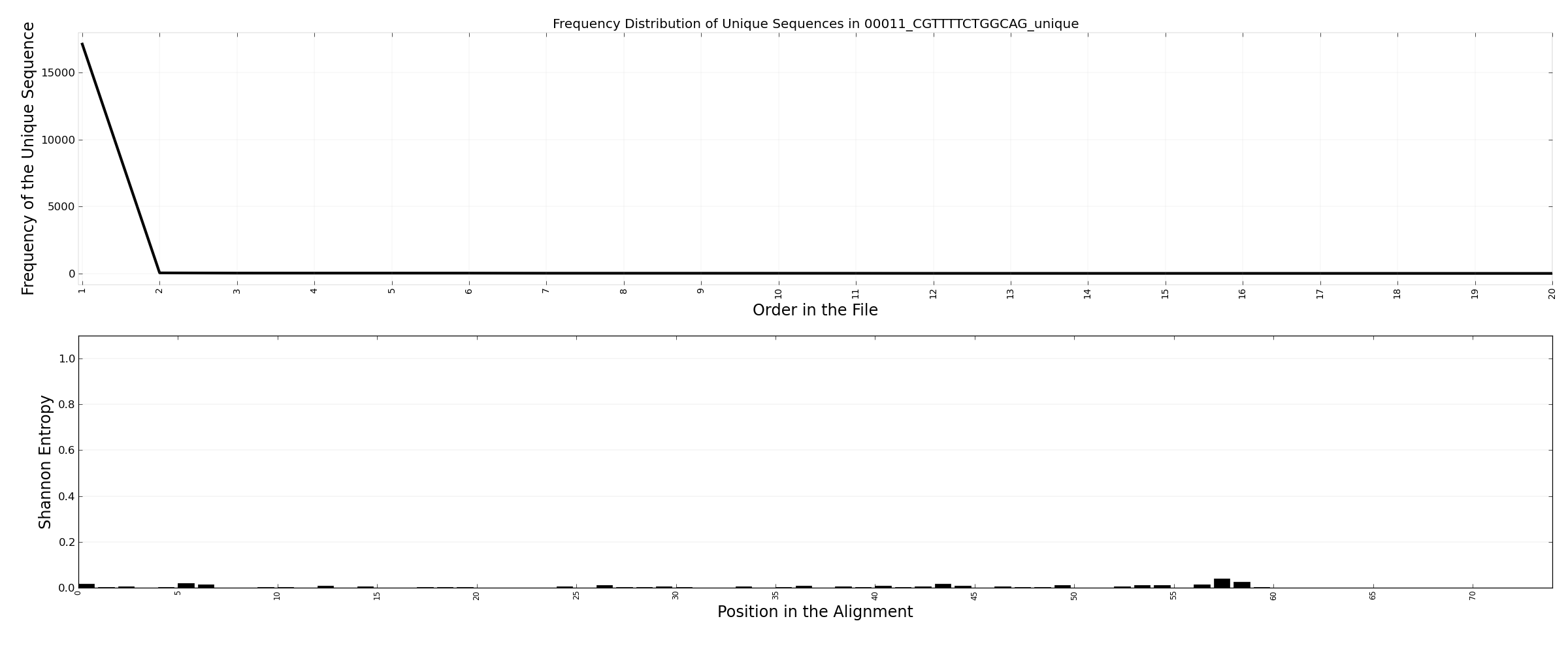

Supplement: Supplementary file 6 [file DataSheet2.ZIP › HTML-OUTPUT/00011_CGTTTTCTGGCAG_unique.png]

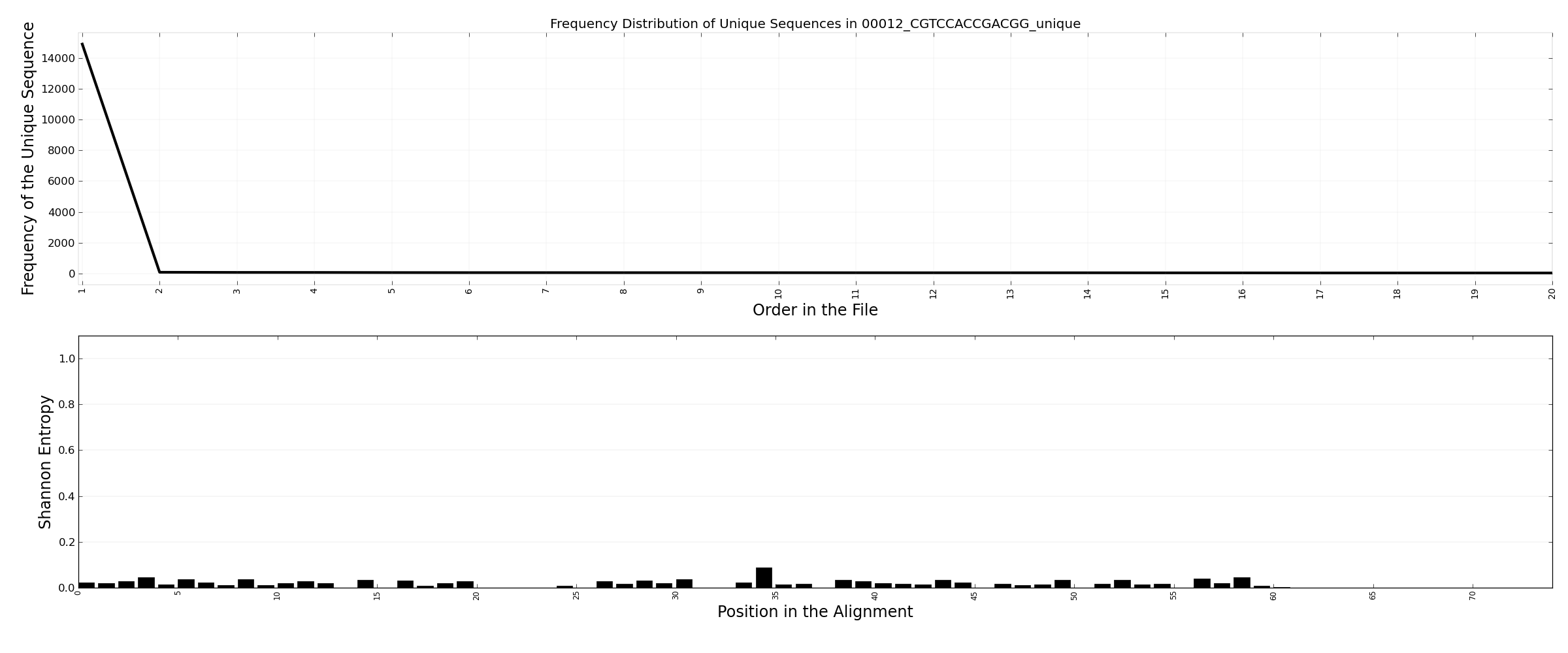

Supplement: Supplementary file 6 [file DataSheet2.ZIP › HTML-OUTPUT/00012_CGTCCACCGACGG_unique.png]

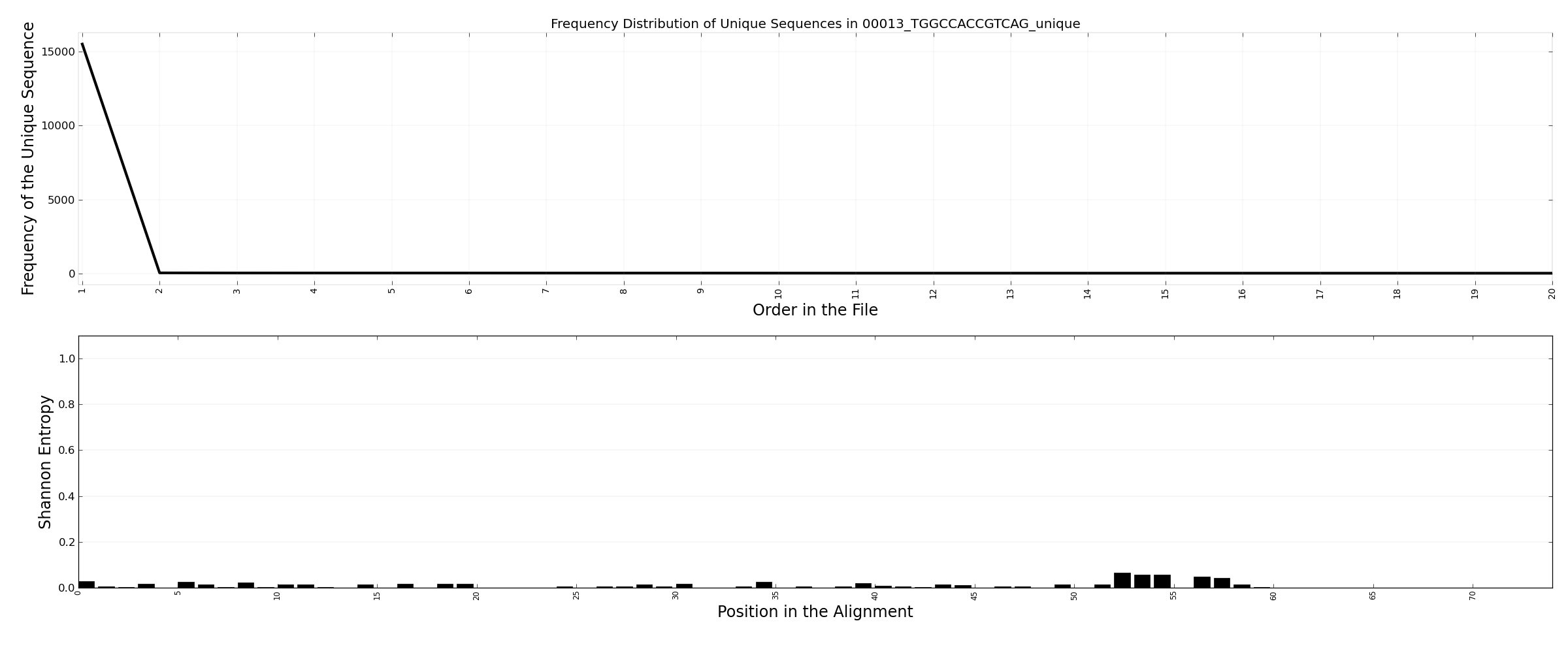

Supplement: Supplementary file 6 [file DataSheet2.ZIP › HTML-OUTPUT/00013_TGGCCACCGTCAG_unique.png]

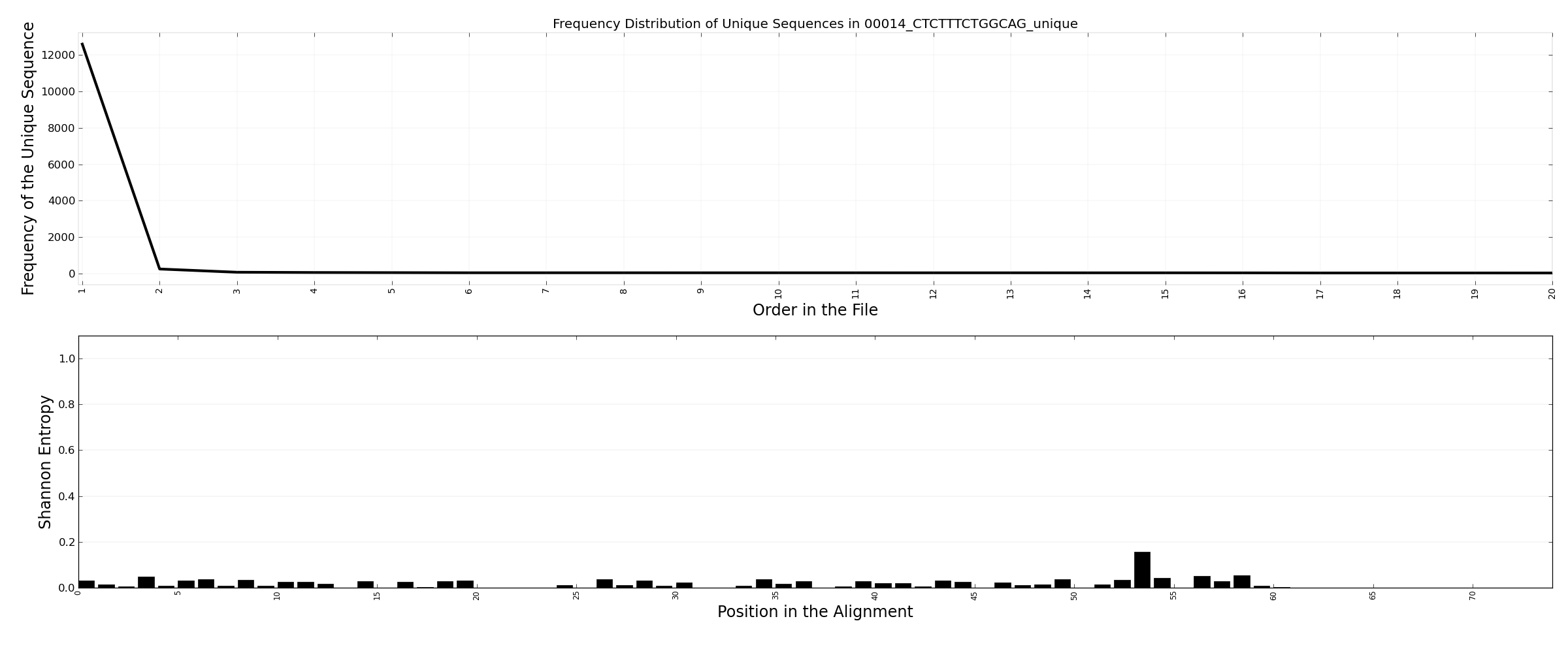

Supplement: Supplementary file 6 [file DataSheet2.ZIP › HTML-OUTPUT/00014_CTCTTTCTGGCAG_unique.png]

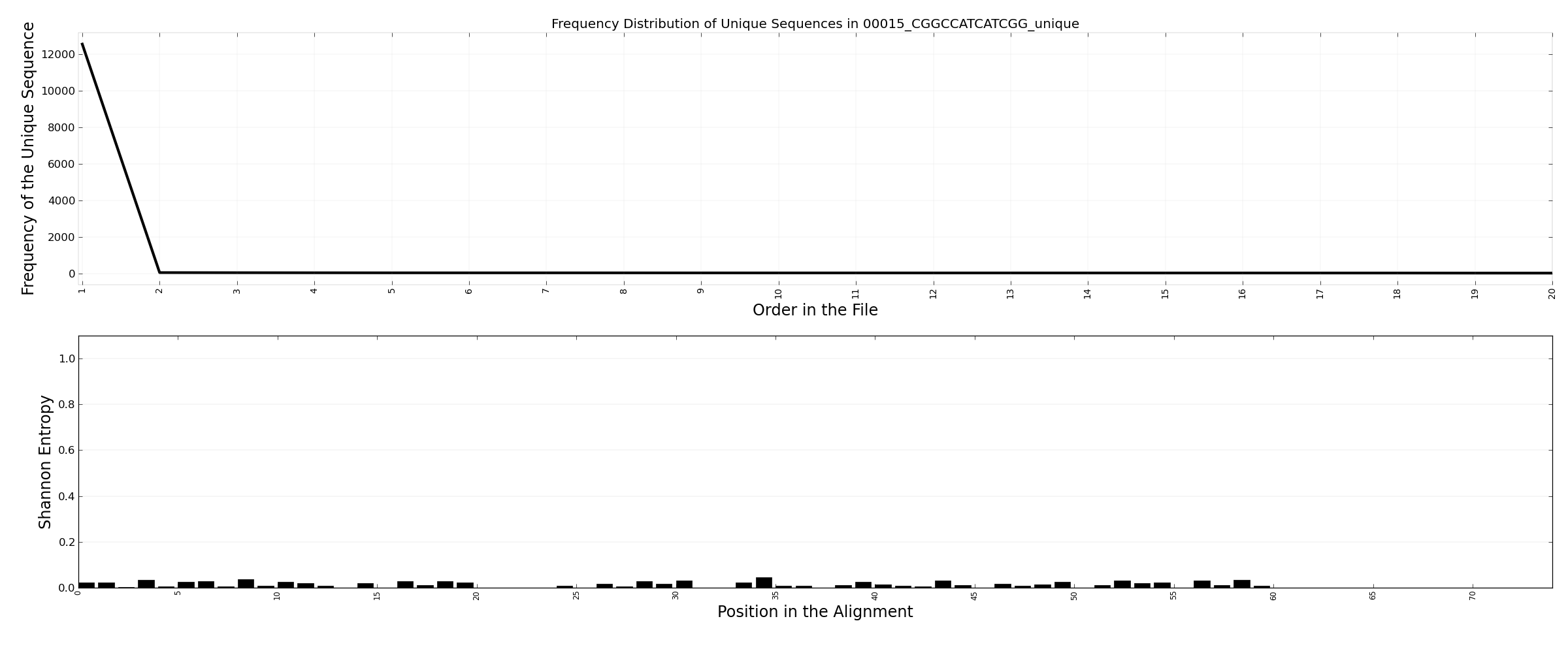

Supplement: Supplementary file 6 [file DataSheet2.ZIP › HTML-OUTPUT/00015_CGGCCATCATCGG_unique.png]

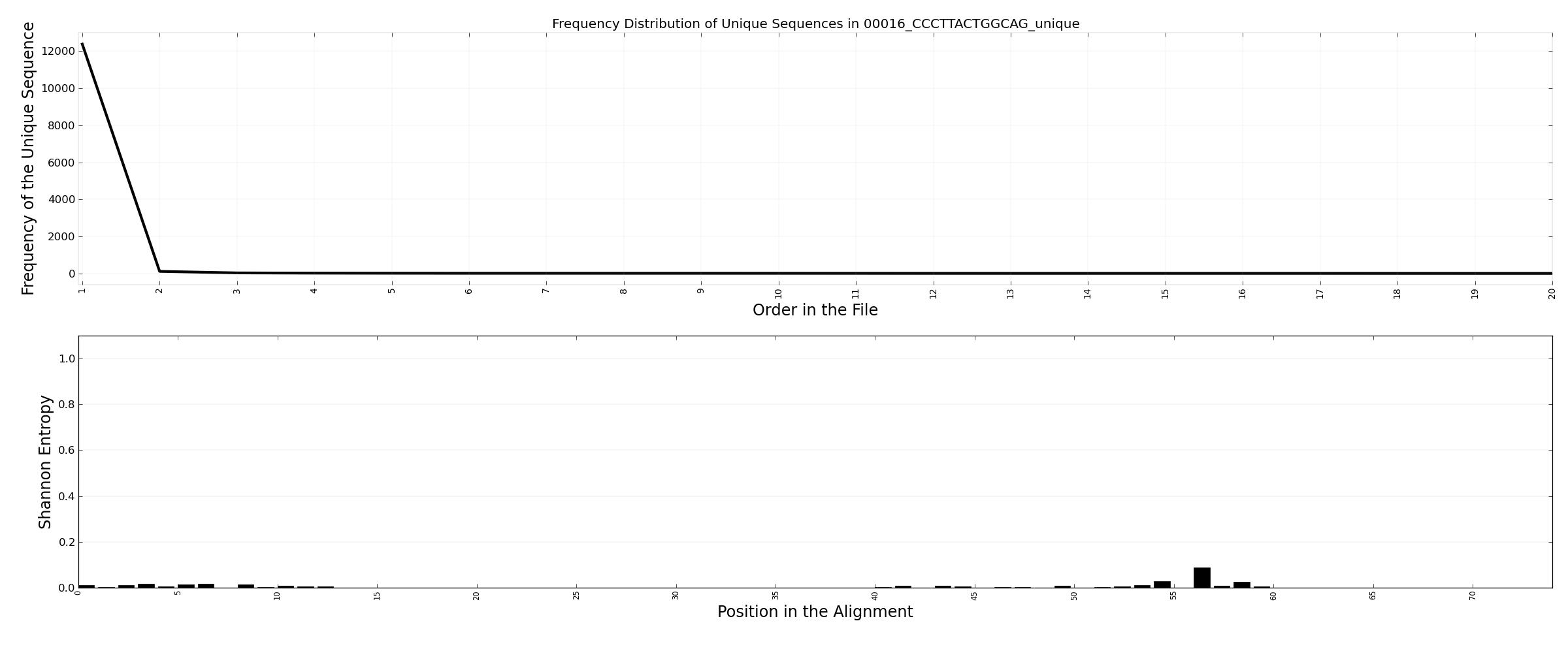

Supplement: Supplementary file 6 [file DataSheet2.ZIP › HTML-OUTPUT/00016_CCCTTACTGGCAG_unique.png]

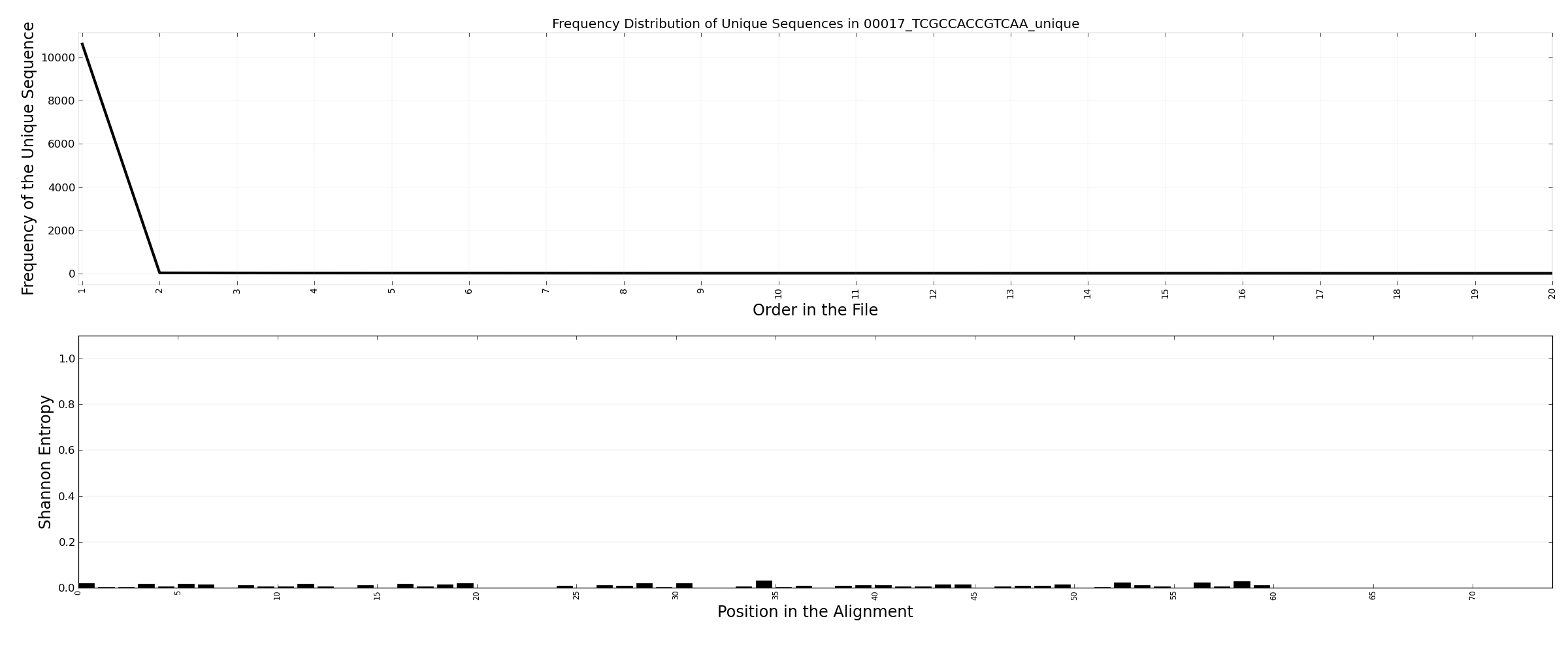

Supplement: Supplementary file 6 [file DataSheet2.ZIP › HTML-OUTPUT/00017_TCGCCACCGTCAA_unique.png]

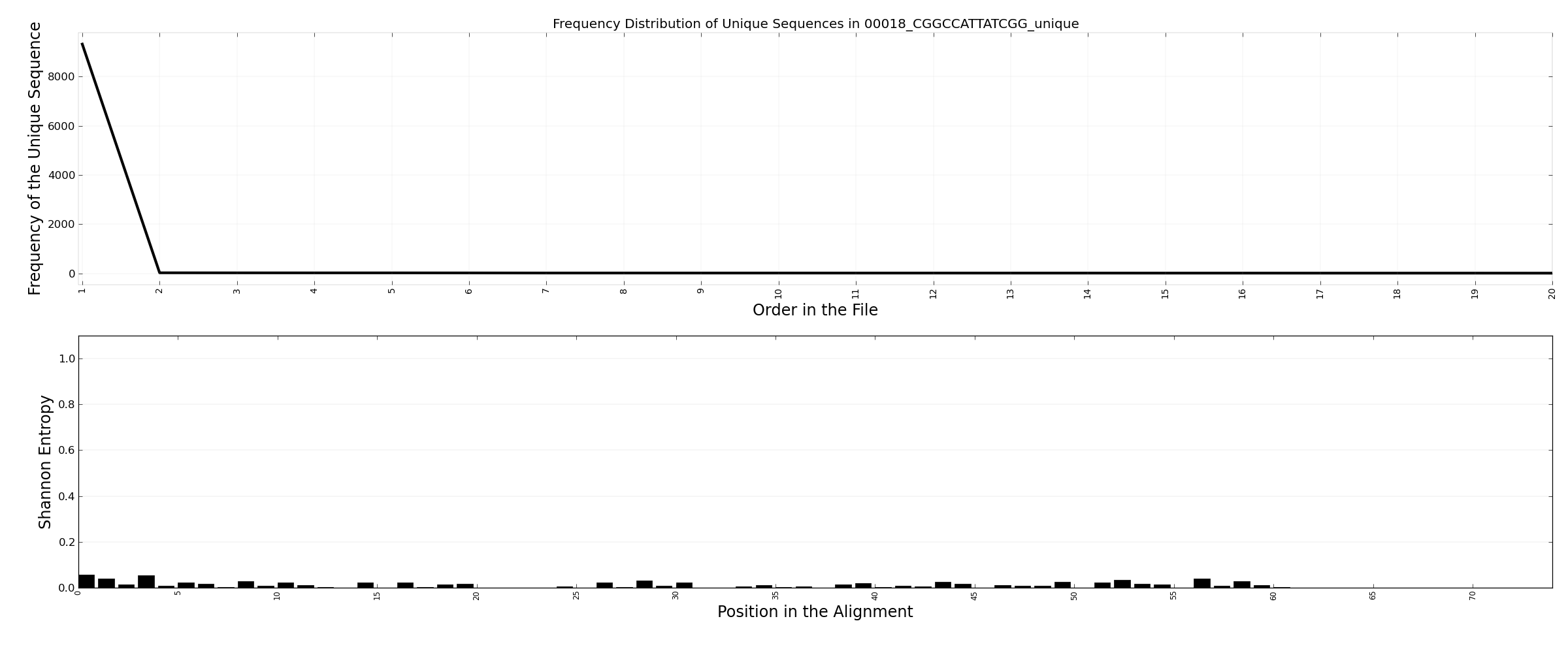

Supplement: Supplementary file 6 [file DataSheet2.ZIP › HTML-OUTPUT/00018_CGGCCATTATCGG_unique.png]

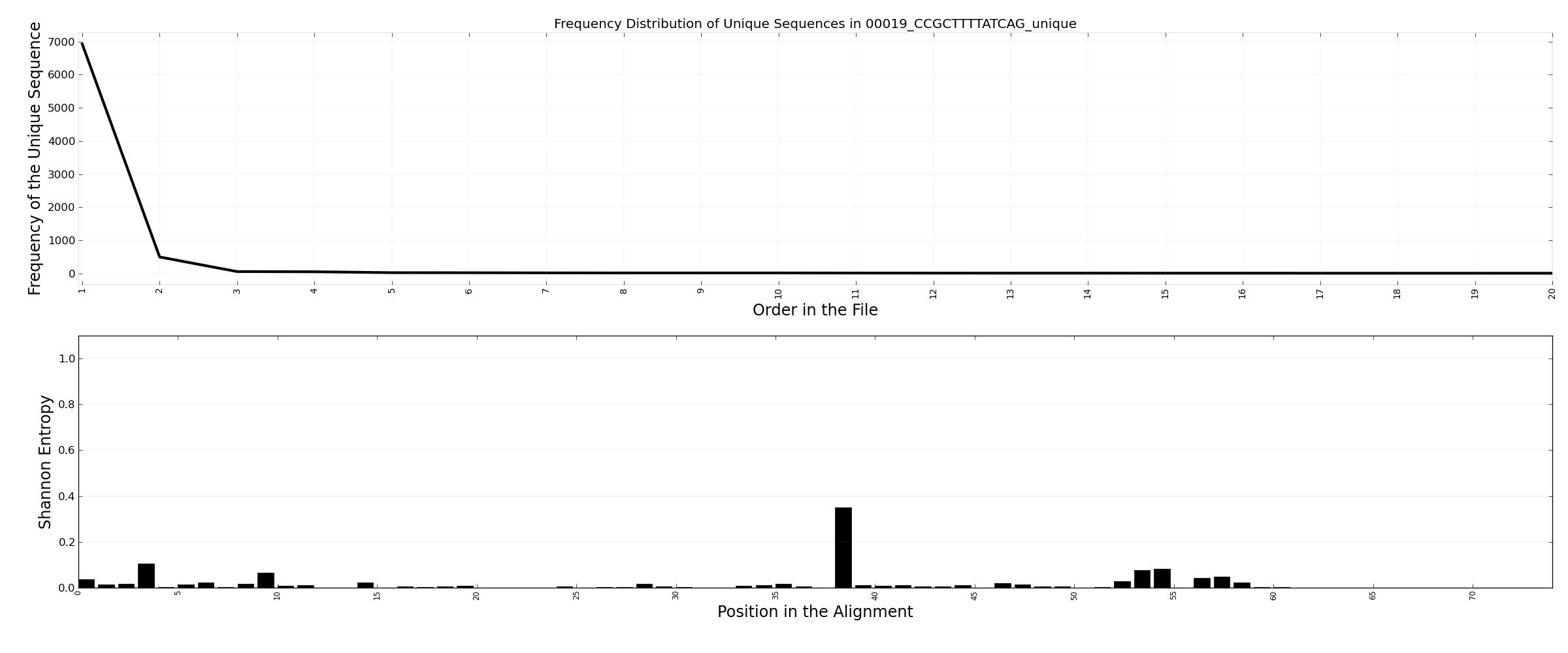

Supplement: Supplementary file 6 [file DataSheet2.ZIP › HTML-OUTPUT/00019_CCGCTTTTATCAG_unique.png]

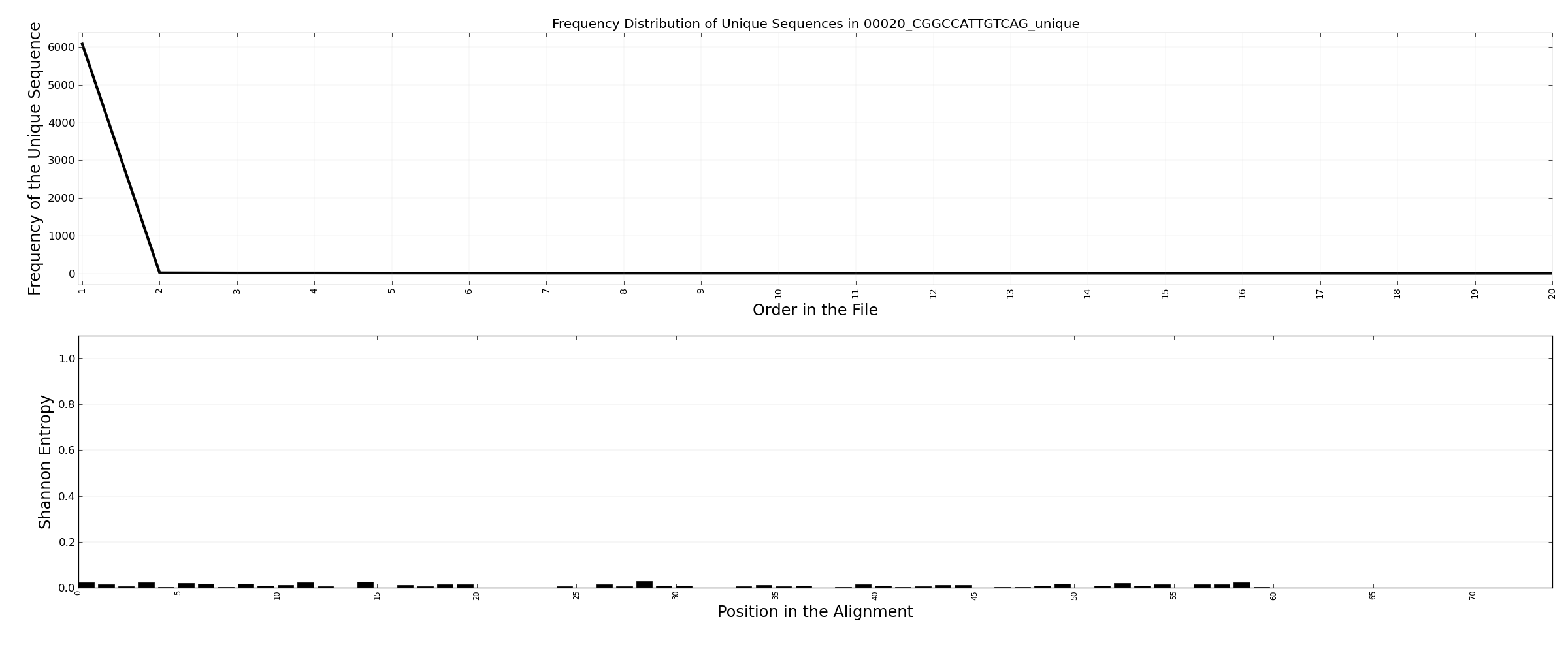

Supplement: Supplementary file 6 [file DataSheet2.ZIP › HTML-OUTPUT/00020_CGGCCATTGTCAG_unique.png]

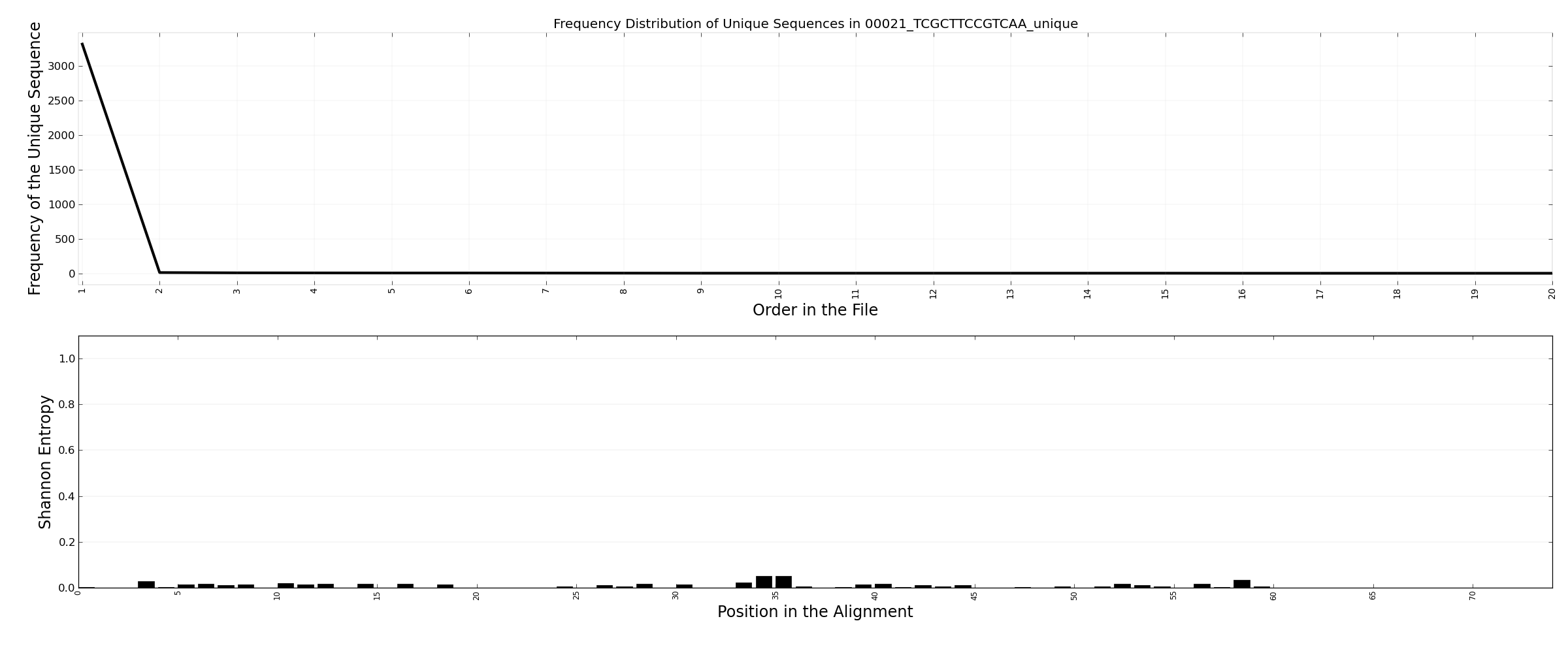

Supplement: Supplementary file 6 [file DataSheet2.ZIP › HTML-OUTPUT/00021_TCGCTTCCGTCAA_unique.png]

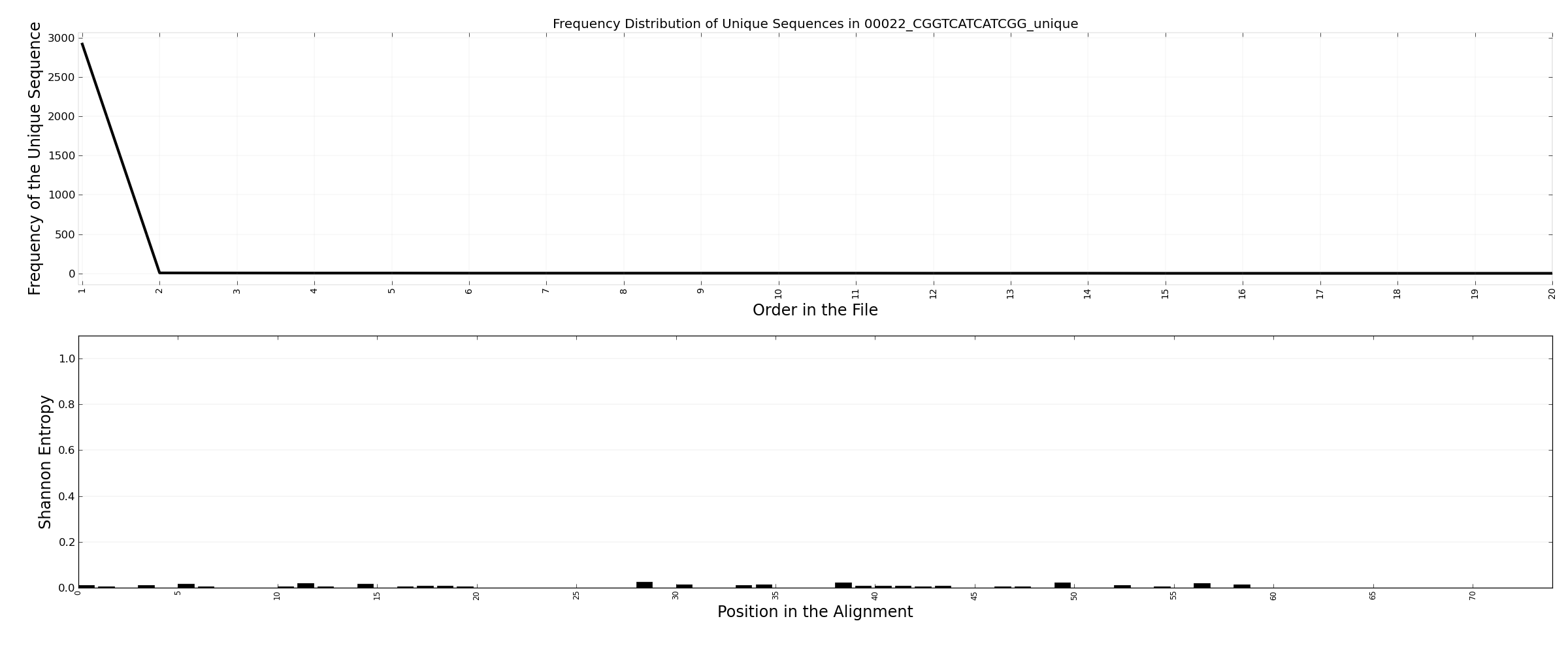

Supplement: Supplementary file 6 [file DataSheet2.ZIP › HTML-OUTPUT/00022_CGGTCATCATCGG_unique.png]

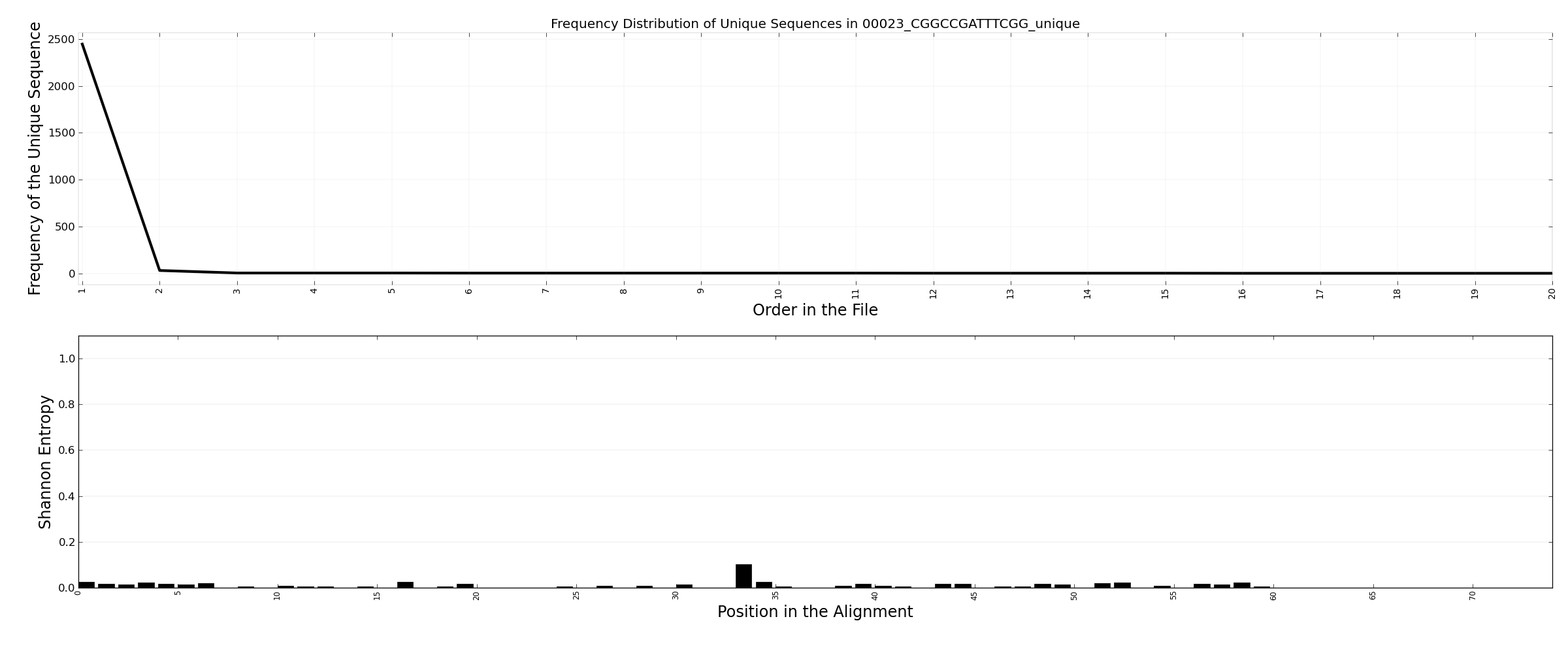

Supplement: Supplementary file 6 [file DataSheet2.ZIP › HTML-OUTPUT/00023_CGGCCGATTTCGG_unique.png]

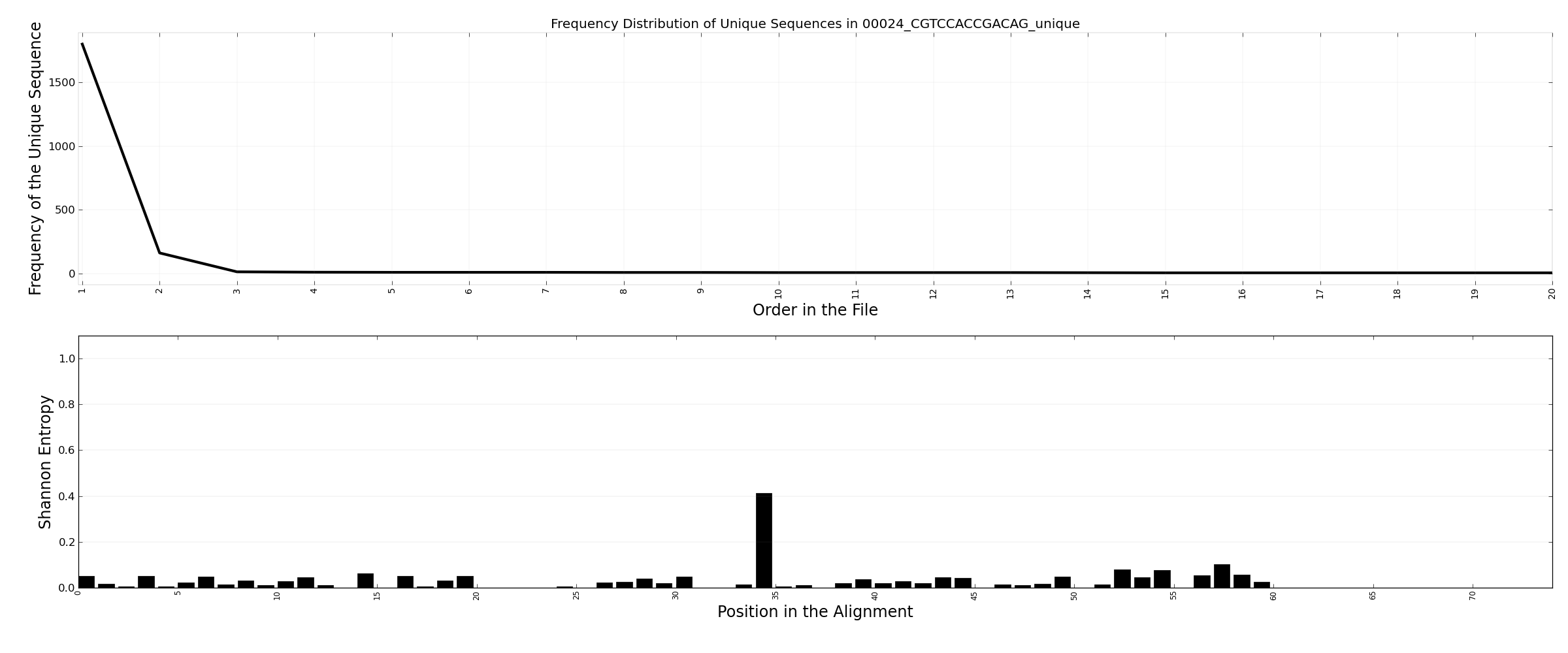

Supplement: Supplementary file 6 [file DataSheet2.ZIP › HTML-OUTPUT/00024_CGTCCACCGACAG_unique.png]

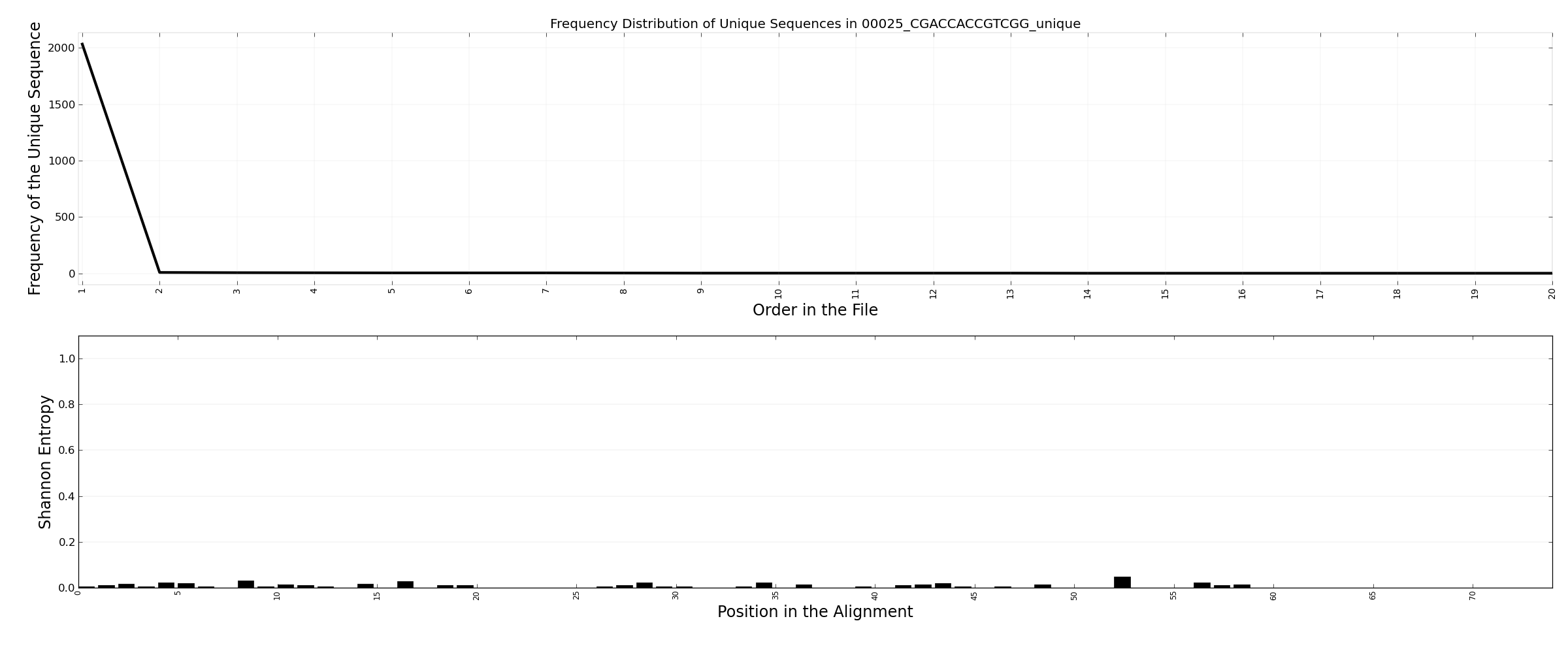

Supplement: Supplementary file 6 [file DataSheet2.ZIP › HTML-OUTPUT/00025_CGACCACCGTCGG_unique.png]

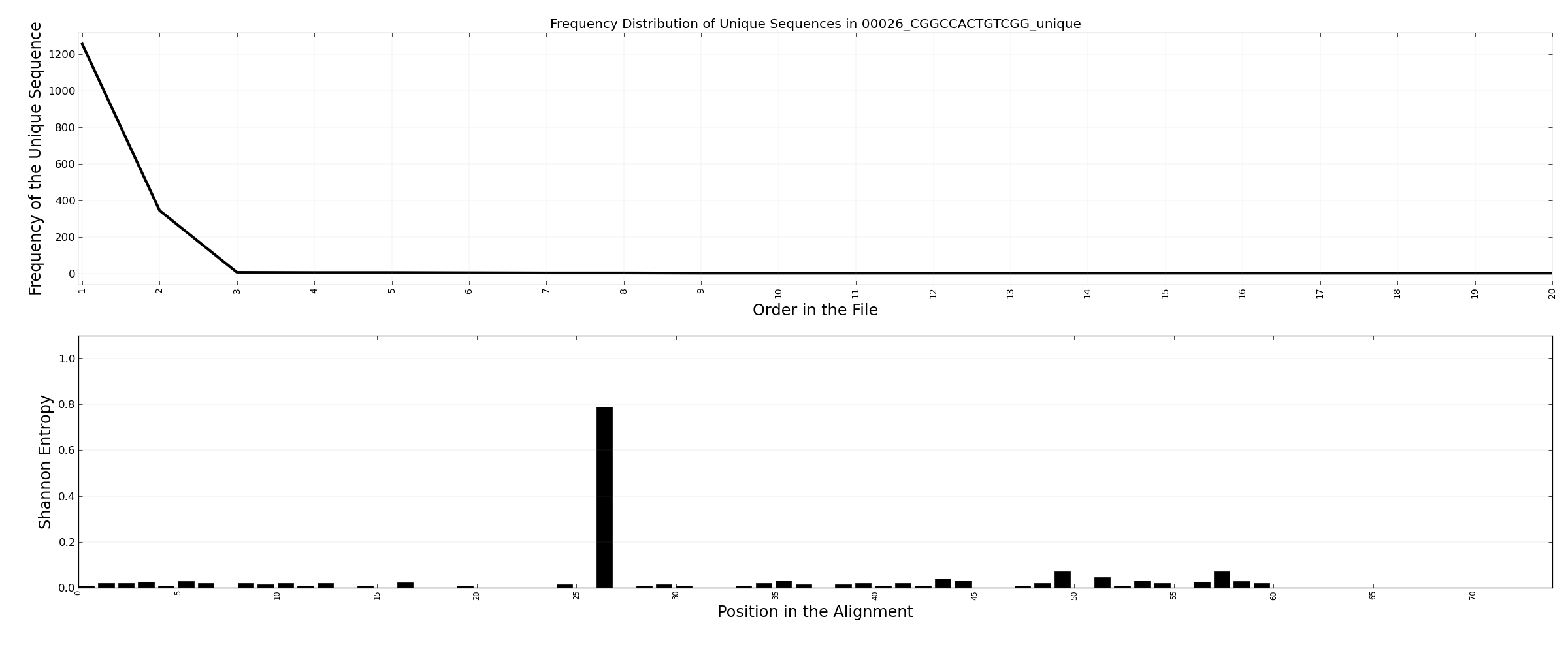

Supplement: Supplementary file 6 [file DataSheet2.ZIP › HTML-OUTPUT/00026_CGGCCACTGTCGG_unique.png]

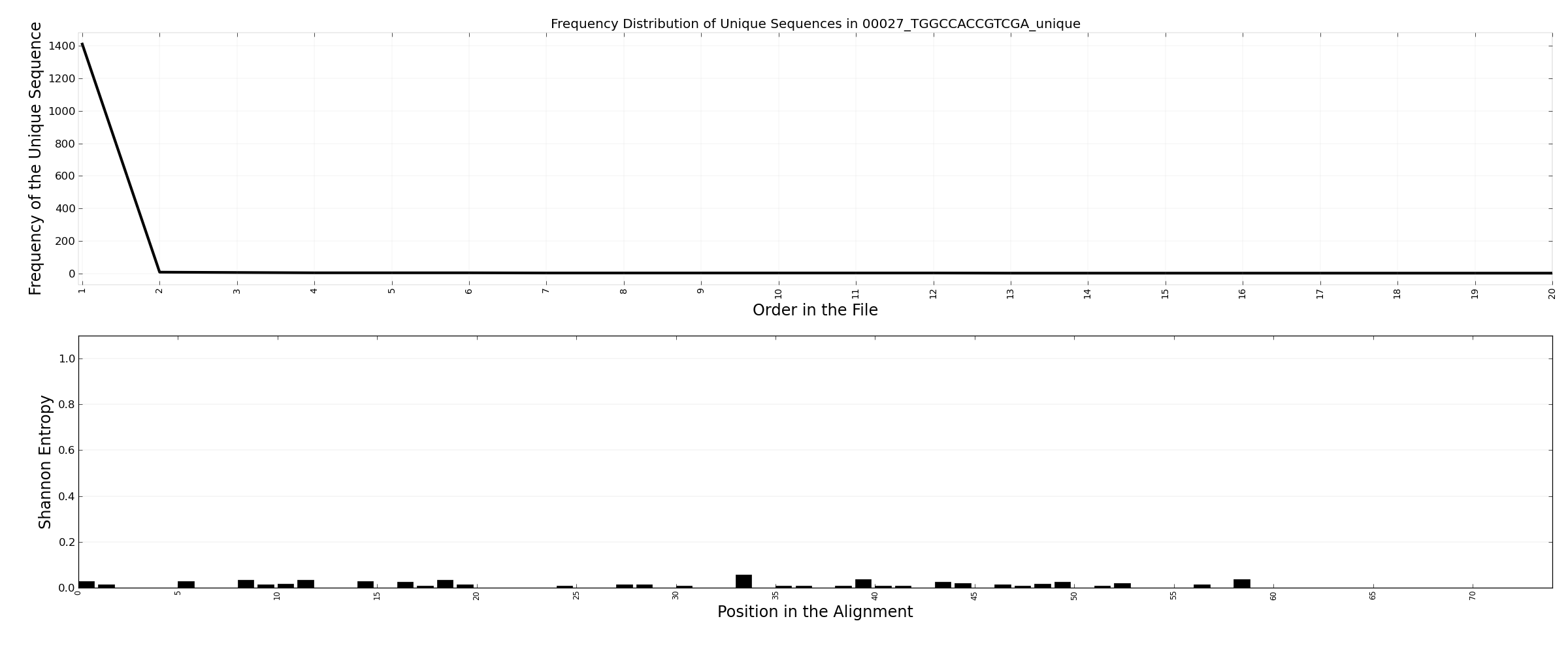

Supplement: Supplementary file 6 [file DataSheet2.ZIP › HTML-OUTPUT/00027_TGGCCACCGTCGA_unique.png]

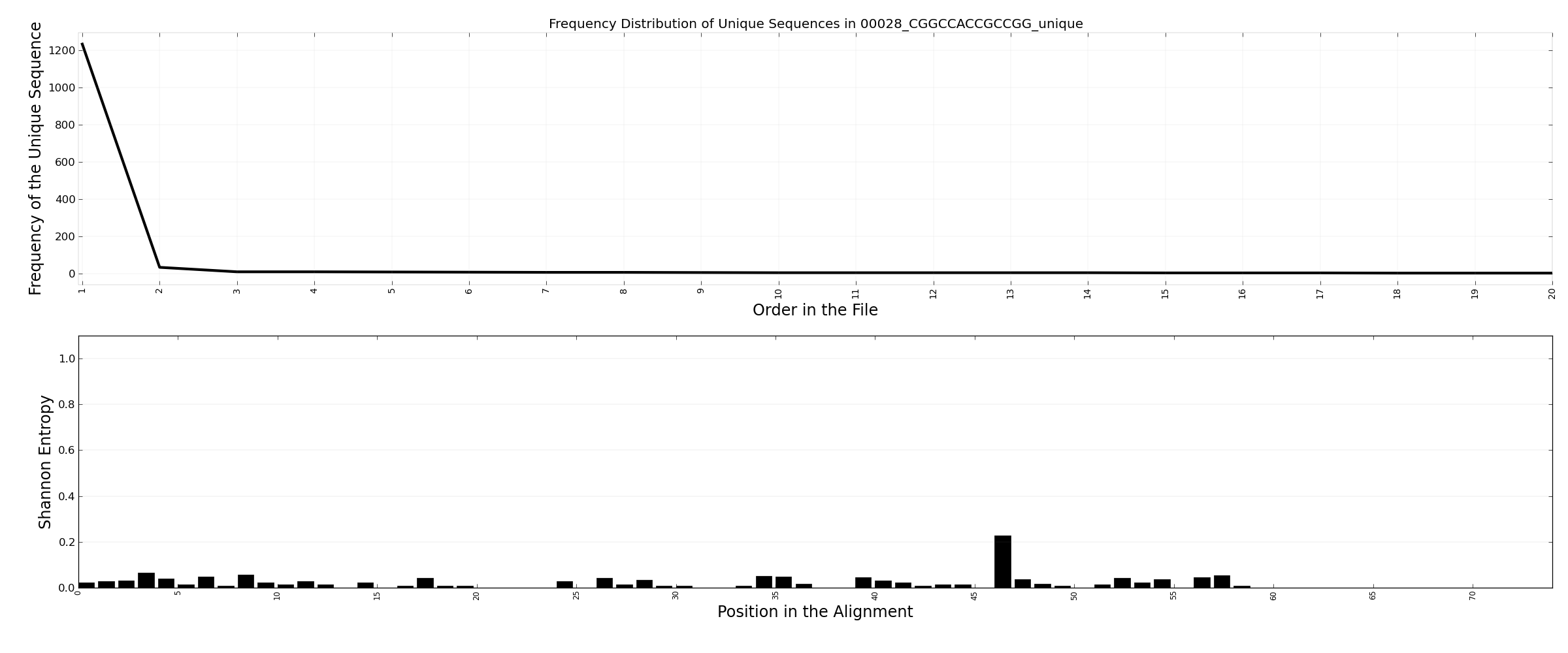

Supplement: Supplementary file 6 [file DataSheet2.ZIP › HTML-OUTPUT/00028_CGGCCACCGCCGG_unique.png]

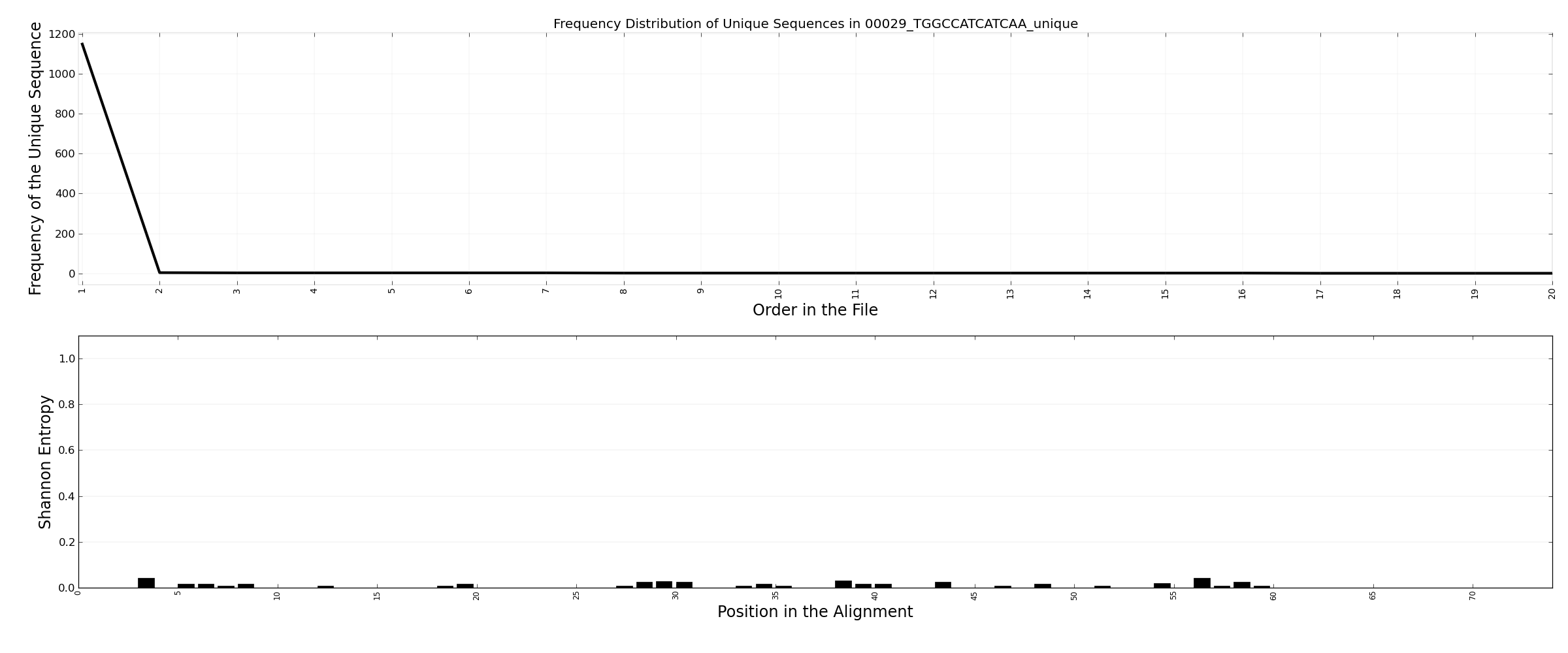

Supplement: Supplementary file 6 [file DataSheet2.ZIP › HTML-OUTPUT/00029_TGGCCATCATCAA_unique.png]

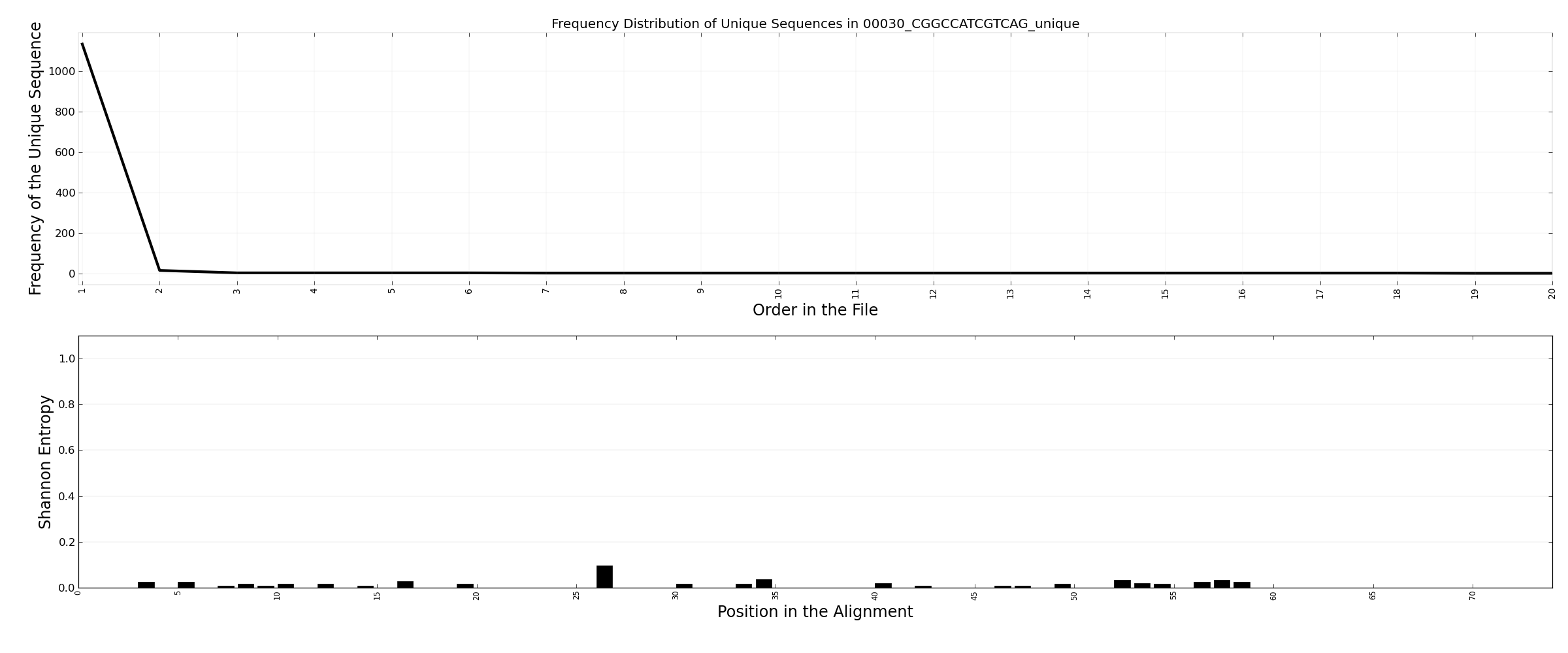

Supplement: Supplementary file 6 [file DataSheet2.ZIP › HTML-OUTPUT/00030_CGGCCATCGTCAG_unique.png]

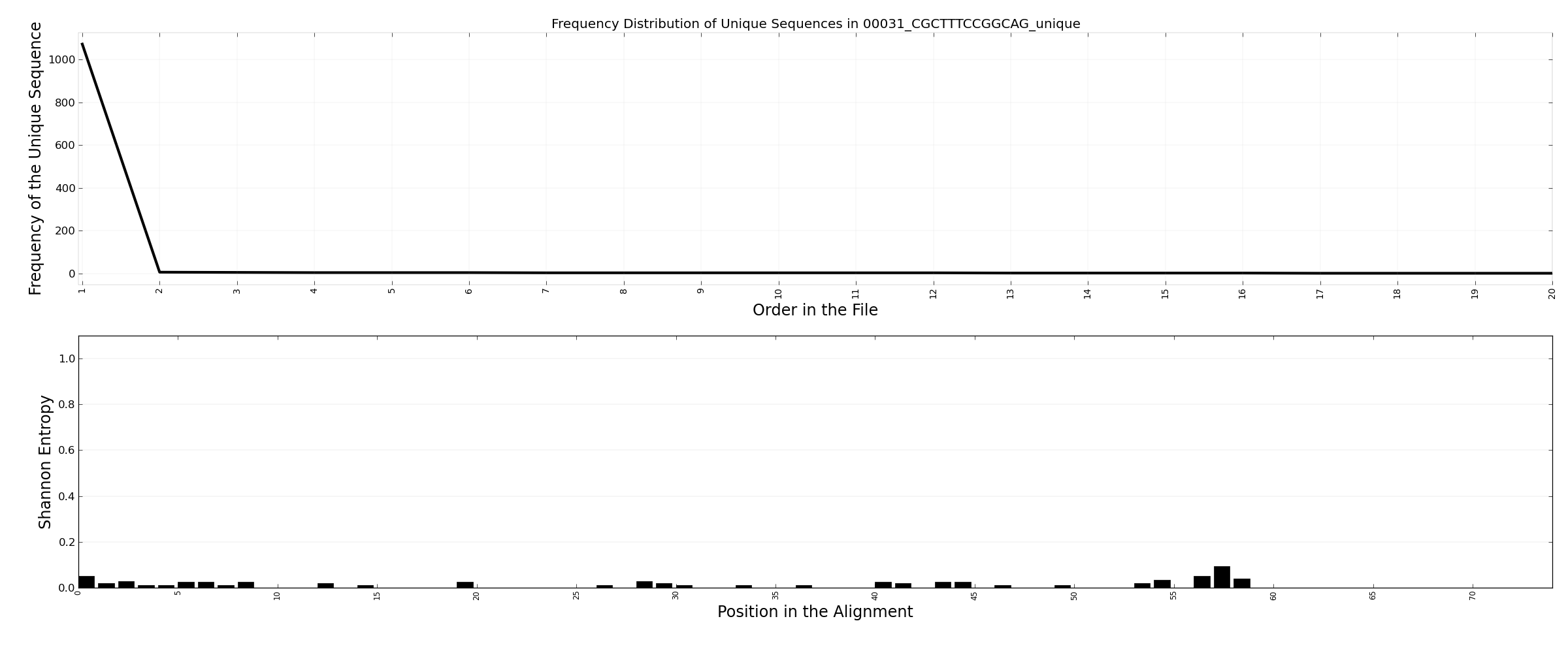

Supplement: Supplementary file 6 [file DataSheet2.ZIP › HTML-OUTPUT/00031_CGCTTTCCGGCAG_unique.png]

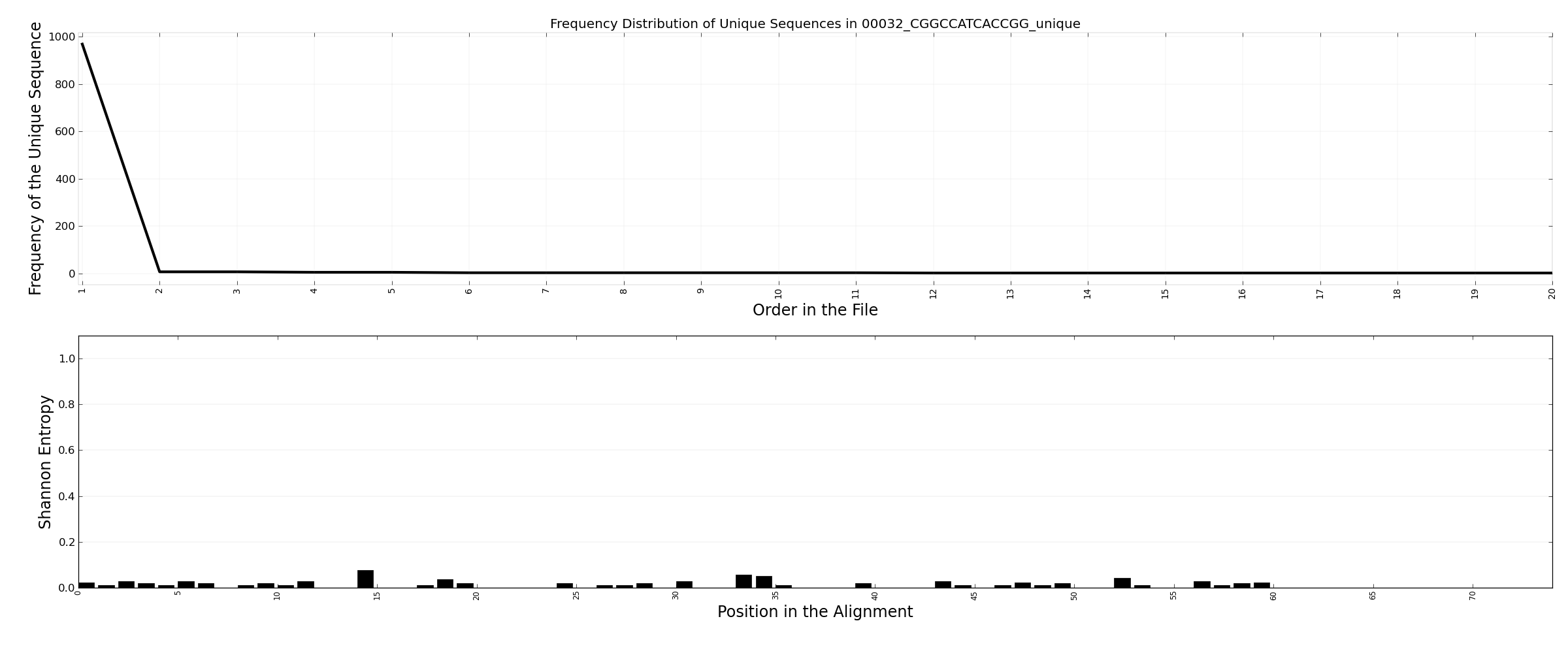

Supplement: Supplementary file 6 [file DataSheet2.ZIP › HTML-OUTPUT/00032_CGGCCATCACCGG_unique.png]

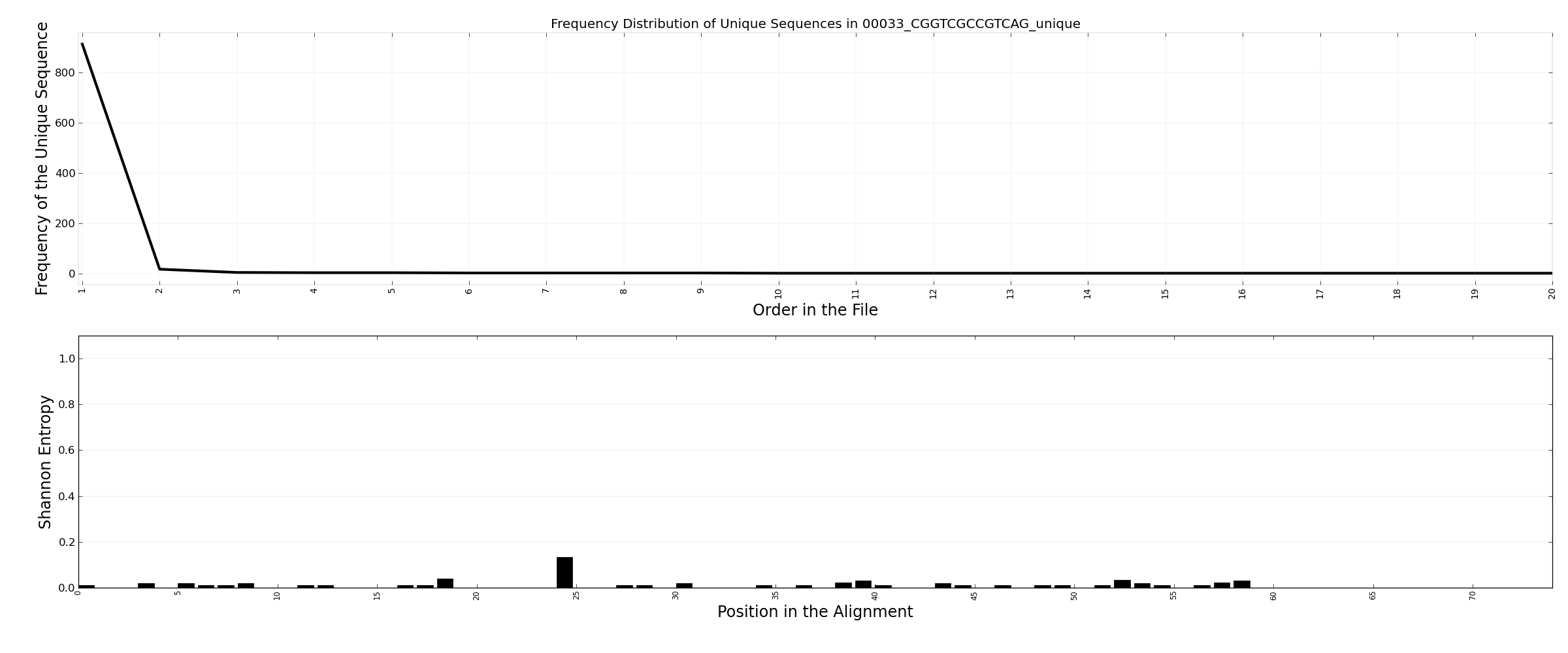

Supplement: Supplementary file 6 [file DataSheet2.ZIP › HTML-OUTPUT/00033_CGGTCGCCGTCAG_unique.png]

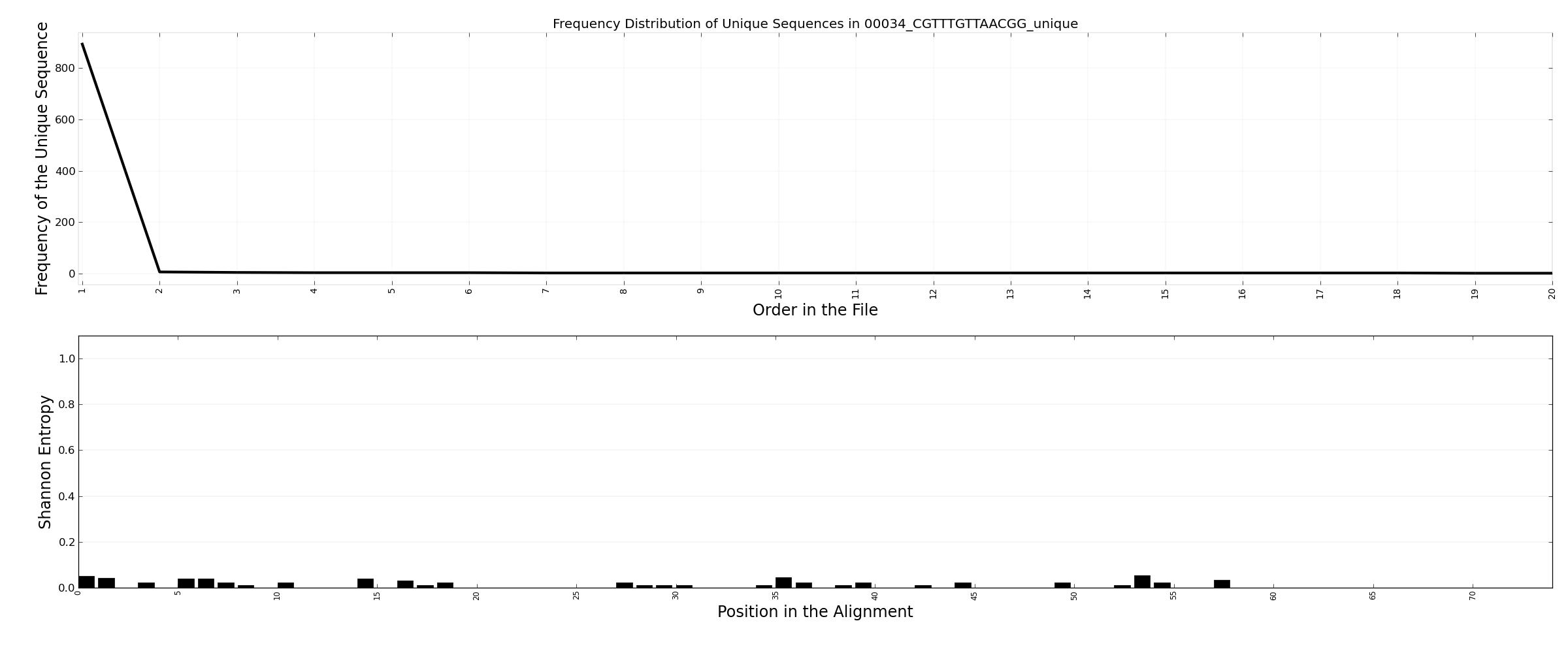

Supplement: Supplementary file 6 [file DataSheet2.ZIP › HTML-OUTPUT/00034_CGTTTGTTAACGG_unique.png]

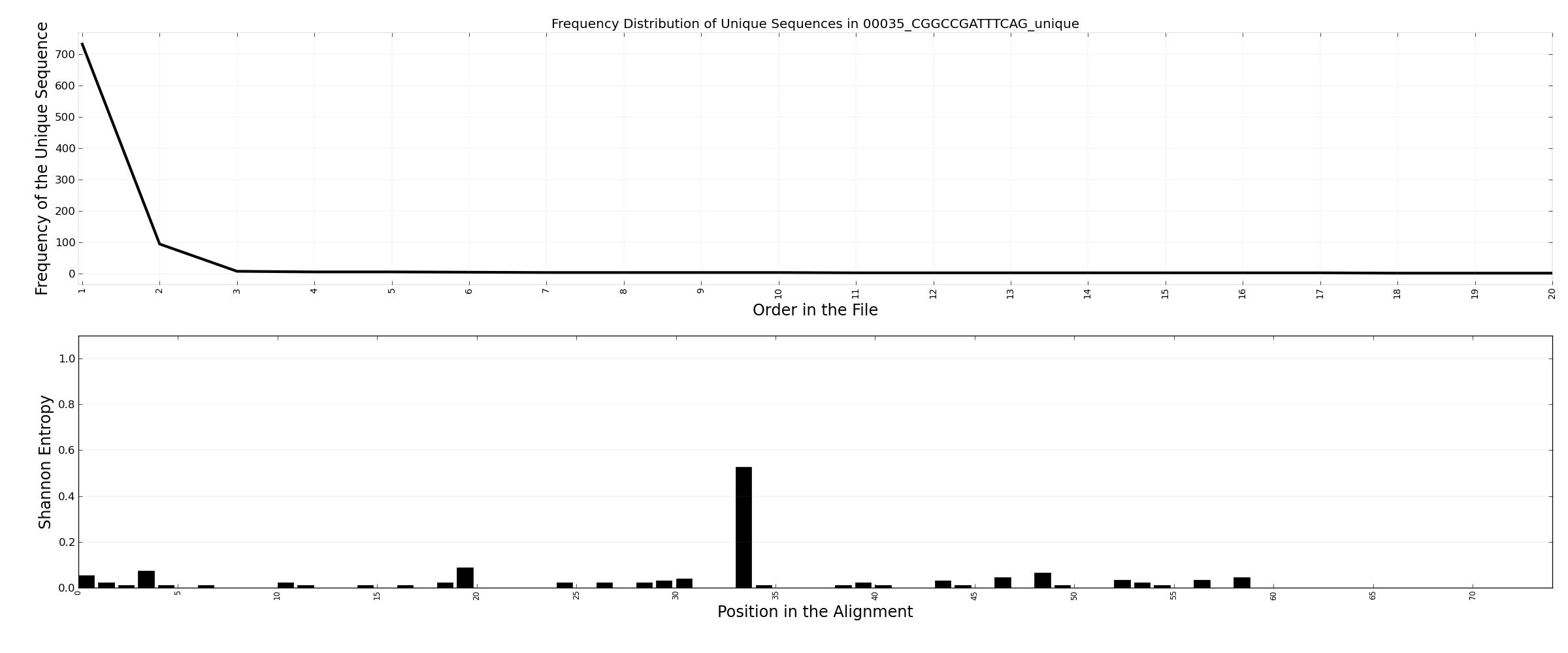

Supplement: Supplementary file 6 [file DataSheet2.ZIP › HTML-OUTPUT/00035_CGGCCGATTTCAG_unique.png]

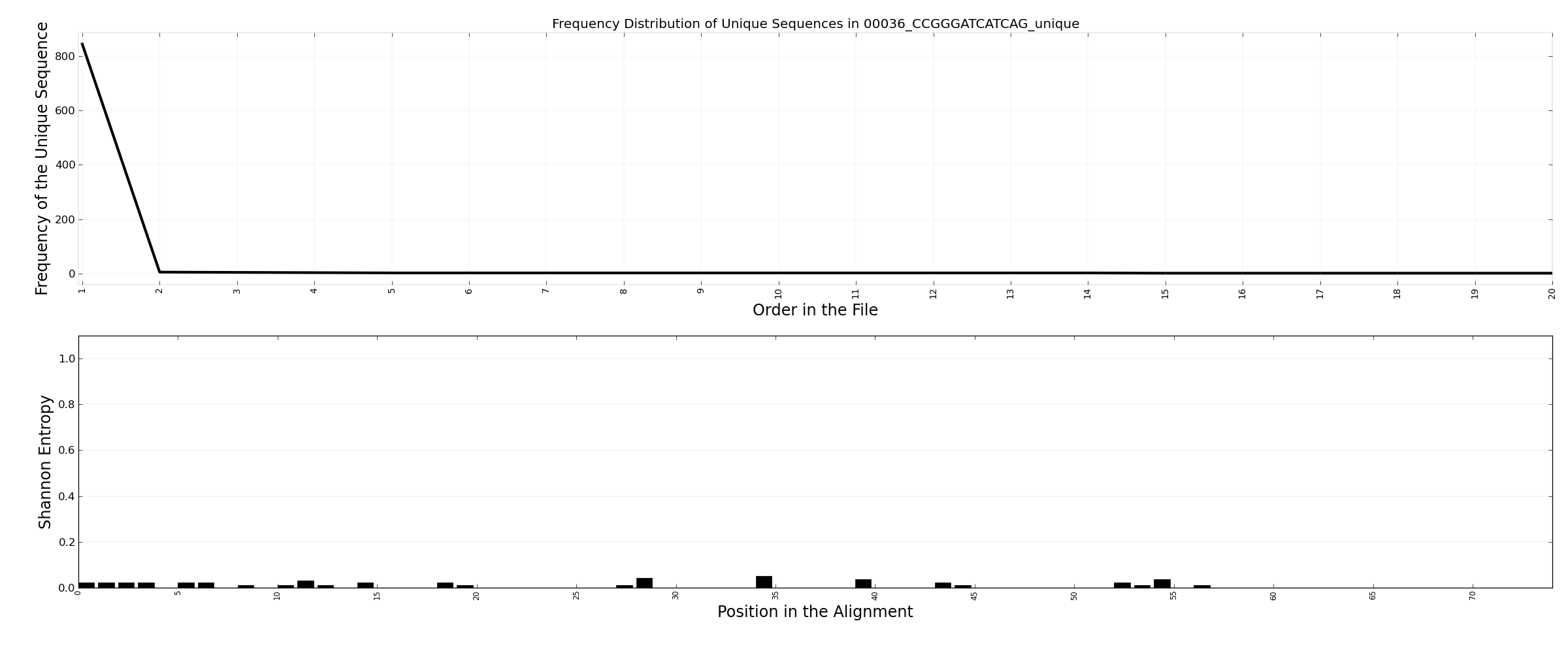

Supplement: Supplementary file 6 [file DataSheet2.ZIP › HTML-OUTPUT/00036_CCGGGATCATCAG_unique.png]

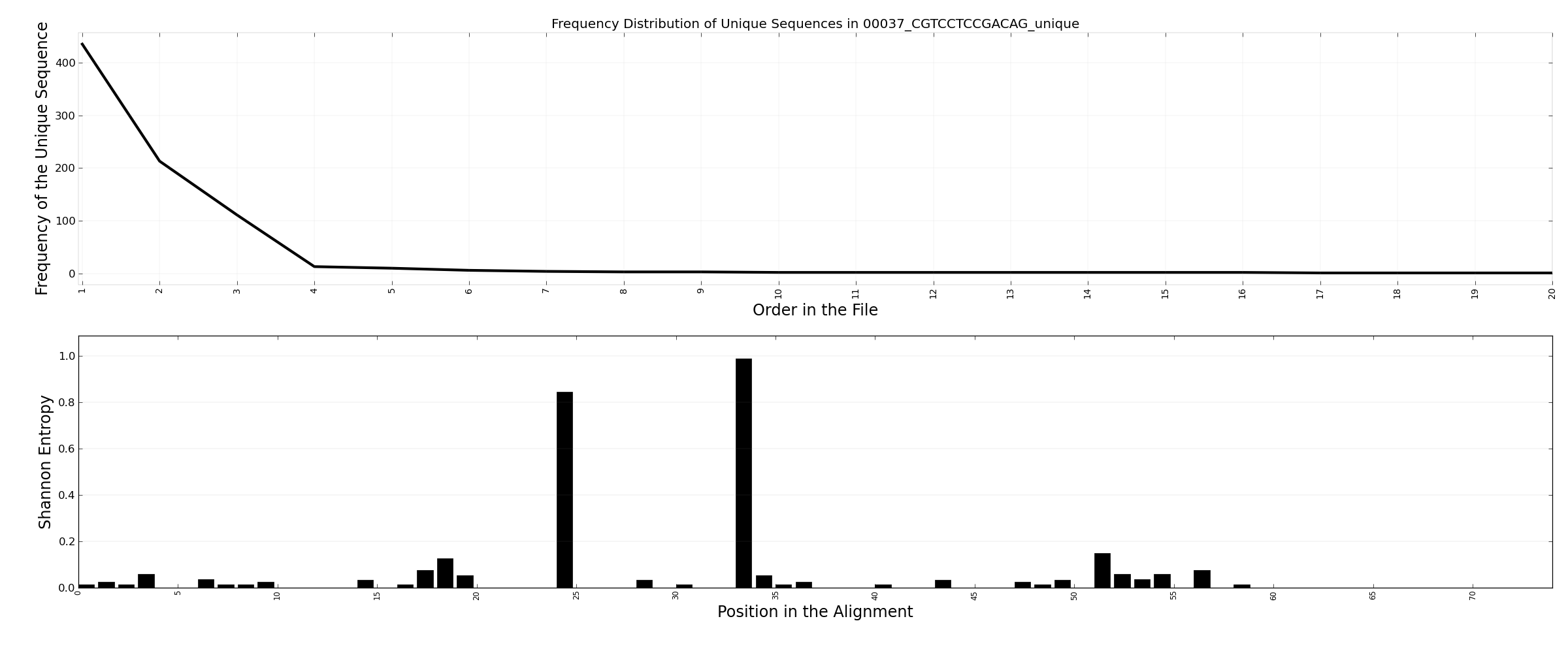

Supplement: Supplementary file 6 [file DataSheet2.ZIP › HTML-OUTPUT/00037_CGTCCTCCGACAG_unique.png]

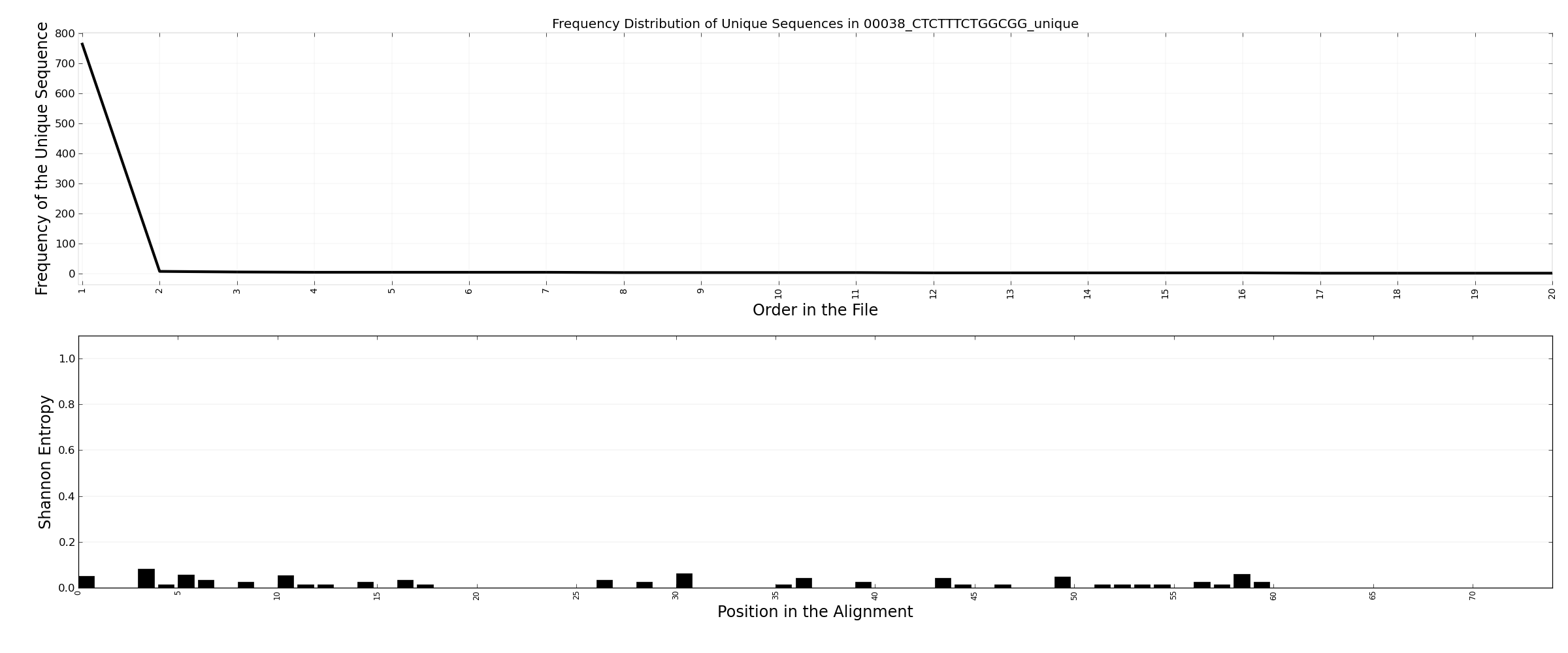

Supplement: Supplementary file 6 [file DataSheet2.ZIP › HTML-OUTPUT/00038_CTCTTTCTGGCGG_unique.png]

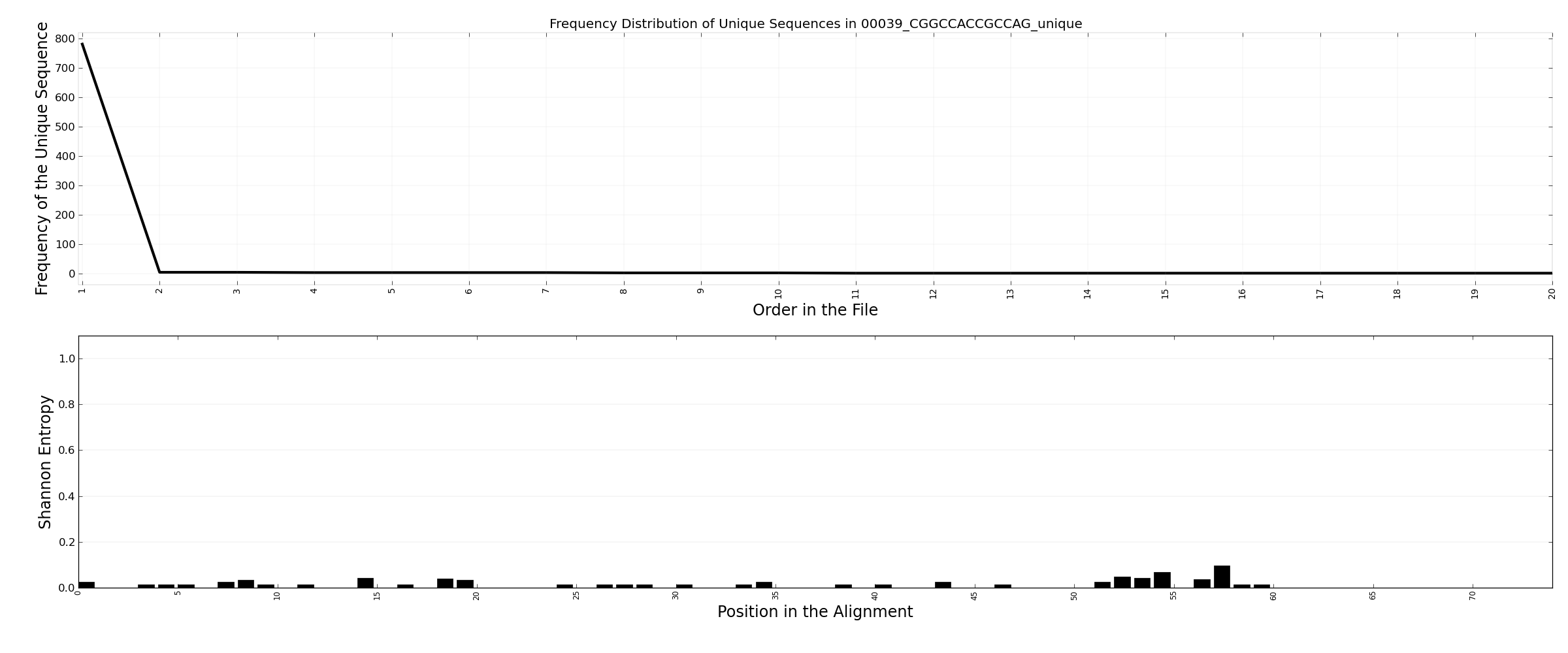

Supplement: Supplementary file 6 [file DataSheet2.ZIP › HTML-OUTPUT/00039_CGGCCACCGCCAG_unique.png]

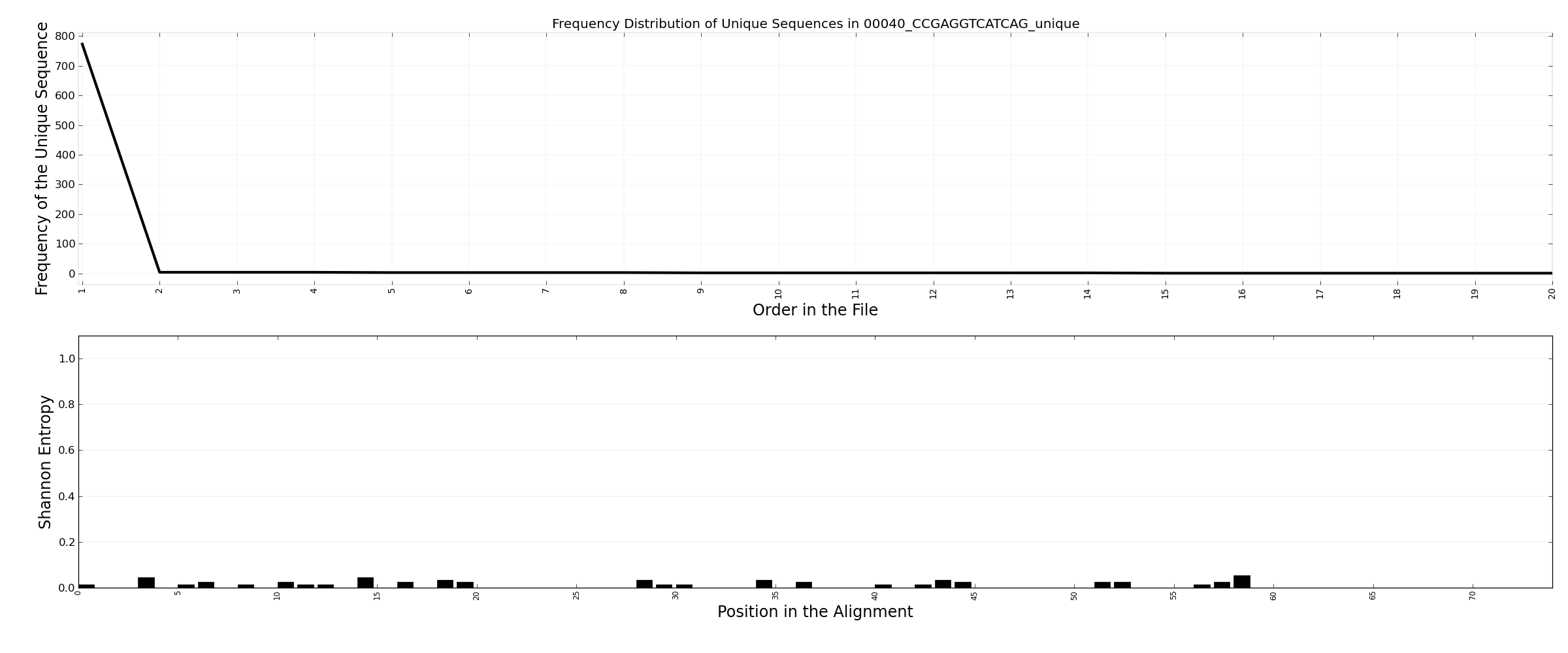

Supplement: Supplementary file 6 [file DataSheet2.ZIP › HTML-OUTPUT/00040_CCGAGGTCATCAG_unique.png]

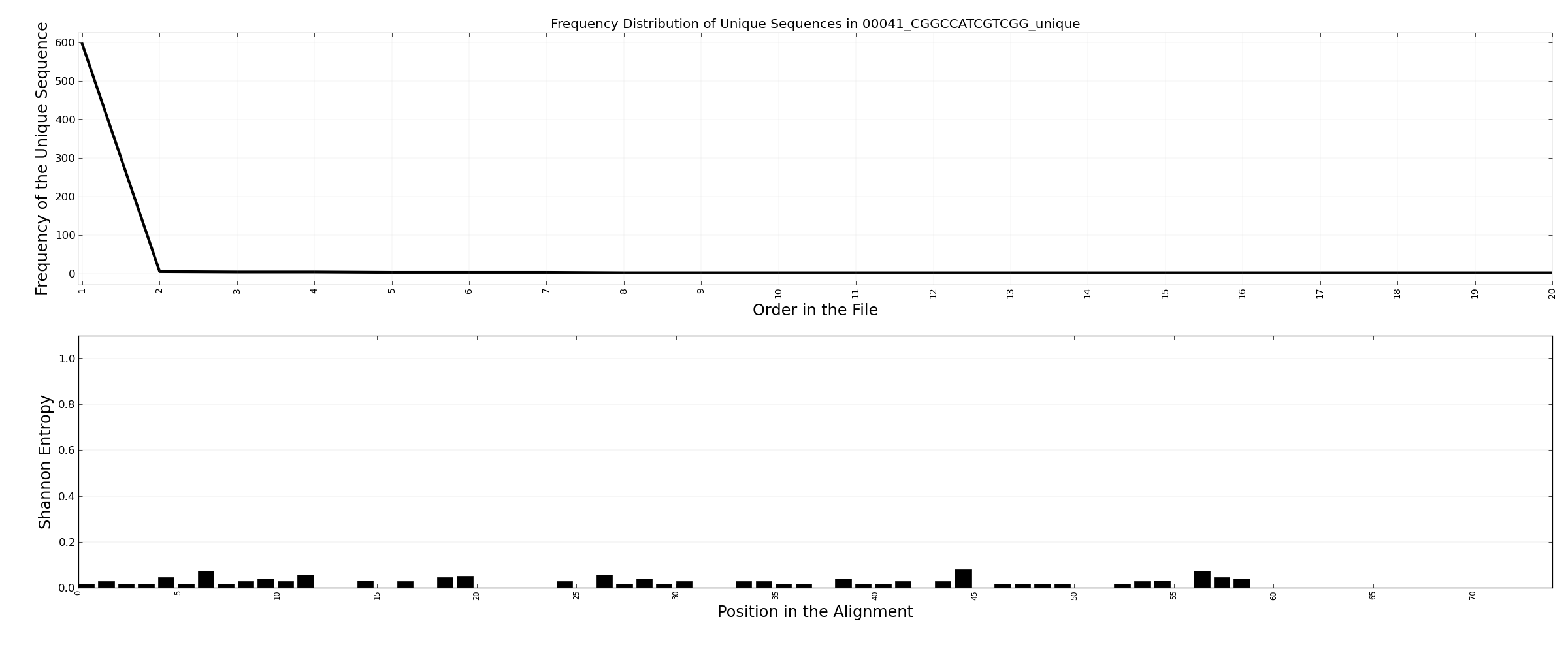

Supplement: Supplementary file 6 [file DataSheet2.ZIP › HTML-OUTPUT/00041_CGGCCATCGTCGG_unique.png]

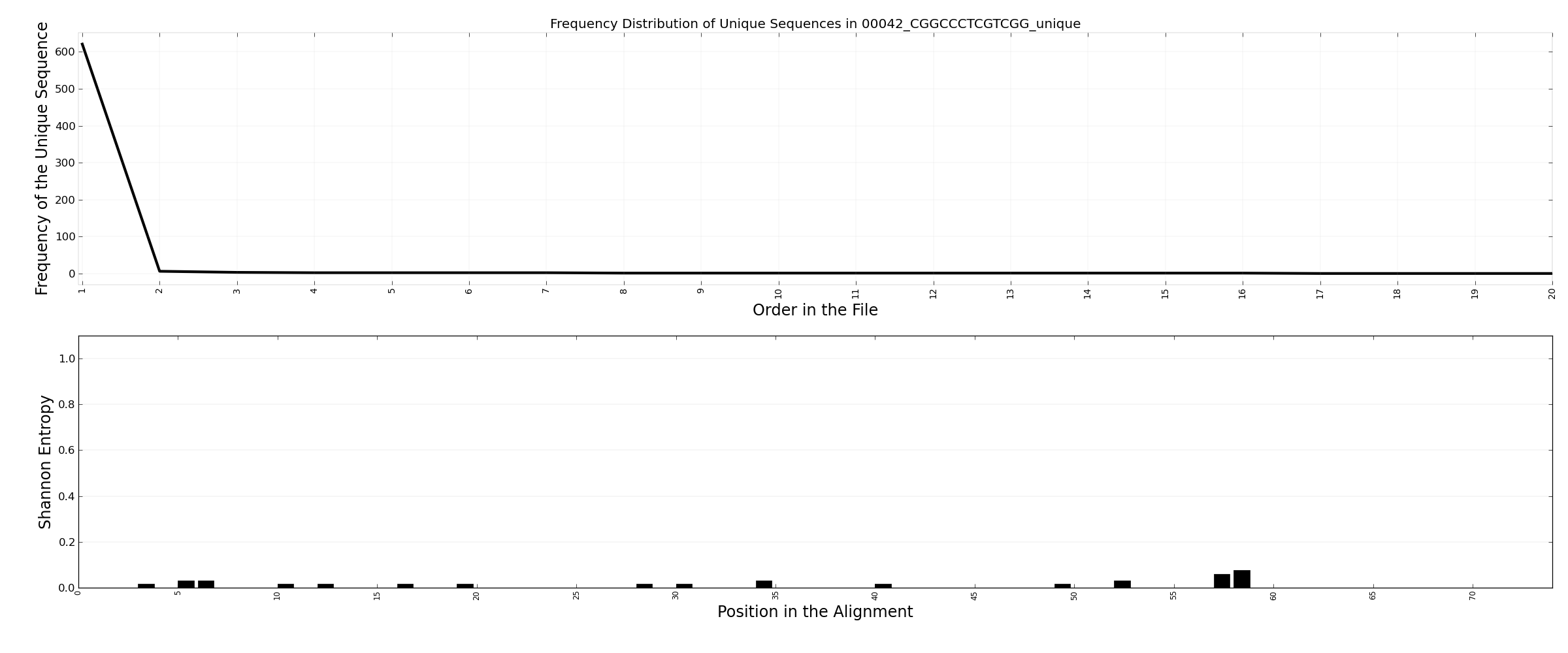

Supplement: Supplementary file 6 [file DataSheet2.ZIP › HTML-OUTPUT/00042_CGGCCCTCGTCGG_unique.png]

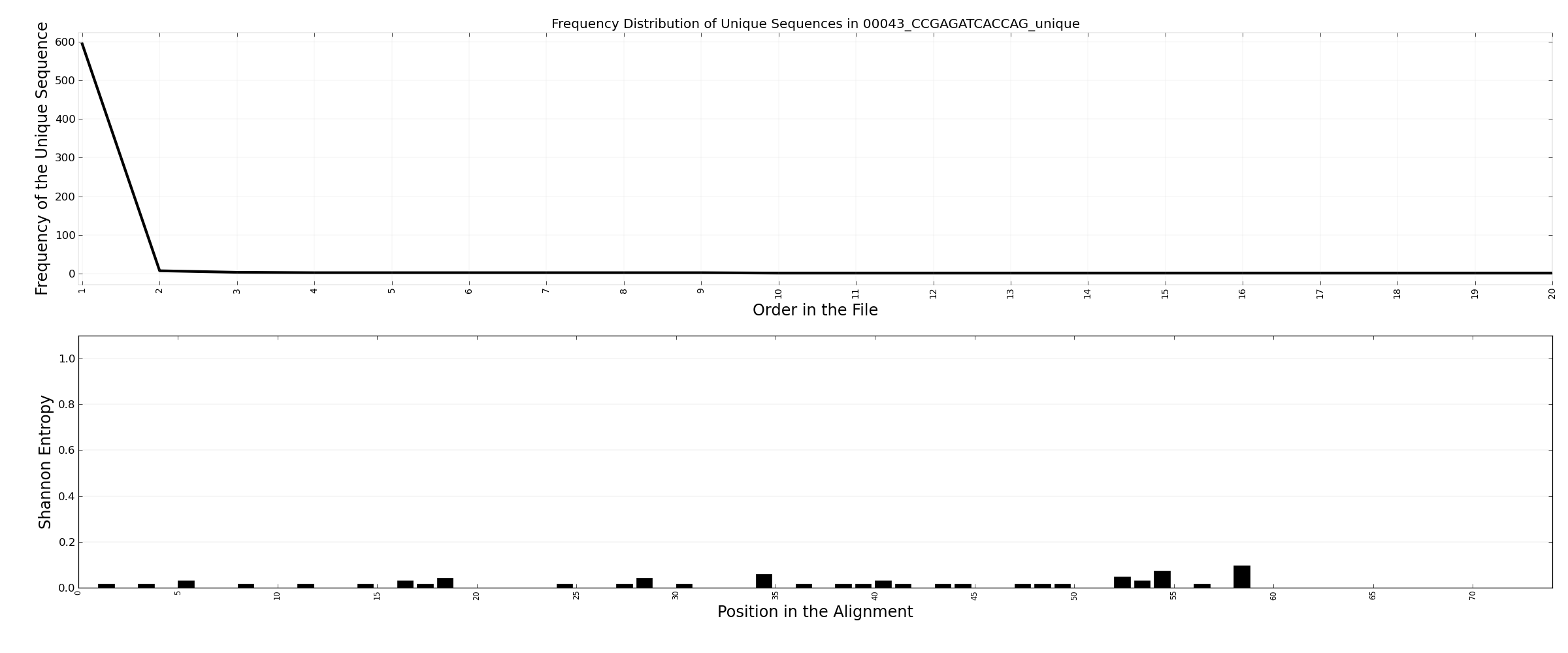

Supplement: Supplementary file 6 [file DataSheet2.ZIP › HTML-OUTPUT/00043_CCGAGATCACCAG_unique.png]

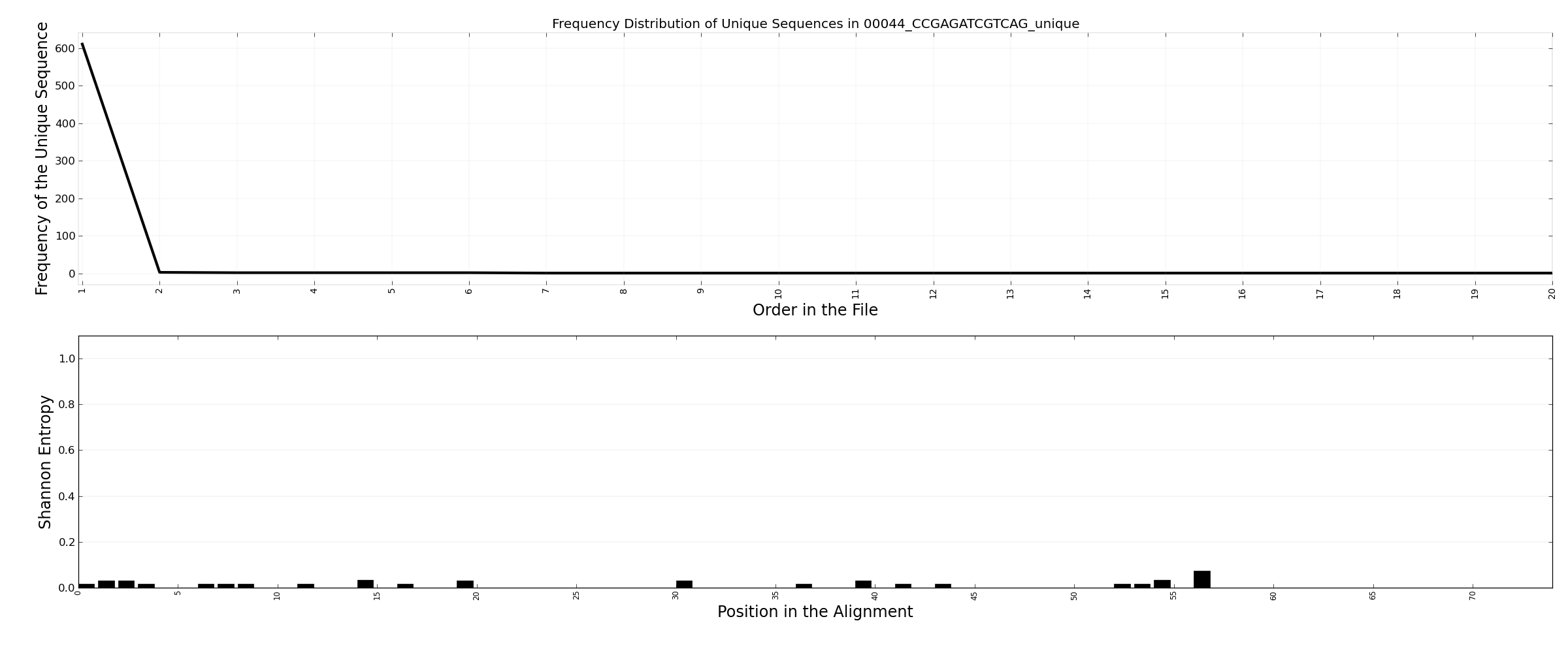

Supplement: Supplementary file 6 [file DataSheet2.ZIP › HTML-OUTPUT/00044_CCGAGATCGTCAG_unique.png]

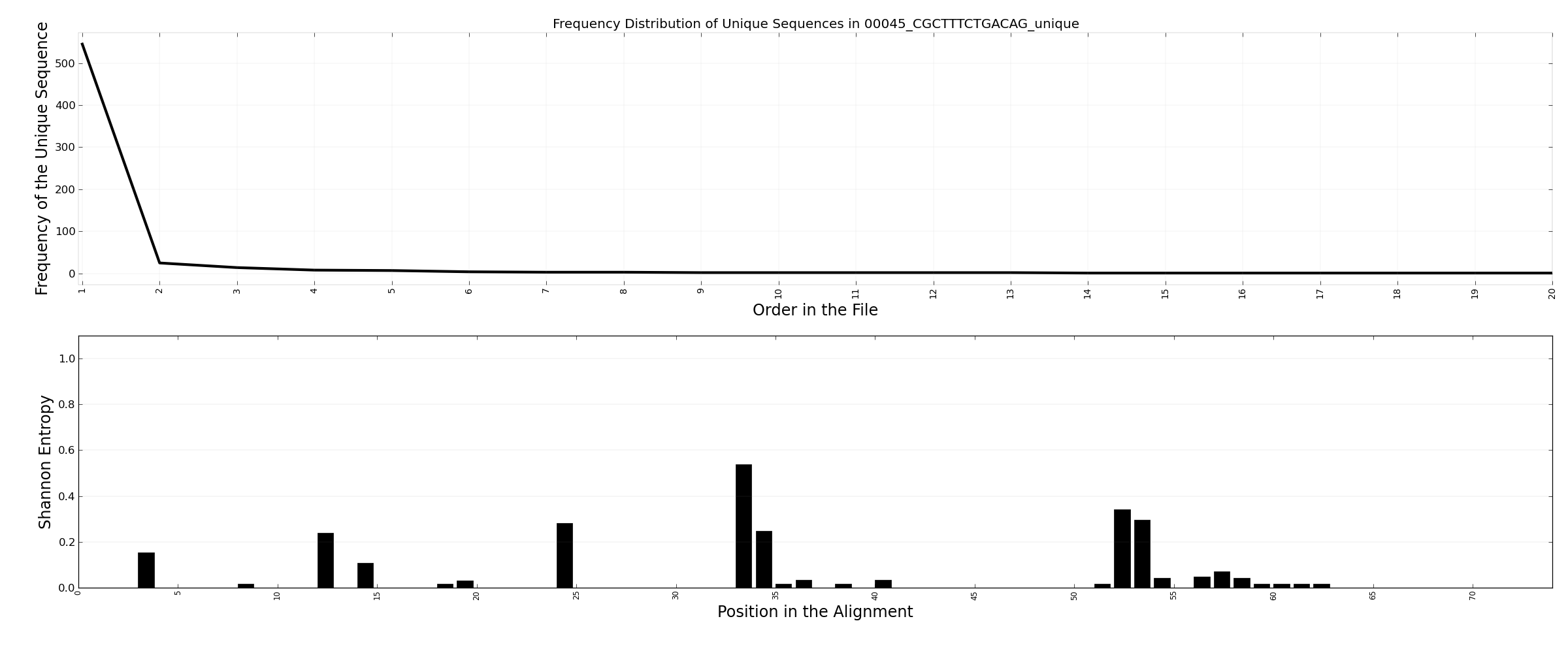

Supplement: Supplementary file 6 [file DataSheet2.ZIP › HTML-OUTPUT/00045_CGCTTTCTGACAG_unique.png]

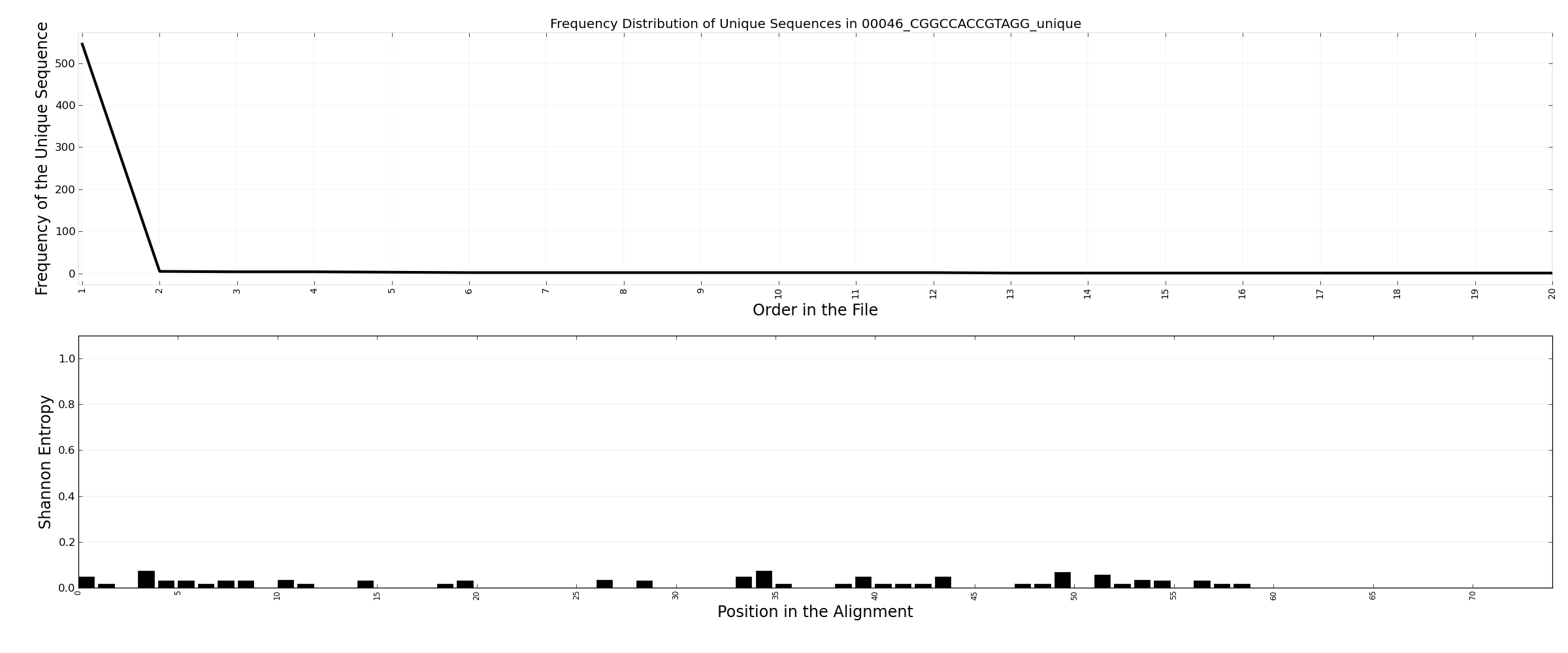

Supplement: Supplementary file 6 [file DataSheet2.ZIP › HTML-OUTPUT/00046_CGGCCACCGTAGG_unique.png]

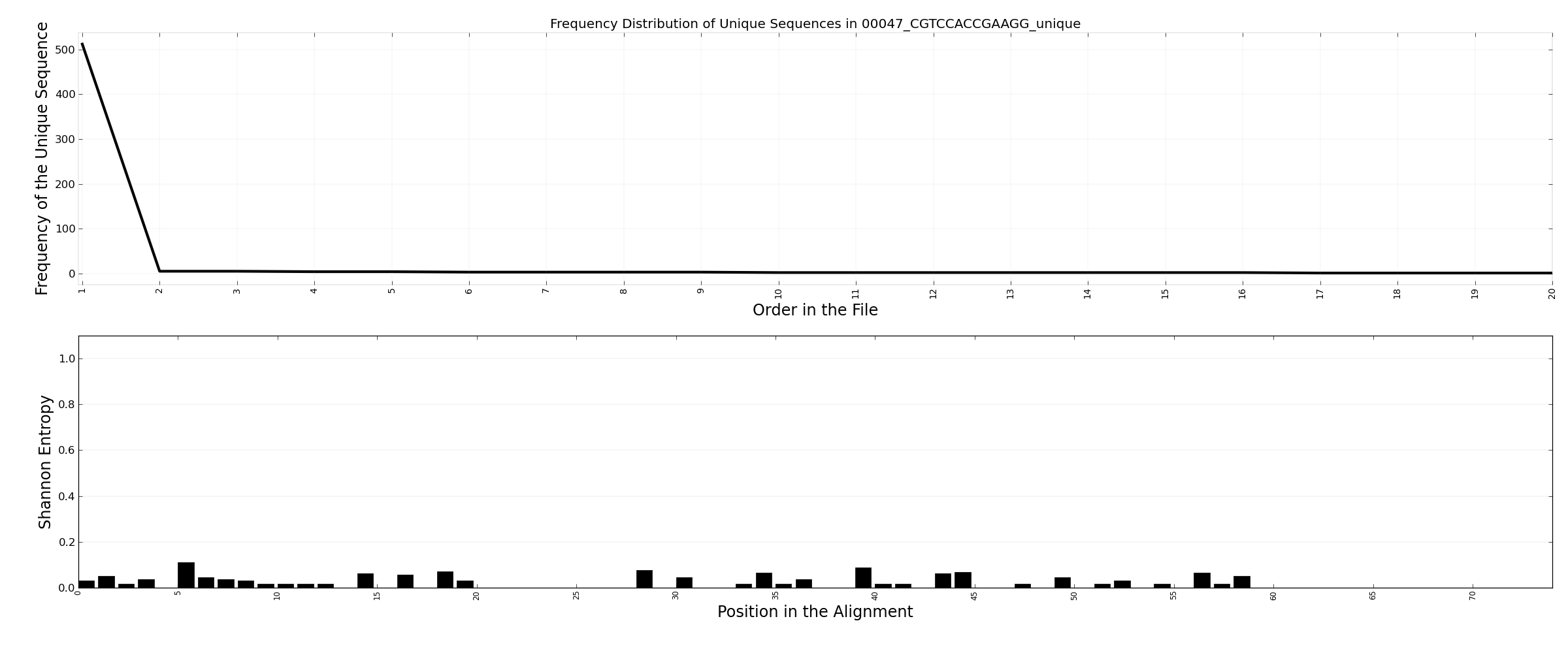

Supplement: Supplementary file 6 [file DataSheet2.ZIP › HTML-OUTPUT/00047_CGTCCACCGAAGG_unique.png]

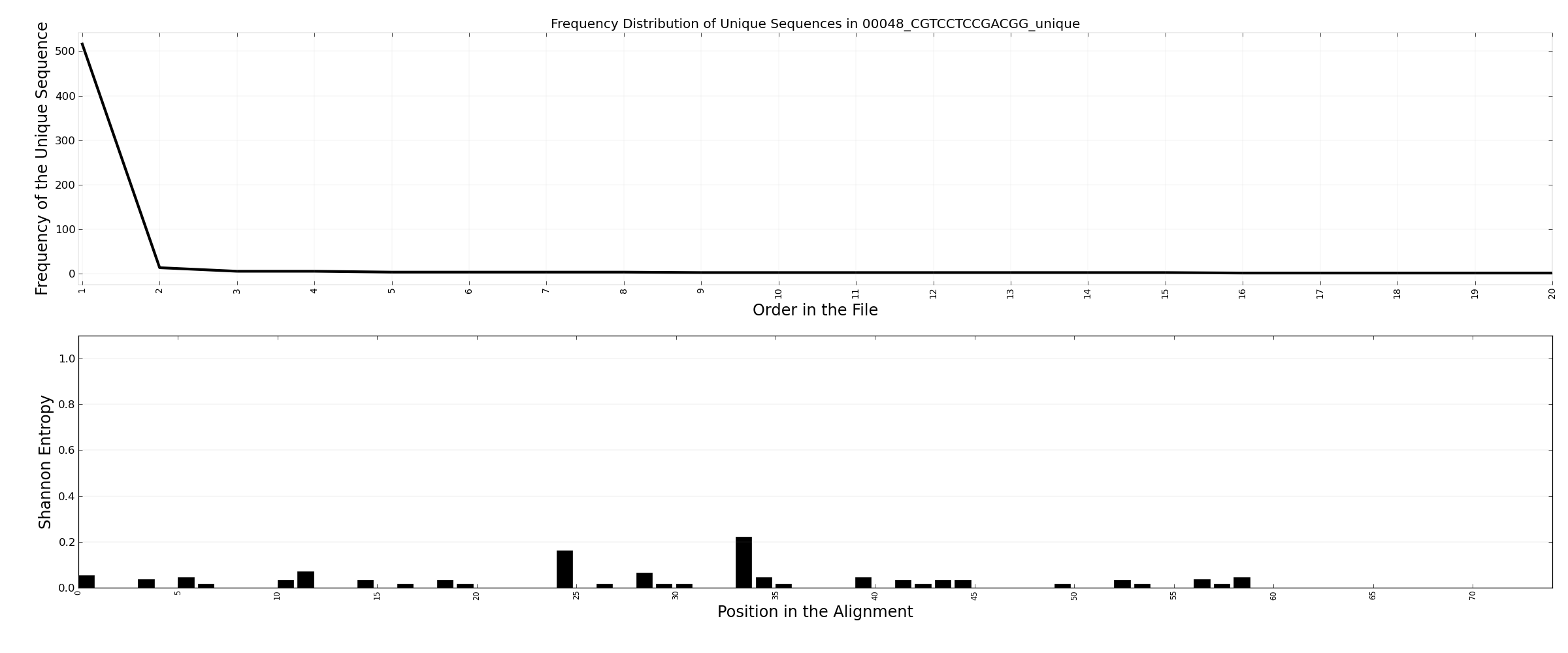

Supplement: Supplementary file 6 [file DataSheet2.ZIP › HTML-OUTPUT/00048_CGTCCTCCGACGG_unique.png]

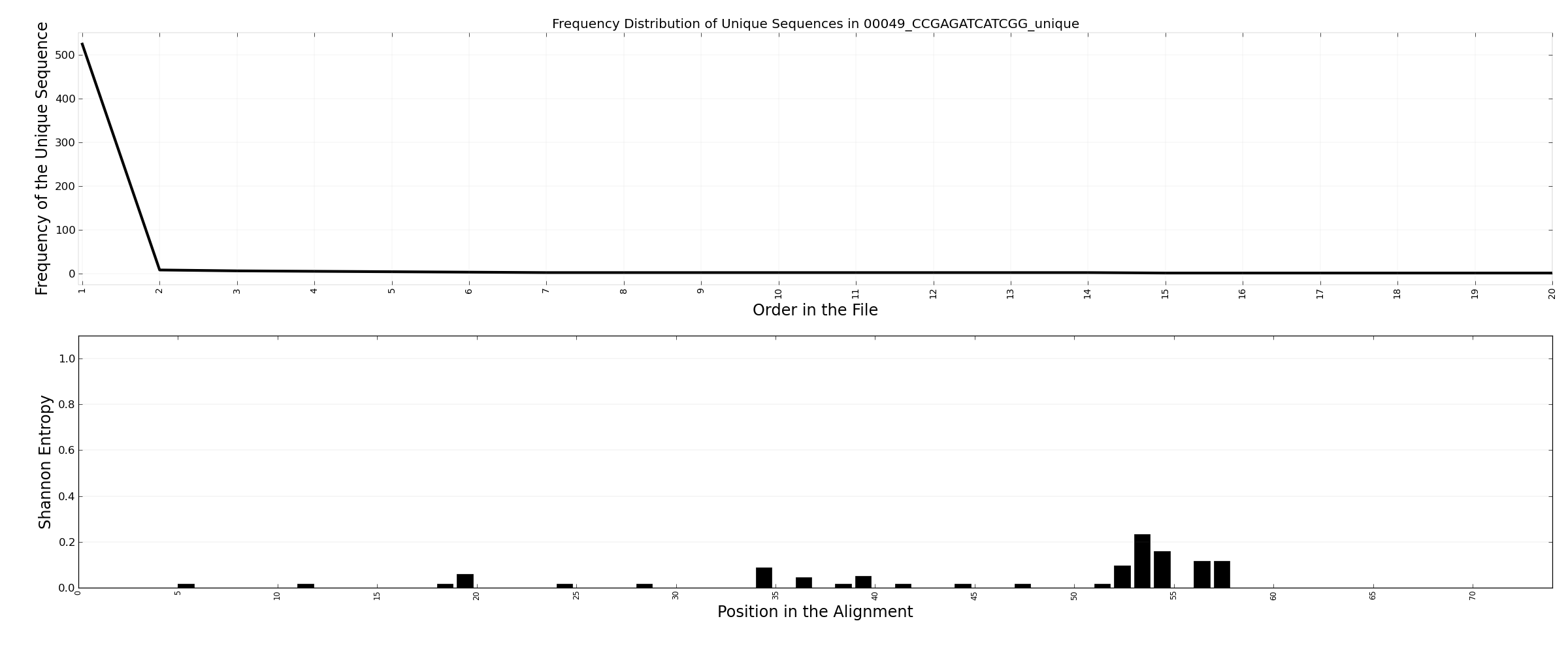

Supplement: Supplementary file 6 [file DataSheet2.ZIP › HTML-OUTPUT/00049_CCGAGATCATCGG_unique.png]

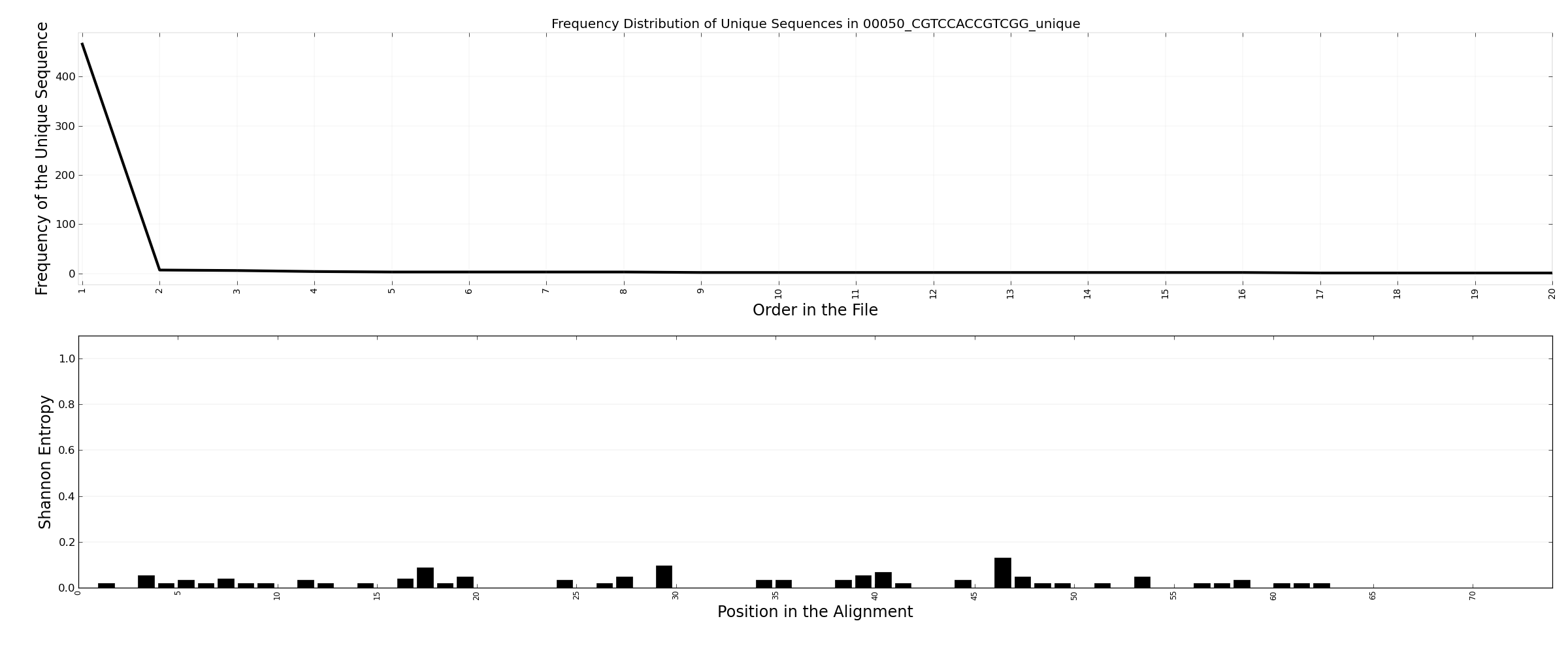

Supplement: Supplementary file 6 [file DataSheet2.ZIP › HTML-OUTPUT/00050_CGTCCACCGTCGG_unique.png]

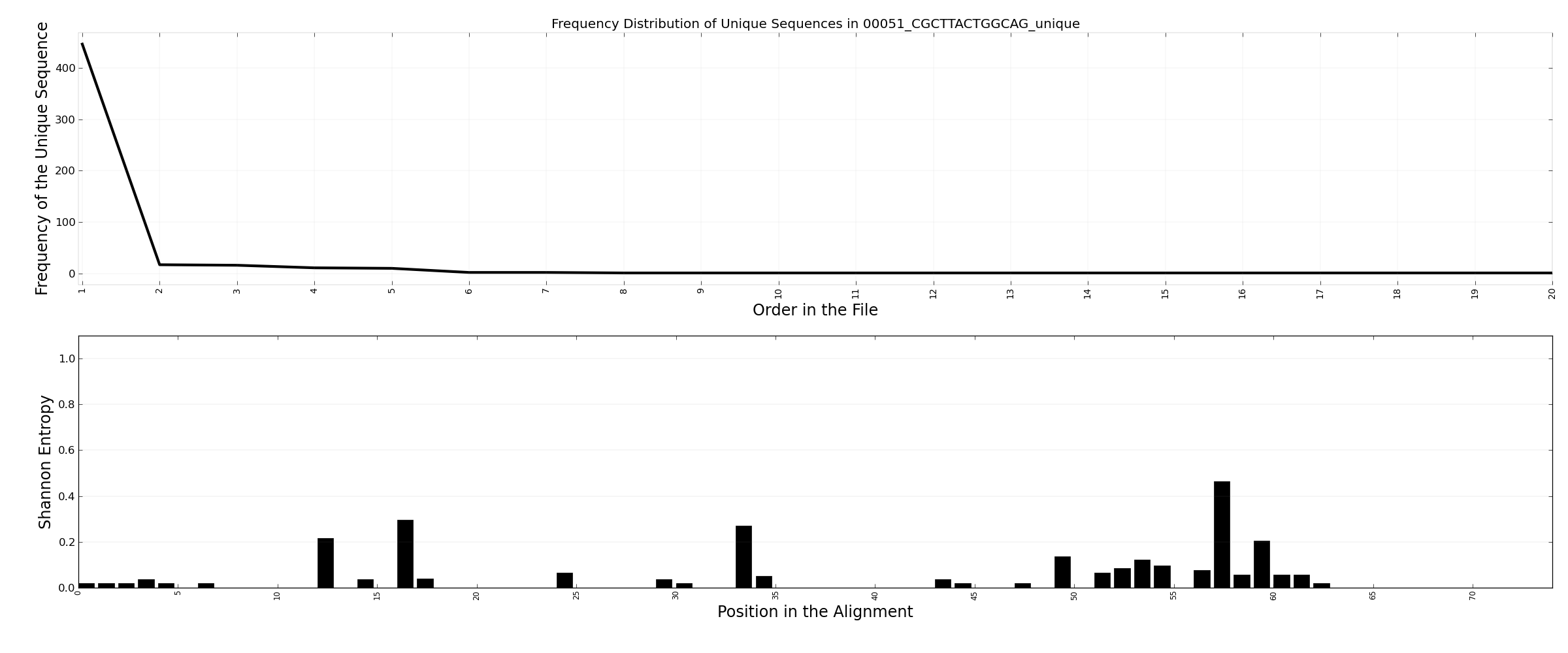

Supplement: Supplementary file 6 [file DataSheet2.ZIP › HTML-OUTPUT/00051_CGCTTACTGGCAG_unique.png]

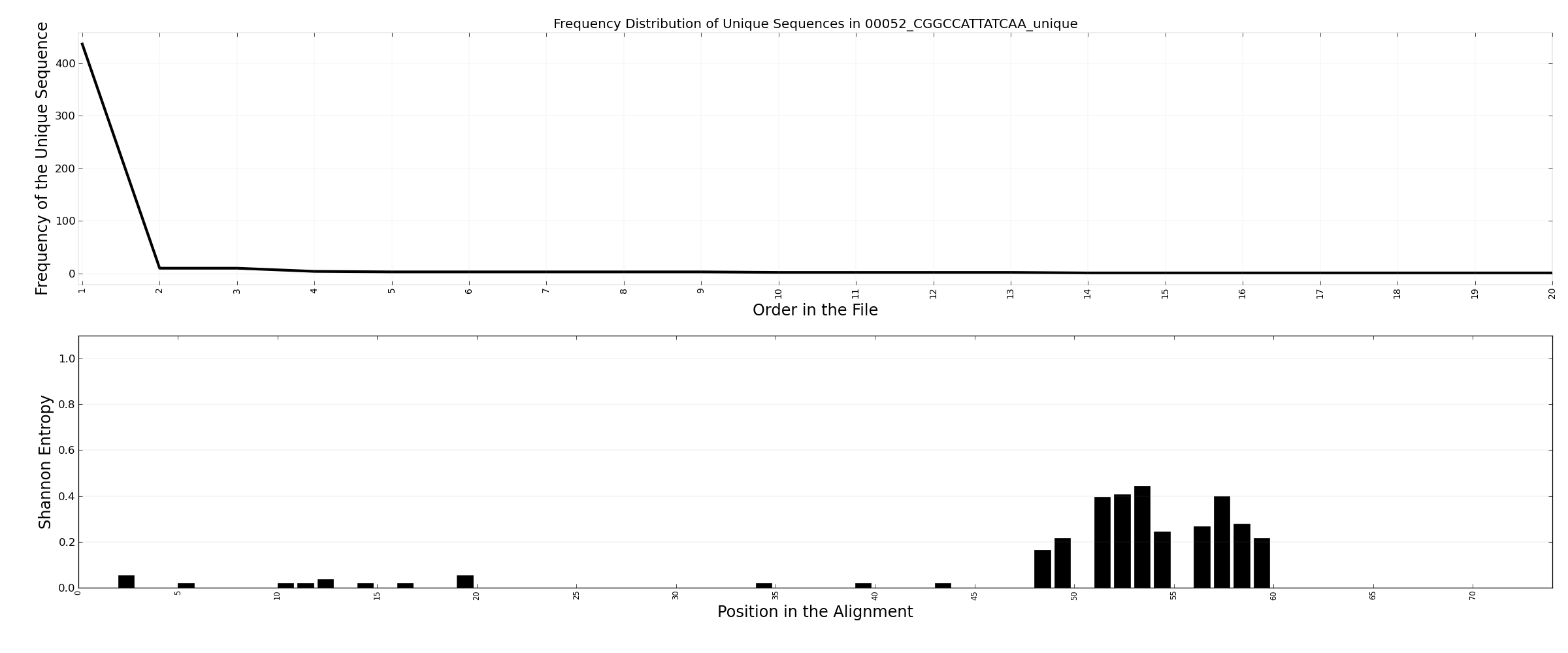

Supplement: Supplementary file 6 [file DataSheet2.ZIP › HTML-OUTPUT/00052_CGGCCATTATCAA_unique.png]

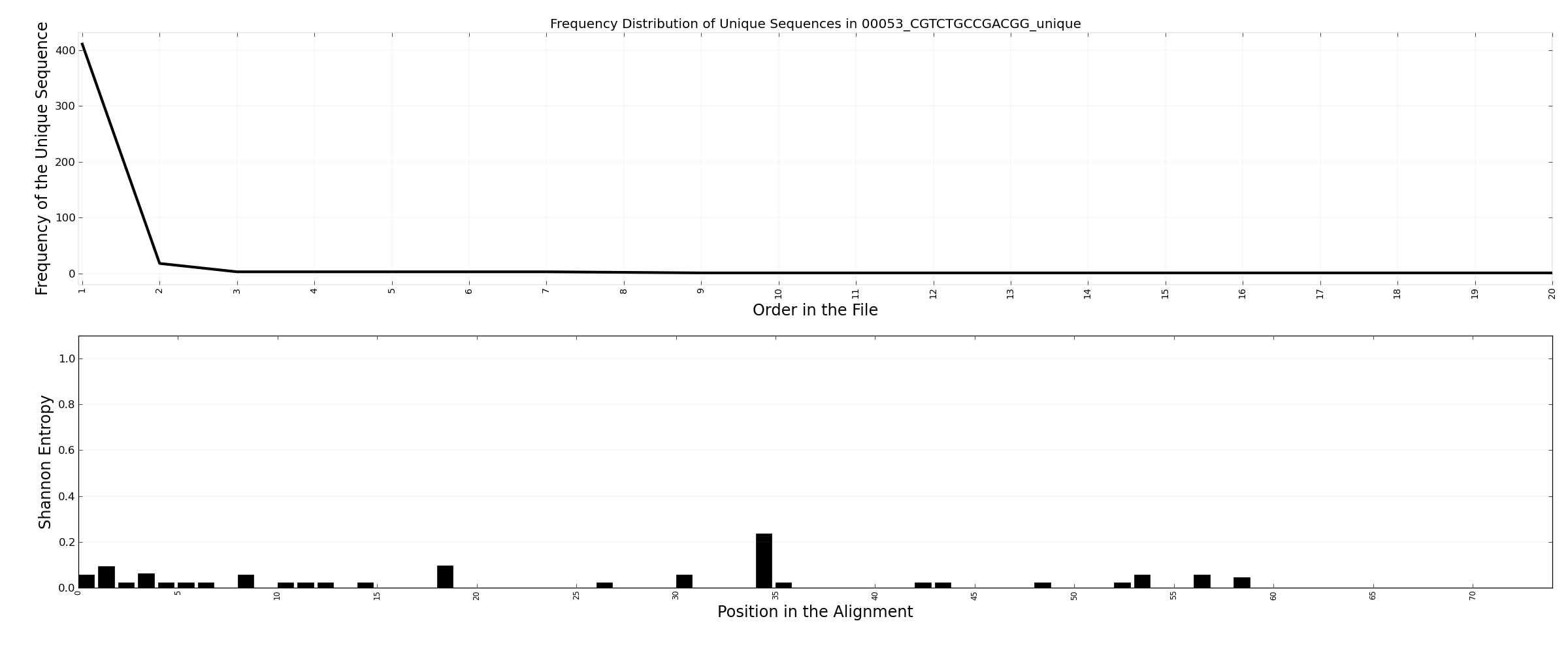

Supplement: Supplementary file 6 [file DataSheet2.ZIP › HTML-OUTPUT/00053_CGTCTGCCGACGG_unique.png]

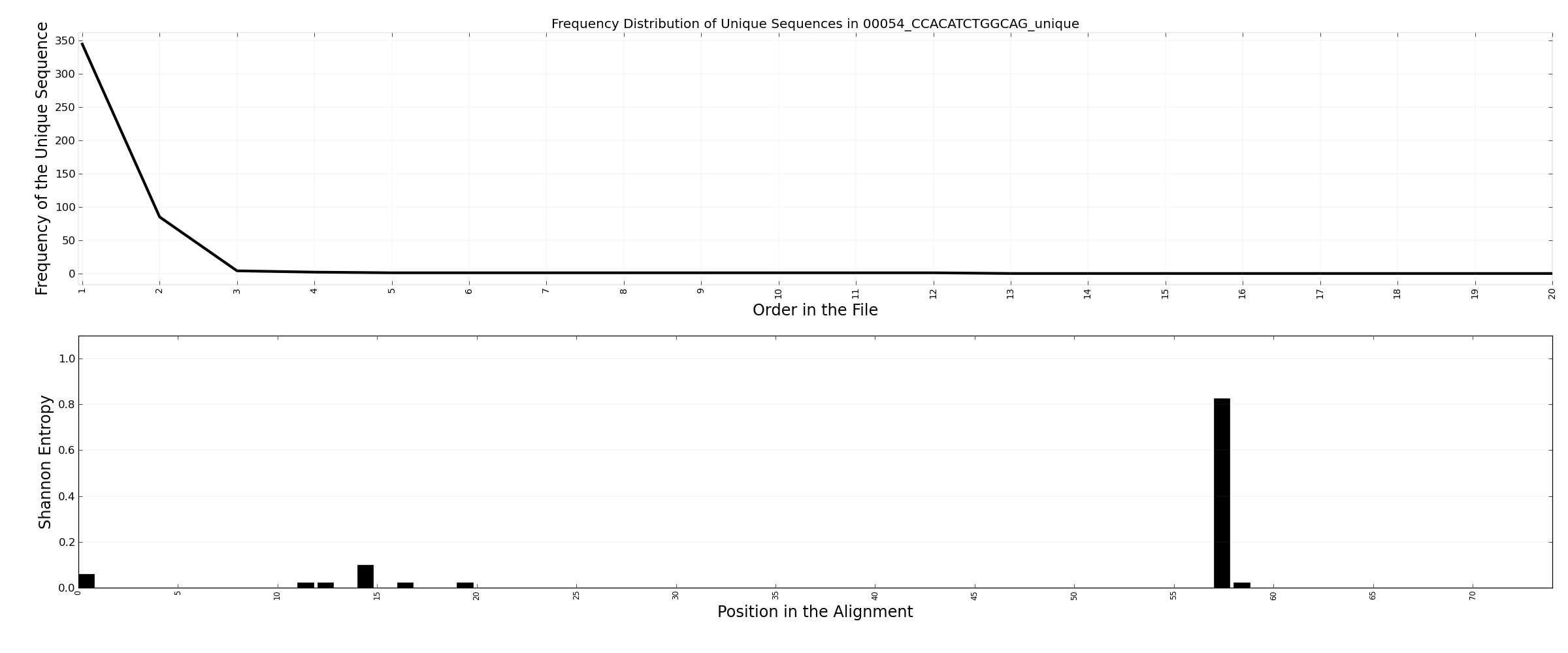

Supplement: Supplementary file 6 [file DataSheet2.ZIP › HTML-OUTPUT/00054_CCACATCTGGCAG_unique.png]

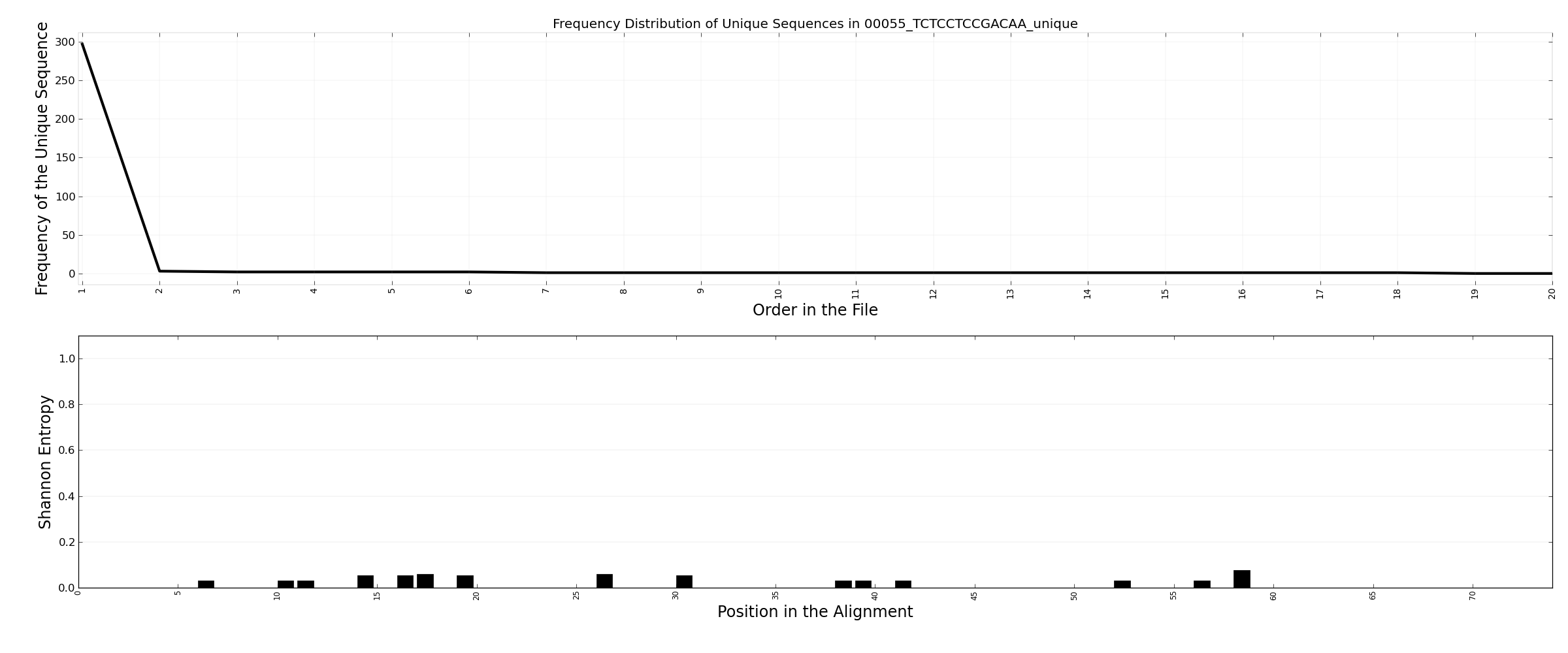

Supplement: Supplementary file 6 [file DataSheet2.ZIP › HTML-OUTPUT/00055_TCTCCTCCGACAA_unique.png]

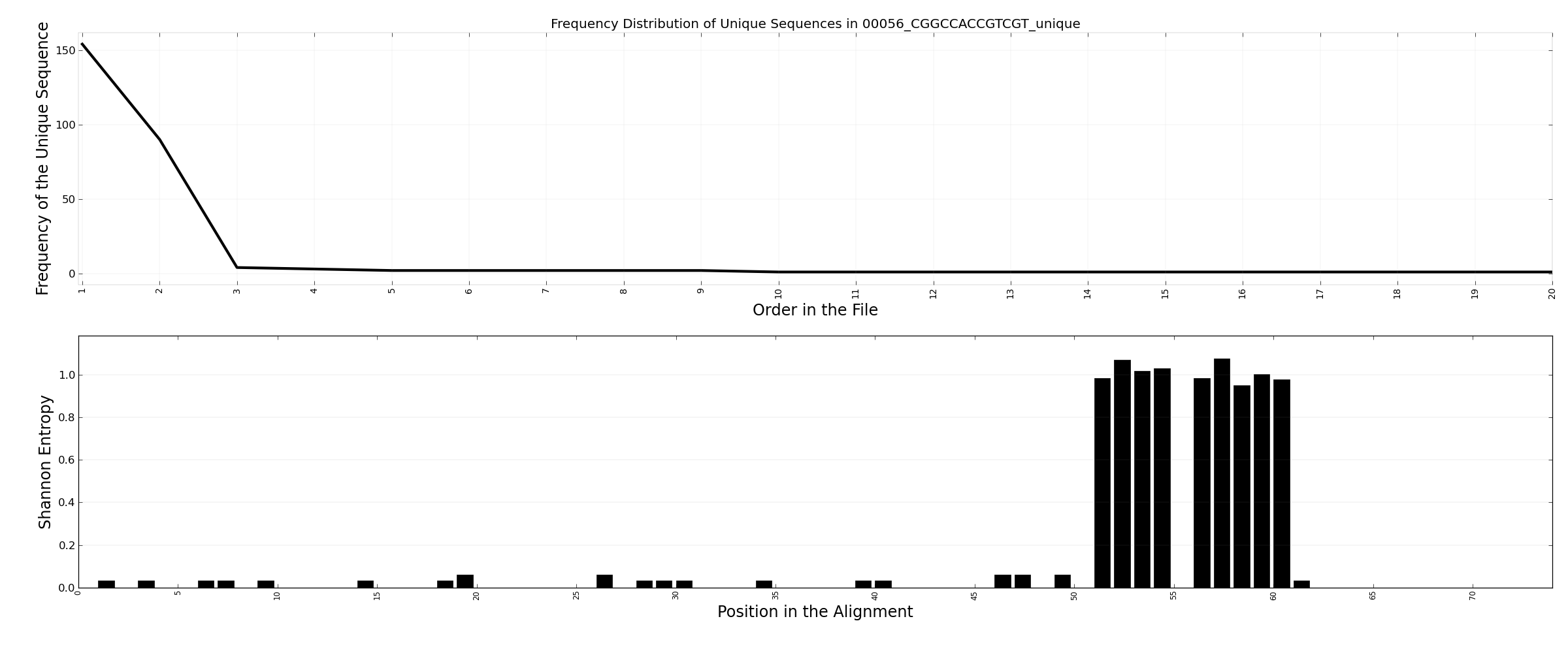

Supplement: Supplementary file 6 [file DataSheet2.ZIP › HTML-OUTPUT/00056_CGGCCACCGTCGT_unique.png]

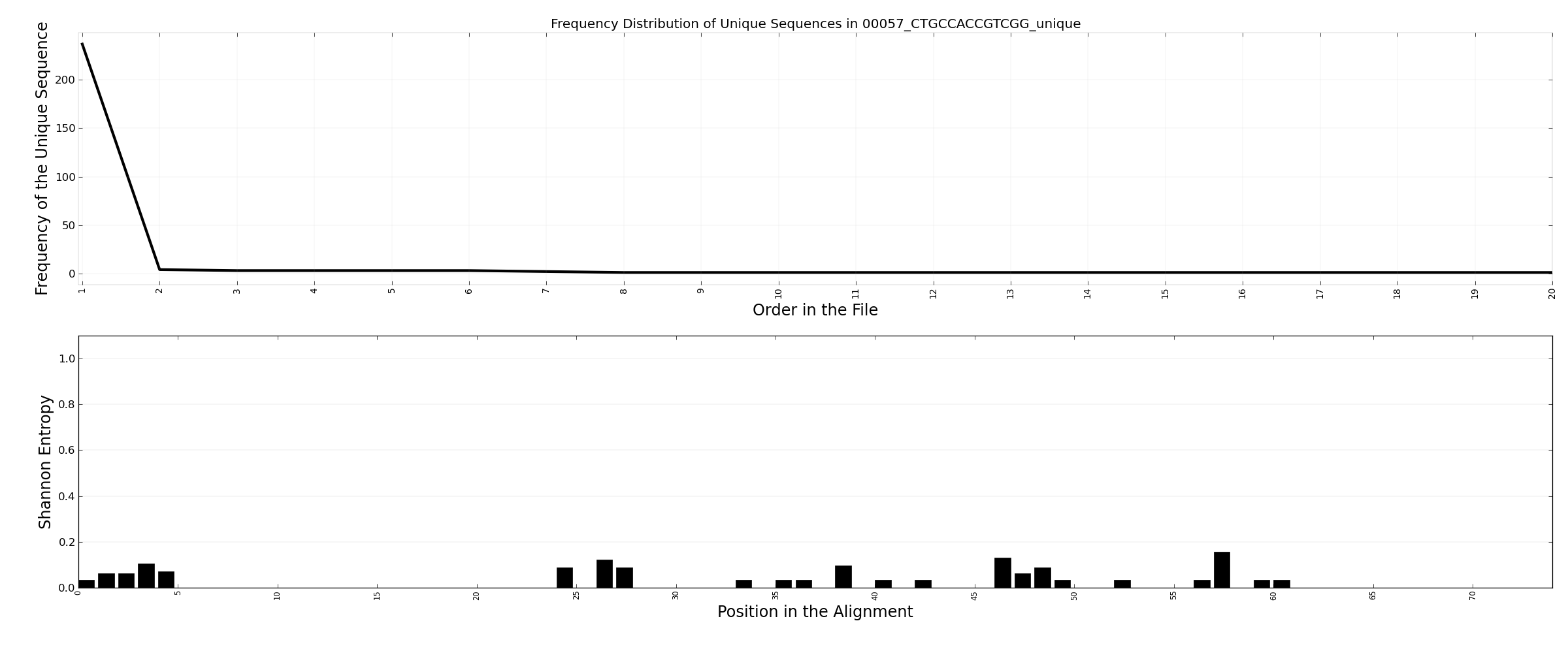

Supplement: Supplementary file 6 [file DataSheet2.ZIP › HTML-OUTPUT/00057_CTGCCACCGTCGG_unique.png]

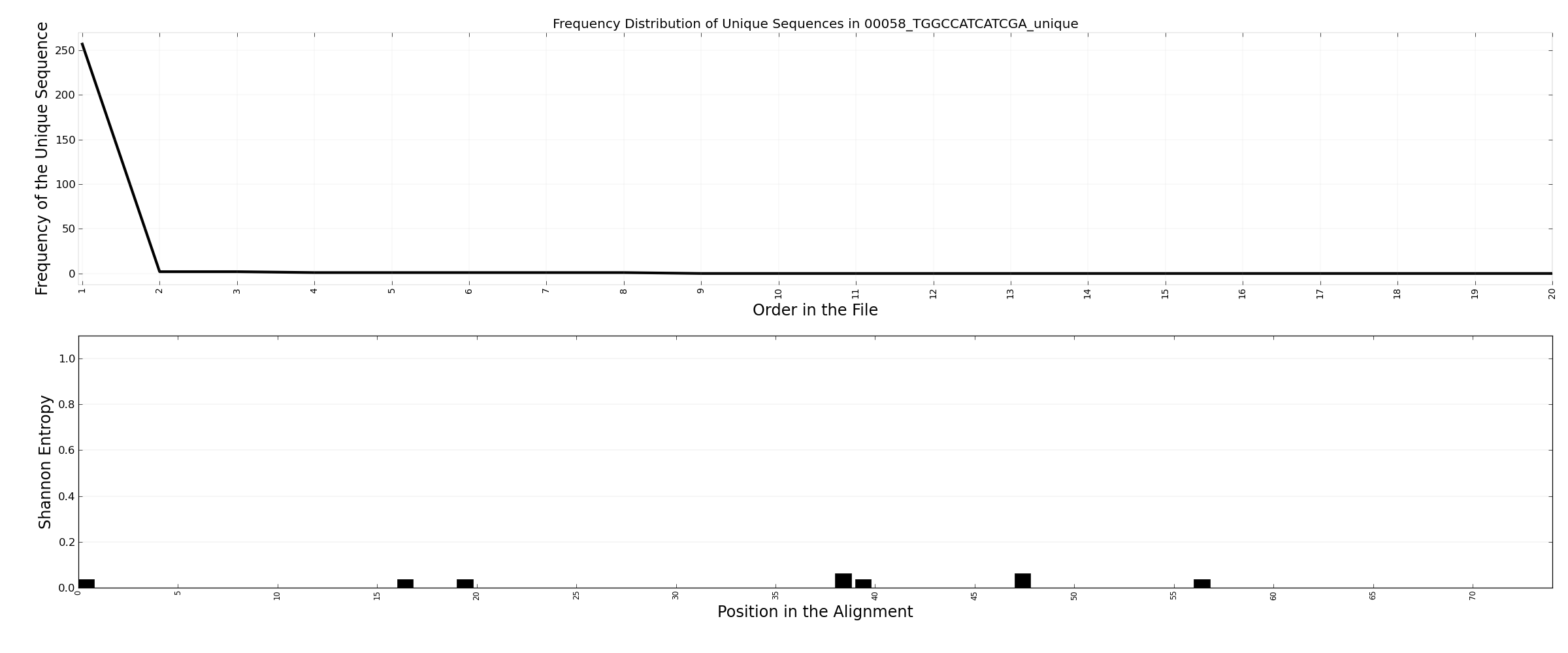

Supplement: Supplementary file 6 [file DataSheet2.ZIP › HTML-OUTPUT/00058_TGGCCATCATCGA_unique.png]

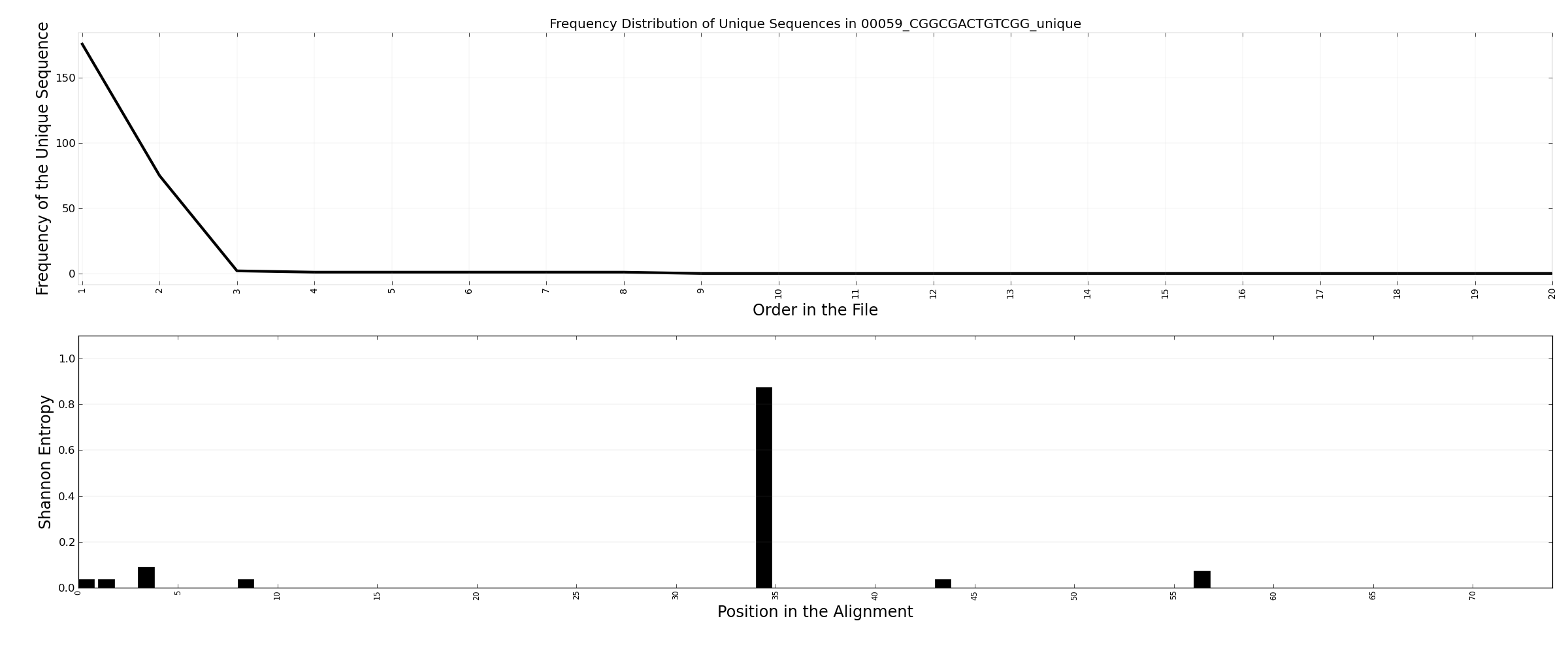

Supplement: Supplementary file 6 [file DataSheet2.ZIP › HTML-OUTPUT/00059_CGGCGACTGTCGG_unique.png]

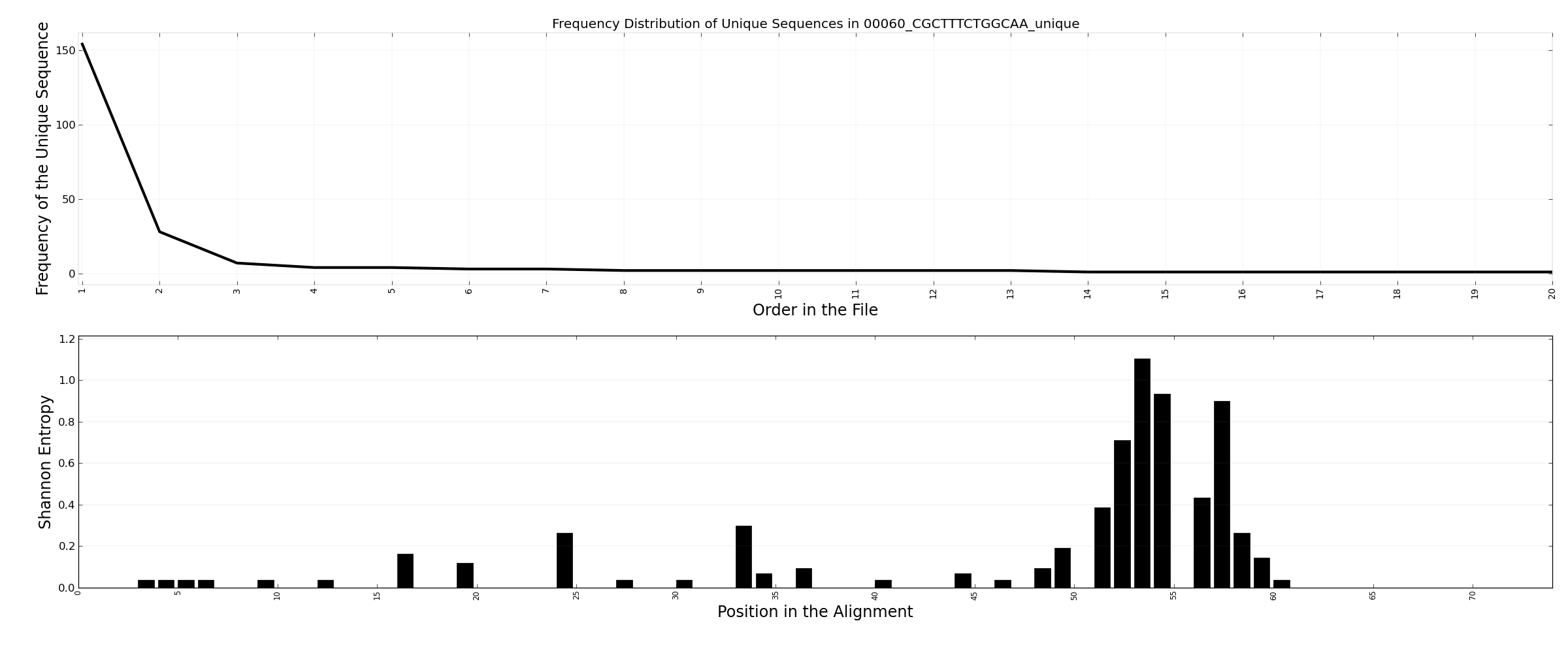

Supplement: Supplementary file 6 [file DataSheet2.ZIP › HTML-OUTPUT/00060_CGCTTTCTGGCAA_unique.png]

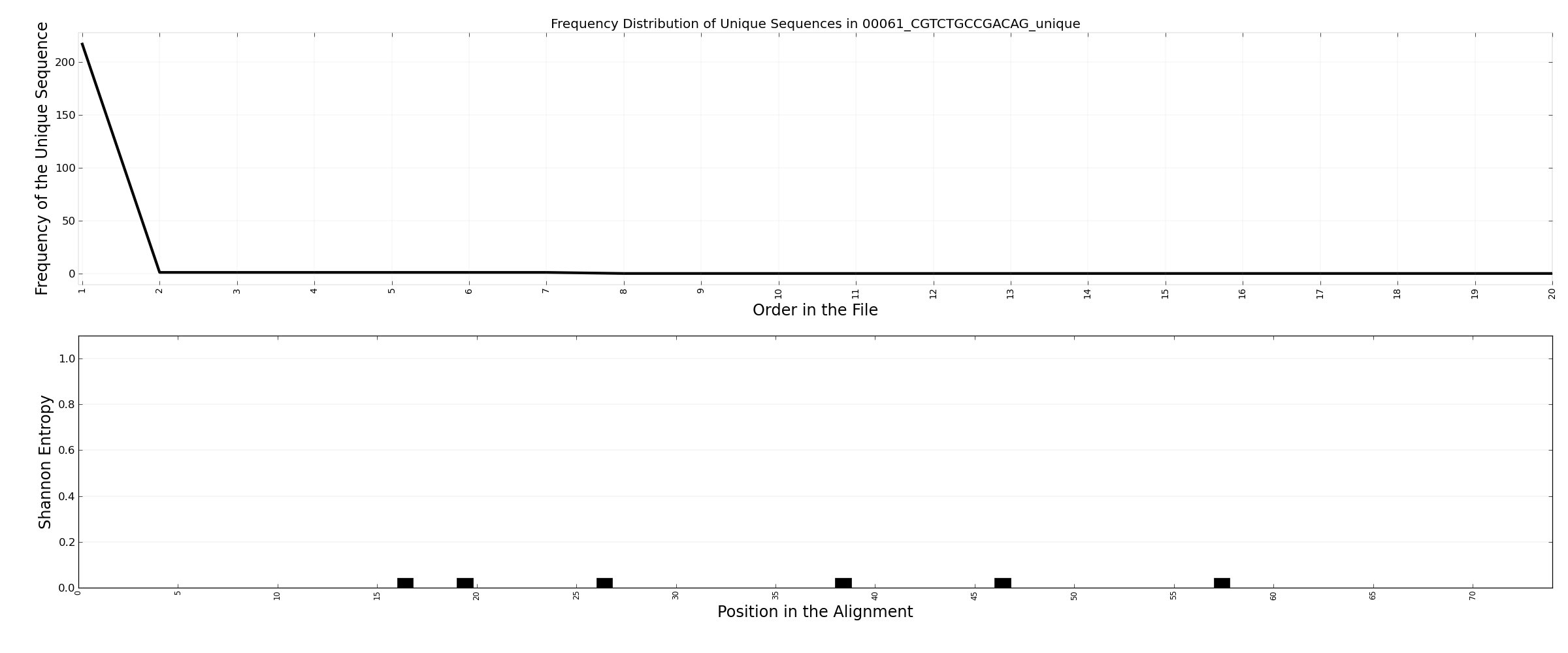

Supplement: Supplementary file 6 [file DataSheet2.ZIP › HTML-OUTPUT/00061_CGTCTGCCGACAG_unique.png]

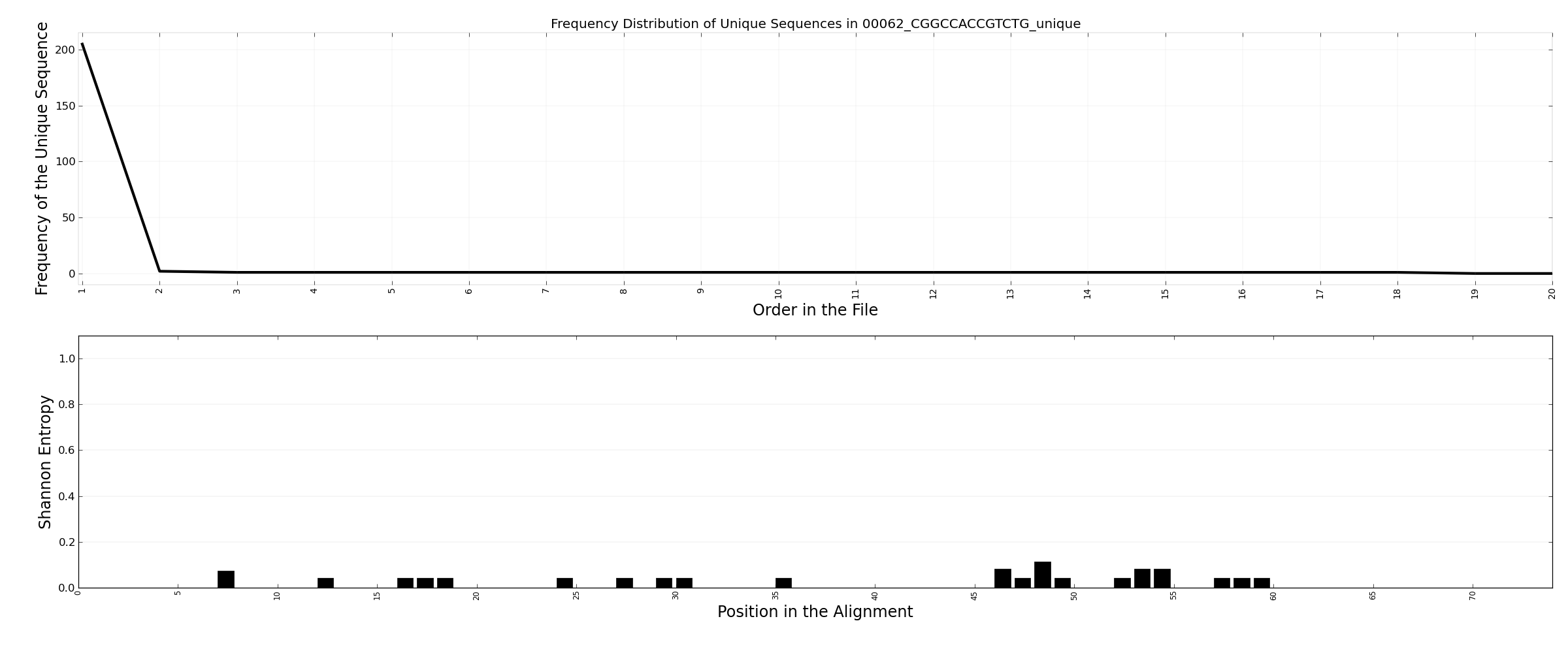

Supplement: Supplementary file 6 [file DataSheet2.ZIP › HTML-OUTPUT/00062_CGGCCACCGTCTG_unique.png]

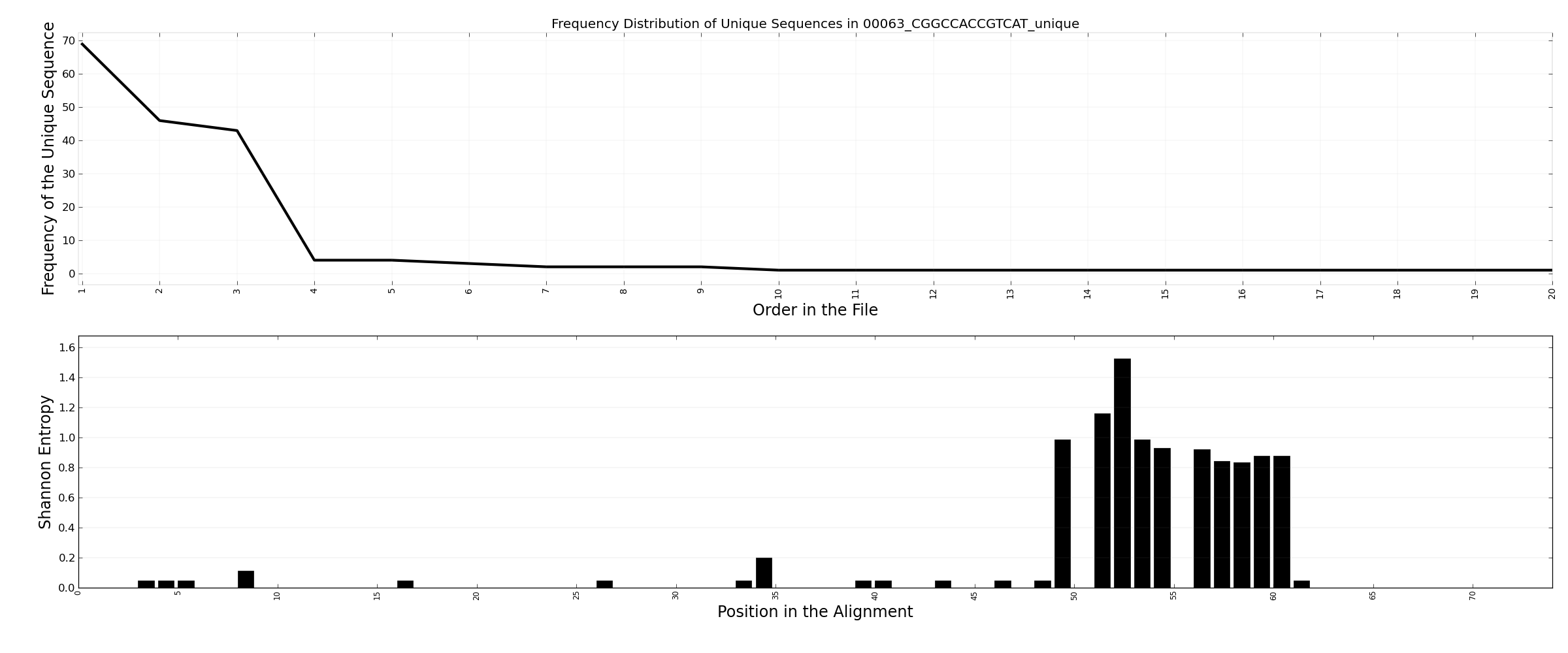

Supplement: Supplementary file 6 [file DataSheet2.ZIP › HTML-OUTPUT/00063_CGGCCACCGTCAT_unique.png]

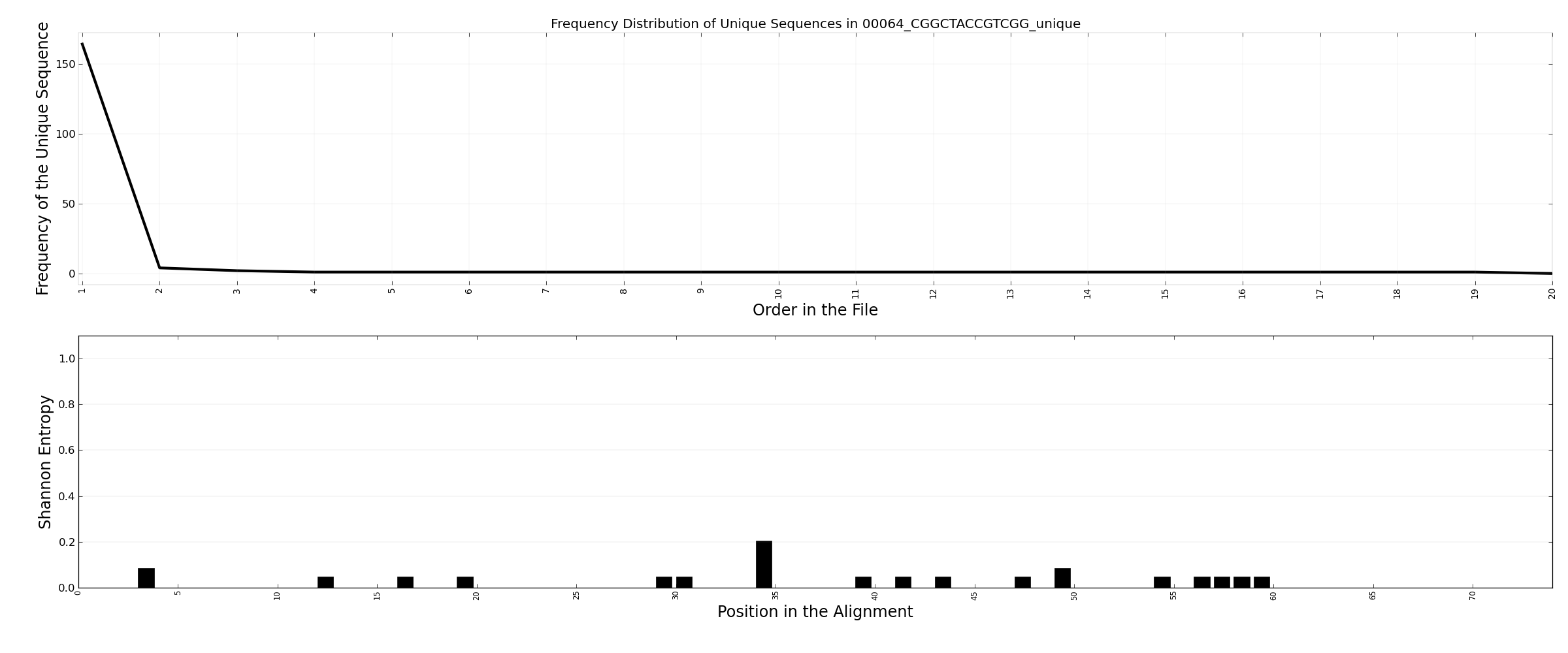

Supplement: Supplementary file 6 [file DataSheet2.ZIP › HTML-OUTPUT/00064_CGGCTACCGTCGG_unique.png]

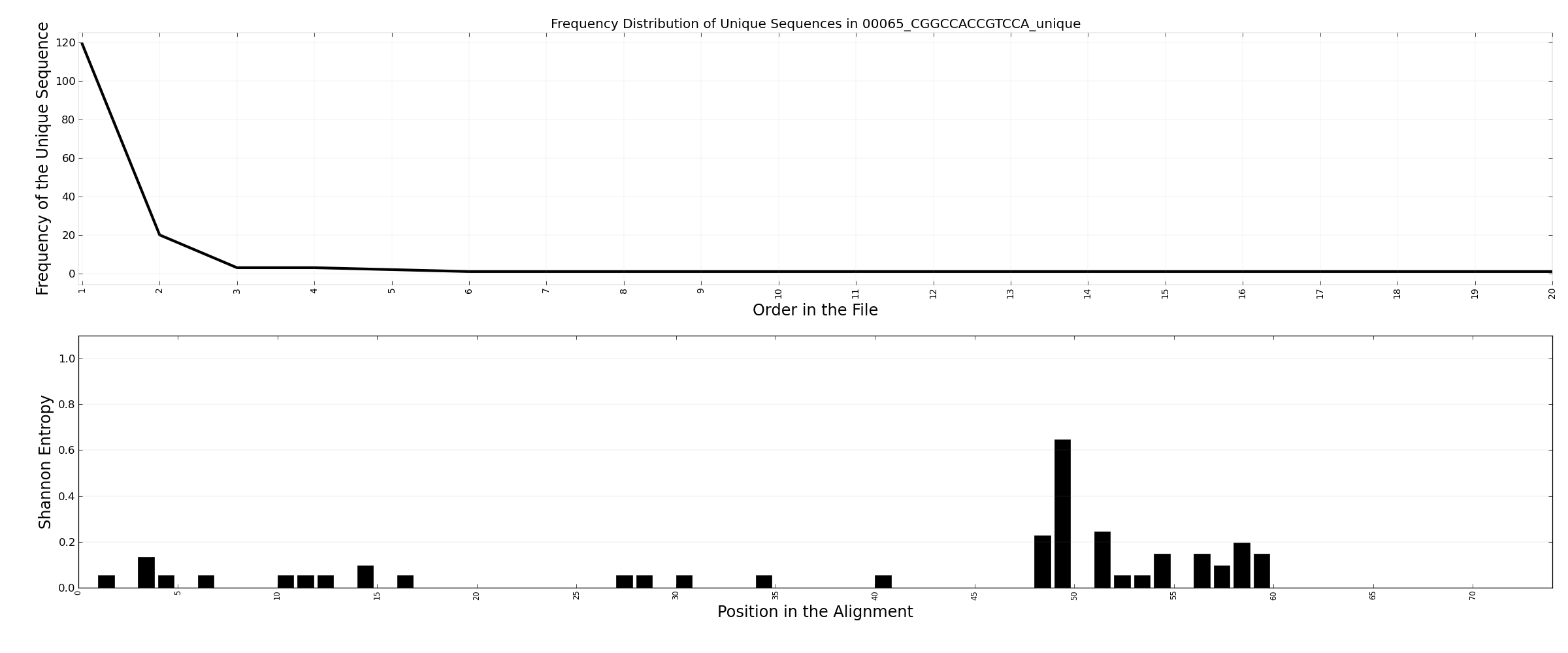

Supplement: Supplementary file 6 [file DataSheet2.ZIP › HTML-OUTPUT/00065_CGGCCACCGTCCA_unique.png]

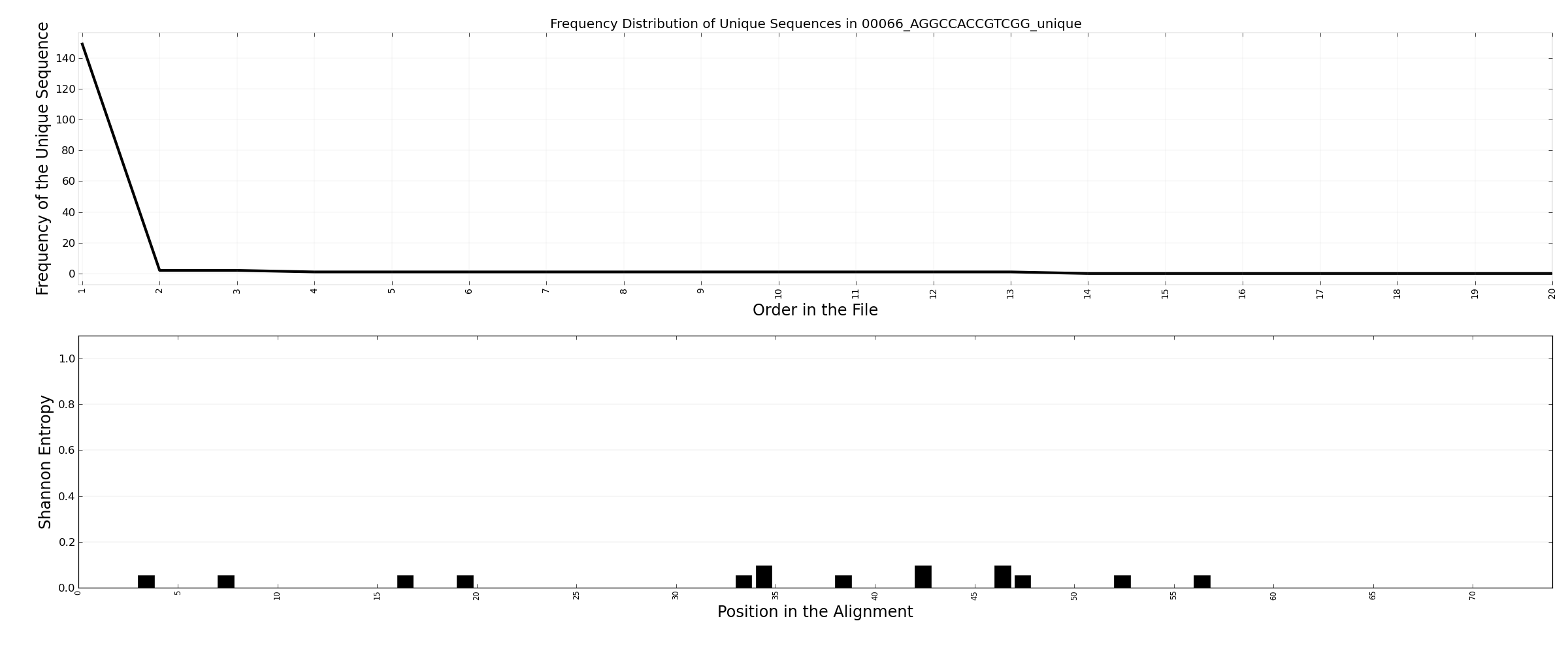

Supplement: Supplementary file 6 [file DataSheet2.ZIP › HTML-OUTPUT/00066_AGGCCACCGTCGG_unique.png]

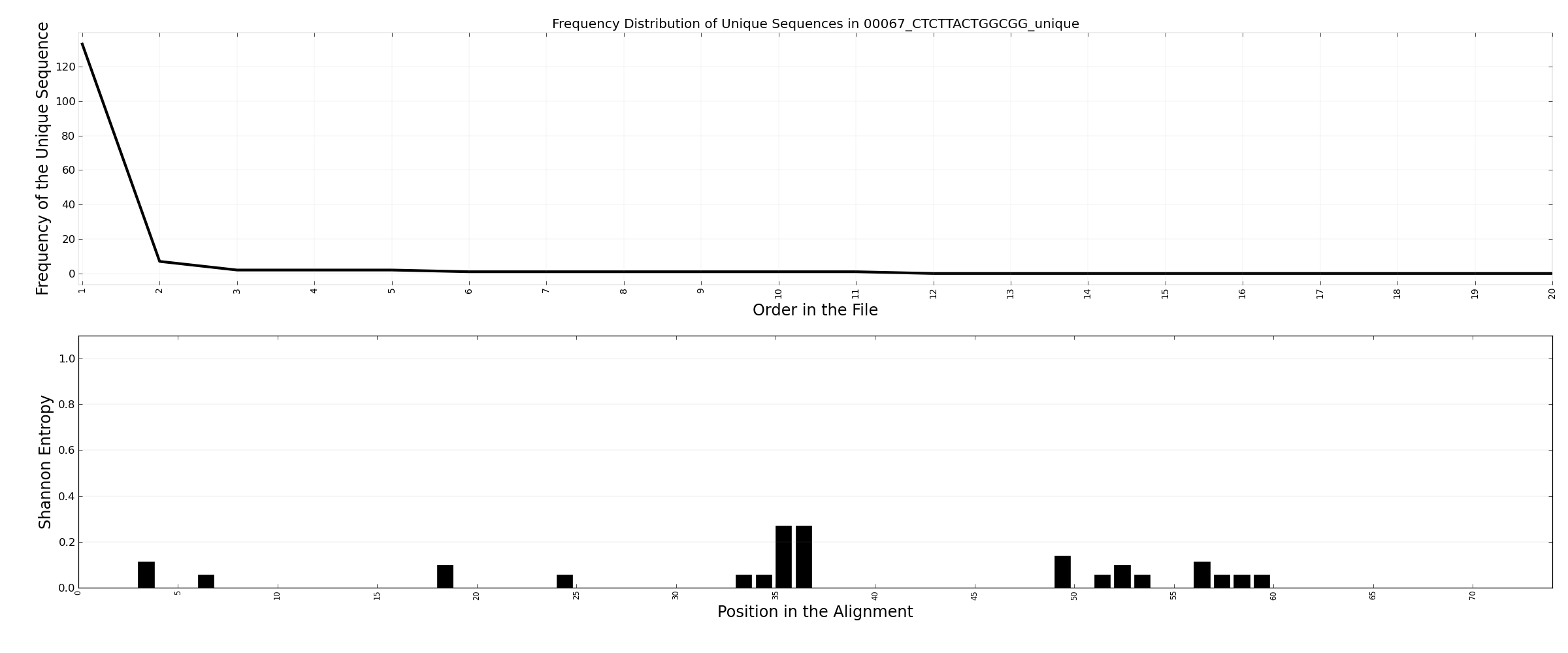

Supplement: Supplementary file 6 [file DataSheet2.ZIP › HTML-OUTPUT/00067_CTCTTACTGGCGG_unique.png]

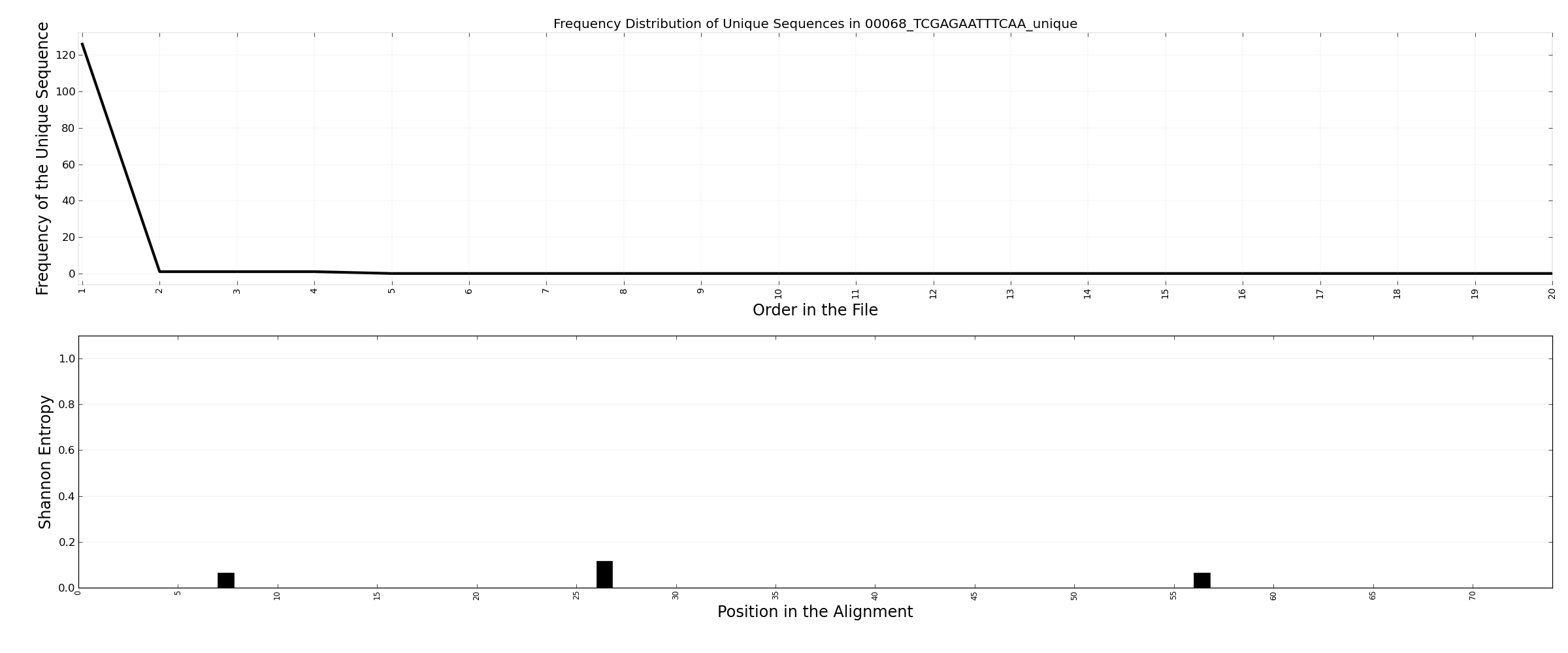

Supplement: Supplementary file 6 [file DataSheet2.ZIP › HTML-OUTPUT/00068_TCGAGAATTTCAA_unique.png]

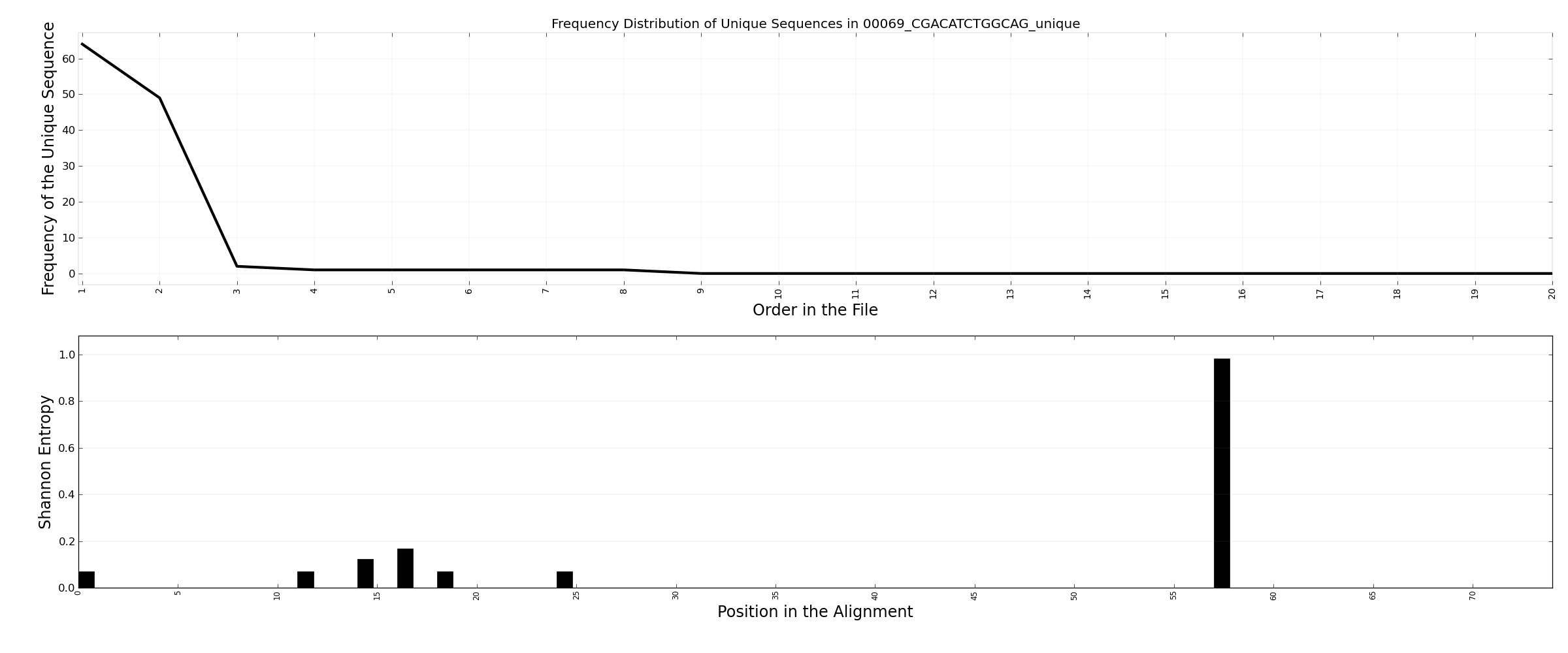

Supplement: Supplementary file 6 [file DataSheet2.ZIP › HTML-OUTPUT/00069_CGACATCTGGCAG_unique.png]

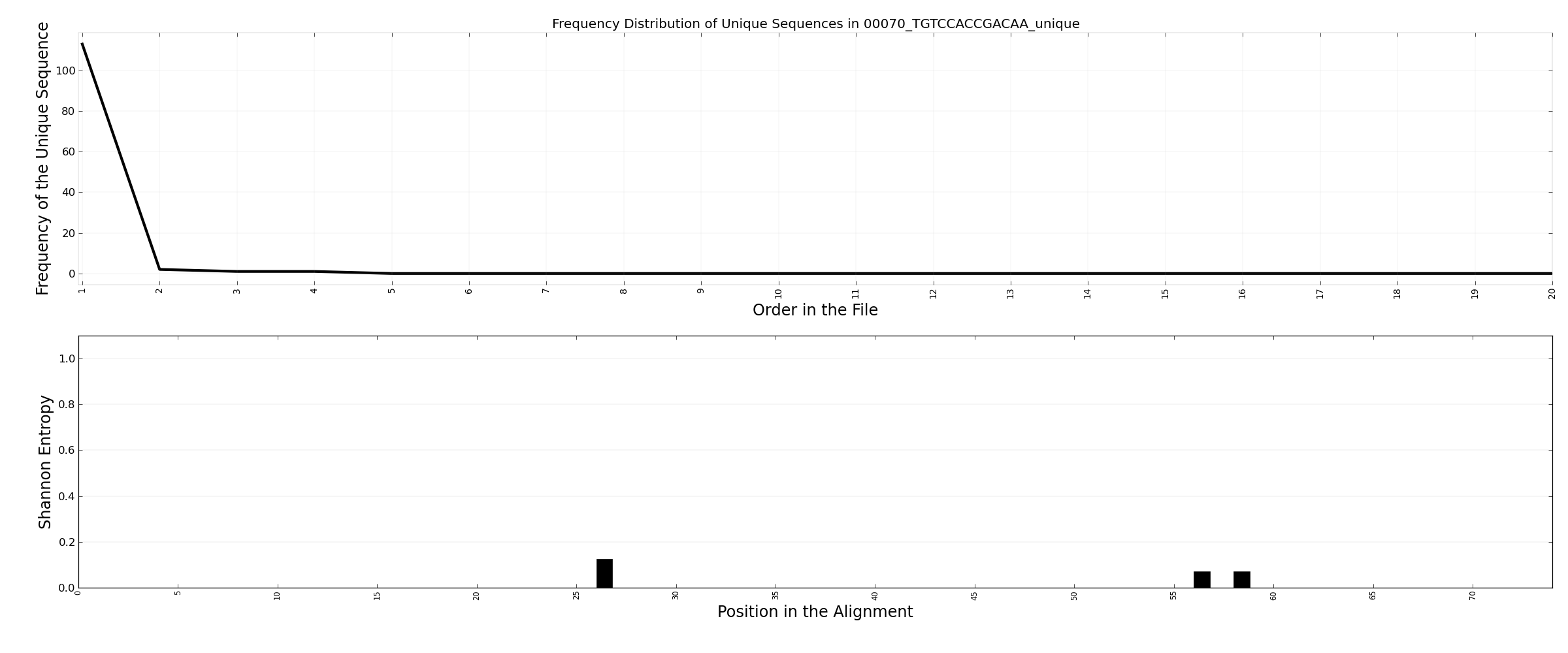

Supplement: Supplementary file 6 [file DataSheet2.ZIP › HTML-OUTPUT/00070_TGTCCACCGACAA_unique.png]

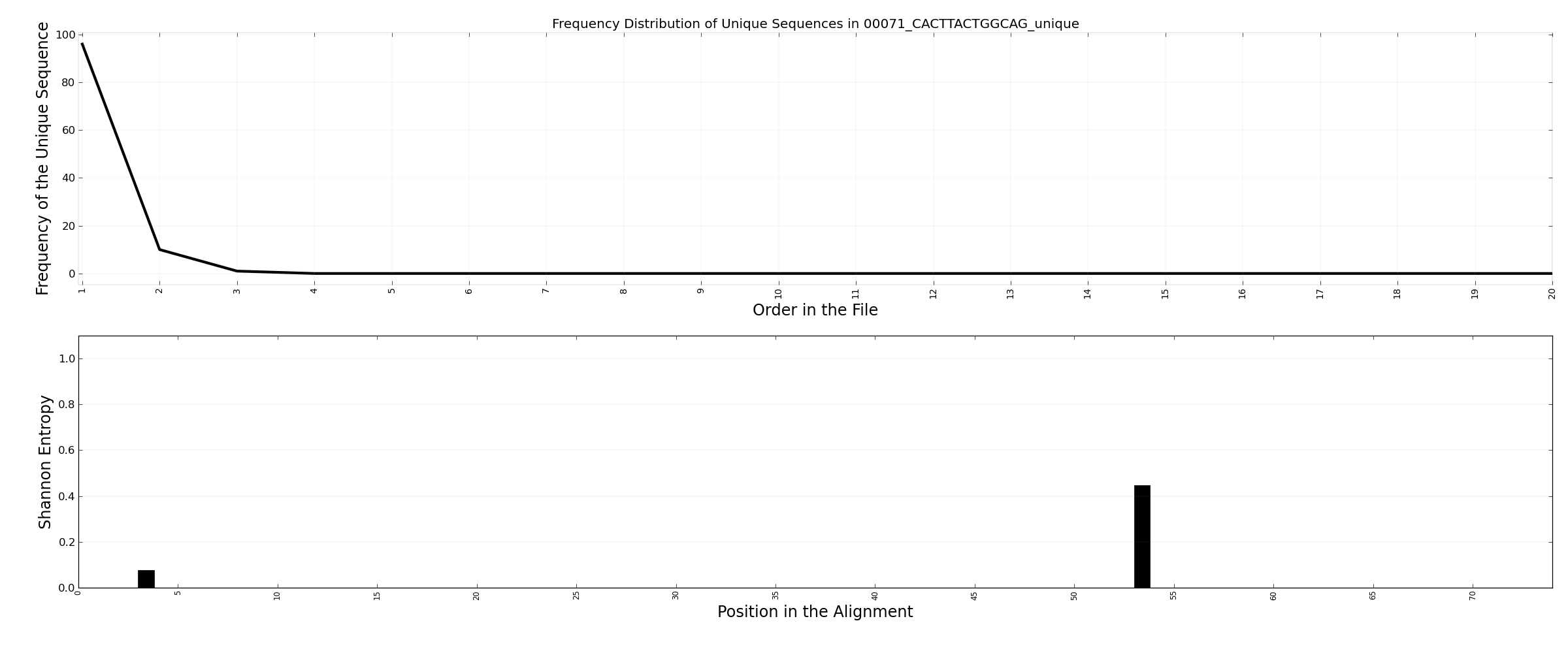

Supplement: Supplementary file 6 [file DataSheet2.ZIP › HTML-OUTPUT/00071_CACTTACTGGCAG_unique.png]

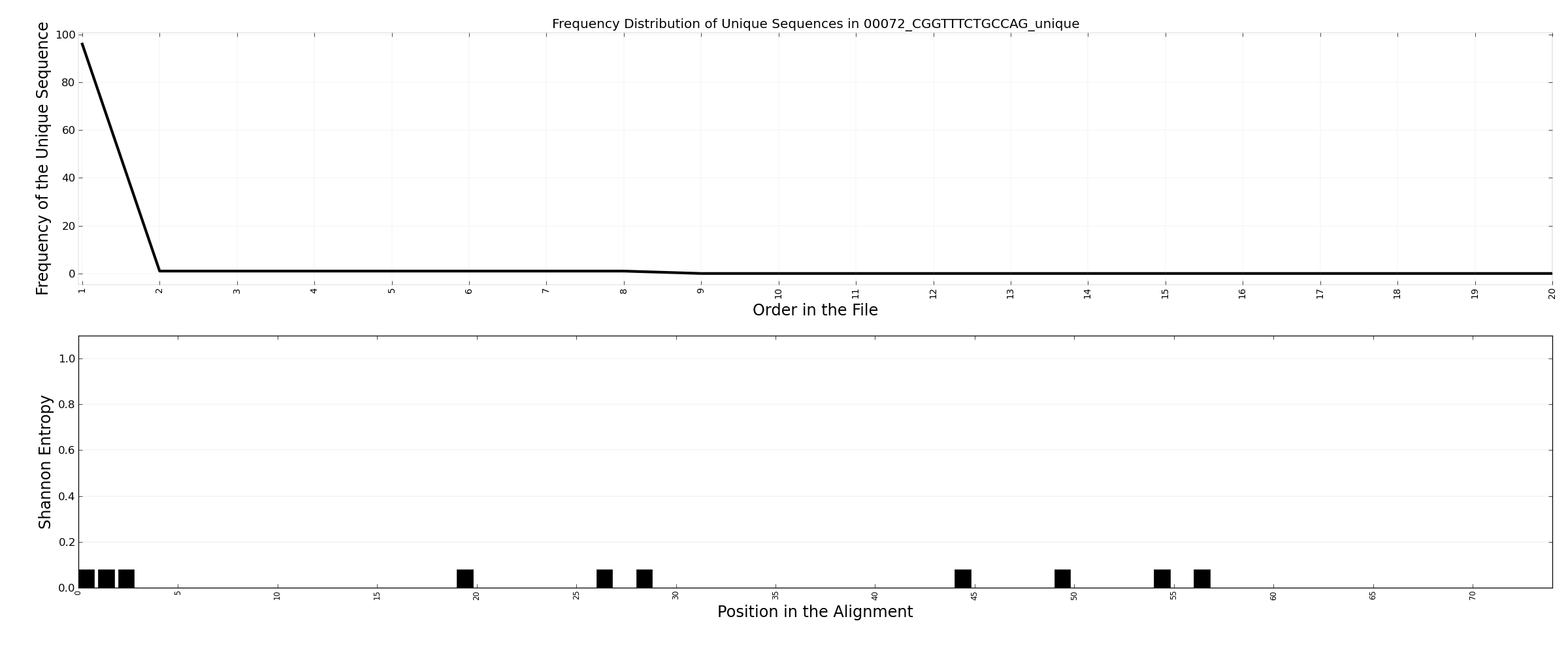

Supplement: Supplementary file 6 [file DataSheet2.ZIP › HTML-OUTPUT/00072_CGGTTTCTGCCAG_unique.png]

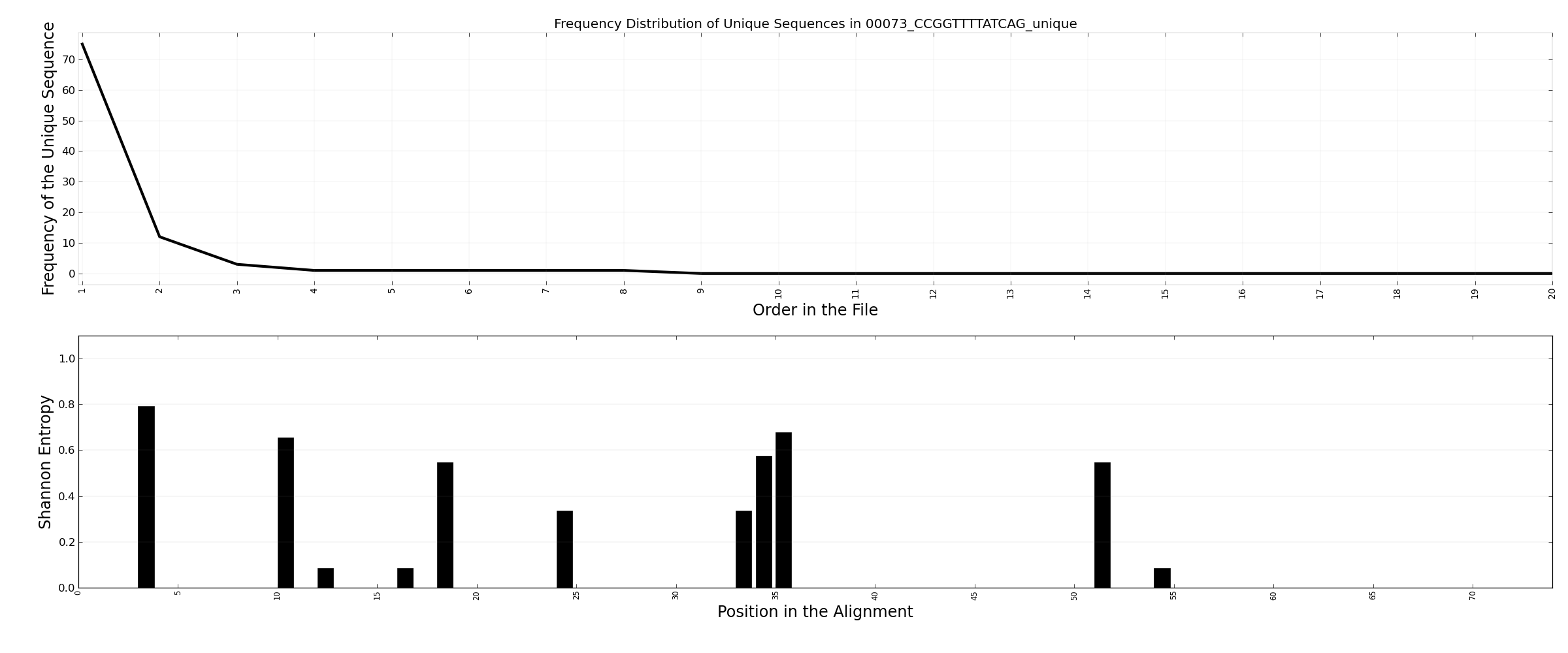

Supplement: Supplementary file 6 [file DataSheet2.ZIP › HTML-OUTPUT/00073_CCGGTTTTATCAG_unique.png]

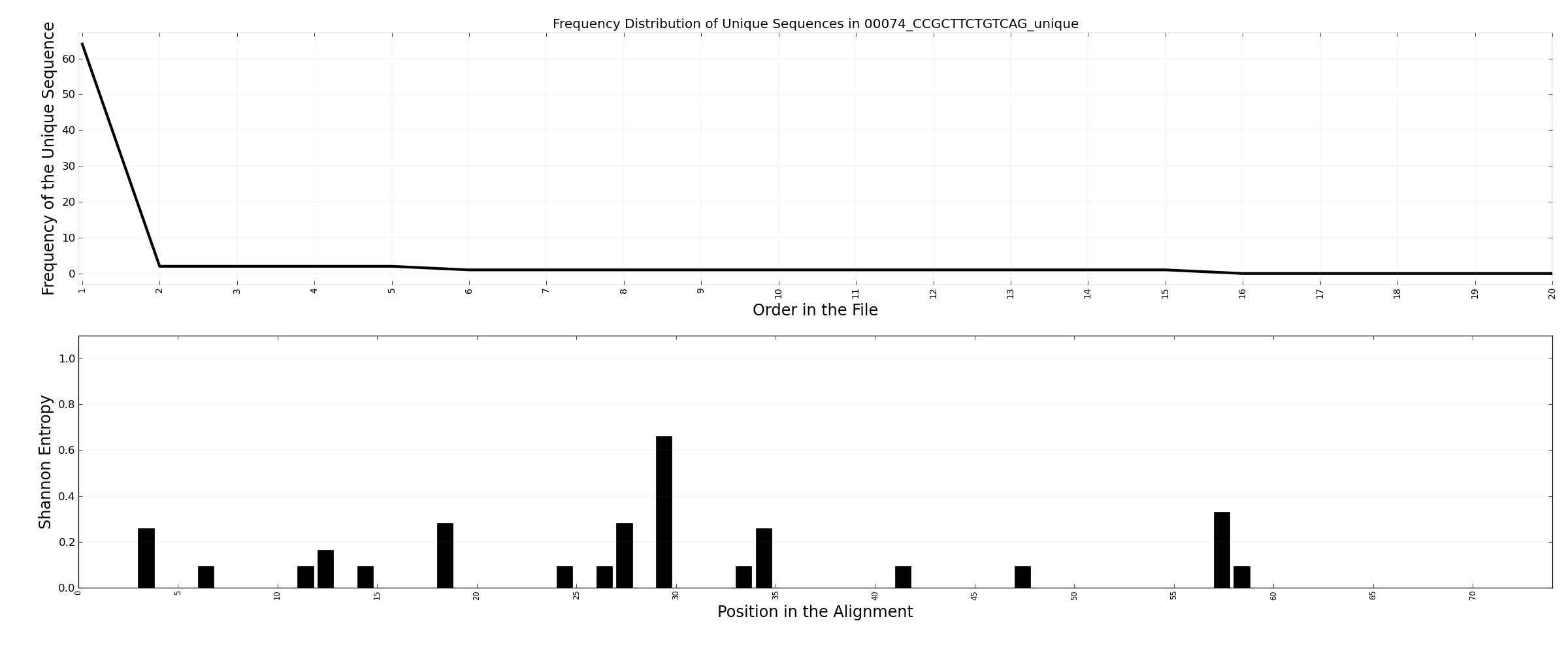

Supplement: Supplementary file 6 [file DataSheet2.ZIP › HTML-OUTPUT/00074_CCGCTTCTGTCAG_unique.png]

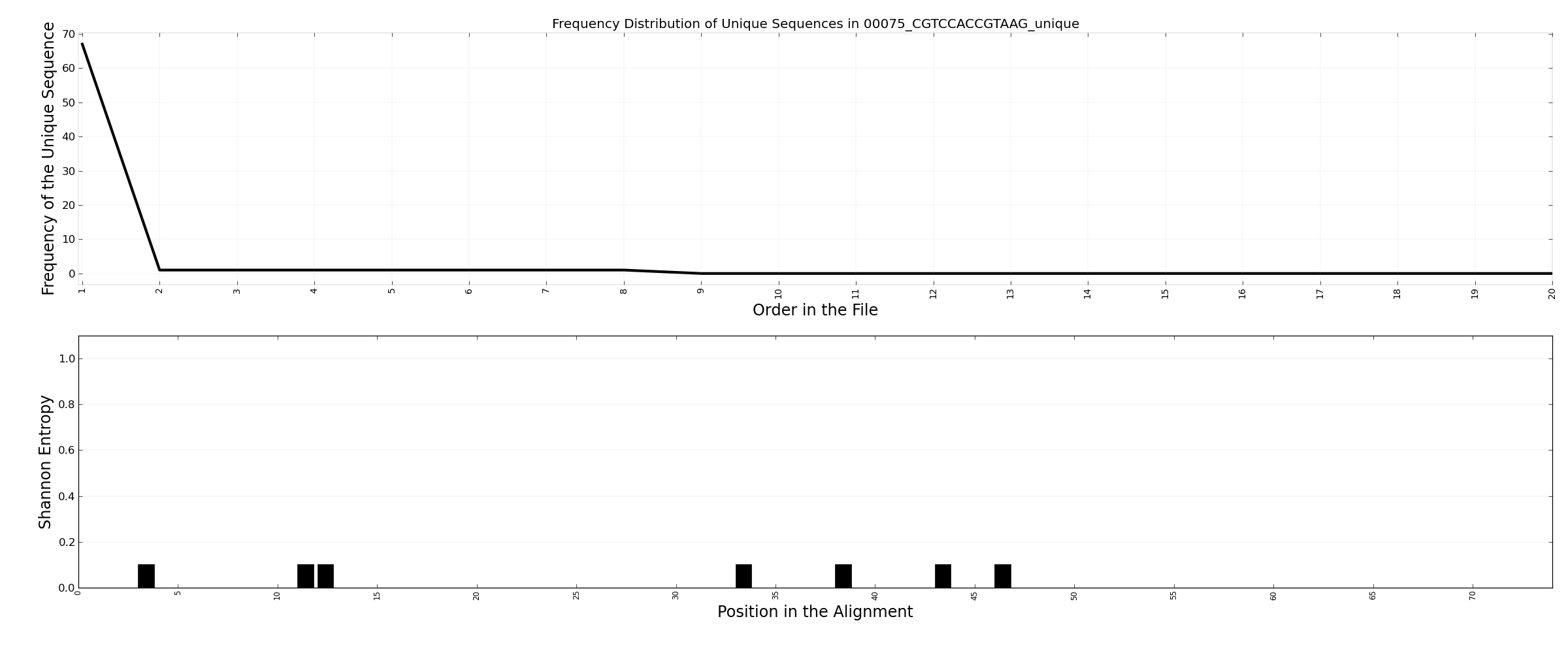

Supplement: Supplementary file 6 [file DataSheet2.ZIP › HTML-OUTPUT/00075_CGTCCACCGTAAG_unique.png]

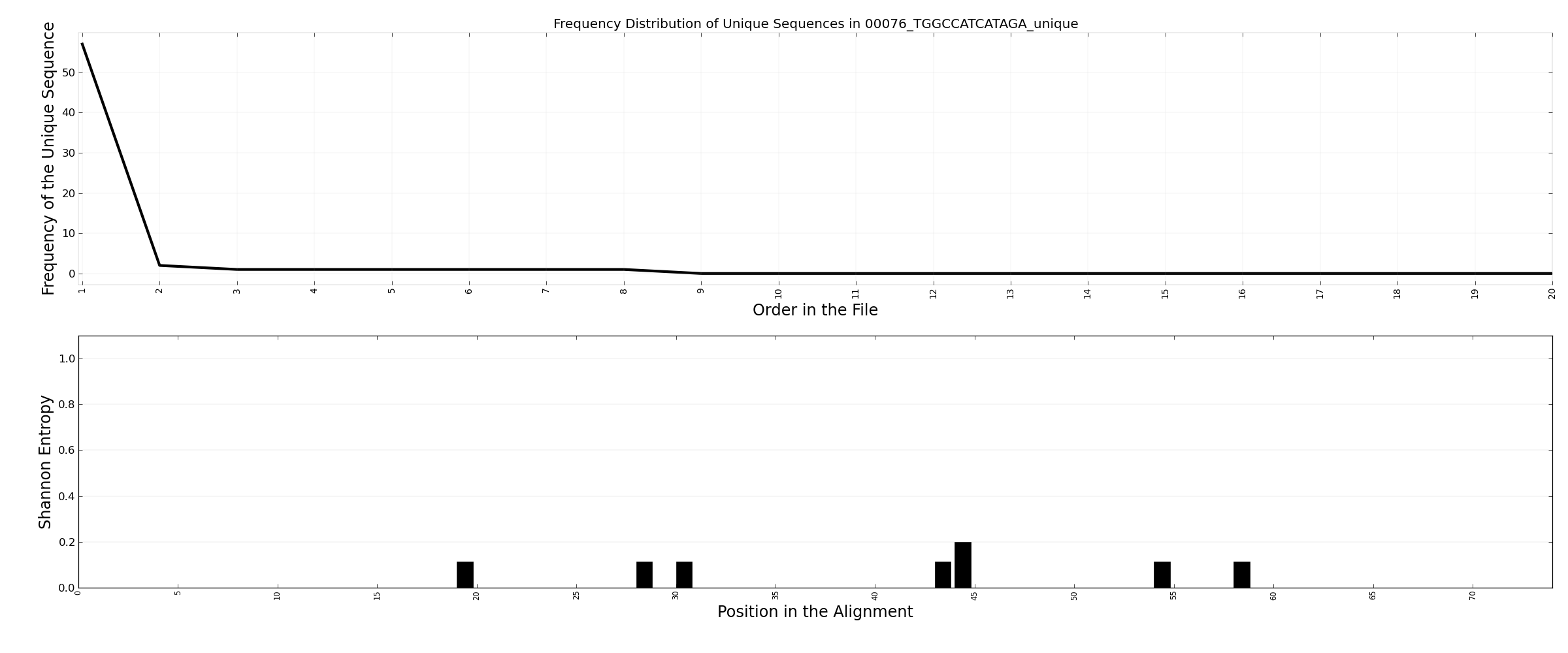

Supplement: Supplementary file 6 [file DataSheet2.ZIP › HTML-OUTPUT/00076_TGGCCATCATAGA_unique.png]

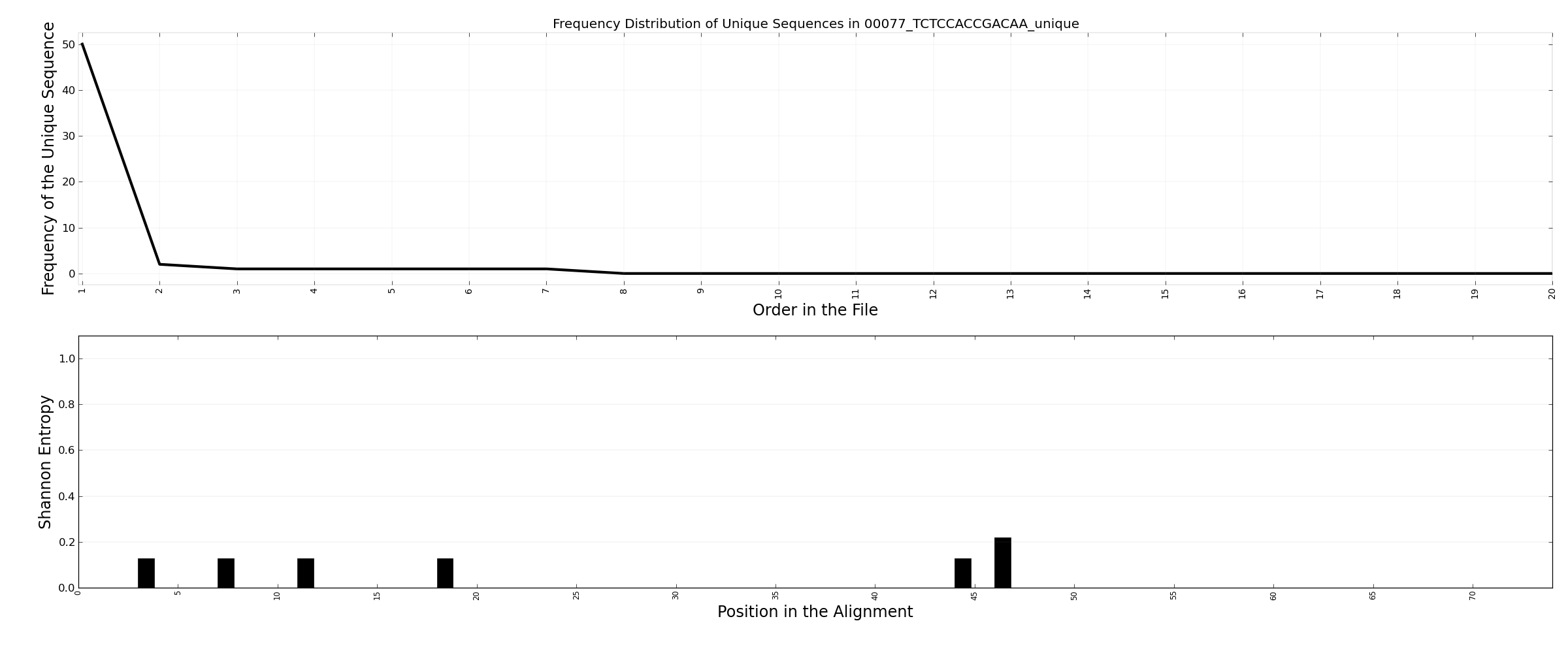

Supplement: Supplementary file 6 [file DataSheet2.ZIP › HTML-OUTPUT/00077_TCTCCACCGACAA_unique.png]

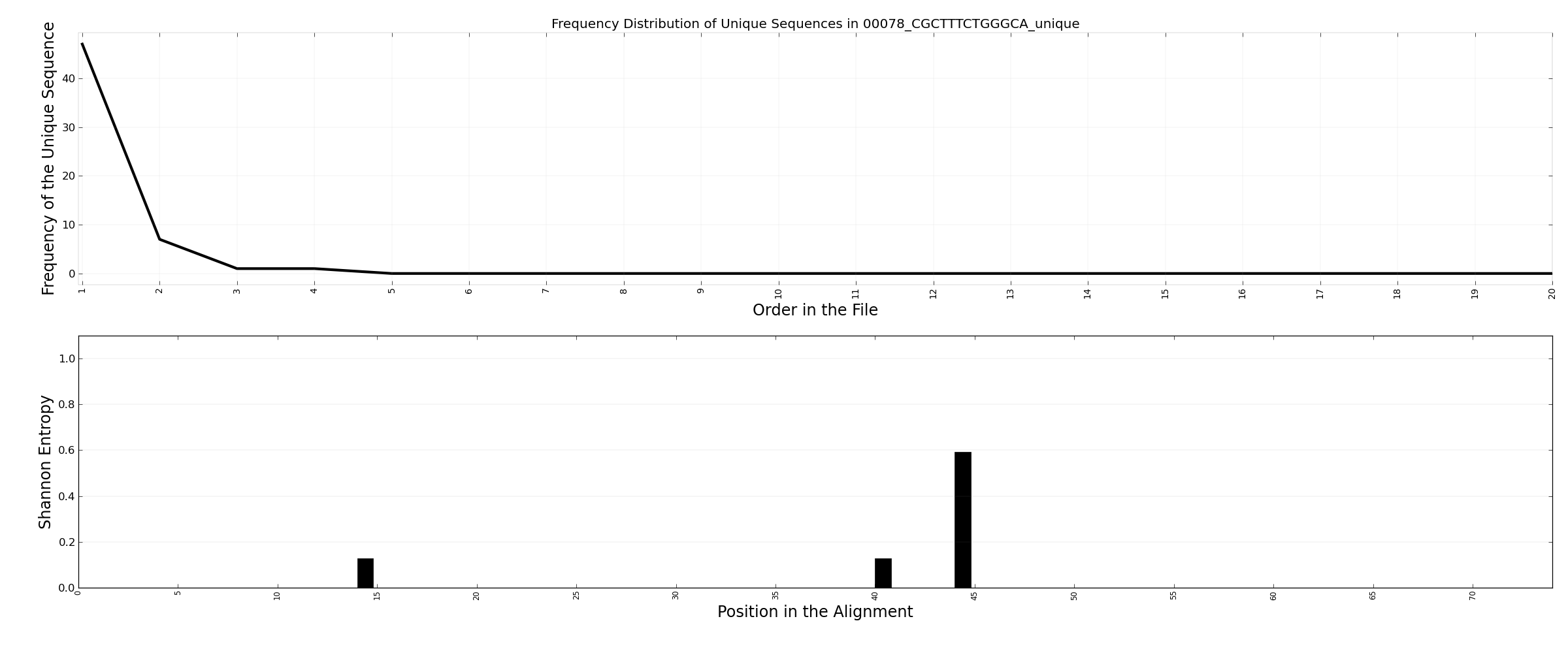

Supplement: Supplementary file 6 [file DataSheet2.ZIP › HTML-OUTPUT/00078_CGCTTTCTGGGCA_unique.png]

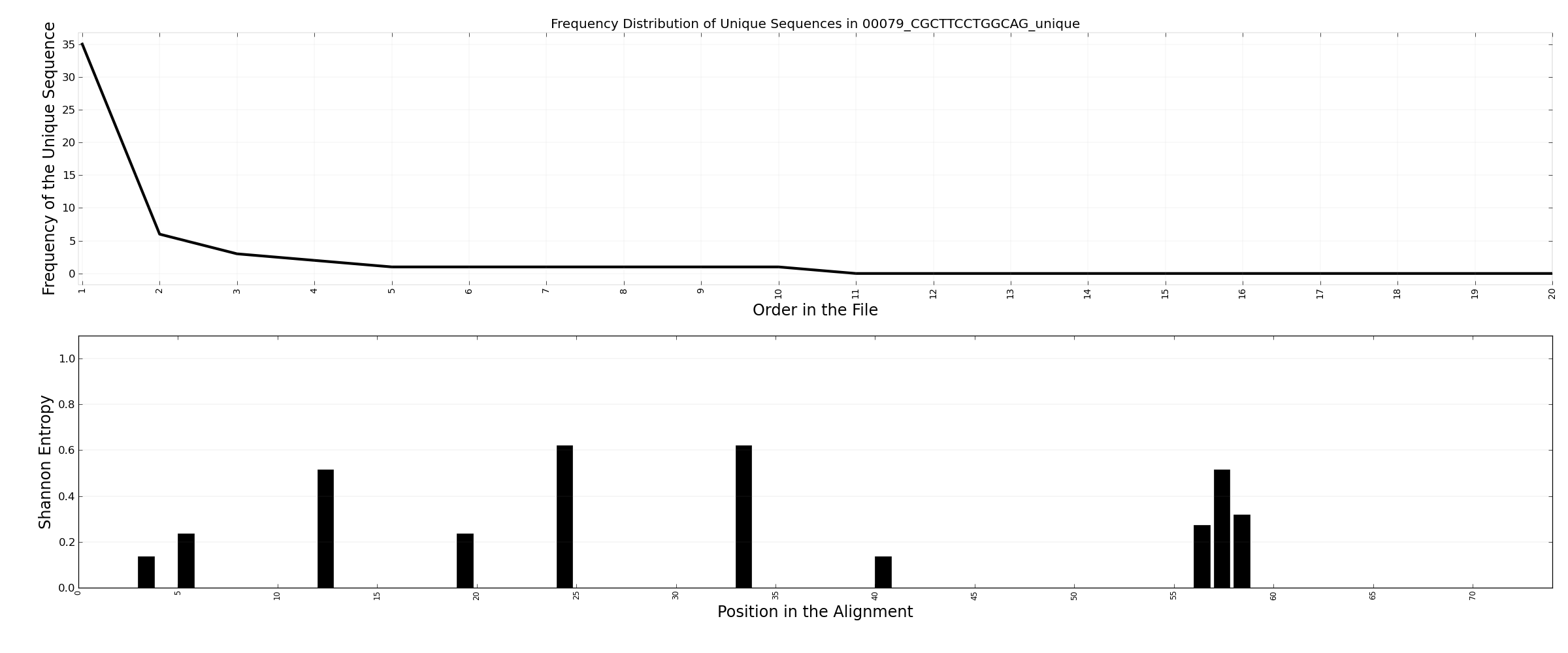

Supplement: Supplementary file 6 [file DataSheet2.ZIP › HTML-OUTPUT/00079_CGCTTCCTGGCAG_unique.png]

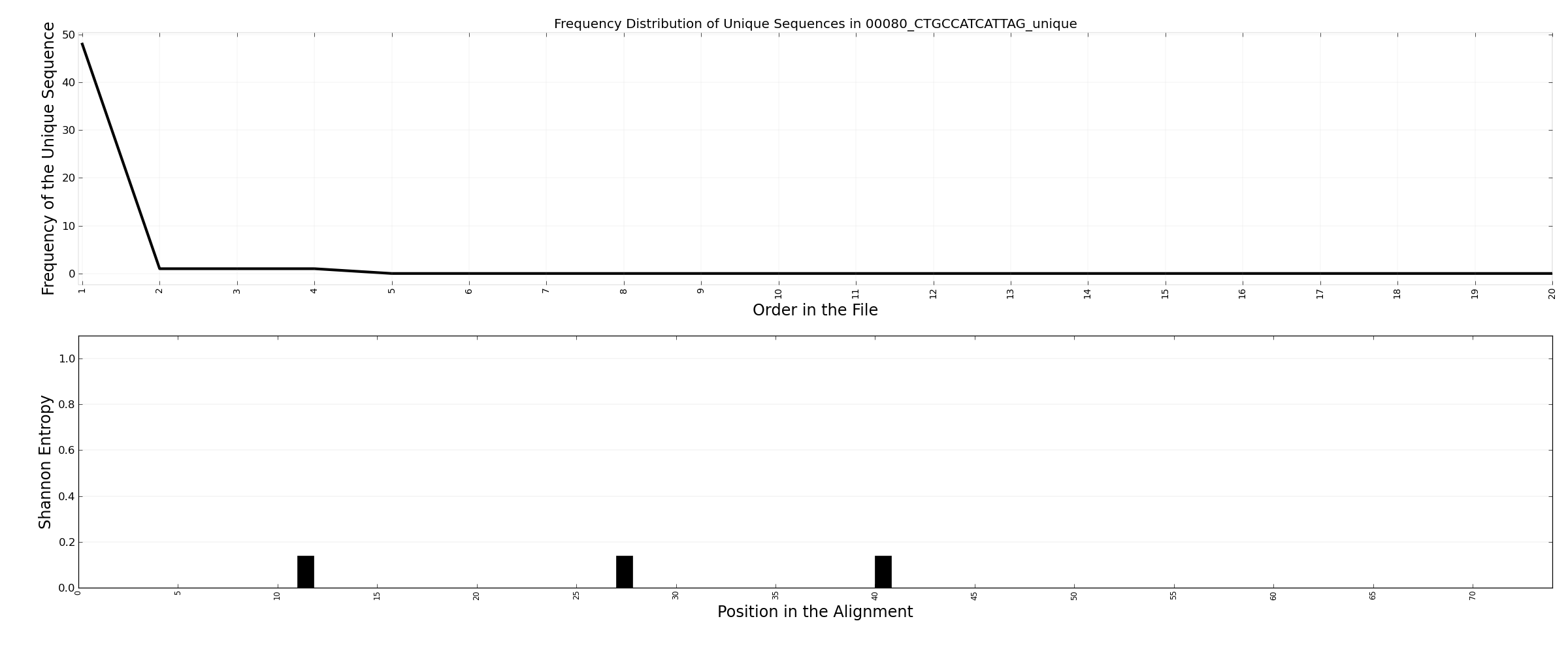

Supplement: Supplementary file 6 [file DataSheet2.ZIP › HTML-OUTPUT/00080_CTGCCATCATTAG_unique.png]

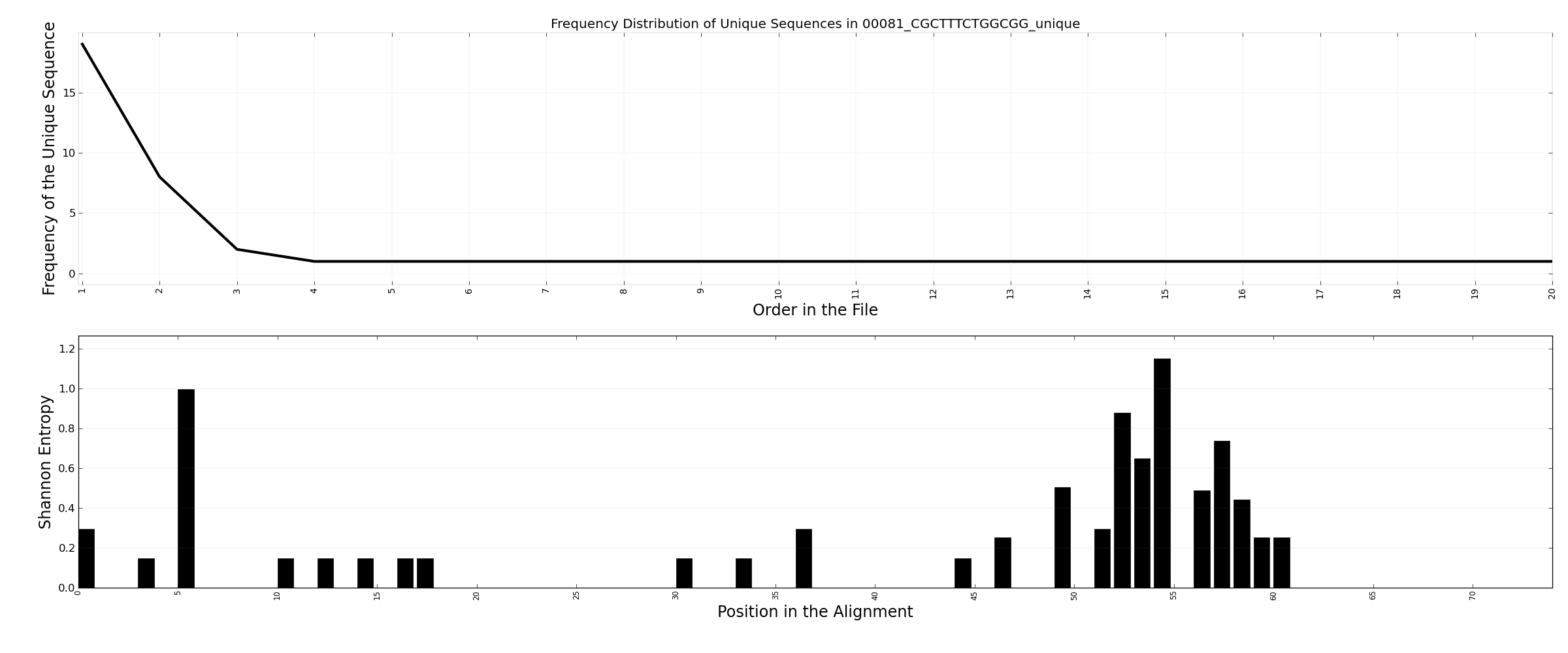

Supplement: Supplementary file 6 [file DataSheet2.ZIP › HTML-OUTPUT/00081_CGCTTTCTGGCGG_unique.png]

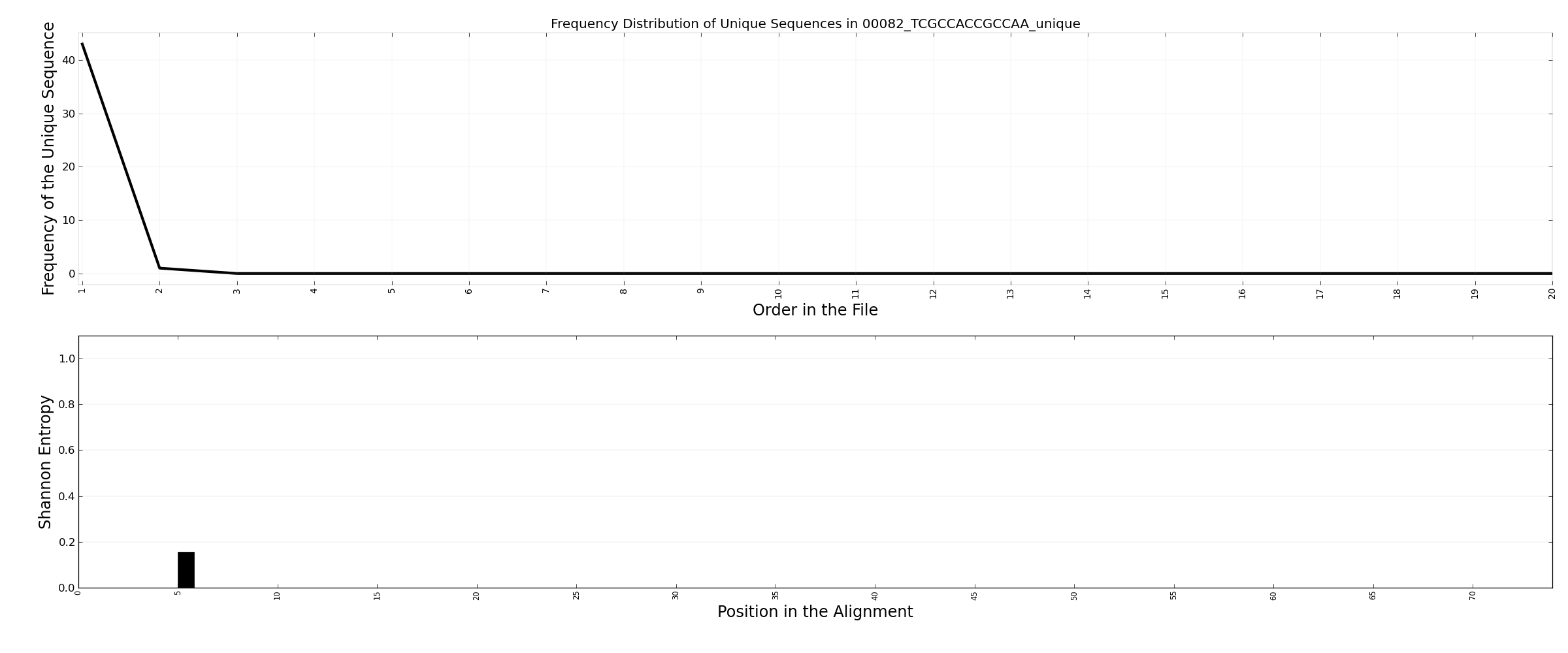

Supplement: Supplementary file 6 [file DataSheet2.ZIP › HTML-OUTPUT/00082_TCGCCACCGCCAA_unique.png]

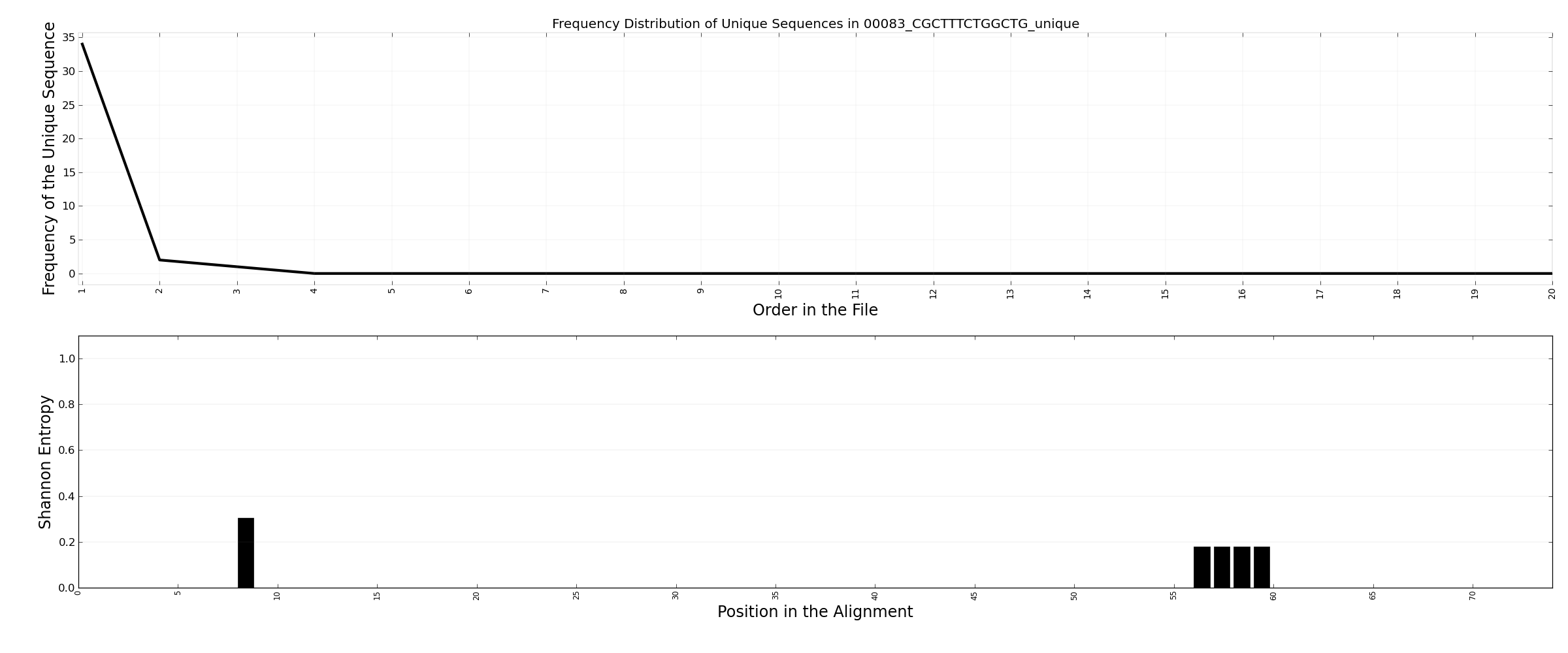

Supplement: Supplementary file 6 [file DataSheet2.ZIP › HTML-OUTPUT/00083_CGCTTTCTGGCTG_unique.png]

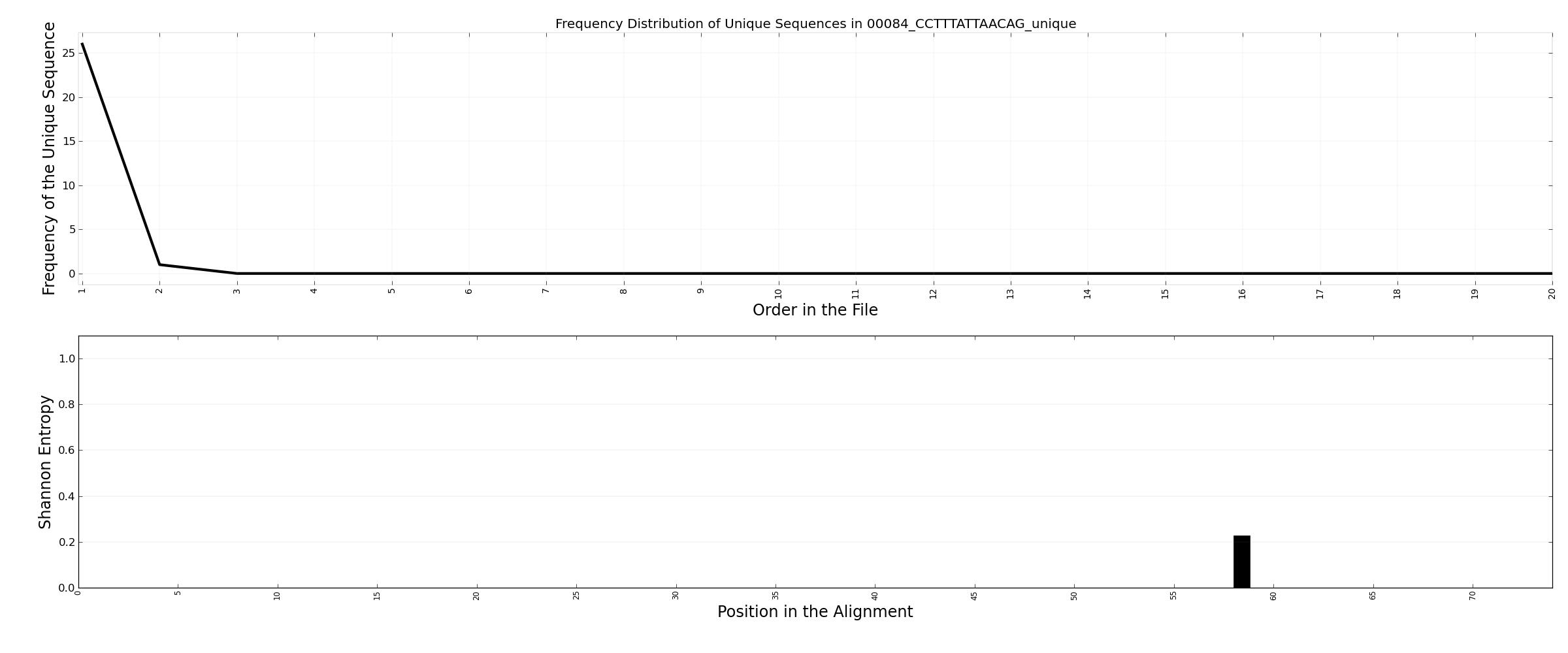

Supplement: Supplementary file 6 [file DataSheet2.ZIP › HTML-OUTPUT/00084_CCTTTATTAACAG_unique.png]

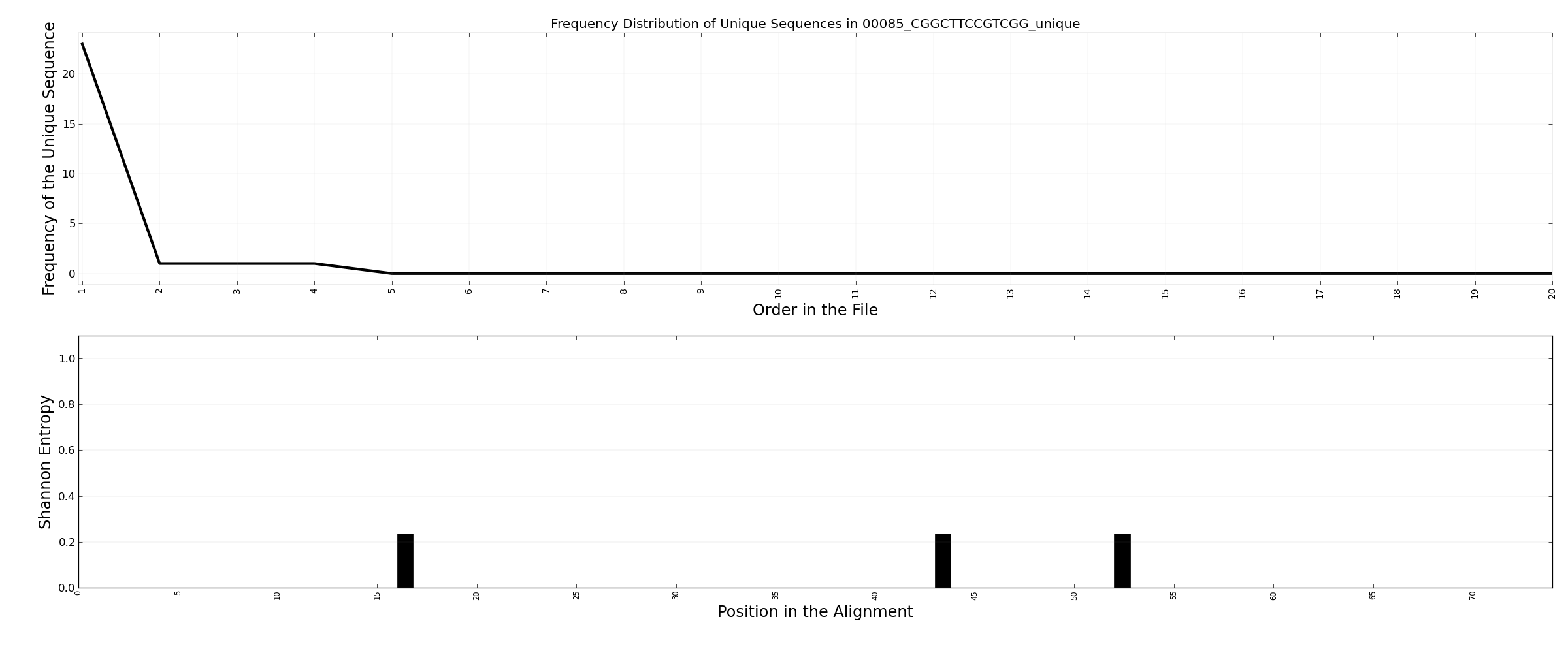

Supplement: Supplementary file 6 [file DataSheet2.ZIP › HTML-OUTPUT/00085_CGGCTTCCGTCGG_unique.png]

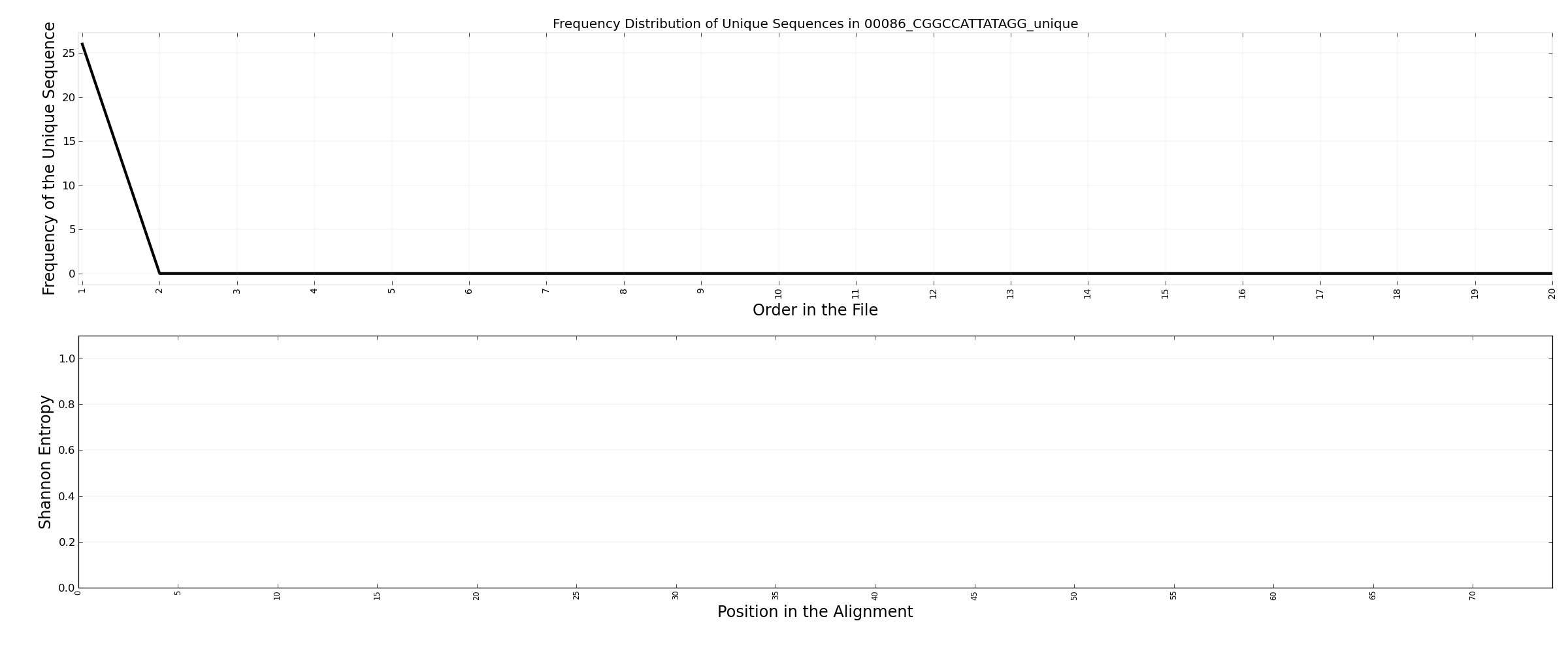

Supplement: Supplementary file 6 [file DataSheet2.ZIP › HTML-OUTPUT/00086_CGGCCATTATAGG_unique.png]

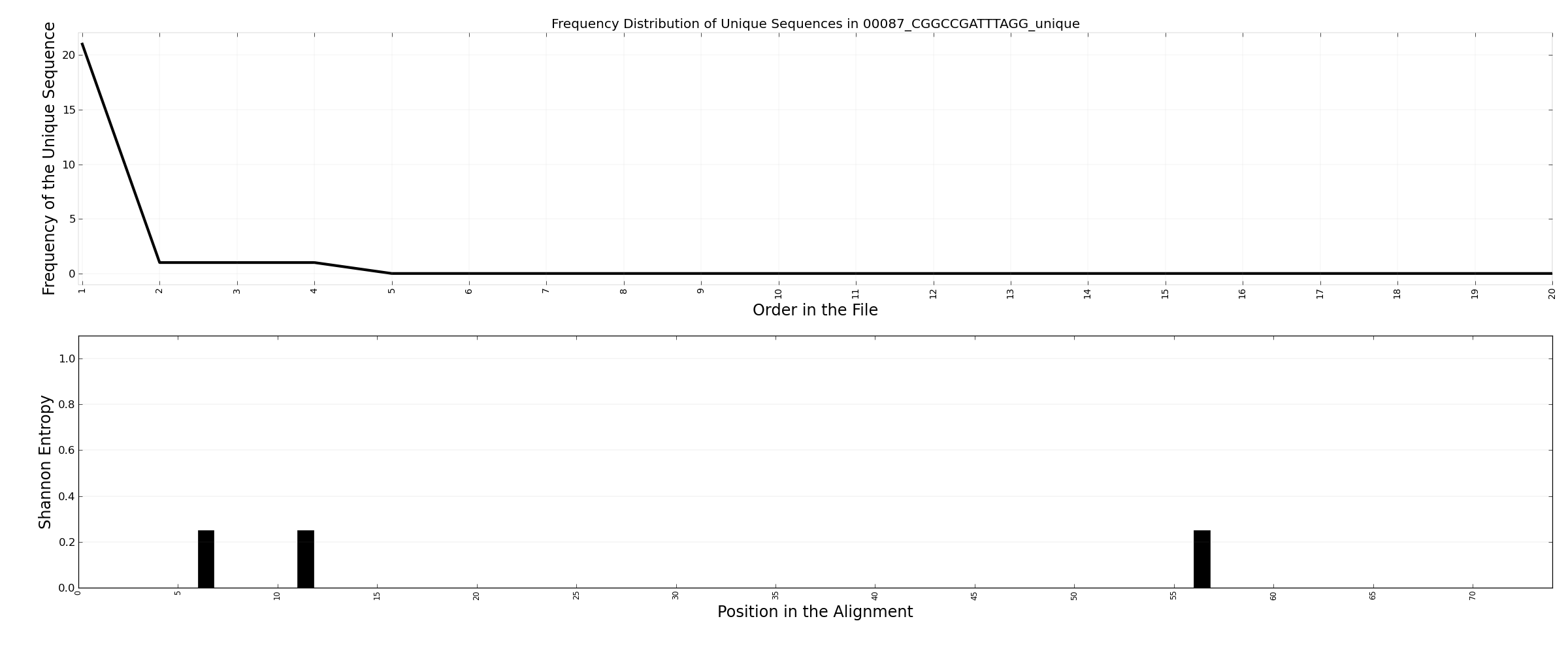

Supplement: Supplementary file 6 [file DataSheet2.ZIP › HTML-OUTPUT/00087_CGGCCGATTTAGG_unique.png]

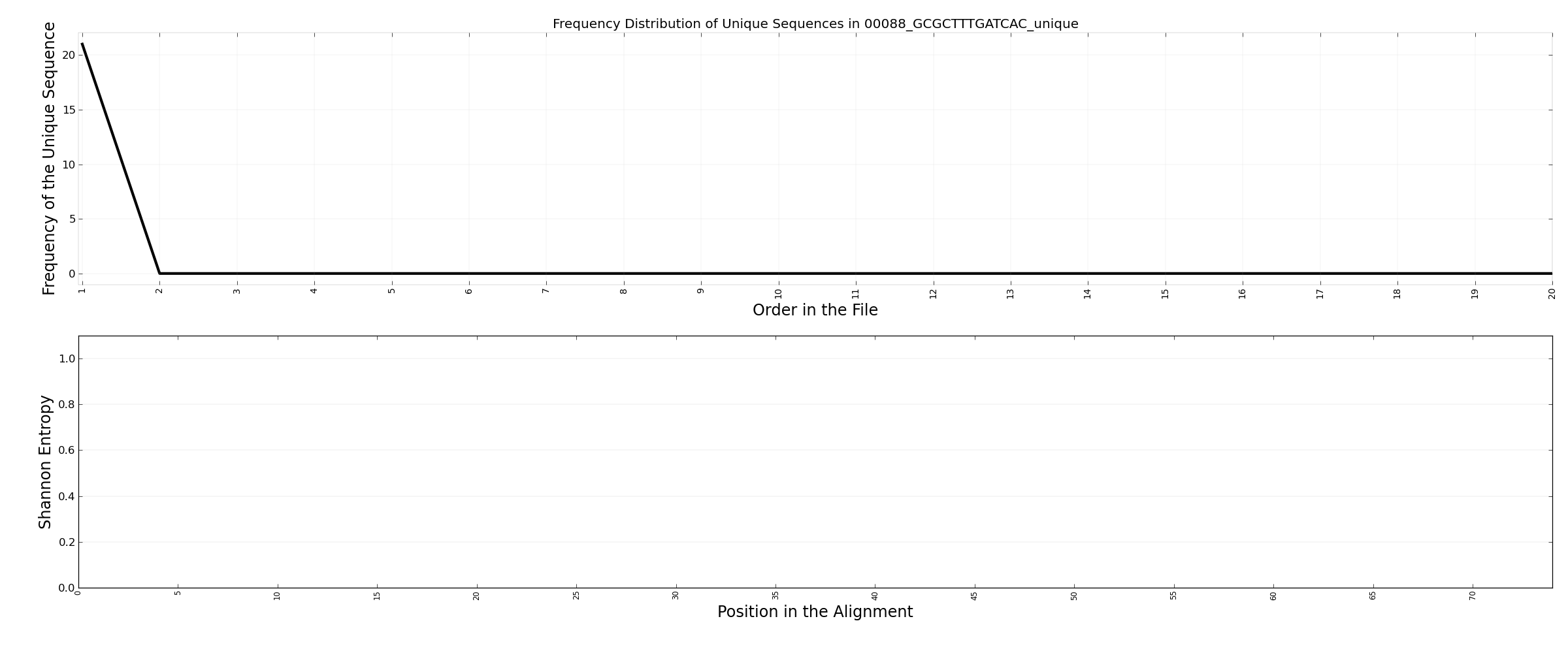

Supplement: Supplementary file 6 [file DataSheet2.ZIP › HTML-OUTPUT/00088_GCGCTTTGATCAC_unique.png]

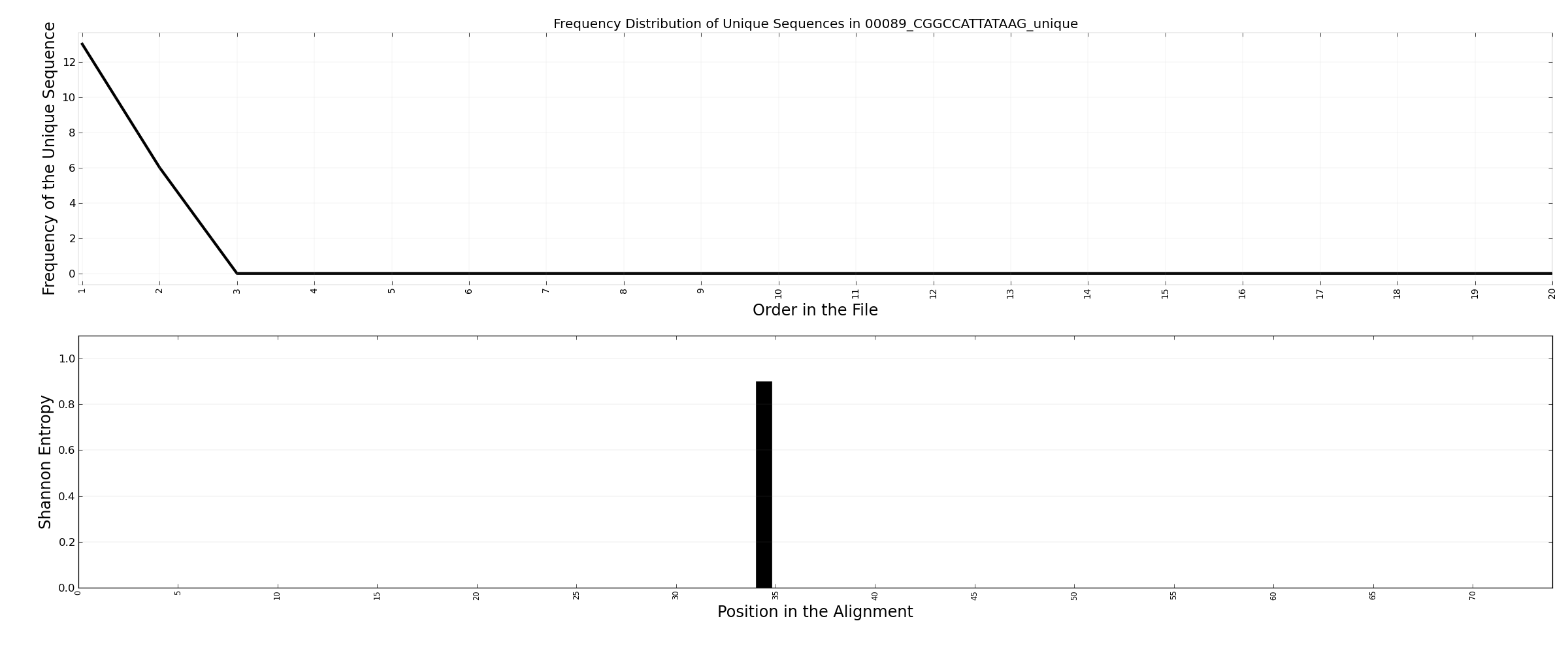

Supplement: Supplementary file 6 [file DataSheet2.ZIP › HTML-OUTPUT/00089_CGGCCATTATAAG_unique.png]

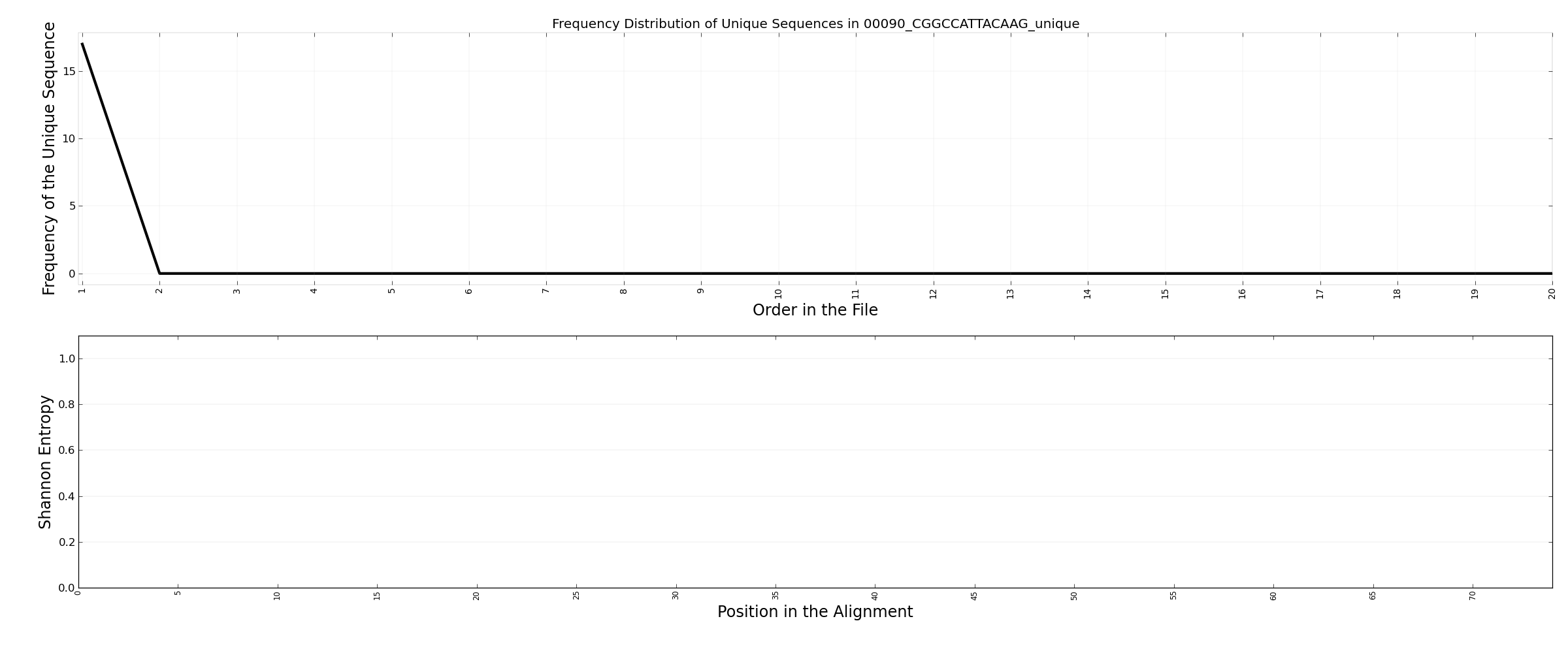

Supplement: Supplementary file 6 [file DataSheet2.ZIP › HTML-OUTPUT/00090_CGGCCATTACAAG_unique.png]

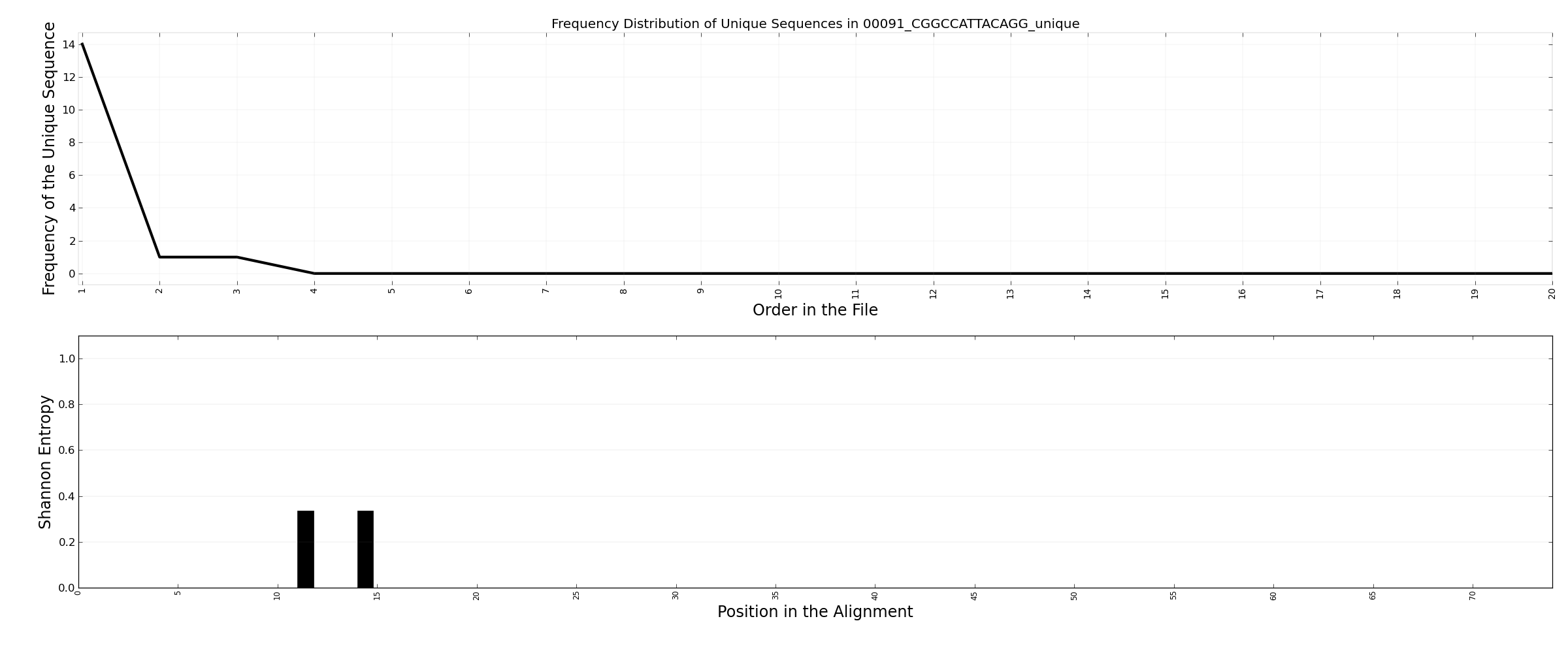

Supplement: Supplementary file 6 [file DataSheet2.ZIP › HTML-OUTPUT/00091_CGGCCATTACAGG_unique.png]

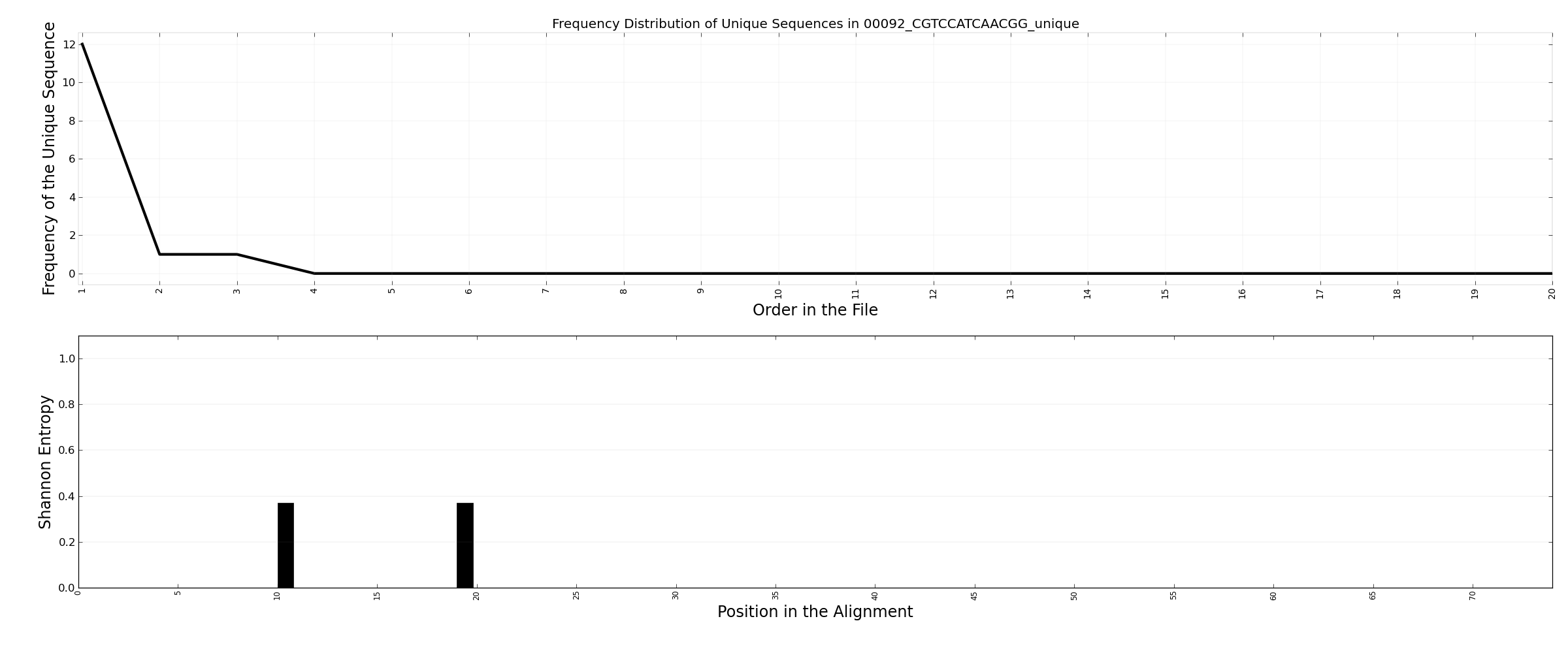

Supplement: Supplementary file 6 [file DataSheet2.ZIP › HTML-OUTPUT/00092_CGTCCATCAACGG_unique.png]

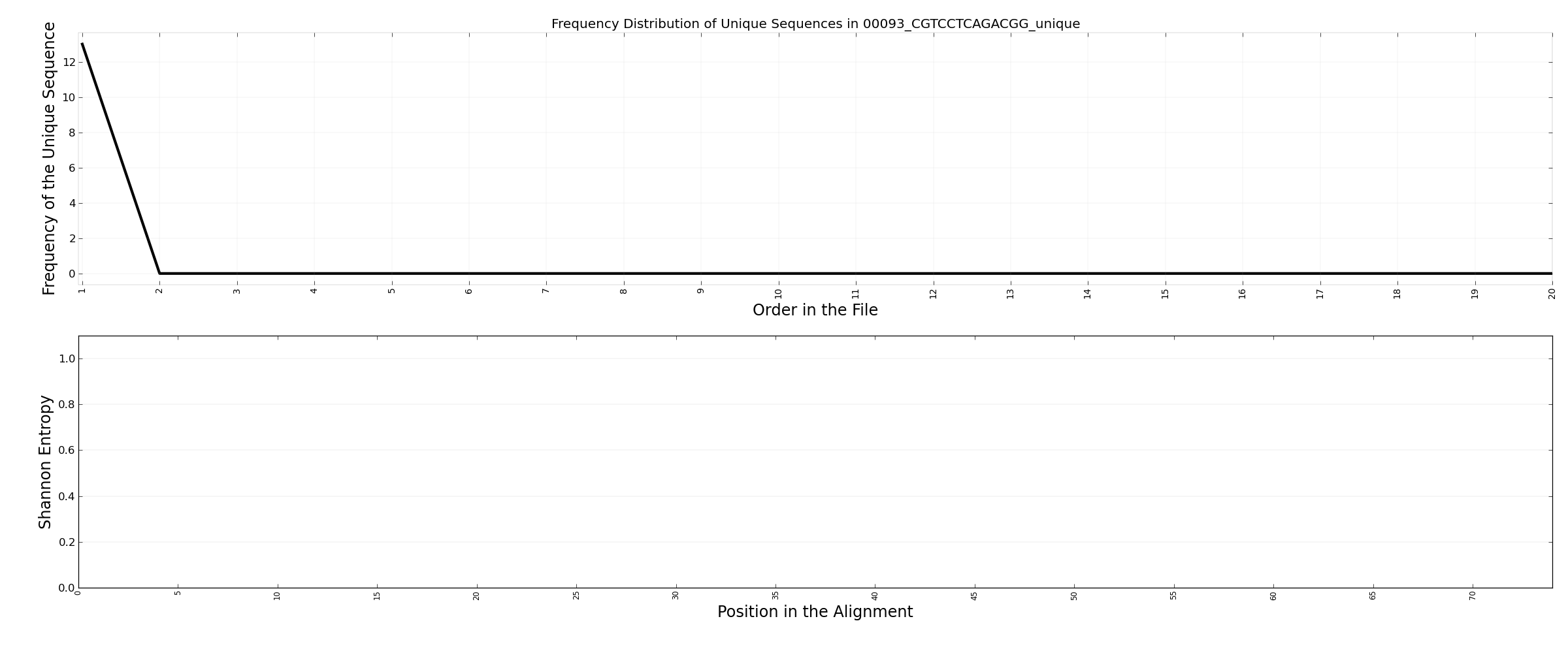

Supplement: Supplementary file 6 [file DataSheet2.ZIP › HTML-OUTPUT/00093_CGTCCTCAGACGG_unique.png]

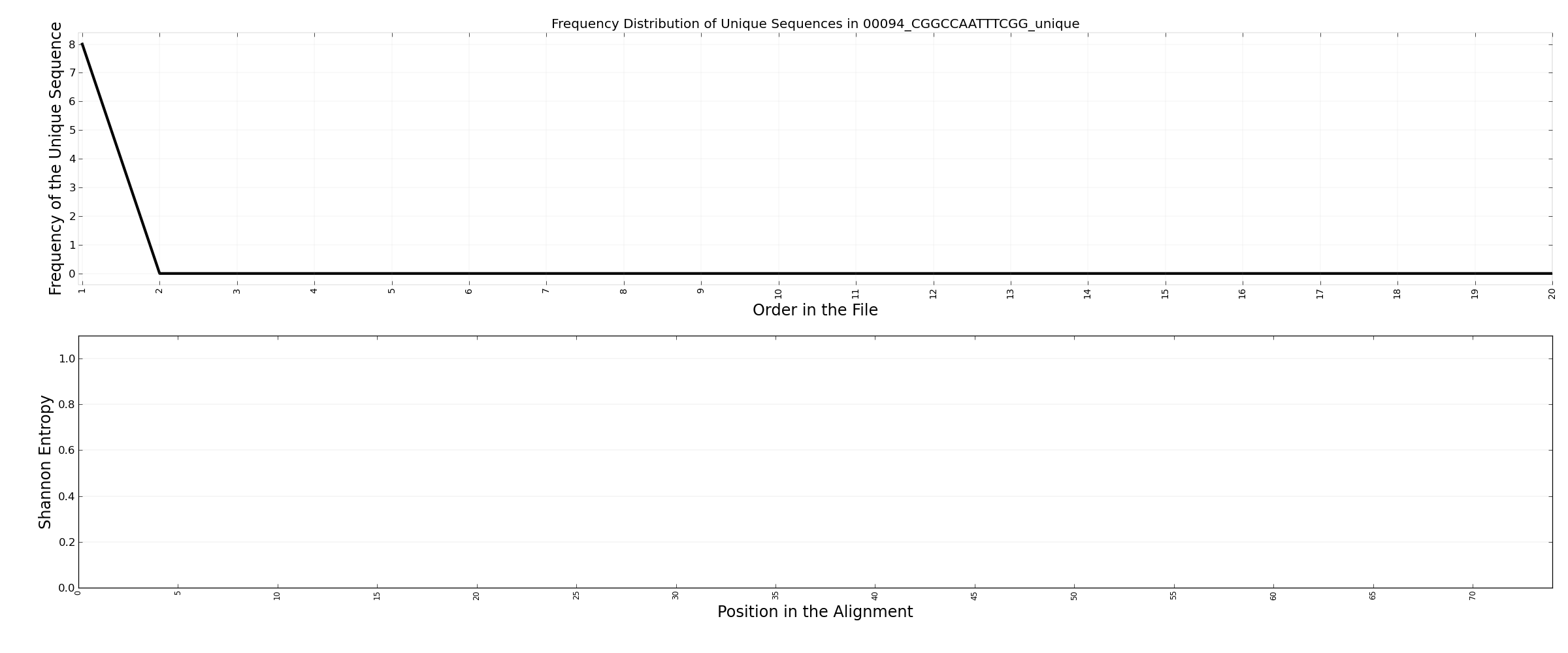

Supplement: Supplementary file 6 [file DataSheet2.ZIP › HTML-OUTPUT/00094_CGGCCAATTTCGG_unique.png]

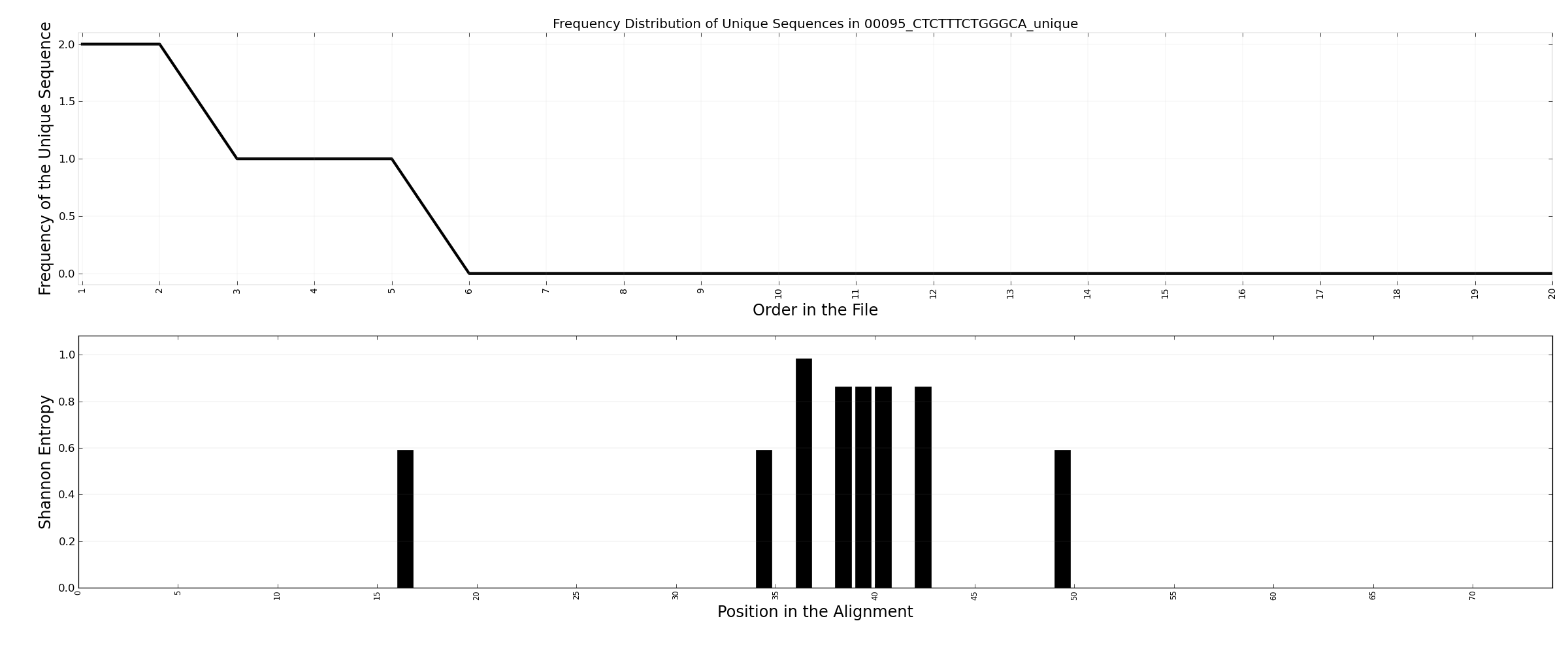

Supplement: Supplementary file 6 [file DataSheet2.ZIP › HTML-OUTPUT/00095_CTCTTTCTGGGCA_unique.png]

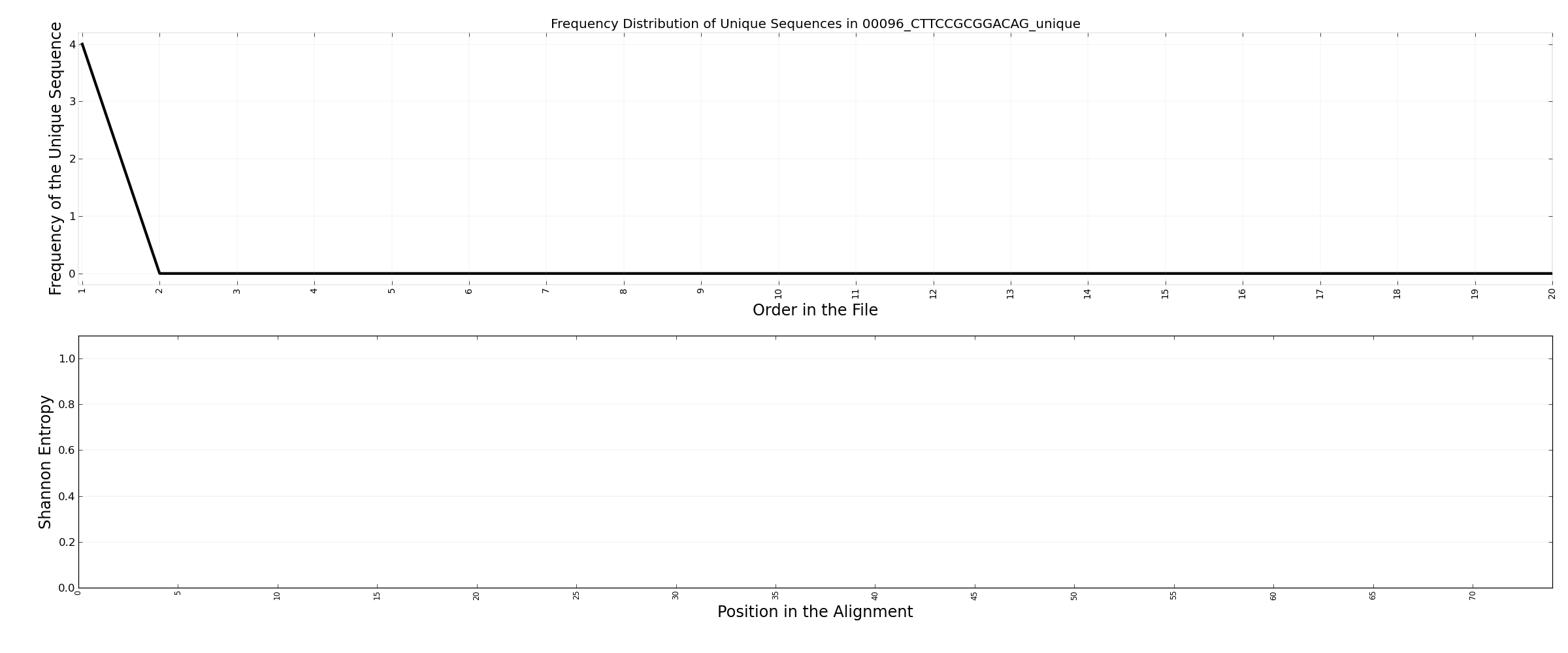

Supplement: Supplementary file 6 [file DataSheet2.ZIP › HTML-OUTPUT/00096_CTTCCGCGGACAG_unique.png]

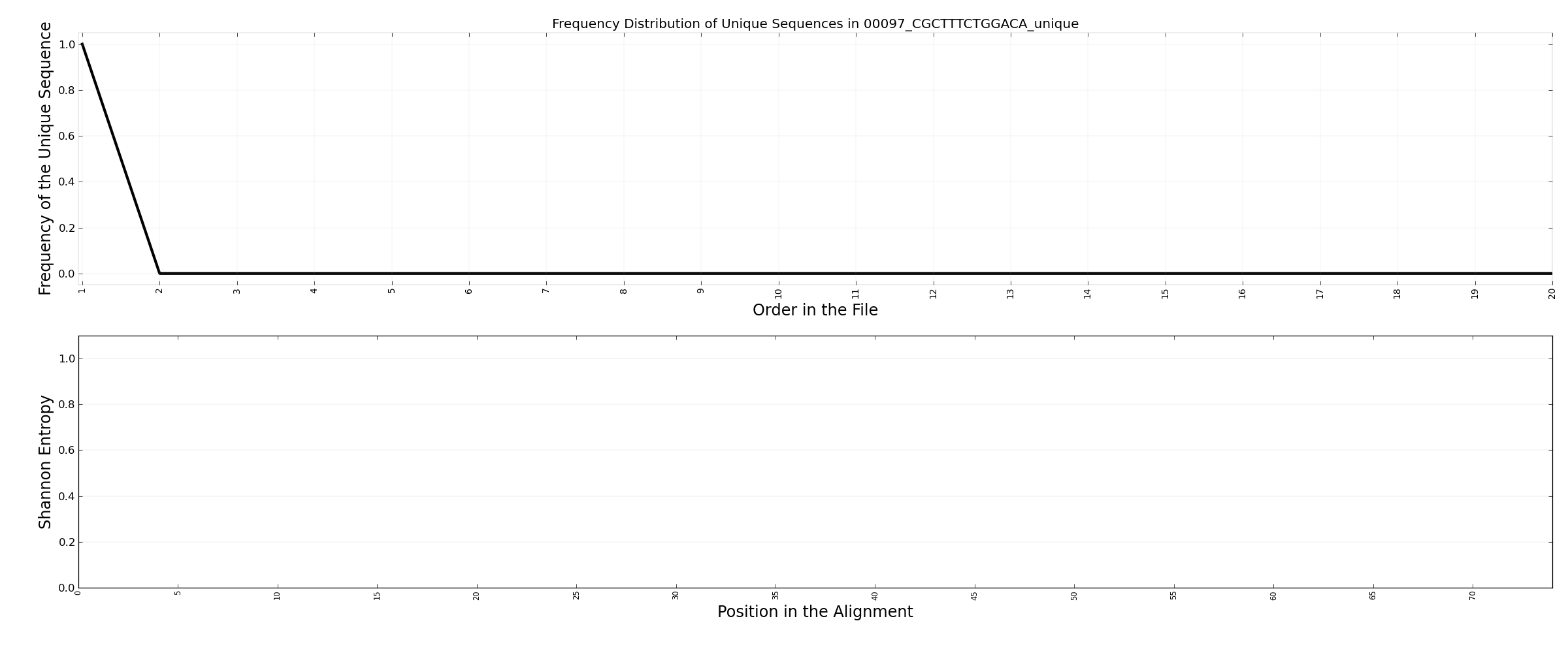

Supplement: Supplementary file 6 [file DataSheet2.ZIP › HTML-OUTPUT/00097_CGCTTTCTGGACA_unique.png]

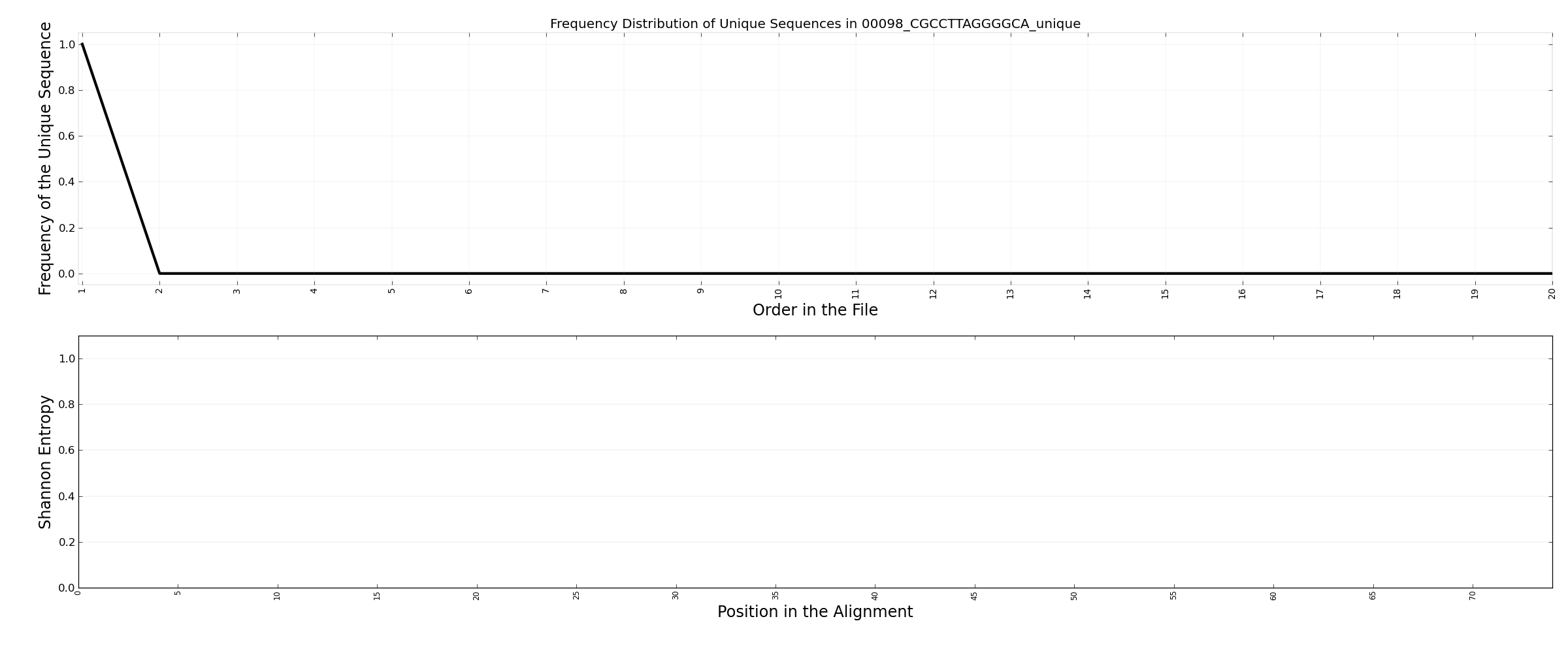

Supplement: Supplementary file 6 [file DataSheet2.ZIP › HTML-OUTPUT/00098_CGCCTTAGGGGCA_unique.png]

## noaquifer-PADDED-WITH-GAPS

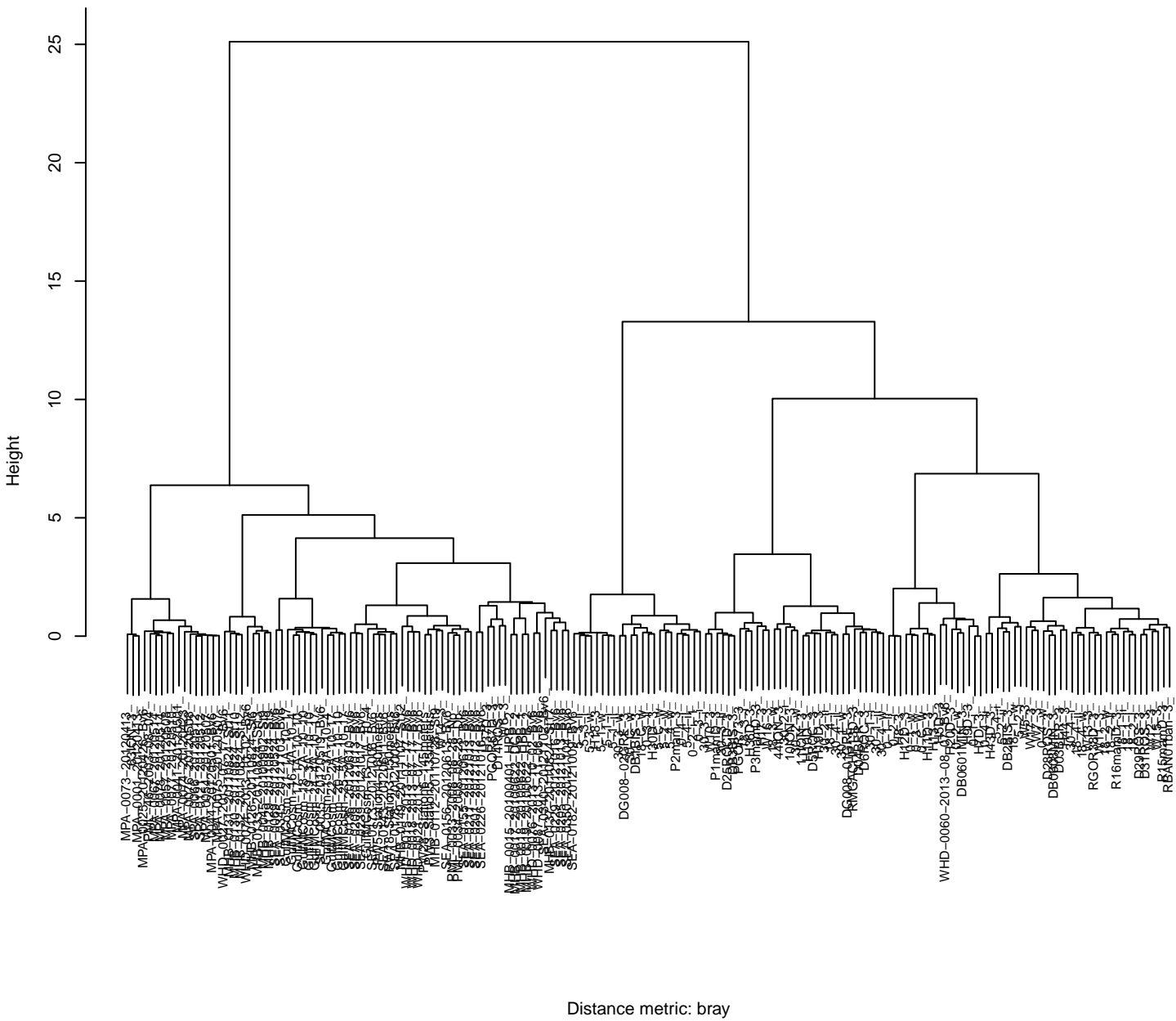

Supplement: Supplementary file 6 [file DataSheet2.ZIP › HTML-OUTPUT/basic_analyses-cluster_analysis-bray.pdf]
