# Supplementary material for: Oligotyping reveals community level habitat selection within the genus Vibrio
Source: Front Microbiol. 2014 Nov 13;5:563. doi: 10.3389/fmicb.2014.00563 (PMC4230168; doi:10.3389/fmicb.2014.00563)
Supplement: Supplementary file 6 [file environment-heatmap_analysis-kulczynski.pdf › HTML-OUTPUT/environment-heatmap_analysis-kulczynski.pdf]

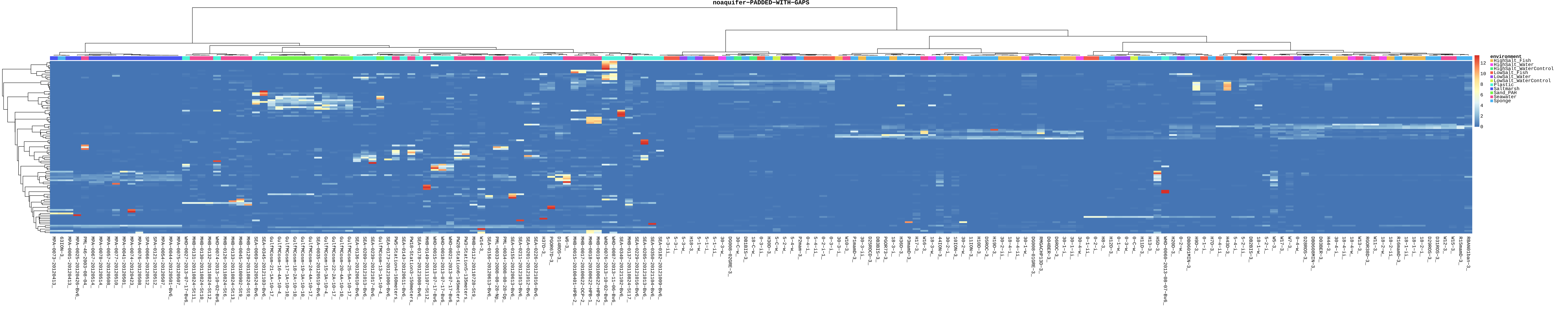
noaquifer-PADDED-WITH-GAPS
environment
HighSalt_Fish
HighSalt_Water
HighSalt_WaterControl
LowSalt_Fish
LowSalt_Water
LowSalt_WaterControl
Plastic
Saltmarsh
Sand_PAH
Seawater
Sponge
12
10
8
6
4
2
0
