# Supplementary material for: Oligotyping reveals community level habitat selection within the genus Vibrio
Source: Front Microbiol. 2014 Nov 13;5:563. doi: 10.3389/fmicb.2014.00563 (PMC4230168; doi:10.3389/fmicb.2014.00563)
Supplement: Supplementary file 6 [file DataSheet2.ZIP › HTML-OUTPUT/oligo_CGTCCACCGAAGG.html]

noaquifer-PADDED-WITH-GAPS :: CGTCCACCGAAGG


Oligotype CGTCCACCGAAGG in "noaquifer-PADDED-WITH-GAPS"

Page that explains diversity within an oligotype.

Main Analysis Page | Oligotyping.org

Unique Sequences Curve

» Abundance curve of unique sequences within CGTCCACCGAAGG, along with entropy values associated with these new set of alignments.

Distribution Among Samples

» Graphs below aim to give an idea about the distribution of unique sequences within CGTCCACCGAAGG among all samples.

This graph is here to remind the total number of reads for each sample that underwent the oligotyping analysis:

This graph shows the actual counts and distribution of unique sequences concealed within CGTCCACCGAAGG. By hovering over dots on the graph you can see the distribution of the first 20 sequences. If there are cases where the number of unique read #1 is competing with the number of other unique reads, it would mean that there is too much diversity trapped in this oligotype and needs to be decomposed by increasing the number of components. Sequences section below might help you decide which components might be appropriate to include.

This graph is similar to the one above, but normalized per sample by percent. Again, this should give you an idea about the concealed diversity.

Sequences

» First 20 Unique sequences

b **CGGCCATTATCAG** |||||||||||||||||||||||||||||||||||||||||||||||||||||||||||||||||||||||||| 

512    **CGTCCACCGAAGG** *b* TACTCTTGACATCCAGAGAATCCAGCGGAGACGCAGGAGTGCCTTAGGGAGCTCTGAGAC--------------

5    **CGTCCACCGAAGG** *b* TACTCCTGACATCCAGAGAATCCAGCGGAGACGCAGGAGTGCCTTAGGGAGCTCTGAGAC--------------

5    **CGTCCACCGAAGG** *b* TACTCTTGACATCCAGAGAATCCAGCGGAGACGCAGGAGCGCCTTAGGGAGCTCTGAGAC--------------

4    **CGTCCACCGAAGG** *b* TACTCTTGACATCCAGAGGATCCAGCGGAGACGCAGGAGTGCCTTAGGGAGCTCTGAGAC--------------

4    **CGTCCACCGAAGG** *b* TACTCTTGACATCCAGAGAATCCAGCGGGGACGCAGGAGTGCCTTAGGGAGCTCTGAGAC--------------

3    **CGTCCACCGAAGG** *b* TACTCTTGACATCCAGGGAATCCAGCGGAGACGCAGGAGTGCCTTAGGGAGCTCTGAGAC--------------

3    **CGTCCACCGAAGG** *b* TACTCTCGACATCCAGAGAATCCAGCGGAGACGCAGGAGTGCCTTAGGGAGCTCTGAGAC--------------

3    **CGTCCACCGAAGG** *b* TACTCTTGACATCCAGAGAATCCAGCGGAGGCGCAGGAGTGCCTTAGGGAGCTCTGAGAC--------------

3    **CGTCCACCGAAGG** *b* TACTCTTGACATCCGGAGAATCCAGCGGAGACGCAGGAGTGCCTTAGGGAGCTCTGAGAC--------------

2    **CGTCCACCGAAGG** *b* TACTCTTGACATCCAGAGAATCCAGCGGAGACGCAGGAGTGCCTTAGGGAGCCCTGAGAC--------------

2    **CGTCCACCGAAGG** *b* TACTCTTGACATCCAGAGAATCCAGCGGAGACGCAGGAGTGCCTTAGGGAGCTCTGGGAC--------------

2    **CGTCCACCGAAGG** *b* TACTCTTGACATCCAGAGAATCCAGCGGAGACGCGGGAGTGCCTTAGGGAGCTCTGAGAC--------------

2    **CGTCCACCGAAGG** *b* TACTCTTGACATCCAGAGAATCCAGCGGAGACGCAGGAGTGCCTTAGGGAGCTCTGAGTC--------------

2    **CGTCCACCGAAGG** *b* TACTCTTGACATCCAGAGAATCCAGCGGAGACGCAGGAGTGCCTCAGGGAGCTCTGAGAC--------------

2    **CGTCCACCGAAGG** *b* TACTCTTGACATCCAGAGAATCCAGCGGAGACGCAGGAGTGCCTTAGGGGGCTCTGAGAC--------------

2    **CGTCCACCGAAGG** *b* TACTCTTGACATCCAGAGAATCCAGCGGAGACGCAGGAGTGCCCTAGGGAGCTCTGAGAC--------------

1    **CGTCCACCGAAGG** *b* TACTCTTGACATCCAGAGAATCCAGCGGAGACGCAGGAGTGCCTTAGGGAGCTCTGTGAC--------------

1    **CGTCCACCGAAGG** *b* TACTCTTGACATCCTGAGAATCCAGCGGAGACGCAGGAGTGCCTTAGGGAGCTCTGAGAC--------------

1    **CGTCCACCGAAGG** *b* TACTCTTGACATCCAGAGAATCCAGCGGAGACGCAGTAGTGCCTTAGGGAGCTCTGAGAC--------------

1    **CGTCCACCGAAGG** *b* TACTCTTGACATCCAGAGAATCCAGCGGAGACGCAGGAGTGCCTTAGGGAGTTCTGAGAC--------------

BLAST Hits

» Alignments obtained for the most frequent sequence of CGTCCACCGAAGG are shown below.

*BLAST results are missing.*

◀ Previous Oligotype :: Main Page :: Next Oligotype ▶ *(48 of 99)*

For questions and comments: meren / mbl.edu
